# Flavonol tri- and tetraglycosides from *Albizia guachapele* Baker: isolation and biological evaluation

**DOI:** 10.1039/d6ra03441a

**Published:** 2026-07-20

**Authors:** Mahmoud Sallam, Ahmed M. Metwaly, Fatma G. Amin, Ibrahim H. Eissa, Ehab A. Ragab

**Affiliations:** a Pharmacognosy and Medicinal Plants Department, Faculty of Pharmacy (Boys), Al-Azhar University Cairo 11884 Egypt m.sallam@azhar.edu.eg ar_ehab@yahoo.com; b Physics Department, Faculty of Science, Alexandria University Alexandria Egypt; c Pharmaceutical Medicinal Chemistry & Drug Design Department, Faculty of Pharmacy (Boys), Al-Azhar University Cairo 11884 Egypt

## Abstract

Phytochemical investigation of the leaves of *Albizia guachapele* Baker (Fabaceae) led to the isolation and identification of two previously unreported kaempferol tetraglycosides, designated Guachapins A and B, together with ten known compounds. Their structures were established by interpretation of spectral data, including UV, ESI-MS, and NMR as kaempferol-3-*O*-β-d-glucopyranosyl-(1→4)-α-l-rhamnopyranosyl-(1→6)-*O*-[α-l-rhamnopyranosyl-(1→2)]-β-d-galactopyranoside, namely Guachapin A (3) and kaempferol-3-*O*-β-d-glucopyranosyl-[6-*O-E*-feruloyl]-(1→4)-α-l-rhamnopyranosyl-(1→6)-*O*-[α-l-rhamnopyranosyl-(1→2)]-β-d-galactopyranoside, namely Guachapin B (4). To provide mechanistic context for the antiproliferative activity of the major known isolate, mauritianin (1), a Topo II-focused *in silico* and cell-based mechanistic evaluation was conducted here for the first time for this compound. Compound 1 was initially evaluated through molecular docking and molecular dynamics (MD) simulation against topoisomerase II (Topo II), revealing a stable and favorable binding interaction at the enzyme's active site. These findings were further supported by *in vitro* assessment of Topo II alpha protein expression, which revealed a concentration-dependent reduction in intracellular Topo II alpha levels. Collectively, these convergent *in silico* and cell-based findings identify compound 1 as a Topo II-related antiproliferative lead candidate, displaying a phenotypic profile (G2/M arrest and predominant late apoptosis) consistent with a Topo II-mediated mechanism of cytotoxicity. Direct mechanistic confirmation through enzymatic and DNA damage assays is identified as a priority for future investigation.

## Abstract 2

Two new kaempferol tetraglycosides were isolated from *Albizia guachapele*; mauritianin inhibits topo II, suppresses MCF-7 proliferation, and induces G2/M arrest and apoptosis.

## Graphical abstract

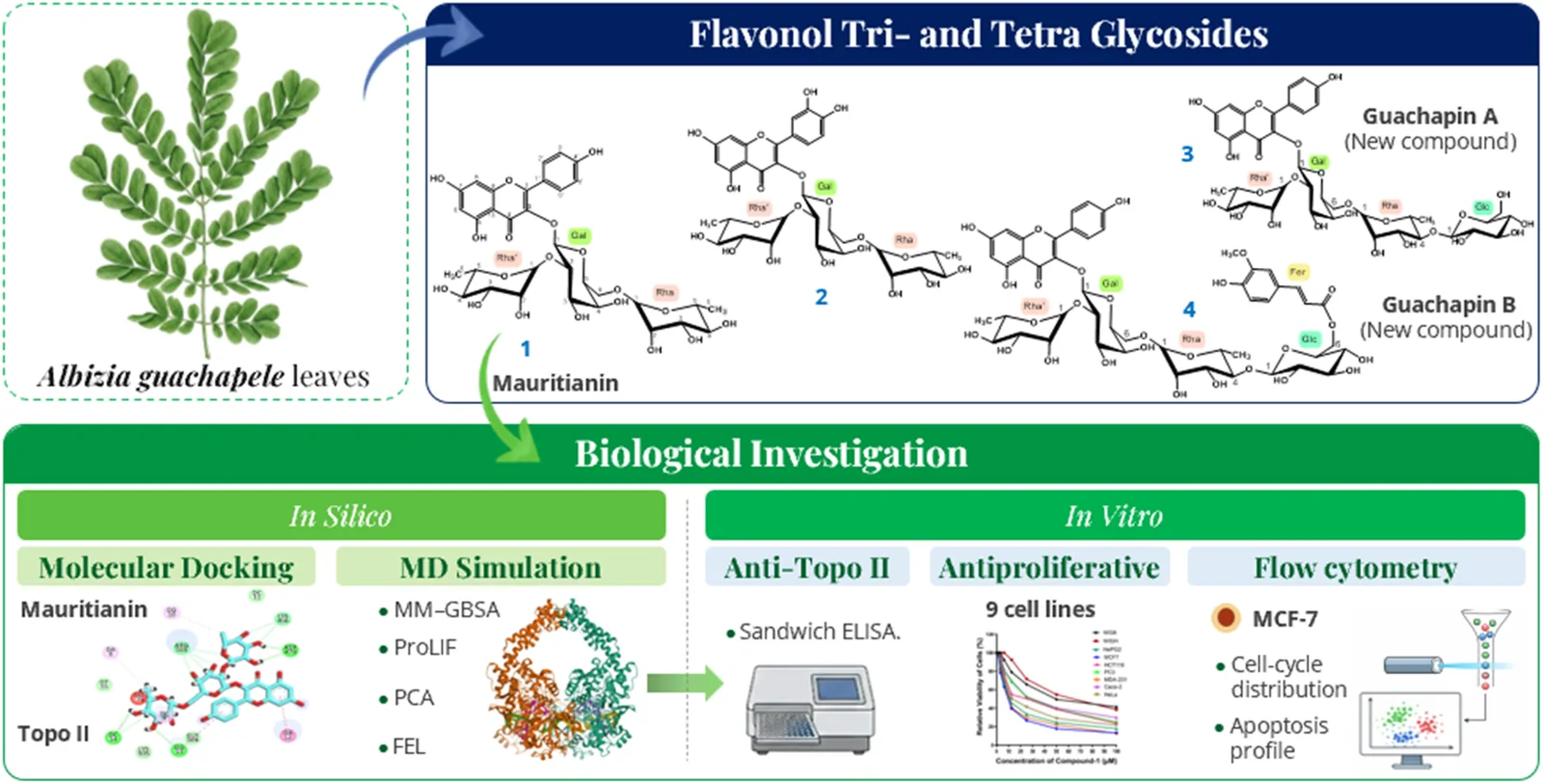

## 1. Introduction

*Albizia* represents a well-known genus of the family Fabaceae, encompassing approximately 150 species that are mainly shrubs and trees distributed across tropical and subtropical areas of Africa, Asia, Australia, and America.^1^ Traditionally, the genus was classified under the subfamily Mimosoideae. However, in 2017, the Legume Phylogeny Working Group (LPWG) established a revised taxonomic reclassification of Fabaceae, in which the former subfamily Mimosoideae was included within the subfamily Caesalpinioideae and is now recognized as the mimosoid clade.^2^*Albizia* spp. are rich in various phytochemicals, primarily including saponins, flavonoids, lignans, alkaloids, and phenolic glycosides. These constituents are primarily responsible for the wide range of pharmacological activities associated with the genus, such as anticancer, immunomodulatory, anti-inflammatory, antimicrobial, antidepressant, antianxiety, antidiabetic, and hepatoprotective effects.^1^

Egypt's climate, especially in some subtropical and arid regions, supports the growth of *Albizia* species that are adaptable to such environments. Several species of *Albizia* are cultivated in Egypt for ornamental and ecological purposes, although exact numbers may vary depending on the region and context of cultivation. The most cultivated species include *A. julibrissin*, *A. lebbeck*, *A. procera*, *A. odoratissima*, *A*. anthelmintica, *A. lucida*, *A. richardiana*, *A. myriophylla*, *A*. chinensis, *A. lophantha*, and *A. guachapele*.^3–5^

*A. guachapele* is widely distributed from southern Mexico through Central America, primarily along the Pacific coast, extending to Panama, and is commonly cultivated as a multipurpose tree.^6^ More recently, it has been introduced into Egypt for ornamental purposes. Despite its broad distribution and multipurpose use, *A. guachapele* remains relatively underexplored, particularly with respect to its phytochemical composition and pharmacological potential.

In this study, a comprehensive phytochemical investigation of *A. guachapele* was conducted, resulting in the identification of five flavonoid glycosides (1–5), including two previously undescribed kaempferol tetraglycosides (3 and 4), along with two lignans (6 and 7) and five additional known compounds (8–12). The chemical structures of the isolated compounds were determined through detailed spectroscopic investigations. To explore the biological relevance of selected constituents, compound 1 was prioritized for *in silico* evaluation against Topo II as a primary computational target, based on the presence of a planar aromatic pharmacophore and hydrogen-bond donor/acceptor features shared with established Topo II-active agents, as well as published precedent for kaempferol-class compounds at this target.^7^ Taken together, the chemical and biological results expand current knowledge of the secondary metabolite profile of this *Albizia* species and underscore its potential as a source of biologically active natural compounds.

## 2. Experimental

### 2.1. General experimental procedures

^1^H and ^13^C NMR spectra were recorded on a Bruker FT-NMR Spectrometer (Avance III HD console) at 400 MHz and 100 MHz, respectively. Chemical shifts (*δ*) are reported in ppm relative to tetramethylsilane (TMS) and coupling constants (*J*) are expressed in Hz. Electrospray ionization mass spectrometry (ESI-MS) in positive and negative ion modes was performed on a XEVO TQD triple quadrupole mass spectrometer (Waters Corporation, Milford, MA, U.S.A), using an ACQUITY UPLC BEH C18 column. UV spectra were acquired on a Shimadzu UV-1800 double-beam spectrophotometer (Shimadzu, Kyoto, Japan) over 190–1100 nm. Detailed instrumental parameters and acquisition settings are provided in the SI.

### 2.2. Plant material

Leaves of *A. guachapele* were harvested in February 2021 from Orman Botanical Garden, Giza governorate, Egypt (30°01′45″N 31°12′47″E/30.02917°N 31.21306°E). Botanical identification was kindly performed by Mrs Teresa Labib, Consultant of Taxonomy at the Ministry of Agriculture. A voucher specimen (Ag-2021) has been stored in the Department of Pharmacognosy and Medicinal Plants, Faculty of Pharmacy (Boys), Al-Azhar University, Nasr City, Cairo.

### 2.3. Extraction and isolation

A total of 2.1 kg of air-dried, powdered leaves of *A. guachapele* were exhaustively extracted at room temperature by maceration with 70% MeOH (3 × 10 L). The combined methanolic extracts were evaporated under reduced pressure at 40 °C to yield 285.5 g of crude residue. The resulting concentrated methanolic extract was then suspended in 500 mL distilled water and subjected to polarity-guided fractionation using *n*-hexane, dichloromethane (DCM) and *n*-butanol (*n*-BuOH) to yield 22.3, 8 and 90 g, respectively.^8^ The *n*-BuOH fraction (30 g) was initially fractionated by column chromatography (CC) over Diaion HP-20 (4.4 × 100 cm), utilizing a stepped gradient of H_2_O–MeOH (100 : 0 to 0 : 100, v/v) to afford five main fractions (Fr. A–E). An aliquot of fraction B (567 mg) was subsequently purified by RP C-18 CC (1.5 × 20 cm) utilizing an aqueous methanol gradient to yield five sub-fractions (B1–B5). Subsequent purification of Fr. B2 (686 mg) over Sephadex LH-20 (2 × 30 cm) with MeOH yielded 11 (25 mg) and 12 (13 mg), whereas Fr. B3 (416 mg) afforded compounds 2 (8 mg), and 3 (10 mg). Fraction C (9 g) was chromatographed over a Sephadex LH-20 column (3 × 70 cm) eluted with MeOH, resulting in the isolation of 1 (70 mg), 4 (7 mg), and 5 (94 mg). The dichloromethane (DCM) fraction (8 g) was subjected to silica gel CC (100 g, 75 × 5 cm) utilizing an *n*-hexane–EtOAc gradient system, yielding major fractions A through E. Fraction A (889 mg) underwent crystallization from hot methanol to produce sub-fraction A1 (90 mg), which was subsequently purified over a Sephadex column (MeOH) to afford 8 (65 mg). Fraction C (1.6 g) was further fractionated using RP C-18 solid-phase extraction (SPE) with an H_2_O–MeOH gradient, resulting in five sub-fractions, C1–C5. Sub-fraction C2 (350 mg) was purified *via* Sephadex (MeOH) to yield a mixture of 9 & 10 (10 mg), while sub-fraction C4 (498 mg) was processed through the same stationary phase to isolate 6 (15 mg) and 7 (10 mg).

#### 2.3.1. Kaempferol-3-*O*-(2,6-di-*O*-α-l-rhamnopyranosyl)-β-*d*-galactopyranoside, mauritianin (1)

Yellow amorphous powder; UV *λ*_max_ (nm) MeOH: 264, 347; NAOMe: 273, 400; AlCl_3_: 274, 402; AlCl_3_/HCl: 274, 399; NaOAc: 274, 385; NaOAc/H_3_BO_3_: 265, 350; ESI-MS *m*/*z* 741 [M + H]^+^, 595 [M + H-Rha]^+^, 449 [M + H-2Rha]^+^, and 287 [M + H-2Rha-Gal]^+^; ^1^H NMR (400 MHz, DMSO-*d*_6_): 6.13 (1H, d, *J* = 1.6 Hz, H-6), 6.34 (1H, d, *J* = 1.6 Hz, H-8), 8.03 (2H, d, *J* = 8.8 Hz, H-2′,6′), 6.85 (2H, d, *J* = 8.8 Hz, H-3′,5′), 12.66 (brs, 5-OH), 5.56 (1H, d, *J* = 7.7, H-1 of Gal), 3.78 (1H, m, H-2 of Gal), 3.59 (1H, m, H-3 of Gal), 3.59 (1H, m, H-4 of Gal), 3.56 (1H, m, H-5 of Gal), 3.56 (1H, m, H-6a of Gal), 3.22 (1H, m, H-6b of Gal), 4.36 (1H, s, H-1 of Rha), 3.38 (1H, m, H-2 of Rha), 3.30 (1H, dd, *J* = 9.3, 3.3, H-3 of Rha), 3.09 (1H, t, *J* = 9.3, H-4 of Rha), 3.33 (1H, d, *J* = 6.2, H-5 of Rha), 1.05 (3H, d, *J* = 6.2, H-6 of Rha), 5.05 (1H, s, H-1 of Rha′), 3.75 (1H, m, H-2 of Rha′), 3.50 (1H, dd, *J* = 9.3, 3.2, H-3 of Rha′), 3.13 (1H, t, *J* = 9.3, H-4 of Rha′), 3.77 (1H, m, H-5 of Rha′), 0.79 (3H, d, *J* = 6.0, H-6 of Rha′); ^13^C NMR (100 MHz, DMSO-*d*_6_): 156.55 (C-2), 132.58 (C-3), 177.10 (C-4), 161.23 (C-5), 99.23 (C-6), 165.9 (C-7), 94.00 (C-8), 156.07 (C-9), 103.35 (C-10), 121.03 (C-1′), 130.78 (C-2′/6′), 115.13 (C-3′/5′), 159.90 (C-4′), 99.01 (C-1 of Gal), 74.92 (C-2 of Gal), 73.87 (C-3 of Gal), 68.58 (C-4 of Gal), 73.34 (C-5 of Gal), 65.17 (C-6 of Gal), 100.12 (C-1 of Rha), 70.46 (C-2 of Rha), 70.73 (C-3 of Rha), 71.95 (C-4 of Rha), 68.33 (C-5 of Rha), 17.97 (C-6 of Rha), 100.64 (C-1 of Rha′), 70.46 (C-2 of Rha′), 70.65 (C-3 of Rha′), 71.95 (C-4 of Rha′), 68.22 (C-5 of Rha′), 17.31 (C-6 of Rha′).

#### 2.3.2. Quercetin-3-*O*-(2,6-di-*O*-α-l-rhamnopyranosyl)-β-d-galactopyranoside (2)

Yellow amorphous powder; UV *λ*_max_ (nm) MeOH: 256, 355; NAOMe: 272, 405; AlCl_3_: 275, 431; AlCl_3_/HCl: 272, 397; NaOAc: 268, 391; NaOAc/H_3_BO_3_: 261, 376; ESI-MS *m*/*z* 757 [M + H]^+^, 611 [M + H-Rha]^+^, and 303 [M + H-2Rha-Gal]^+^; ^1^H NMR (400 MHz, DMSO-*d*_6_): 6.19 (1H, d, *J* = 2.0 Hz, H-6), 6.40 (1H, d, *J* = 2.0 Hz, H-8), 7.50 (1H, d, *J* = 2.2 Hz, H-2′), 6.82 (1H, d, *J* = 8.5 Hz, H-5′), 7.69 (1H, dd, *J* = 8.5, 2.2 Hz, H-6′), 12.71 (brs, 5-OH), 5.59 (1H, d, *J* = 7.7, H-1 of Gal), 3.82 (1H, m, H-2 of Gal), 3.62 (1H, m, H-3 of Gal), 3.60 (1H, m, H-4 of Gal), 3.56 (1H, m, H-5 of Gal), 3.57 (1H, m, H-6a of Gal), 3.22 (1H, m, H-6b of Gal), 4.40 (1H, s, H-1 of Rha), 3.37 (1H, m, H-2 of Rha), 3.29 (1H, dd, *J* = 9.2, 2.8, H-3 of Rha), 3.09 (1H, t, *J* = 9.2, H-4 of Rha), 3.35 (1H, m, H-5 of Rha), 1.06 (3H, d, *J* = 6.2, H-6 of Rha), 5.07 (1H, s, H-1 of Rha′), 3.74 (1H, m, H-2 of Rha′), 3.50 (1H, dd, *J* = 9.2, 2.8, H-3 of Rha′), 3.14 (1H, t, *J* = 9.2, H-4 of Rha′), 3.78 (1H, m, H-5 of Rha′), 0.81 (3H, d, *J* = 6.2, H-6 of Rha′); ^13^C NMR (100 MHz, DMSO-*d*_6_): 156.72 (C-2), 133.30 (C-3), 177.67 (C-4), 161.71 (C-5), 99.15 (C-6), 164.65 (C-7), 93.96 (C-8), 156.66 (C-9), 104.32 (C-10), 121.56 (C-1′), 116.16 (C-2′), 145.33 (C-3′), 148.87 (C-4′), 115.62 (C-5′), 122.50 (C-6′), 99.45 (C-1 of Gal), 75.24 (C-2 of Gal), 74.36 (C-3 of Gal), 69.01 (C-4 of Gal), 73.74 (C-5 of Gal), 65.35 (C-6 of Gal), 100.45 (C-1 of Rha), 70.89 (C-2 of Rha), 71.04 (C-3 of Rha), 72.35 (C-4 of Rha), 68.75 (C-5 of Rha), 18.40 (C-6 of Rha), 101.00 (C-1 of Rha′), 70.89 (C-2 of Rha′), 71.04 (C-3 of Rha′), 72.35 (C-4 of Rha′), 68.64 (C-5 of Rha′), 17.37 (C-6 of Rha′).

#### 2.3.3. Kaempferol-3-*O*-β-d-glucopyranosyl-(1→4)-α-l-rhamnopyranosyl-(1→6)-*O*-[α-l-rhamnopyranosyl-(1→2)]-β-d-galactopyranoside, guachapin A (3)

Yellow amorphous powder; UV *λ*_max_ (nm) MeOH: 266, 348; NAOMe: 274, 401; AlCl_3_: 273, 400; AlCl_3_/HCl: 272, 395; NaOAc: 275, 384; NaOAc/H_3_BO_3_: 266, 350; ESI-MS *m*/*z* 903 [M + H]^+^, 757 [M + H-Rha]^+^, 595 [M + H-Rha-Glc]^+^, 449 [M + H-2Rha-Glc]^+^, and 287 [M + H-2Rha-Glc-Gal]^+^; ^1^H NMR (400 MHz, DMSO-*d*_6_) and ^13^C NMR (100 MHz, DMSO-*d*_6_): see Table 1. 2D NMR spectra (COSY, ROESY, HSQC, HMBC, TOCSY, and HSQC-TOCSY): detailed data are provided SI. HRMS analysis could not be performed due to instrument unavailability; structural assignment is based on the comprehensive spectroscopic evidence described above (UV, ESI-MS/MS fragmentation, 1D and 2D NMR), which provided consistent and unambiguous structural assignments.

**Table 1:** ^1^H and ^13^C NMR spectral data for flavonol tetraglycosides 3 and 4 in DMSO-*d*_6_ (*δ* in ppm, *J* in Hz)

No.	3		4		No.	3		4	
	*δ*_H_	*δ*_C_	*δ*_H_	*δ*_C_ a		*δ*_H_	*δ*_C_	*δ*_H_	*δ*_C_ a
**Aglycone**					**Glc**				
2	—	155.11	—	—	1	4.32 d (7.8)	104.70	4.36 d (8.8)	105.17
3	—	132.26	—	—	2	2.98 t (8.4)	74.85	3.02 m	74.16
4	—	176.49	—	—	3	3.15 m	76.64	3.19 m	76.75
5	—	161.06	—	162.00	4	3.05 m	70.71	3.11 m	70.43
6	5.98 brs	100.30	6.18 d (2.1)	99.18	5	3.07 m	77.00	3.41^b^	74.14
7	—	163.50	—	164.80	6a	3.65 brd (10.8)	61.13	4.21 m	64.11
8	6.18 brs	94.39	6.40 d (1.9)	94.01	6b	3.43 m		4.01 m	
9	—	156.73	—	156.27	**Rha′**				
10	—	102.03	—	103.76	1	5.04 s	100.58	5.04 s	100.61
1′	—	121.07	—	121.34	2	3.74 m	70.57	3.73 m	70.93
2′	8.00 d (8.8)	130.60	8.03 d (8.8)	131.22	3	3.51 m	70.68	3.50 m	70.78
3′	6.83 d (8.8)	115.11	6.84 d (8.8)	115.68	4	3.14 m	71.91	3.14 m	72.19
4′	—	159.87	—	160.37	5	3.80 m	68.19	3.77 m	68.54
					6	0.78 d (6.1)	17.30	0.77 d (6.1)	17.55
5′	6.83 d (8.8)	115.11	6.84 d (8.8)	115.68	**Fer**				
6′	8.00 d (8.8)	130.60	8.03 d (8.8)	131.22	1			—	125.50
5-OH	12.63 brs	—	12.67 brs	—	2			7.29 d (1.6)	111.60
**Gal**					3			—	147.22
1	5.56 d (7.7)	98.96	5.55 d (7.6)	99.15	4			—	149.81
2	3.78 m	75.04	3.78 m	75.85	5			6.78 d (8.1)	115.95
3	3.61 m	74.59	3.61 m	74.43	6			7.09 dd (8.1, 1.7)	123.59
4	3.58 m	68.42	3.56 m	68.90	7			7.52 d (16.0)	145.88
5	3.54 m	73.05	3.53 m	73.40	8			6.44 d (16.0)	114.65
6a	3.59 m	64.77	3.57 m	65.19	9			—	167.0
6b	3.18 m		3.18 m		OMe			3.80 s	55.73
**Rha**									
1	4.38 s	99.81	4.38 s	99.90					
2	3.46 m	69.98	3.44 m	70.30					
3	3.54 m	70.57	3.53 m	71.03					
4	3.37 m	82.36	3.33^b^	83.22					
5	3.47 m	66.73	3.45 m	66.84					
6	1.15 d (6.0)	17.81	1.09 d (6.0)	18.08					

a Obtained from HSQC and HMBC spectra.

b Masked by water peak.

#### 2.3.4. Kaempferol-3-*O*-β-d-glucopyranosyl-[6-*O-E*-feruloyl]-(1→4)-α-l-rhamnopyranosyl-(1→6)-*O*-[α-l-rhamnopyranosyl-(1→2)]-β-d-galactopyranoside, guachapin B (4)

Yellow amorphous powder; UV *λ*_max_ (nm) MeOH: 267, 333; NAOMe: 270, 390; AlCl_3_: 270, 393; AlCl_3_/HCl: 270, 373; NaOAc: 275, 340; NaOAc/H_3_BO_3_: 267, 333; ESI-MS *m*/*z* 1079 [M + H]^+^, 933 [M + H-Rha]^+^, 449 [M + H-2Rha-Glc-Fer]^+^, and 287 [M + H-2Rha-Glc-Fer-Gal]^+^; ^1^H NMR (400 MHz, DMSO-*d*_6_) and ^13^C NMR (100 MHz, DMSO-*d*_6_): see Table 1. 2D NMR spectra (ROESY, HSQC, HMBC, TOCSY, and HSQC-TOCSY): detailed data are provided SI. HRMS analysis could not be performed due to instrument unavailability; structural assignment is based on the comprehensive spectroscopic evidence described above (UV, ESI-MS/MS fragmentation, 1D and 2D NMR), which provided consistent and unambiguous structural assignments.

#### 2.3.5. Quercetin 3-*O*-α-l-rhamnopyranoside, quercitrin (5)

Yellow crystals; ESI-MS *m*/*z* 449 [M + H]^+^, and 303 [M + H-Rha]^+^; ^1^H NMR; ^13^C NMR: detailed data are provided SI.

#### 2.3.6. Pinoresinol (6)

Amorphous powder; ESI-MS *m*/*z* 359 [M + H]^+^, 717 [2M + H]^+^, 341 [M + H–H_2_O]^+^, 323 [M + H–2H_2_O]^+^; ^1^H NMR; ^13^C NMR: detailed data are provided SI.

#### 2.3.7. Pinoresinol dimethyl ether, eudesmin (7)

Amorphous powder; ESI-MS *m*/*z* 387 [M + H]^+^, 369 [M + H–H_2_O]^+^, 351 [M + H–2H_2_O]^+^; ^1^H NMR; ^13^C NMR: detailed data are provided SI.

#### 2.3.8. Lupeol (8)

White needle crystals; ESI-MS *m*/*z* 426 [M + H]^+^; ^1^H NMR; ^13^C NMR: detailed data are provided SI.

#### 2.3.9. Loliolide (9) and 3-hydroxy-5,6-epoxy-β-ionone (10)

Colorless oil; ESI-MS *m*/*z* 197 [M + H]^+^ and 225 [M + H]^+^ respectively; ^1^H NMR; ^13^C NMR: detailed data are provided SI.

#### 2.3.10. Adenosine (11)

Colorless needles; ESI-MS *m*/*z* 268 [M + H]^+^; ^1^H NMR; ^13^C NMR: detailed data are provided SI.

#### 2.3.11. Tryptophan (12)

Yellowish-white solid; ESI-MS *m*/*z* 205 [M + H]^+^; ^1^H NMR; ^13^C NMR: detailed data are provided SI.

### 2.4. Acid hydrolysis

Each compound (1–4; 5 mg each) was refluxed with 2 M HCl in methanol (5 mL) at 80 °C for 2 h. After evaporation to dryness, the residue was dissolved in distilled water (10 mL) and partitioned with EtOAc (3 × 20 mL). The combined organic extracts were concentrated to yield the aglycones, which were identified by co-TLC against kaempferol and quercetin standards. The aqueous layer was neutralized with 2 N KOH, concentrated under reduced pressure at 40 °C to 1 mL, and the resulting sugars were identified by co-TLC with authentic standards.^9,10^ Further method details are provided in the SI.

### 2.5. Biological studies

#### 2.5.1. *In silico* studies

##### 2.5.1.1. Molecular docking

The crystal structure of the DNA–Topo II complex (PDB ID: 3QX3) was retrieved from the Protein Data Bank. Crystallographic water molecules were removed and a single chain with the co-crystallized ligand was retained. Protein protonation was performed using GB/VI electrostatics with a 15 Å distance cut-off, dielectric constants of 2 (protein) and 80 (solvent), and van der Waals functional form 800R3 with a 10 Å cut-off. Energy minimization was conducted using the AM1 Hamiltonian followed by optimization with the MMFF94x force field. The active site was defined as all residues within 5 Å of the co-crystallized ligand. Compound 1 and doxorubicin were sketched using ChemBioDraw Ultra 14.0, converted to 3D, protonated, and energy-minimized using the MM2 force field (10 000 steps, 2 fs intervals). The co-crystallized ligand (etoposide) was extracted from the prepared DNA–Topo II complex (PDB: 3QX3), re-docked into the binding site under identical protocol conditions, and the RMSD between the top-ranked redocked pose and the crystallographic pose was calculated as 0.491 Å.^11^ Docking was performed generating 30 poses per ligand using the ASE scoring function with rigid receptor refinement; the highest-scoring pose was selected for analysis. Results were visualized using Discovery Studio 4.0. The docking methodology followed established procedures reported previously.^12^ Full details of ligand structure generation, protein preparation, protonation parameters, force fields, cut-offs, minimization procedures, and docking protocol validation are provided in the SI.

##### 2.5.1.2. Molecular dynamics (MD) simulation

System preparation was carried out using CHARMM-GUI solution builder. The complex was solvated with TIP3P water in a 10 nm cubic box with a 1 nm buffer distance, neutralized with NaCl (0.154 M), and parametrized using the CHARMM36m force field under periodic boundary conditions. Energy minimization was performed using the steepest descent algorithm until maximum force fell below 100 kJ mol^−1^ nm^−1^ or after 100 000 steps. Equilibration was conducted in two stages: NVT ensemble (V-rescale thermostat, 310 K), followed by NPT ensemble (V-rescale thermostat + Berendsen barostat, 310 K, 1 atm). The production run (NPT, 200 ns) employed a Nosé–Hoover thermostat (310 K) and Parrinello–Rahman barostat (1 atm). Equations of motion were propagated using the leapfrog integrator with a 1 fs time step during equilibration and 2 fs during production. The LINCS algorithm constrained hydrogen bond lengths; electrostatics were treated using Particle Mesh Ewald (PME) with a 1.2 nm cut-off. Trajectory frames were recorded every 0.1 ns (2000 total frames) and analyzed using VMD-TK scripts. Backbone root main square deviation (RMSD), ligand RMSD, radius of gyration (RoG), solvent-accessible surface area (SASA), root main square fluctuation (RMSF), hydrogen bond count, centroid distance, and ligand–amino acid interaction fingerprints (ProLIF) were computed from the trajectories.^13–23^ Full details of system setup, force fields, cut-offs, equilibration parameters, and analysis procedures are provided in the SI.

##### 2.5.1.3. Binding free energy calculation using molecular mechanics/generalized born surface area (MM-GBSA)

The binding free energies of the ligands were computed *via* the gmx_MMPBSA program applying the MM-GBSA approach. Decomposition analysis was carried out to determine residues within 1 nm of the ligand that contribute to binding affinity. Simulations were conducted with a solvation parameter (igb) of 5, an ionic strength of 0.154 M, and internal/external dielectric constants of 1.0 and 78.5, respectively. The calculations were based on 2000 frames from the MD trajectories. Detailed equations (eqn (1)–(6)) and the full computational procedure are provided in the SI.^24,25^

##### 2.5.1.4. Principal component analysis (PCA)

The motions of the α-carbons of the Topo II–DNA complex were evaluated using mass-weighted covariance matrices and principal component analysis (PCA).^24^ For combined and individual trajectory analyses, the final equilibrium frames were used as references during alignment. Eigenvectors corresponding to dominant atomic motions were obtained by diagonalizing the covariance matrix with gmx covar, and eigenvalues quantified the magnitude of motion, with the first principal component representing the largest variance. GROMACS gmx anaeig was used to analyze the essential subspace, including variance captured, scree plots, and eigenvector distributions. Cosine content (ci) was computed for the first ten PCs to assess meaningful dynamics *versus* random motion.^26–28^ Full details of covariance matrix construction, eigenvector analysis, and the cosine content calculation (eqn (7)) are provided in the SI.

##### 2.5.1.5. Free energy landscape (FEL)

To characterize the conformational energy landscape sampled by the predicted Mauritianin–Topo II–DNA complex during the MD simulation, free energy landscapes (FELs) were constructed from the trajectory.^27^ The identified energy minima represent preferred conformational states within the modelled system; they do not correspond to experimentally measured binding states or provide mechanistic evidence of Topo II inhibition. Two-dimensional FEL representations were constructed by projecting the system onto selected reaction coordinates, with probability distributions utilized to determine the relative free energies of different states. The gmx sham command in GROMACS was used for analysis. Full details of the FEL calculations, reaction coordinate selection, and equation (eqn (8)) are provided in the SI.

#### 2.5.2. In vitro studies

##### 2.5.2.1. Topoisomerase II enzyme inhibition assay

The effect of compound 1 (mauritianin) and doxorubicin (reference drug) on intracellular Topo II alpha protein levels was assessed using the Human Topo II Alpha ELISA Kit (ab288584, Abcam, Cambridge, UK), a 90 min, single-wash sandwich immunoassay based on SimpleStep ELISA® technology.^29,30^ This kit quantifies Topo II alpha protein in cell lysates and does not measure catalytic activity directly; a reduced immunoassay signal in compound-treated lysates may therefore reflect transcriptional or translational downregulation of Topo II alpha protein expression, enhanced proteasomal degradation, or steric interference with antibody–epitope recognition—none of which are mechanistically equivalent to direct enzymatic inhibition or cleavable-complex stabilization. Standard functional assays (including plasmid DNA relaxation or kinetoplast DNA decatenation) would be required to assess catalytic inhibition directly and are identified as a priority for future work.

Varying concentrations of mauritianin (0.1–1000 nM) were incubated with Topo II-containing cell lysates under kit-recommended conditions. Following incubation, wells were washed once and bound complexes were detected using HRP–TMB (horseradish peroxidase–tetramethylbenzidine) substrate; the colorimetric reaction was stopped and absorbance measured at 450 nm using a microplate reader. Assay sensitivity was 36.55 pg mL^−1^ per manufacturer's specification. The following controls were included in each assay run: (i) blank control—lysis buffer without cells or compound, used to establish background absorbance; (ii) vehicle control (negative control)—cell lysate from untreated MCF-7 cells supplemented with the equivalent volume of DMSO (≤0.1% v/v final concentration), serving as the 100% reference signal for percentage reduction calculations; and (iii) positive control—doxorubicin at 1 µM, a concentration well within the range documented to reduce Topo II alpha expression in MCF-7 cell lysates, included to confirm assay sensitivity and inter-run consistency.

##### 2.5.2.2. Cytotoxicity assay

Cytotoxic activity of compound 1 (mauritianin) was evaluated against a panel of human cell lines: normal human lung fibroblast (WI-38) and human amnion (WISH) as non-cancerous references, and hepatocellular carcinoma (HepG-2), cervical carcinoma (HeLa), prostate carcinoma (PC-3), mammary gland carcinoma (MCF-7), colorectal adenocarcinoma (Caco-2), colorectal carcinoma (HCT-116), and breast adenocarcinoma (MDA-MB-231) as malignant cell lines. All cell lines were obtained from ATCC and supplied through the Holding Company for Biological Products and Vaccines (VACSERA, Cairo, Egypt). Doxorubicin was used as a reference anticancer agent for comparative purposes.

Cells were routinely maintained in RPMI-1640 medium (Sigma, St. Louis, USA) supplemented with 10% fetal bovine serum (FBS; GIBCO, UK), 100 U mL^−1^ penicillin, and 100 µg mL^−1^ streptomycin at 37 °C in a humidified incubator with 5% CO2. Cytotoxicity was assessed using the MTT (3-(4,5-dimethylthiazol-2-yl)-2,5-diphenyltetrazolium bromide) colorimetric assay, based on the reduction of MTT by mitochondrial succinate dehydrogenase in metabolically active cells to yield insoluble purple formazan, the quantity of which is directly proportional to viable cell number.

For the assay, cells were seeded into 96-well plates at a density of 1.0 × 10^4^ cells per well and allowed to adhere for 48 h under standard culture conditions. Following adhesion, cells were treated with compound 1 or doxorubicin at varying concentrations (1.56–100 µM) and incubated for an additional 24 h. MTT solution (5 mg mL^−1^, 20 µL per well) was then added and incubated for 4 h to allow formazan crystal formation. Crystals were dissolved by addition of 100 µL DMSO per well, and absorbance was measured at 570 nm using a microplate reader (EXL 800, USA).

The following controls were included on each assay plate: (i) blank control—culture medium without cells, used to correct for background absorbance at 570 nm; (ii) vehicle control (negative control)—cells treated with DMSO at the maximum vehicle concentration used across all treatment wells (≤0.1% v/v), representing 100% cell viability; and (iii) positive control—doxorubicin at 1 µM, included on each plate to confirm inter-plate assay consistency.

Cell viability (%) was calculated as: (*A*_treated_/*A*_control_) × 100. The half-maximal inhibitory concentration (IC_50_)—defined as the concentration reducing cell viability by 50% relative to the vehicle control—was determined from dose–response curves plotted as cell viability (%) *versus* log concentration using GraphPad Prism (version 9.0). The selectivity index (SI) was calculated as: SI = IC_50_ (normal cell line)/IC_50_ (cancer cell line); SI ≥ 2 was considered indicative of selective cytotoxicity toward malignant cells. All experiments were conducted in triplicate technical wells within a single experimental run (*n* = 3 technical replicates); results are expressed as mean of triplicate measurements.

##### 2.5.2.3. Flow cytometry analysis

Flow cytometric analysis was performed following previously described protocols.^32^

###### 2.5.2.3.1. Cell cycle distribution

Using propidium iodide (PI) staining, the effect of compound 1 (mauritianin) on cell cycle progression was evaluated in MCF-7 cells. Cells were treated with mauritianin at its IC_50_ concentration for 48 h, harvested (1 × 10^6^ cells), washed with PBS, and preserved in 70% ethanol at −20 °C for a minimum of 12 h. The fixed cells were incubated in the dark with 50 µg mL^−1^ PI, 100 µg mL^−1^ RNase A, and 0.1% Triton X-100 for 30 min at 37 °C. Flow cytometry was carried out on a BD FACSAria III (488 nm excitation, 617/25 nm emission), with a minimum of 20 000 single-cell events recorded per sample. DNA histograms were processed using ModFit LT 5.0 with the Dean-Jett-Fox model to quantify the percentage of cells across G0/G1, S, and G2/M phases. Experiments were performed in three independent biological replicates (*n* = 3); results are expressed as mean ± SD.

###### 2.5.2.3.2. Apoptosis induction

To determine the pro-apoptotic potential of compound 1 (mauritianin), MCF-7 cells were analyzed *via* annexin V-FITC/PI dual staining (BD Pharmingen). Cells (5 × 10^5^) were harvested, washed with PBS, and suspended in binding buffer. Following the addition of annexin V-FITC and PI (5 µL each), the mixture was kept in the dark and incubated for 15 minutes at room temperature. Flow cytometric analysis was performed using a BD FACSAria III system, with single-staining and fluorescence-minus-one (FMO) controls ensuring precise gating and compensation. The absence of a positive apoptosis-inducing control is acknowledged as a methodological limitation that reduces within-experiment confirmation of assay sensitivity. Data were analyzed using FACSDiva 8.0.1 software, classifying cells into viable, apoptotic (early and late), and necrotic populations, enabling quantification of apoptosis and necrosis induced by mauritianin.^33^ Experiments were performed in three independent biological replicates (*n* = 3); results are expressed as mean ± SD.

#### 2.5.3. Statistical analysis

All biological experiments were performed in three independent biological replicates, each conducted in triplicate wells (*n* = 3), unless otherwise stated. Data are expressed as mean ± standard deviation (SD). IC_50_ and EC_50_ values were determined by nonlinear regression analysis of dose–response curves using GraphPad Prism (version 9.0, GraphPad Software, San Diego, CA, USA). Selectivity indices (SI) were calculated as the ratio of IC_50_ against normal cell lines (WI-38 or WISH) to IC_50_ against each cancer cell line. Statistical comparisons between groups were performed using one-way analysis of variance (ANOVA) followed by Tukey's post hoc test for multiple comparisons; an independent samples *t*-test was applied for pairwise comparisons where appropriate. A *p*-value <0.05 was considered statistically significant. Statistical significance in figures is denoted as: *p < 0.05, **p < 0.01, ***p < 0.001; ns = not significant. Error bars in all biological data figures represent mean ± SD (*n* = 3).

## 3. Results and discussion

### 3.1. Structure elucidation

**Fig. 1:**
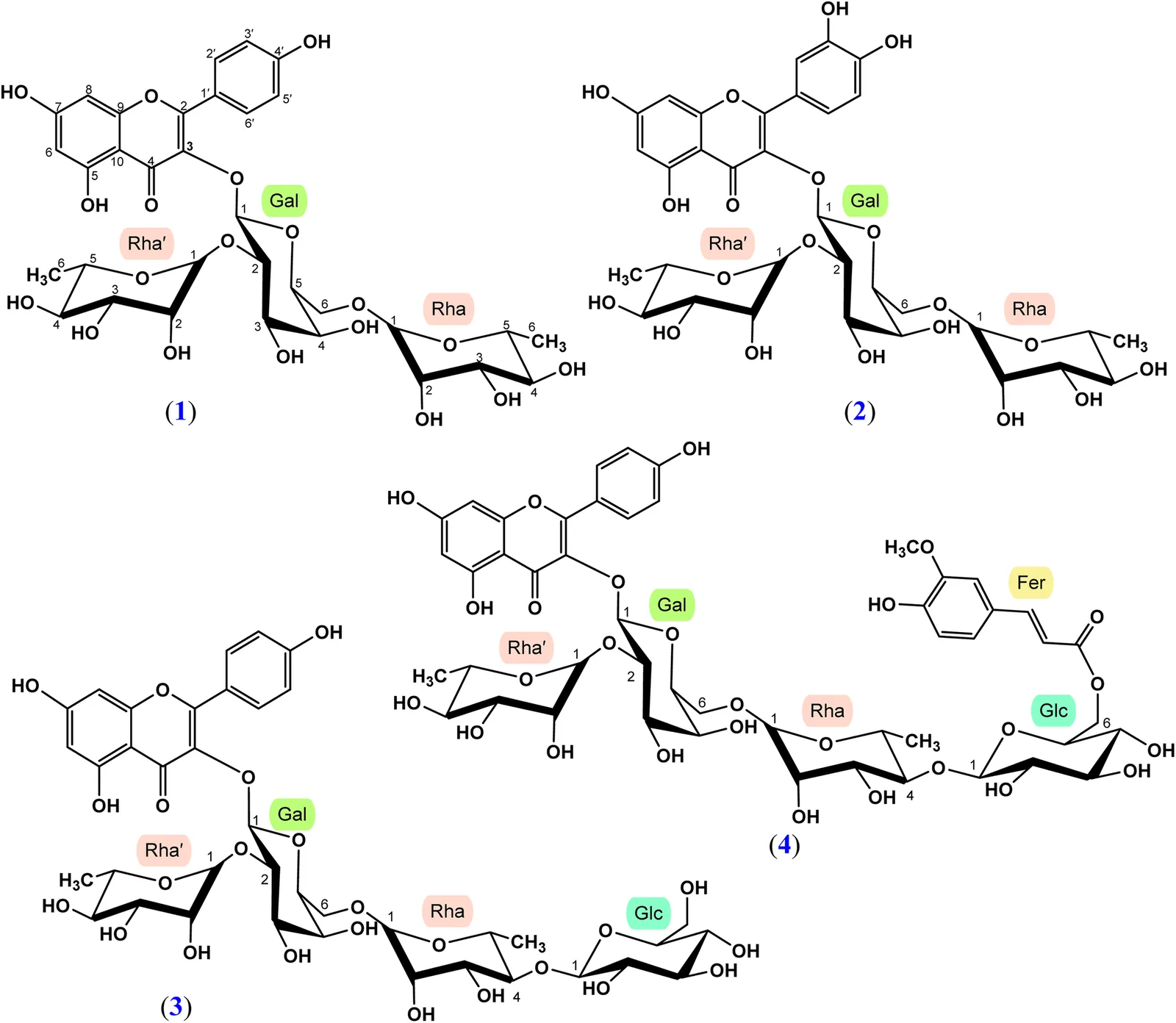
Chemical structures of flavonol tri- and tetraglycosides (1–4) from *A. guachapele* leaves.

Twelve compounds (1–12) were obtained from the dichloromethane and *n*-butanol extracts of *A. guachapele* leaves by a series of chromatographic methods. The structures of the two previously unreported compounds (3 and 4) (Fig. 1) were determined through comprehensive interpretation of their spectroscopic data, including ^1^H and ^13^C NMR, HMBC, HSQC, ROESY, COSY, TOCSY, HSQC-TOCSY experiments, together with UV and ESI-MS analyses. The chemical structures of ten previously described compounds (1, 2 and 5–12) were elucidated *via* comparative analysis of their ^1^H and ^13^C NMR spectra with reported literature data as kaempferol-3-*O*-(2,6-di-*O*-α-l-rhamnopyranosyl)-β-d-galactopyranoside (mauritianin, 1),^34,35^ quercetin-3-*O*-(2,6-di-*O*-α-l-rhamnopyranosyl)-β-d-galactopyranoside (2),^35,36^ quercetin 3-*O*-α-l-rhamnopyranoside (quercitrin, 5),^37–39^ pinoresinol (6),^40^ pinoresinol dimethyl ether (eudesmin, 7),^41–43^ lupeol (8),^44–47^ loliolide (calendin, 9),^48,49^ 3-hydroxy-5,6-epoxy-β-ionone (10),^48,49^ adenosine (11),^50–52^ and tryptophan (12).^53,54^

Guachapin A (3) was isolated as a yellow amorphous powder. Positive ion ESI-MS analysis showed an [M + H]^+^ ion peak at *m*/*z* 903, while negative ion ESI-MS displayed an [M − H]^−^ ion peak at *m*/*z* 901. Furthermore, fragment ion peaks were detected in the positive ion mode spectrum of compound 3 at *m*/*z* 757 [M + H-Rha]^+^, *m*/*z* 595 [M + H-Rha-Glc]^+^, 449 [M + H-2Rha-Glc]^+^ and 287 [M + H-2Rha-Glc-Gal]^+^, together with a fragment ion peak in the negative ion mode at *m*/*z* 755 [M − H-Rha]^−^, indicating one rhamnose and one glucose units are terminal sugars, thus confirming a branched oligosaccharide chain. UV analysis of 3 was consistent with a kaempferol 3-*O*-glycoside with a free 7-OH, evidenced by the +9 nm bathochromic shift in Band II upon NaOAc addition.^55^

The ^1^H NMR spectrum of 3 (Table 1) exhibited signals characteristic of a kaempferol aglycone: a chelated 5-OH singlet (*δ* 12.63), an AA′BB′ pattern for the para-disubstituted B ring (*δ* 8.00 and 6.83, each 2H, d, *J* = 8.8 Hz), and two A-ring protons at *δ* 5.98 and 6.18 (H-6 and H-8).^37^

The tetra-saccharide moiety of 3 were evidenced from the observed four anomeric proton signals at *δ* 5.56 (*J* = 7.7 Hz, H-1 of Gal), *δ* 5.04 (s, H-1 of Rha′), *δ* 4.38 (s, H-1 of Rha), and *δ* 4.32 (*J* = 7.8 Hz, H-1 of Glc), corresponding to one galactose, two rhamnoses, and one glucose, respectively. The two highly upfield ^1^H NMR doublets at *δ* 0.78 (*J* = 6.1 Hz, H-6 of Rha′) and *δ* 1.15 (*J* = 6.0 Hz, H-6 of Rha) were assigned to the two secondary CH_3_ groups of Rha′ and Rha, respectively.^37^

The ^13^C NMR spectrum of 3 (Table 1) showed 37 signals: 24 from the tetrasaccharide and 13 from the kaempferol aglycone, including the characteristic equivalent carbon pairs C-2′/C-6′ (*δ* 130.60) and C-3′/C-5′ (*δ* 115.11), with four anomeric signals at *δ* 98.96 (Gal), 99.81 (Rha), 100.58 (Rha′), and 104.70 (Glc).^39,56^ The upfield secondary methyl resonances at *δ* 17.30 (C-6 of Rha′) and *δ* 17.81 (C-6 of Rha) confirmed the two rhamnoses. Overall interpretation of the ^13^C NMR data confirmed that all four sugar units exist in the pyranose configuration.

**Fig. 2:**
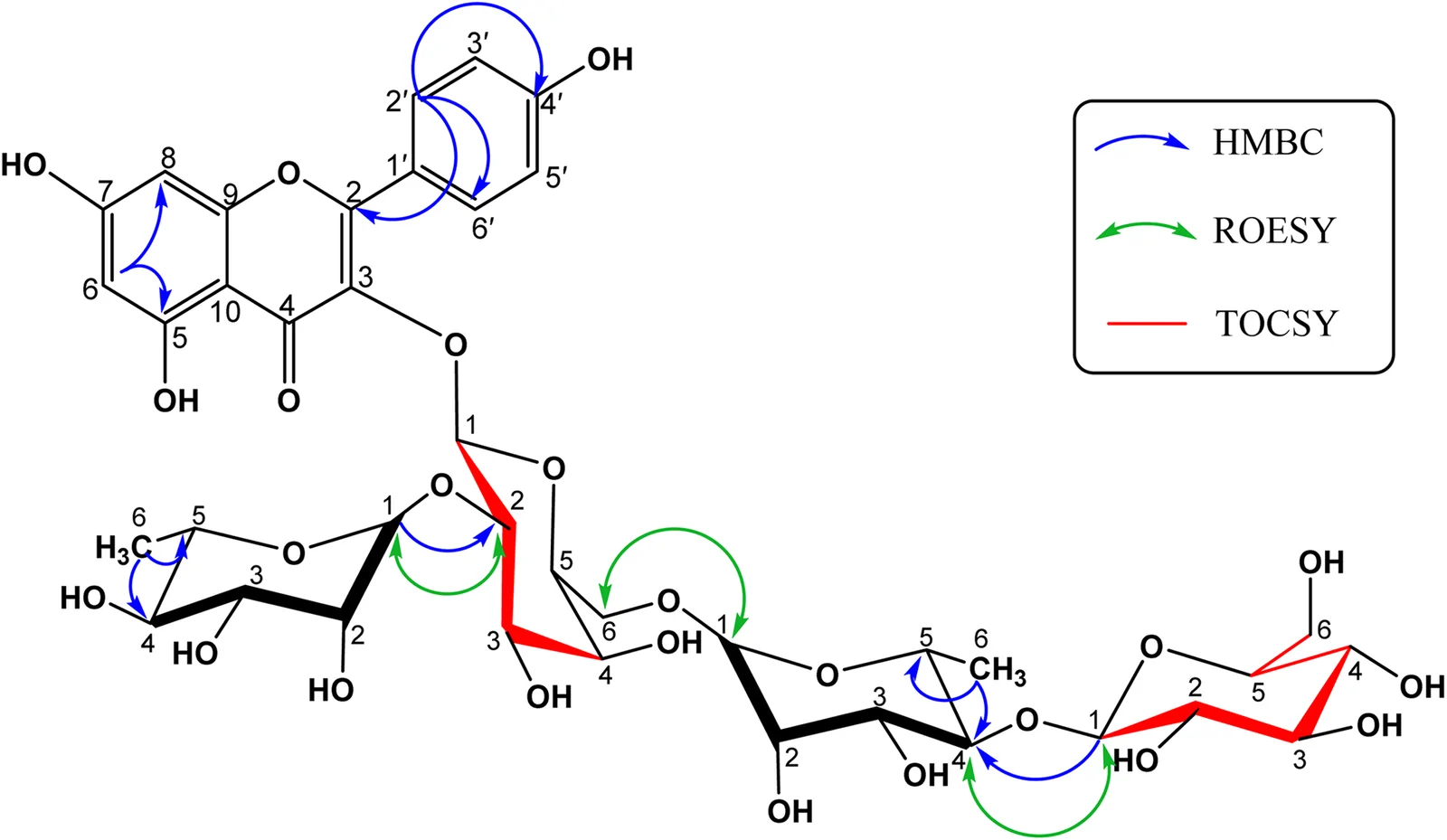
Key correlations of compound 3 provided by 2D NMR spectra: HMBC, ROESY and TOCSY.

Using COSY, ROESY, HSQC, TOCSY, HSQC-TOCSY, and HMBC experiments (Fig. 2), unambiguous assignment of the protons and carbons of each sugar in the tetra-saccharide chain as well as their sites of attachment were determined. The anomeric proton of each monosaccharide and their corresponding carbons were assigned using HSQC spectrum: *δ*_H_ 5.56 and *δ*_C_ 98.96 for Gal, *δ*_H_ 4.32 and *δ*_C_ 104.70 for Glc, *δ*_H_ 4.38 and *δ*_C_ 99.81 for Rha, and *δ*_H_ 5.04 and *δ*_C_ 100.58 for Rha′ [HSQC]. Starting from the downfield signals of the anomeric protons of each sugar as well as the upfield signals of the secondary CH_3_ protons of the two rhamnose units, unambiguous assignments of protons of each spin system and their corresponding carbons were determined using COSY, TOCSY, and HSQC-TOCSY experiments. The sequence and the site of attachment sugar chain were established based on the HMBC and ROESY correlations. The HMBC correlations between H-1 of Glu (*δ* 4.32) and C-4 of Rha (*δ* 82.36), as well as the ROESY correlations between H-1 of Glu (*δ* 4.32) and H-4 of Rha (*δ* 3.37) confirmed that Glu was attached to the C-4 position of the Rha. The attachment of Rha to C-6 of Gal was clearly established from the ROESY correlations between the rhamnosyl anomeric proton (*δ* 4.38) and H-6a (*δ* 3.59)/H-6b (*δ* 3.18) of Gal. The HMBC correlations between H-1 of Rha′ (*δ* 5.04) and C-2 of Gal (*δ* 75.04), along with the ROESY correlations between H-1 of Rha′ (*δ* 5.04) and H-2 (*δ* 3.78) of Gal established that the Rha′ was linked to the C-2 of Gal.

Furthermore, the downfield shifts of C-2 (*δ* 75.04) and C-6 (*δ* 64.77) in addition to the upfield shifts of C-l (*δ* 98.96) and C-5 (*δ* 73.05) of Gal indicated the rhamnosylation of Gal at C-2 and C-6 positions, respectively. Similarly, the downfield shifts of C-4 (*δ* 82.36) along with upfield shifts of C-3 (*δ* 70.57) and C-5 (*δ* 66.73) of Rha indicated the glucosylation of Rha at the C-4 position. The ^1^H and ^13^C NMR downfield shifts of CH_2_-6a of the Gal (*δ*_H_ 3.59) and (*δ*_C_ 64.77)] indicated the attachment of Rha at C-6 of the Gal moiety. The placement of the tetra-saccharide chain at C-3 of the aglycone was evidenced by the clearly observed deshielding of C-2 (*δ* 155.11) and shielding of C-3 (*δ* 132.26) of the aglycone relative to the corresponding carbons of kaempferol aglycone.^39,56^ Upon acid hydrolysis, compound 3 afforded d-galactose, d-glucose, and l-rhamnose as sugar moieties and kaempferol aglycone as identified by comparison with authentic samples. The β-anomeric configurations for the glucose and galactose residues were evident from the *J*_H1–H2_ values of 7.8 Hz and 7.7 Hz, respectively. The appearance of the anomeric signals of the rhamnose moieties as singlets indicated the α-configurations of the two rhamnose residues.^57^ Based on the foregoing evidence, compound 3 was identified as kaempferol-3-*O*-β-d-glucopyranosyl-(1→4)-α-l-rhamnopyranosyl-(1→6)-*O*-[α-l-rhamnopyranosyl-(1→2)]-β-d-galactopyranoside. To the best of our knowledge, this is the first report describing the isolation of this compound and was named “Guachapin A”.

Guachapin B (4) was isolated as a yellow amorphous powder. UV analysis of compound 4 indicated it was a flavonoid acylated with a hydroxycinnamic acid derivative. This was characterized by a broadened Band I, which was shifted to a shorter wavelength (*λ*_max_ 333 nm) relative to a non-acylated flavonol glycoside. The spectrum of hydroxycinnamic acid appeared superimposed on the flavonoid backbone. Notably, feruloylated derivatives consistently exhibited Band I at 330–334 nm; this serves as an additional spectroscopic feature that can aid in the direct annotation of such feruloylated glycosides in hyphenated analyses.^58,59^ The UV spectral data of 4 also indicated the flavonoid nature of 4 with free OH groups at C-5 (shift with A1C1_3_ and A1C1_3_/HC1), C-7 (shift with NaOAc), C-4′ (shift with NaOMe) and a substituted C-3 hydroxyl group.^55^ The ESI-MS displayed a pseudomolecular ion at *m*/*z* 1079 [M + H]^+^, indicating that 4 has 176 mass units more than compound 3, suggesting the presence of a feruloyl moiety in 4 relative to 3.^60^ Compound 4 was also presumed to be a flavonoid glycoside derivative, from the similarity of its spectral data (UV and NMR) and those of 3.

The ^1^H NMR spectrum of 4 (Table 1) displayed aglycone signals identical to those of 3, confirming the same kaempferol nucleus.^37^ The presence of four anomeric signals at *δ* 5.55 (*J* = 7.6 Hz, H-1 of Gal), *δ* 4.38 (s, H-1 of Rha), *δ* 4.36 (*J* = 8.8 Hz, H-1 of Glc) and *δ* 5.04 (s, H-1 of Rha′) along with the two doublets at *δ* 0.77 (*J* = 6.1 Hz, H-6 of Rha′) and *δ* 1.09 (*J* = 6.0 Hz, H-6 of Rha) is in close agreement with the tetrasaccharide moiety of 3.

**Fig. 3:**
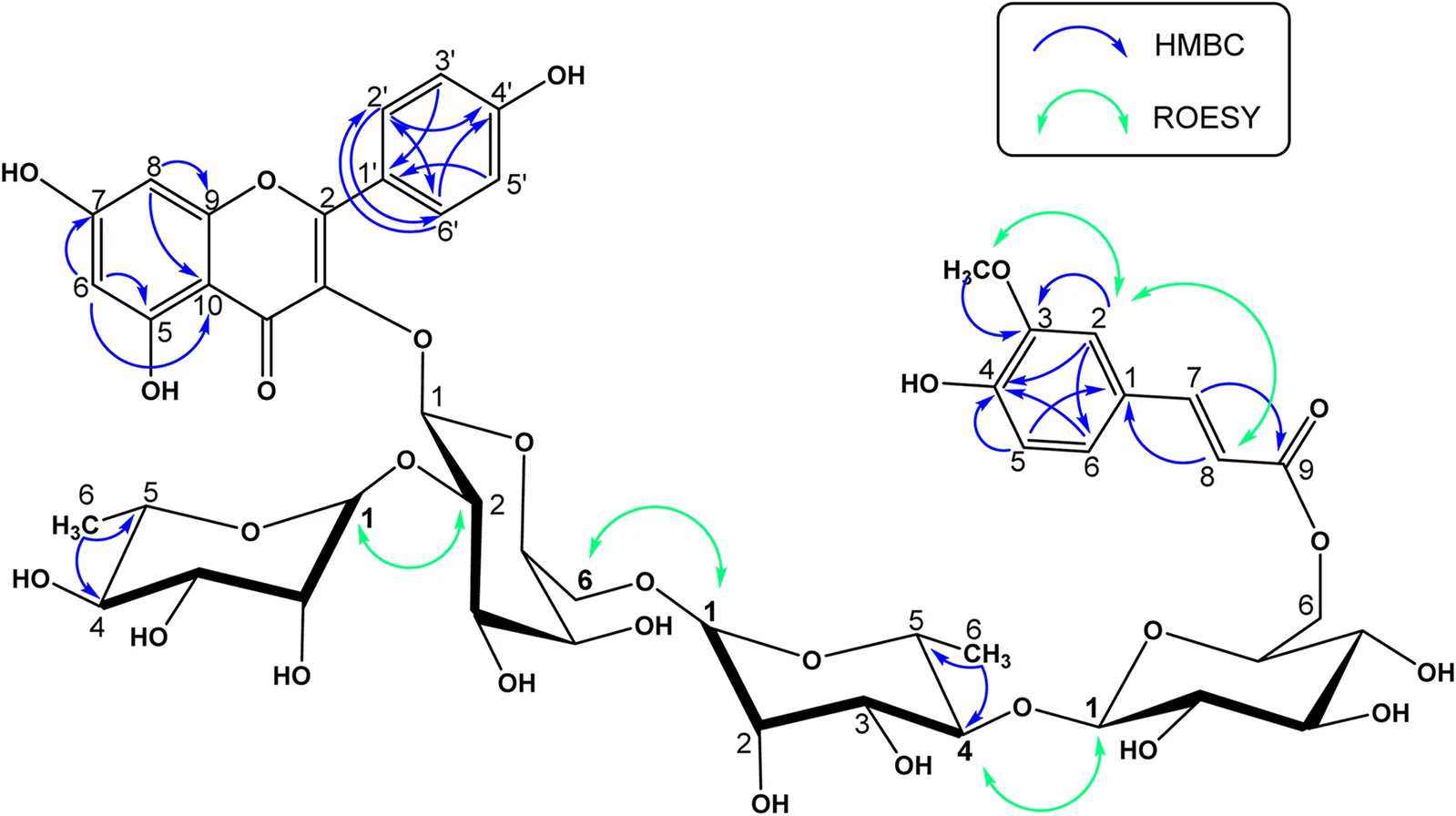
Key correlations of compound 4 provided by 2D NMR spectra: HMBC and ROESY.

The concerted use of ^1^H NMR and 2D (HSQC, HMBC and ROESY) data in 4 as well as ^13^C NMR data obtained from HMBC and HSQC spectra (Table 1 and Fig. 3) showed the existence of one additional feruloyl moiety attached to position C-6 of Glu. The extra resonances for the feruloyl unit indicated by the signals of two *trans*-olefinic protons at *δ* 7.52 (1H, d, *J* = 16.0 Hz) and *δ* 6.44 (1H, d, *J* = 16.0 Hz) attributed to H-7 and H-8, respectively, an ABX system at *δ* 7.29 (1H, d, *J* = 1.6. Hz), *δ* 6.78 (1H, d, *J* = 8.1 Hz) and *δ* 7.09 (1H, dd, *J* = 8.1, 1.7 Hz) attributed to H-2, H-5 and H-6 of the tri-substituted phenyl ring, respectively, and one OCH_3_ signal at *δ* 3.80 (3H, s).

Similarly, the site of attachment and the sequence of the sugar moieties in 4 were confirmed to be the same as in 3 from the ROESY correlations (Fig. 3). The linkage of Glu to C-4 of Rha was evidenced from the ROESY correlations between H-1 of Glu (*δ* 4.36) and H-4 of Rha (*δ* 3.33). The observed ROESY correlations between the rhamnosyl anomeric proton (*δ* 4.38) and H-6a (*δ* 3.57)/H-6b (*δ* 3.18) of Gal confirmed the attachment of Rha to C-6 of Gal. Furthermore, the ROESY spectrum showed a correlation between H-1 of Rha′ (*δ* 5.04) and H-2 of Gal (*δ* 3.78), indicating the attachment of Rha′ to the C-2 position of Gal. The assignment of the site of linkage of the feruloyl unit to the C-6 position of Glu was based on the downfield shift of H_2_-6 (*δ* 4.21 and 4.01) and C-6 (*δ* 64.11) of the Glc unit. Acid hydrolysis of 4 afforded d-glucose, d-galactose, and l-rhamnose as sugar moieties, in addition to kaempferol and ferulic acid, which were confirmed by comparison with authentic samples. Based on the foregoing data, compound 4 was established to contain an additional feruloyl moiety *versus* compound 3. Therefore, compound 4 was deduced to be kaempferol-3-*O*-β-d-glucopyranosyl-[6-*O-E*-feruloyl]-(1→4)-α-l-rhamnopyranosyl-(1→6)-*O*-[α-l- rhamnopyranosyl-(1→2)]-β-d-galactopyranoside. This is the first report of the isolation of this compound and was named Guachapin B. This study constitutes the first phytochemical study of *A*. guachapele and describes the isolation and complete structural characterization of two previously unreported compounds (3 and 4).

### 3.2. *In silico* studies

#### 3.2.1. Molecular docking

**Fig. 4:**
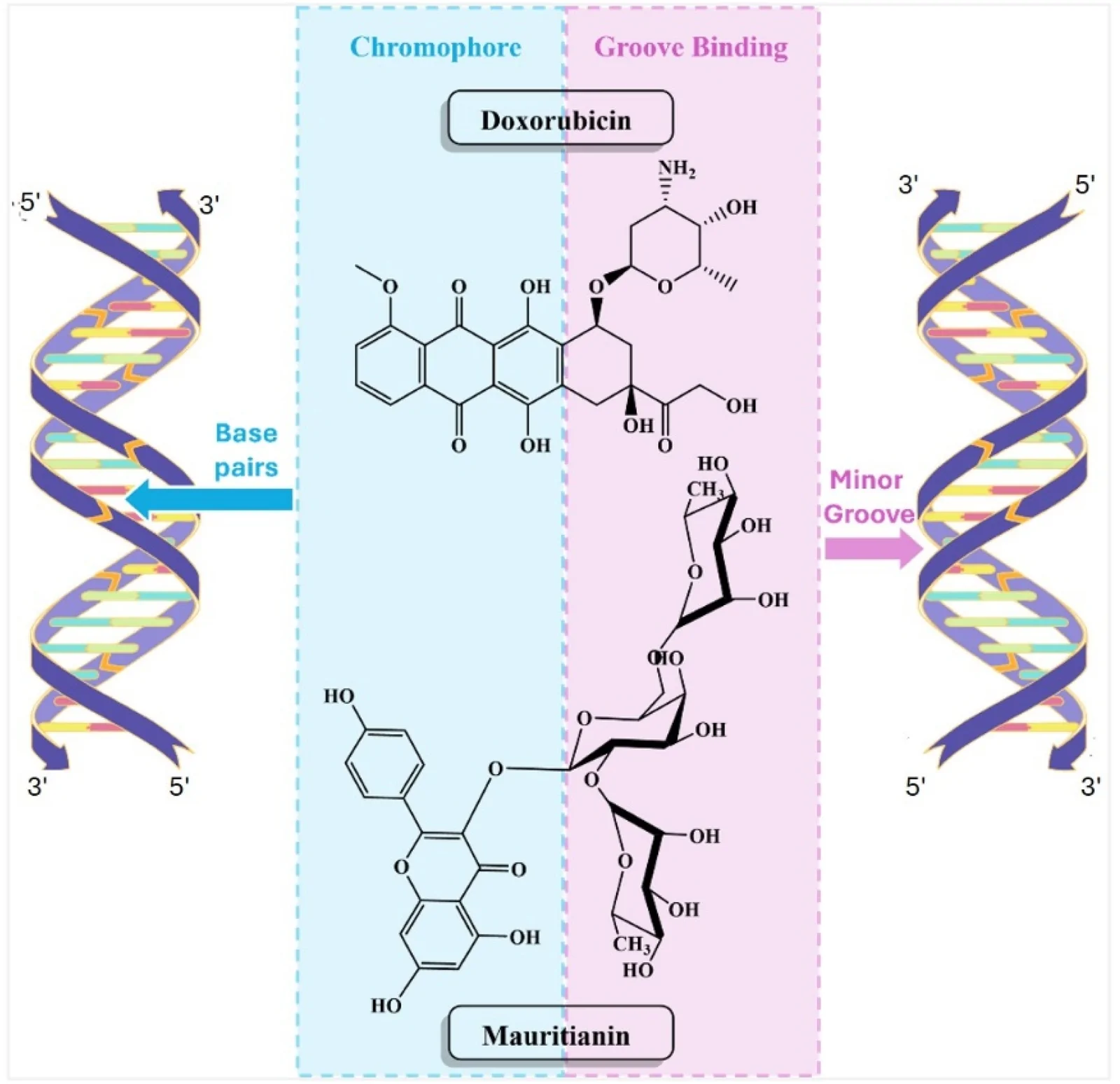
Structural comparison of pharmacophoric features shared between doxorubicin and compound 1 (mauritianin), highlighting the planar aromatic system and hydrogen-bond donor/acceptor groups relevant to DNA–Topo II interaction.

Topo II poisons and DNA intercalators possess two key pharmacophoric features: a planar polyaromatic system (chromophore) that enables intercalation between DNA base pairs, and a side chain or functional group capable of interacting with the DNA minor groove and orienting toward the Topo II enzyme. Application of these criteria to compound 1 revealed structural features (a planar polyaromatic chromophore and multiple hydroxyl groups) that are shared with known Topo II-active compounds including doxorubicin (Fig. 4). These features informed the selection of the DNA–Topo II complex as the docking target and provide a structural rationale for exploring a Topo II-related mechanism, though they do not constitute experimental evidence of DNA intercalation. It should be noted that this pharmacophoric analysis identifies Topo II as a plausible and prioritized computational target for compound 1 and does not imply exclusivity of interaction. Flavonoid glycosides are known to engage multiple protein targets, and the structural features identified here may simultaneously support interactions with kinases, transcription factors, or other enzymes. The docking study is therefore interpreted as a target-focused hypothesis rather than a comprehensive mechanistic characterization.

A molecular docking study was conducted for compound 1, with doxorubicin serving as the reference compound. The aim was to predict the preferred binding geometry of compound 1 within the DNA–Topo II active site and to generate a computational rationale for its biological evaluation. The calculated binding free energies (Δ*G*) are presented in Table 2. Spontaneous and thermodynamically favorable binding is indicated by the negative Δ*G* values.

**Table 2:** The calculated Δ*G* (kcal mol^−1^) and interactions of compound 1 in comparison to doxorubicin at the DNA–Topo II active site

Compounds	Δ*G*	Type of interaction	Interacting residues
Compound 1	−33.48	Hydrogen bond	Cyt14, Gua13, Asp479, Lys456, and Arg503
		Hydrophobic interaction	Ade12 and Gua13
Doxorubicin	−24.79	Hydrogen bond	Gua10, Thy9, Gua13, and Arg503
		Hydrophobic interaction	Thy9 and Ade12

**Fig. 5:**
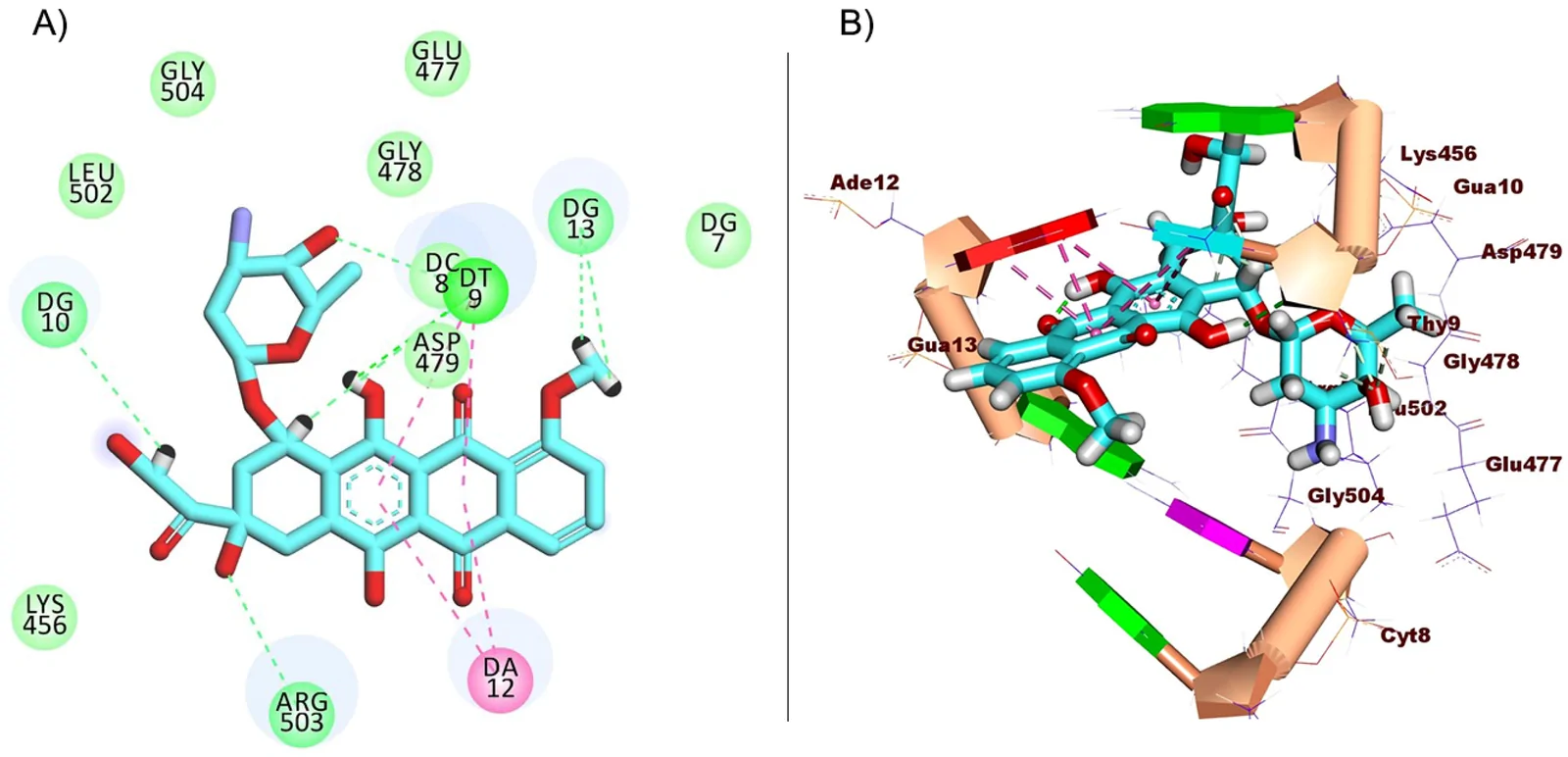
Doxorubicin binding to the DNA–Topo II complex; 2D (A) and 3D (B). Pink = π-interactions; green = hydrogen bonds.

**Fig. 6:**
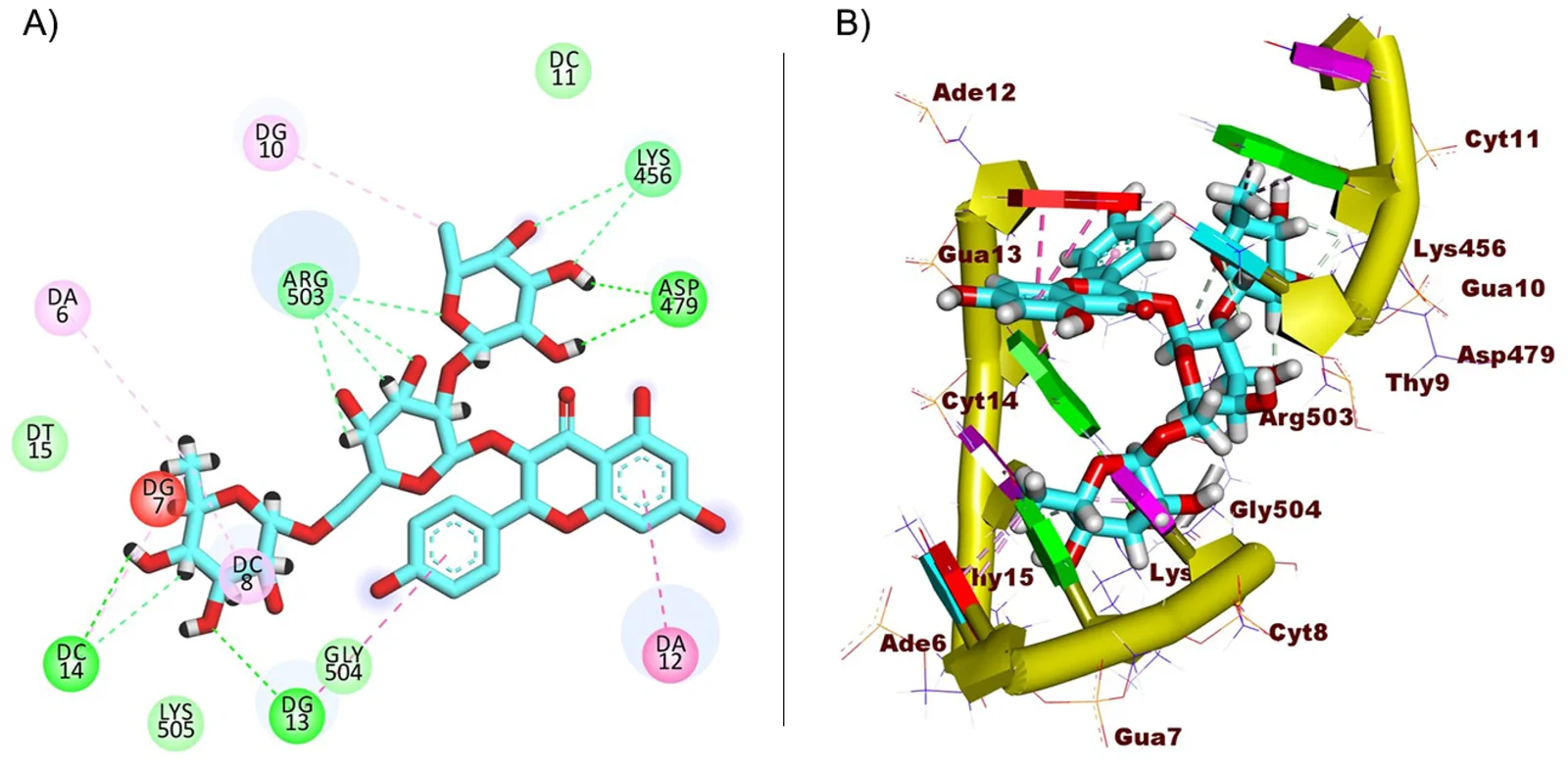
Compound 1 binding to the DNA–Topo II complex; 2D (A) and 3D (B). Pink = π-interactions; green = hydrogen bonds.

The predicted binding mode of doxorubicin exhibited a binding affinity of −24.79 kcal mol^−1^. Its planar aromatic scaffold engaged in four π–π stacking interactions with key nucleobases Thy9 and Ade12, in addition to forming seven hydrogen bonds with Gua10, Thy9, Gua13, and Arg503 (Fig. 5). Compound 1 (mauritianin) demonstrated a markedly stronger predicted affinity of −33.48 kcal mol^−1^. Its planar aromatic nucleus established π-stacking interactions with Ade12 and Gua13. The molecule also formed eleven hydrogen bonds involving Cyt14, Gua13, Asp479, Lys456, and Arg503, complemented by five hydrophobic contacts (Fig. 6). This interaction profile highlights the strong predicted binding affinity of compound 1 for the DNA–Topo II complex and provides a computational rationale for its advancement to MD simulations. It should be noted that docking scores reflect predicted binding free energies within the modelled system and do not directly distinguish between intercalation, groove binding, or allosteric modes of interaction, nor between catalytic inhibition and cleavable-complex stabilization.

#### 3.2.2. Molecular dynamics (MD) simulation

**Fig. 7:**
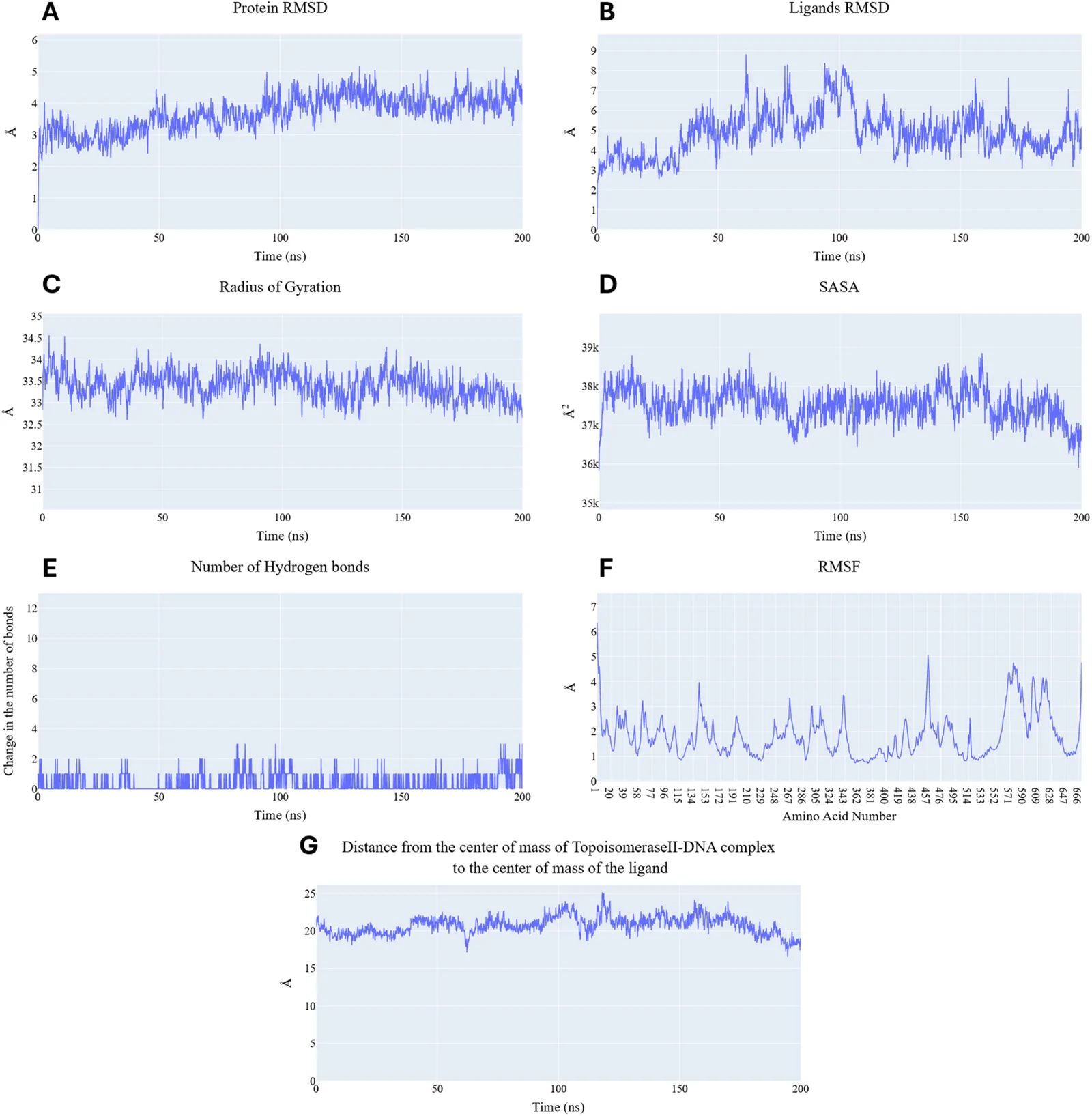
Molecular dynamics simulation analysis of the mauritianin–Topo II/DNA complex over 200 ns: (A) protein RMSD, (B) ligand RMSD, (C) radius of gyration, (D) SASA, (E) hydrogen bonds, (F) RMSF, (G) center-of-mass distance.

A 200 ns MD simulation was conducted to assess the conformational stability of the predicted compound 1–Topo II–DNA complex.^61^ Backbone RMSD stabilized at ∼3 Å, increasing modestly to ∼4 Å after 100 ns before plateauing (Fig. 7A). Ligand RMSD fluctuated initially, peaking at ∼8 Å at 30 ns, then converging to ∼5 Å for the remainder of the trajectory (Fig. 7B), indicating stable binding pose convergence. RoG and SASA values remained largely constant throughout (Fig. 7C and D), confirming overall protein structural integrity. Hydrogen bond analysis revealed persistent formation of approximately one hydrogen bond, with occasional instances of two (Fig. 7E). RMSF profiles showed generally low C-α fluctuations (∼2.5 Å); elevated mobility was noted at LYS965 (5 Å), ASN1083 (4.7 Å), GLU1111 (4 Å), and LEU1146 (4 Å)—residues distal from the binding site (Fig. 7F). Centroid distance analysis confirmed consistent ligand positioning at an average distance of ∼20 Å throughout the simulation (Fig. 7G). Collectively, these metrics support stability of the predicted complex.

**Fig. 8:**
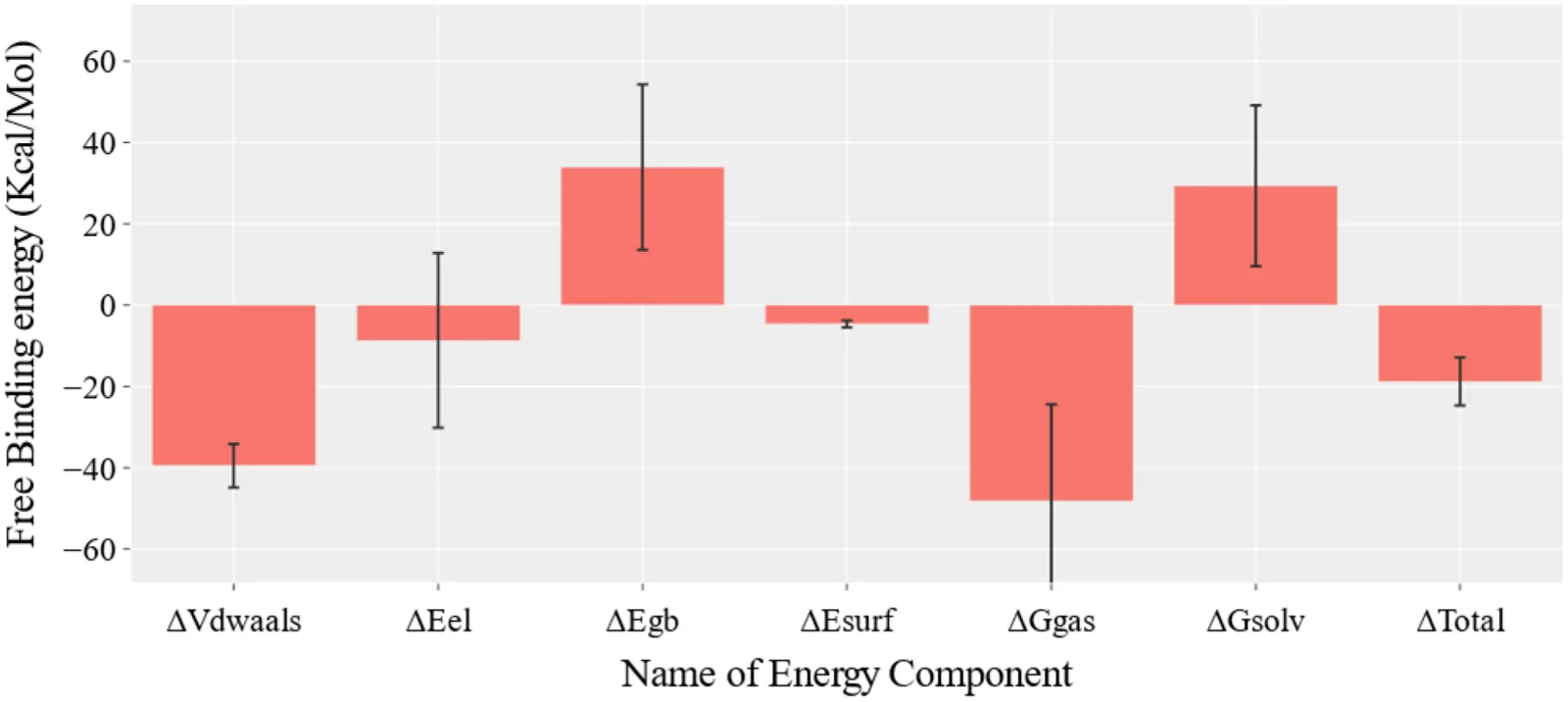
Individual MM-GBSA energy components and their corresponding values for the system; error bars indicate standard deviations.

**Fig. 9:**
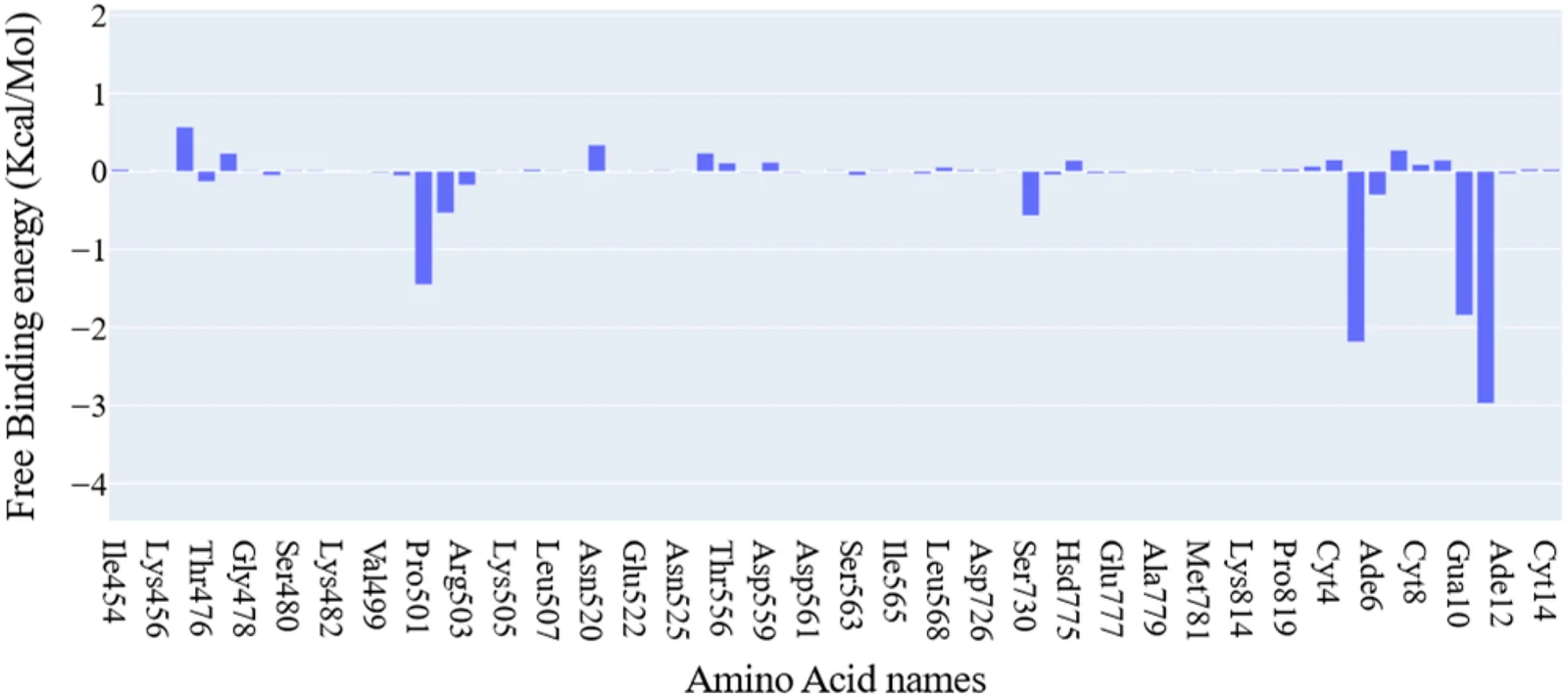
Decomposition of binding free energy for the mauritianin–Topo II–DNA complex, showing residues within 1 nm of the ligand.

MM-GBSA analysis yielded an overall binding free energy of −18.76 kcal mol^−1^, dominated by van der Waals interactions (−39.5 kcal mol^−1^) with additional electrostatic contributions (−8.65 kcal mol^−1^) (Fig. 8). Per-residue decomposition identified Cytosine11 (−2.9 kcal mol^−1^), Guanine5 (−2.19 kcal mol^−1^), Guanine10 (−1.9 kcal mol^−1^), and PRO501 (−1.45 kcal mol^−1^) as the principal stabilizing contributors, with minor destabilizing contributions from LEU457 (+0.6 kcal mol^−1^) and ASN520 (+0.34 kcal mol^−1^) (Fig. 9).

**Fig. 10:**
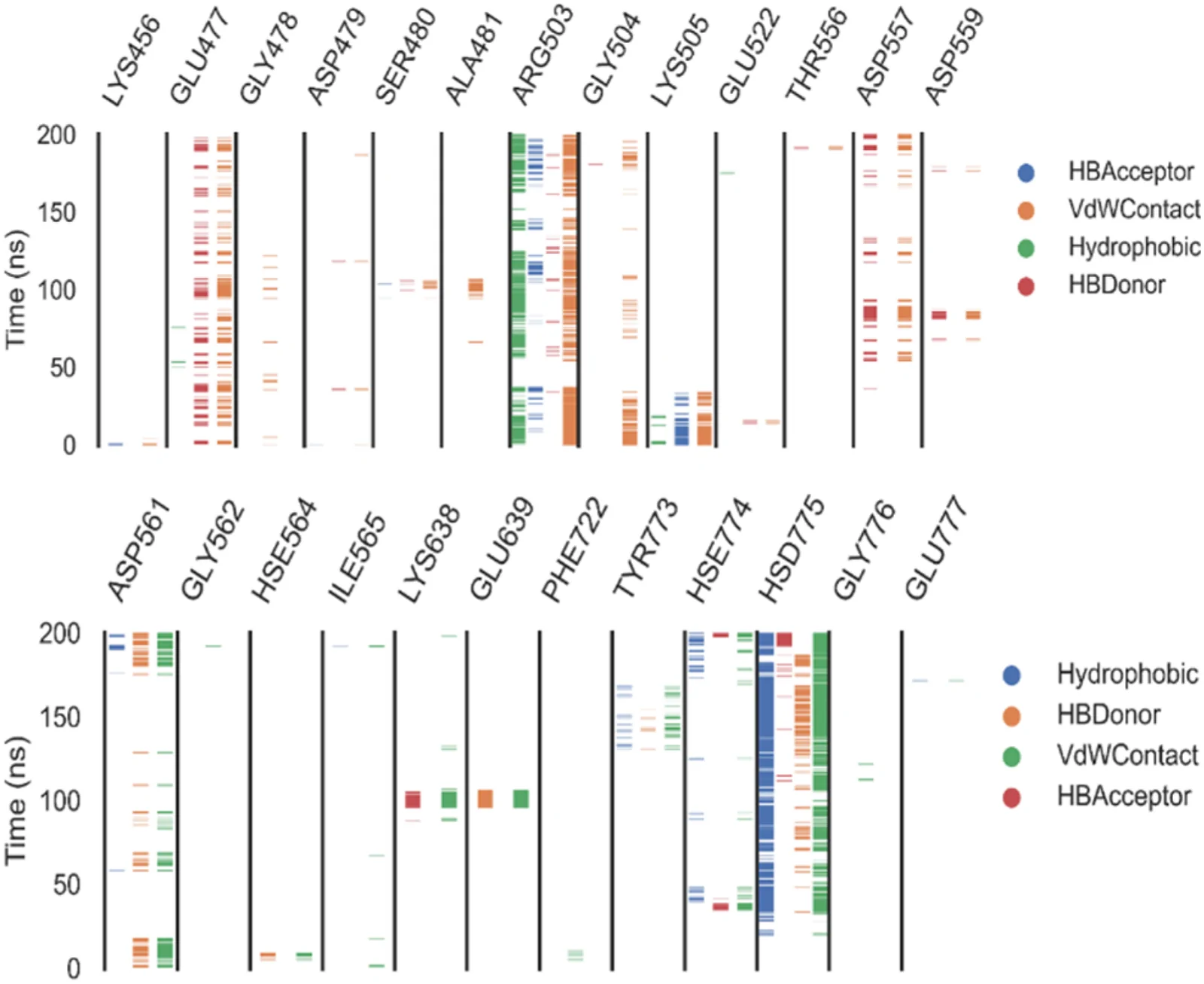
Amino acids interaction profiles within the Topo II–DNA complex, with their interaction types and occurrence throughout the whole simulation.

ProLIF fingerprint analysis identified HSD775 as the most consistently interacting residue, maintaining hydrophobic contact in 54% of simulation frames (Fig. 10).

**Fig. 11:**
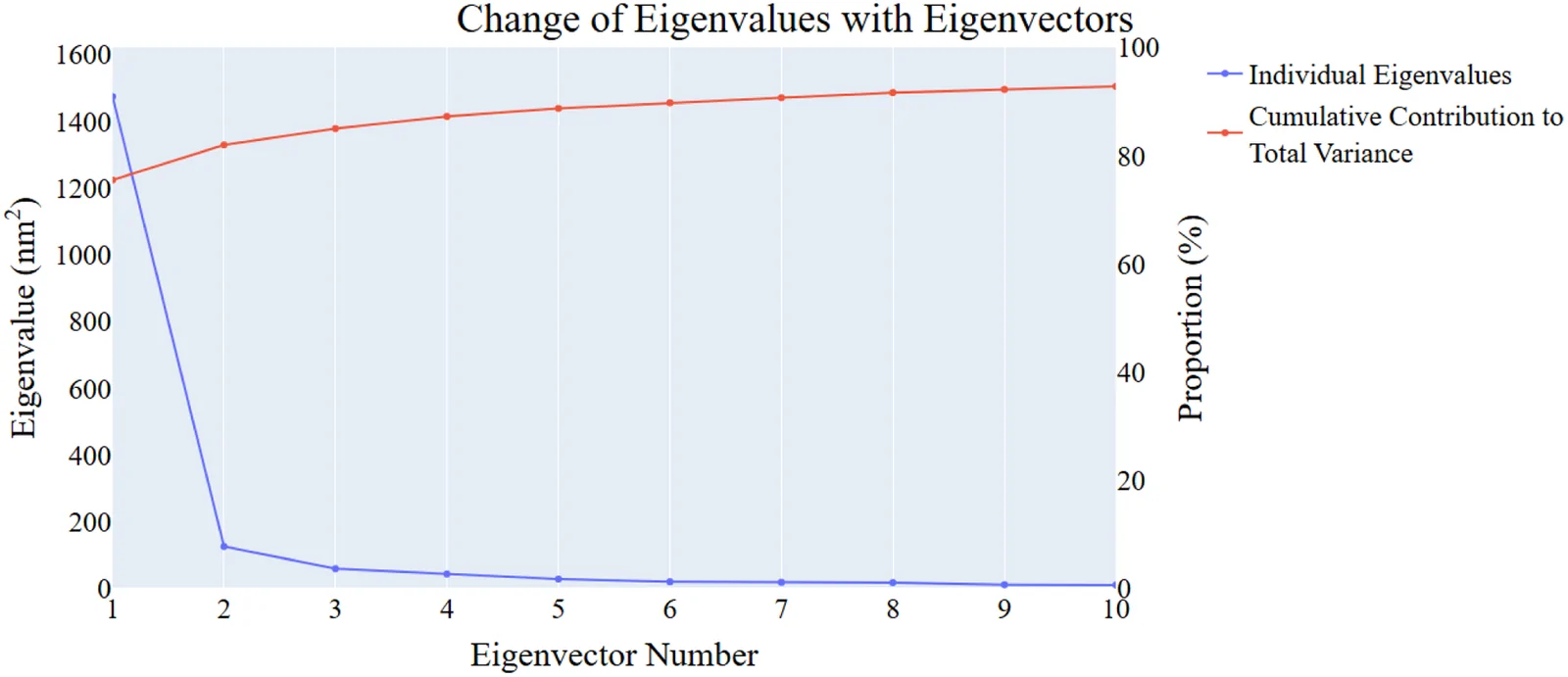
Scree plot showing eigenvalue decay with increasing eigenvector number (blue line) and the cumulative variance explained (red line).

**Fig. 12:**
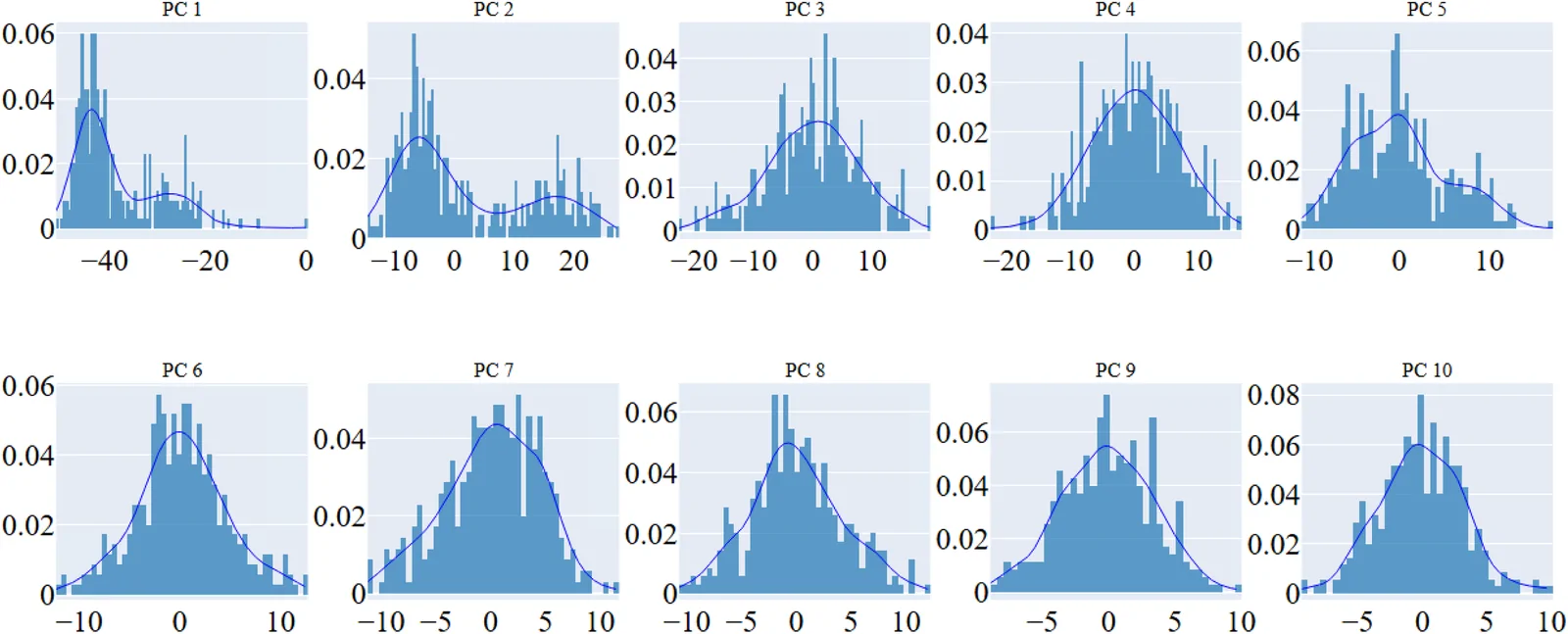
Histograms showing the distributions of the first ten PCs.

**Fig. 13:**
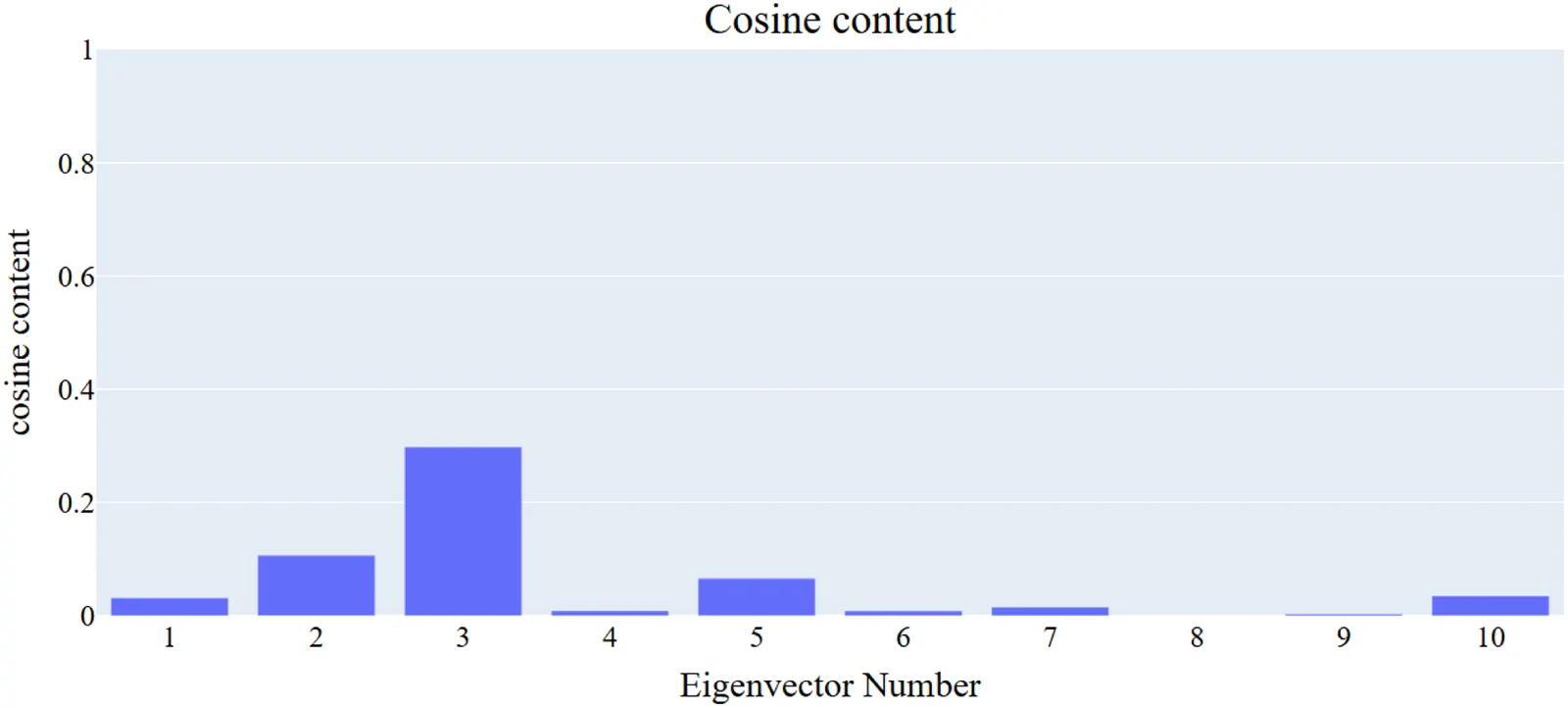
Cosine content values of the first ten eigenvectors for the two trajectories.

PCA of C-α coordinates revealed that PC1 alone captured 75.5% of total variance, with the first three PCs collectively accounting for 84.9% (Fig. 11 and 12). Cosine content values for the first ten PCs were below 0.1—except PC2 (0.11) and PC3 (0.29)—confirming that the dominant motions represent meaningful, non-stochastic dynamics (Fig. 13).

**Fig. 14:**
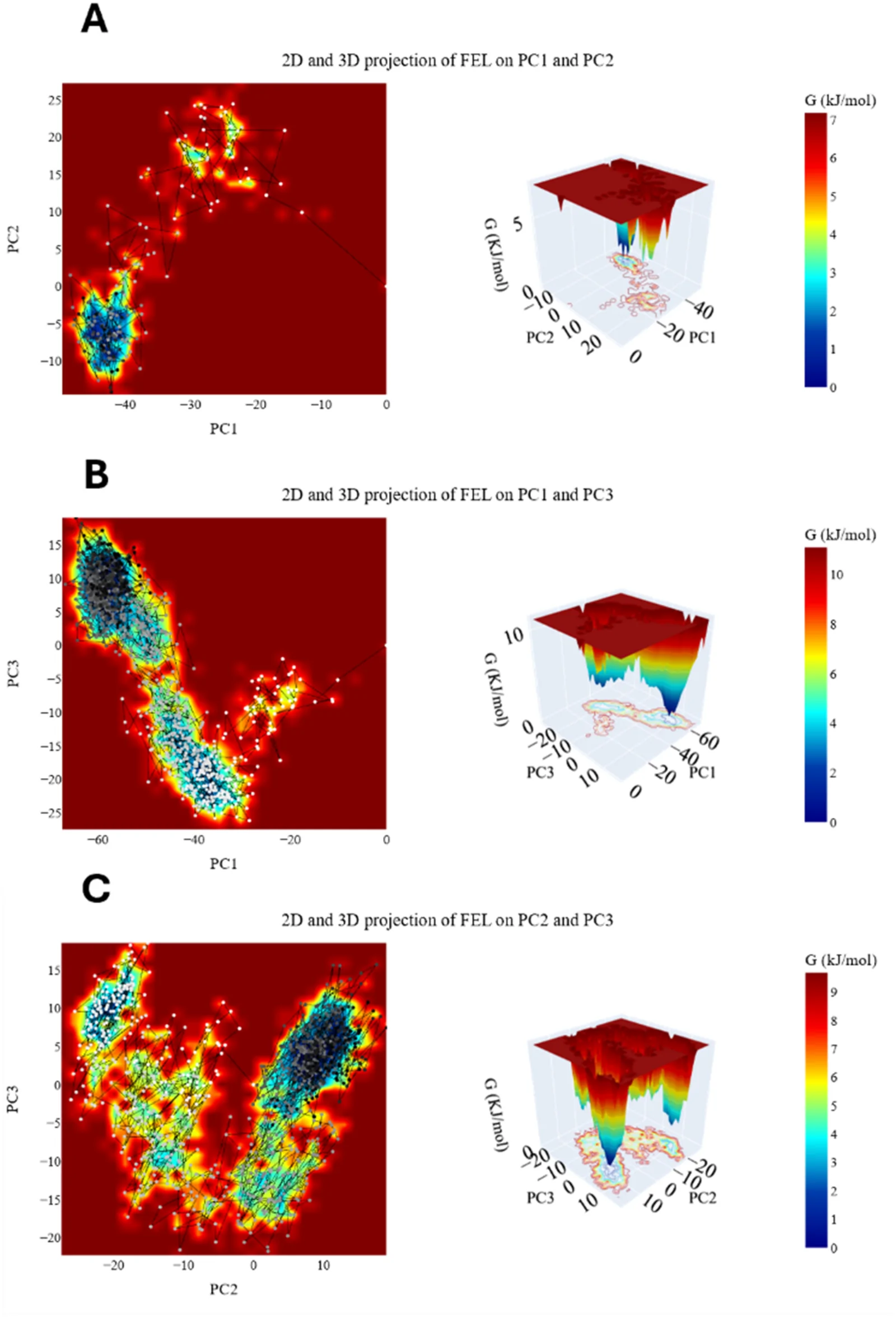
Combined 2D and 3D FEL projections of the mauritianin–Topo II–DNA complex along PCs 1–2 (A), 1–3 (B), and 2–3 (C). White-to-black dots show trajectory frames over time, and the color scale represents free energy calculated using gmx sham.

FEL projections onto PC1–PC2, PC1–PC3, and PC2–PC3 subspaces revealed convergence to well-defined global energy minima in all three projections (Fig. 14A–C), with minimal energy differences between adjacent basins (0.0–0.281 kJ mol^−1^), consistent with a conformationally stable complex throughout the simulation.

### 3.3. *In vitro* studies

#### 3.3.1. Topoisomerase II inhibitory activity

The effect of compound 1 on intracellular Topo II alpha protein levels was evaluated using a sandwich ELISA-based immunoassay. Treatment with compound 1 produced a concentration-dependent reduction in detectable Topo II alpha protein, with an EC_50_ of 15.12 ng mL^−1^ (20.43 nM), compared to 30.16 nM for doxorubicin under identical conditions. This indicates that compound 1 reduces Topo II alpha protein levels more potently than doxorubicin at equivalent concentrations in this assay format.

It must be noted that a reduced sandwich ELISA signal in treated lysates can arise from at least three mechanistically distinct processes (reduced protein expression, enhanced proteasomal degradation of Topo II alpha, or steric epitope masking) none of which constitute direct enzymatic inhibition or cleavable-complex stabilization. The observed concentration-dependent reduction in detectable Topo II alpha protein is therefore interpreted as an indirect indicator of a Topo II-related cellular effect, corroborative of the strong binding affinity predicted by molecular docking and MD simulations, rather than as a functional inhibition constant. Confirmation by plasmid DNA relaxation or decatenation assays remains necessary to characterize the precise mode of Topo II interaction.

#### 3.3.2. Cytotoxic activity

**Fig. 15:**
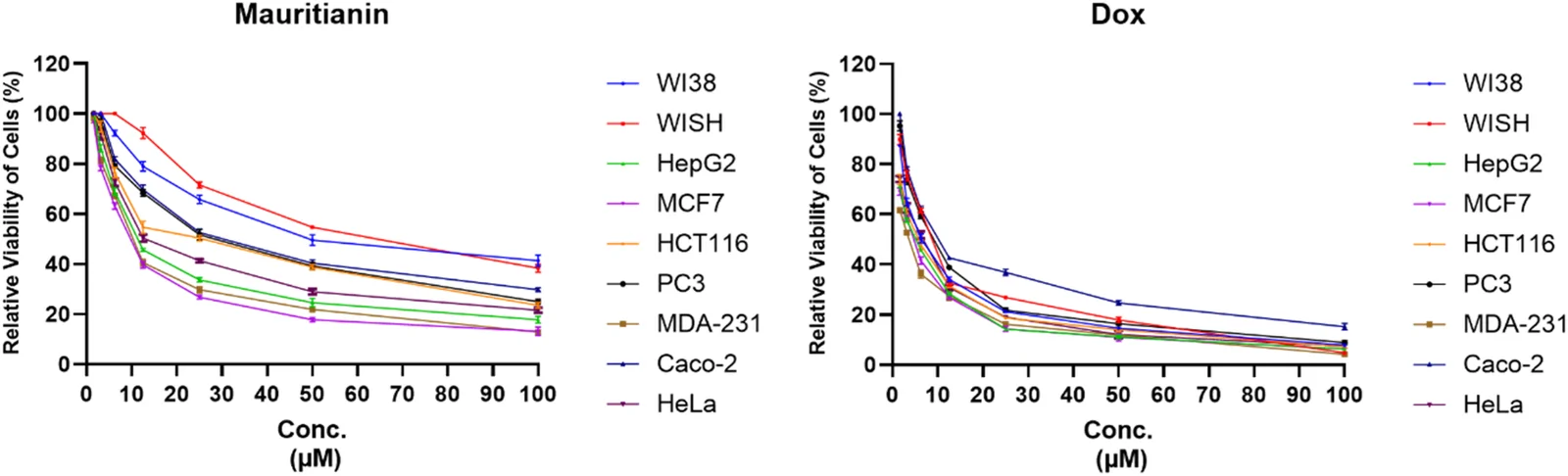
Anticancer activity of compound 1 compared with doxorubicin at different concentrations.

The cytotoxic activity of compound 1 (mauritianin) was evaluated by MTT assay against a panel of nine human cell lines: two normal lines—lung fibroblast (WI-38) and amnion (WISH)—and seven cancer lines representing breast (MCF-7, MDA-MB-231), liver (HePG-2), cervical (HeLa), prostate (PC-3), and colorectal (HCT-116, Caco-2) cancers.^31^ The values of the average relative viability (%) of cell lines treated with compound 1 or doxorubicin (reference compound) across concentrations (1.56–100 µM) are shown in Fig. 15. Exact IC_50_ values and selectivity indices for both compound 1 and doxorubicin are presented in Table 3.

**Table 3:** IC_50_ values (µM) of compound 1 and doxorubicin against human cell lines, with selectivity indices (SI) of compound 1^a^

Cell line	IC_50_ cmpd 1 (µM)	IC_50_ DOX (µM)	SI of cmpd 1 (WI-38)	SI of cmpd 1 (WISH)
MCF-7	10.28	4.17	5.47	6.02
MDA-MB-231	11.66	3.18	4.82	5.31
HePG-2	14.03	4.50	4.01	4.41
HeLa	18.13	5.57	3.10	3.41
HCT-116	24.88	5.23	2.26	2.49
PC-3	29.76	8.87	1.89	2.08
Caco-2	33.04	12.49	1.70	1.87
WI-38	56.20	6.72	NA	NA
WISH	61.90	8.72	NA	NA

a SI = IC_50_ (normal cell line)/IC_50_ (cancer cell line); SI ≥ 2 indicates selective cytotoxicity. IC_50_ values expressed as mean ± SD (*n* = 3 independent experiments, each in triplicate). DOX = doxorubicin (positive control). Cytotoxicity classification: 1–10 µM (very strong), 11–20 µM (strong), 21–50 µM (moderate), 51–100 µM (weak), >100 µM (non-cytotoxic). NA: not applicable (normal cell lines used as SI reference). Compound 1 exhibited strong cytotoxic activity against MCF-7 (IC_50_ = 10.28 µM), MDA-MB-231 (11.66 µM), HePG-2 (14.03 µM), and HeLa (18.13 µM) cell lines, moderate activity against HCT-116 (24.88 µM), PC-3 (29.76 µM), and Caco-2 (33.04 µM), and weak activity against normal WI-38 (56.20 µM) and WISH (61.90 µM) cell lines. Doxorubicin demonstrated superior absolute potency across all lines (IC_50_ = 3.18–12.49 µM), as expected for a clinically optimized cytotoxic agent.

Critical comparison of selectivity indices, however, reveals a substantially more favorable therapeutic window for compound 1. Doxorubicin SI values against WI-38 ranged from 0.54 (Caco-2) to 2.11 (MDA-MB-231), indicating comparable or greater toxicity toward normal cells relative to several cancer lines. By contrast, compound 1 achieved SI values of 1.70–5.47 (*vs.* WI-38) and 1.87–6.02 (*vs.* WISH), with the strongest selectivity observed for MCF-7 (SI = 5.47; 6.02) and MDA-MB-231 (SI = 4.82; 5.31). These results identify breast cancer cell lines as the most selectively affected targets and demonstrate that compound 1, despite its lower absolute potency relative to doxorubicin, possesses a meaningfully superior cancer-cell selectivity profile.^62,63^ Compound 1 is therefore characterized as a phytochemical lead scaffold warranting structural optimization to improve potency while retaining the observed selectivity advantage.

To contextualize the antiproliferative activity of compound 1 within the broader flavonol glycoside literature, its IC_50_ values and Topo II-related profile are compared with previously reported analogues in Table 4. Among previously reported flavonol glycosides with Topo II-related antiproliferative activity, isoquercitrin (quercetin-3-*O*-glucoside) represents the most directly comparable monoglycoside, having been shown to directly inhibit Topo II in a DNA relaxation assay alongside S-phase arrest and caspase-3-mediated apoptosis in HepG2 cells.^64^ Rutin (quercetin-3-*O*-rutinoside), a structurally related diglycoside, induced G2/M arrest at 20 µM and G0/G1 arrest at 50 µM in MCF-7 and MDA-MB-231 cells, alongside apoptosis induction and ABC transporter inhibition, though without a direct Topo II enzymatic assay.^65^ Kaempferol-7-*O*-β-D-glucoside, a kaempferol monoglycoside isomer, induced G2/M arrest *via* Cyclin B1/Cdk1 downregulation in HeLa cells in a p53-independent manner, with mitochondrial apoptosis and NF-κB inhibition, representing the closest aglycone-matched comparator to compound 1.^66^ Particularly noteworthy in the context of the present study are flavonol glycosides from Leguminosae: Tselepi *et al.* reported that an acylated kaempferol glycoside isolated from *Vicia faba* and *Lotus edulis* acted as the strongest catalytic inhibitor among nine polyphenols tested against human Topo II (IC_50_: 240–600 µM), also inhibiting the growth of MCF-7, HeLa, and HepG2 cells^67^—a finding directly parallel to the present results from *A. guachapele*, also a Leguminosae member. Collectively, compound 1 (mauritianin) compares favourably with these reported flavonol glycosides in antiproliferative potency against MCF-7 cells (IC_50_ = 10.28 µM) and selectivity index (SI = 5.47–6.02), while its Topo II alpha protein-level reduction profile represents a distinct assay type from the catalytic assays used for the comparators above. Direct enzymatic comparison awaits functional Topo II assay data. For a comprehensive review of flavonoid topoisomerase inhibition mechanisms and structure–activity relationships, see Sitarek *et al.*^7^

**Table 4:** Comparison of Topo II-related antiproliferative activity of selected flavonol glycosides

Compound	Aglycone	Cell line	IC_50_ (µM)	Topo II assay	Reference
Mauritianin (1)	Kaempferol	MCF-7	10.28	Topo II alpha protein reduction; EC_50_ = 20.43 nM (sandwich ELISA)	This study
Isoquercetin (quercetin-3-*O*-glucoside)	Quercetin	HepG-2	NR^a^	Direct topo II inhibition (DNA relaxation assay); S-phase arrest; caspase-3 apoptosis	64
Rutin (quercetin-3-*O*-rutinoside)	Quercetin	MCF-7; MDA-MB-231	20–50 µM (effect conc.)	G2/M (20 µM) and G0/G1 (50 µM) arrest; apoptosis; ABC transporter inhibition; chemosensitization	65
Kaempferol-7-*O*-β-D-glucoside	Kaempferol	HeLa	NR^a^	G2/M arrest (cyclin B1/Cdk1 decrease, p53-independent); Bax↑, Bcl-2↓; mitochondrial apoptosis; NF-κB inhibition	66
Acylated kaempferol glycoside	Kaempferol	MCF-7; HeLa; HepG2	NR^a^	Catalytic topo I + II inhibition (human topo II IC_50_: 240–600 µM); growth inhibition of MCF-7, HeLa, HepG2	67
Quercetin/luteolin	Quercetin; luteolin	CHO AA8	NR^a^	Catalytic topo II inhibition; high-yield endoreduplication; G2/M-related DNA damage	68

a NR = not reported as a discrete IC_50_ value in the cited study; effect concentrations or qualitative inhibition reported instead.

It should be noted that the observed activity may not originate exclusively from the intact glycoside; intracellular glycosidase-mediated deglycosylation could generate kaempferol or partially deglycosylated intermediates as the pharmacologically active species. Definitive attribution requires LC-MS metabolite profiling of treated cell lysates or direct comparison with the kaempferol aglycone, and is identified as a future experimental priority.

#### 3.3.3. Flow cytometric analysis of the cell cycle and apoptosis

Having established a coherent *in silico* profile (comprising a strong predicted binding affinity at the DNA–Topo II active site, stable complex geometry across 200 ns of MD simulation, and a favorable binding free energy decomposition) compound 1 was advanced to cell-based biological evaluation. The *in vitro* studies described below were conducted independently to characterize the antiproliferative, Topo II alpha protein-level, and mechanistic cellular effects of compound 1. These experimental observations are presented as consistent with, and supportive of, the computational hypothesis, rather than as its direct confirmation. The relationship between *in silico* binding predictions and the observed cellular phenotype requires further mechanistic investigation using functional enzymatic assays.

MCF-7 cells were selected for mechanistic follow-up based on three criteria: the lowest IC_50_ among all tested cancer lines (10.28 µM), the highest selectivity indices against normal cells (SI = 5.47 *vs.* WI-38; 6.02 *vs.* WISH), and their well-established Topo II expression and doxorubicin-responsive phenotype, which makes them the standard model for Topo II-related mechanistic investigations in breast cancer research.

##### 3.3.3.1. Cell‐cycle distribution analysis

**Fig. 16:**
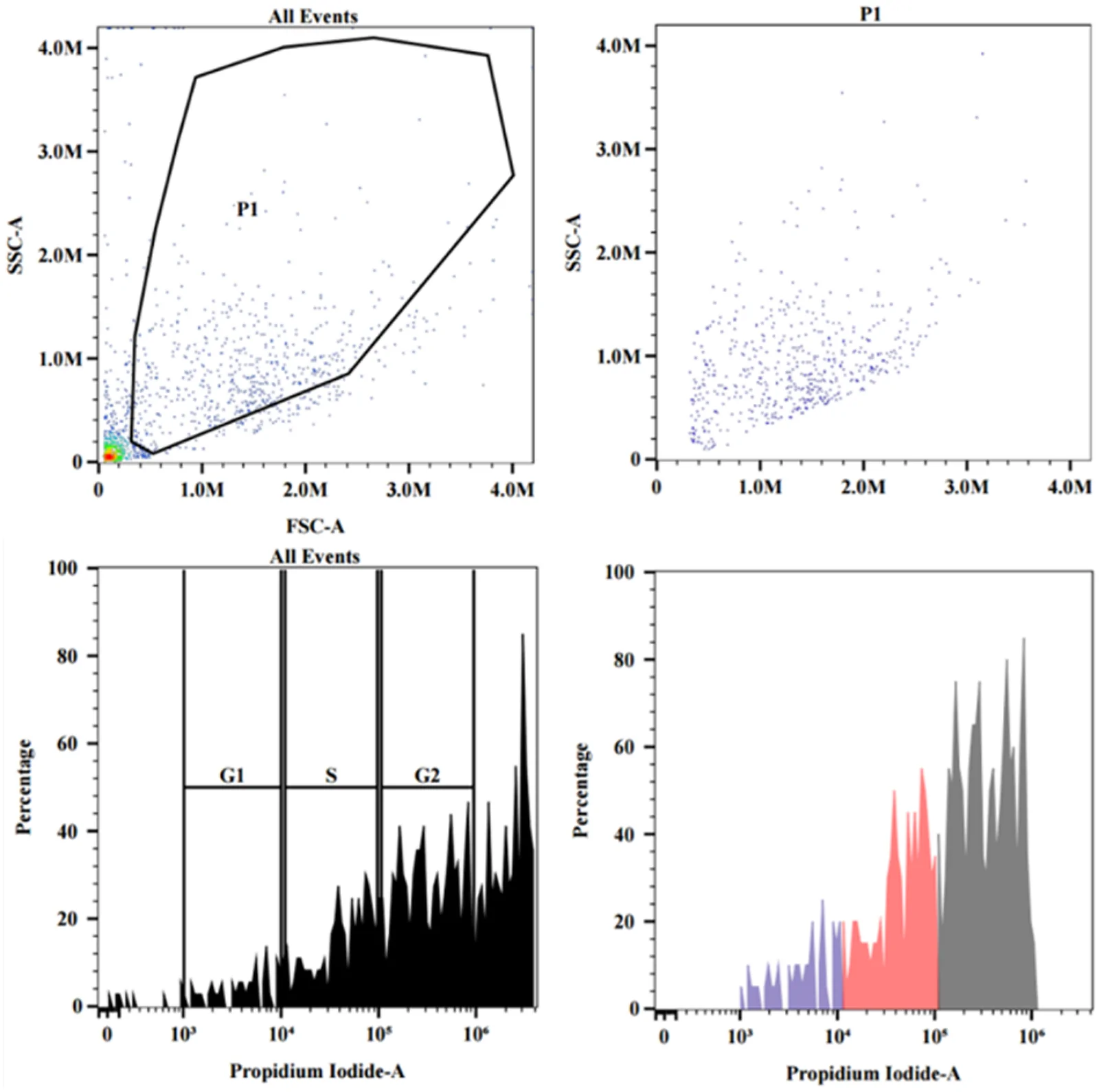
Cell-cycle distribution of MCF-7 cells following treatment with compound 1.

Flow cytometry was conducted to examine the influence of compound 1 on the cell-cycle distribution of MCF-7 cells (Fig. 16). Following treatment, a pronounced alteration in the normal cell-cycle profile was observed. The proportion of cells in the G1 phase decreased to 3.68%, whereas those in the S phase and G2/M phase were 11.27% and 21.63%, respectively. This distribution pattern indicates a marked accumulation of cells in the G2/M phase, indicating G2/M arrest. The substantial reduction in the G1 population alongside the rise in G2/M phase population, implies that the treatment may interfere with DNA replication and mitotic entry. While G2/M arrest is a well-documented cellular response to Topo II-active agents, it is a non-specific phenotypic endpoint also associated with CDK1/cyclin B inhibition, Aurora kinase disruption, spindle assembly checkpoint activation, and other mechanisms unrelated to Topo II. The observed G2/M accumulation therefore supports but does not confirm a Topo II-mediated mechanism, and contributions from alternative targets (including kinases and mitochondrial pathways known to be modulated by kaempferol glycosides) cannot be excluded from the present data.

##### 3.3.3.2. Apoptosis profile analysis

**Fig. 17:**
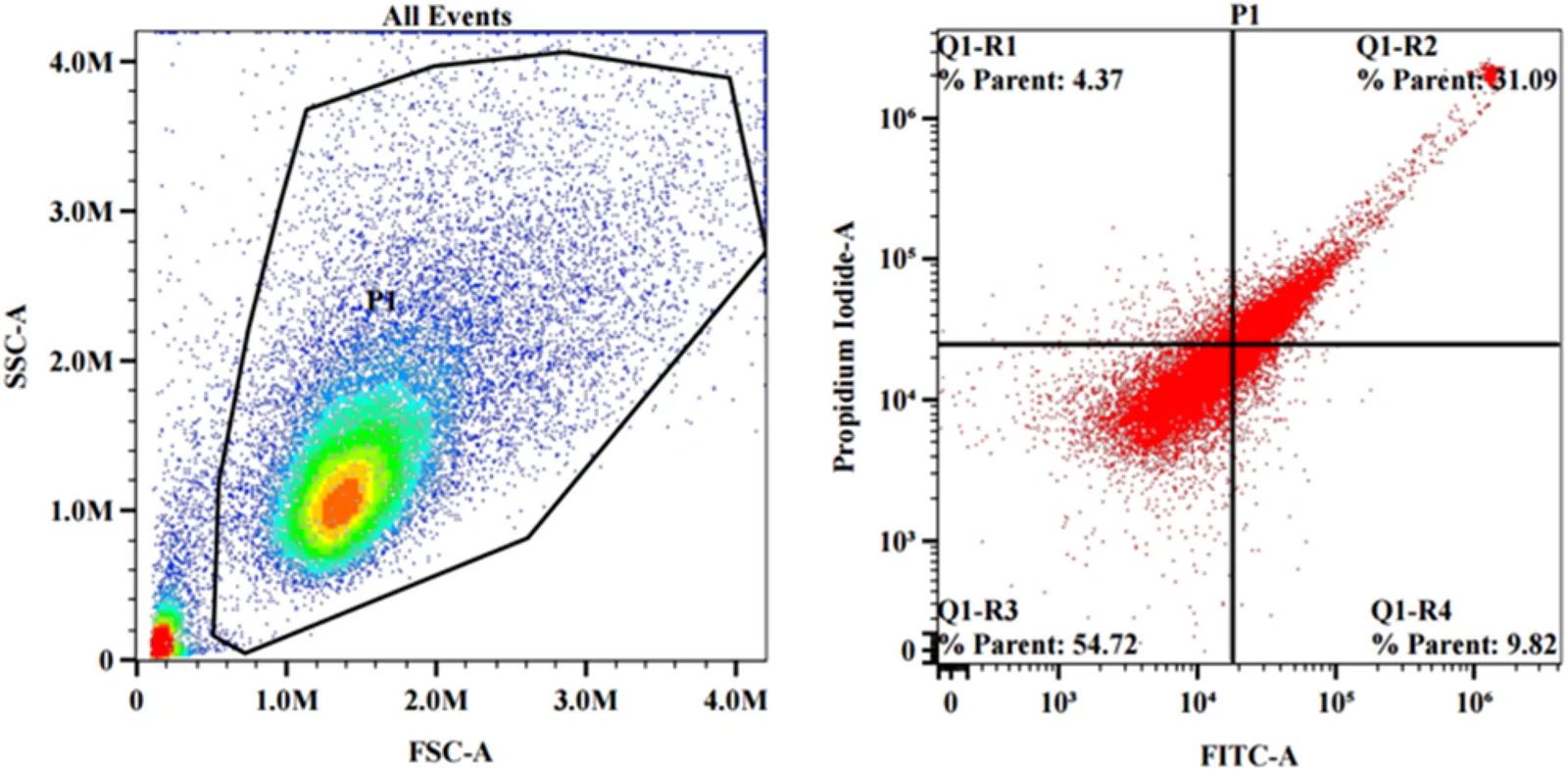
Annexin V/PI apoptosis assay of MCF-7 cells treated with compound 1.

To further investigate whether the observed G2/M arrest was associated with the induction of apoptosis, annexin V/propidium iodide (PI) double-staining flow cytometry was performed. Compound 1 markedly increased the total apoptotic population (early + late apoptosis) to 40.91%, with early apoptosis constituting 9.82% and late apoptosis 31.09%. In addition, the necrotic cell population reached 4.37%. The predominance of late apoptosis suggests that the cytotoxic effect of compound 1 is primarily mediated through apoptosis rather than necrosis. These findings are consistent with the cell-cycle data, indicating that the G2/M accumulation may be linked to DNA damage-induced apoptotic cell death (Fig. 17). Together, these results reveal a cytotoxic profile (G2/M arrest coupled with predominant late apoptosis) that is consistent with a Topo II-related mechanism of action and broadly resembles the phenotypic profile of doxorubicin, though direct mechanistic equivalence cannot be established from the present data. It is further acknowledged that the apoptotic profile observed (predominant late apoptosis with a smaller early apoptotic fraction) is consistent with, but not exclusive to, Topo II-mediated cytotoxicity. Mitochondrial pathway activation, Bcl-2 family modulation, and ROS-driven apoptosis (all documented mechanisms for kaempferol and its glycosides) would produce an indistinguishable annexin V/PI staining profile without pathway-specific mechanistic assays.

## 4. Conclusion

The first phytochemical investigation of *A. guachapele* leaves yielded twelve isolates, with the primary chemical novelty residing in two previously undescribed kaempferol tetraglycosides, Guachapins A (3) and B (4), representing the first natural products characterized from this species and expanding the known structural diversity of flavonol glycosides. The biological evaluation focused on the major known isolate, mauritianin (1), providing its first Topo II-focused mechanistic characterization. Molecular docking predicted strong binding of compound 1 to the DNA–Topo II active site (Δ*G* = −33.48 kcal mol^−1^), with MD simulations confirming the conformational stability of the predicted complex over 200 ns. *In vitro*, compound 1 exhibited potent antiproliferative activity against MCF-7 cells (IC_50_ = 10.28 µM), alongside concentration-dependent reduction of intracellular Topo II alpha protein levels, G2/M phase accumulation, and a predominantly late apoptotic cell death profile. Selectivity index analysis confirmed preferential toxicity toward MCF-7 and MDA-MB-231 over normal cell lines (SI = 5.47–6.02 and 4.82–5.31, respectively), supporting compound 1 as a phytochemical lead scaffold for structural optimization. Collectively, these convergent findings identify compound 1 as a Topo II-associated antiproliferative lead candidate with a cellular phenotype consistent with a putative Topo II-related mechanism of cytotoxicity.

Several limitations of the present study should be acknowledged. First, no direct functional Topo II assays were performed (including plasmid DNA relaxation, decatenation, cleavage complex trapping (ICE bioassay), comet assay, or γ-H2AX immunostaining) that would be required to distinguish between Topo II poisoning, catalytic inhibition, non-specific DNA binding, or general cytotoxicity as the operative mechanism; the biological findings are therefore interpreted as hypothesis-generating rather than mechanistically conclusive. Second, the ELISA-based Topo II alpha protein quantification is subject to interpretive ambiguity (reduced signal may reflect expression downregulation, enhanced protein degradation, or epitope interference) and cannot substitute for functional catalytic assays. Third, compound 1 was evaluated against a single computational target, and given the well-documented multi-target promiscuity of kaempferol glycosides (including CDK inhibition, PI3K/Akt modulation, mitochondrial perturbation, and ROS modulation) contributions from alternative targets to the observed cytotoxic phenotype cannot be excluded; multi-target docking, network pharmacology analysis, and target-specific assays are identified as future priorities. Fourth, pathway-level apoptosis characterization (caspase-3/7/8/9 activation, Bax/Bcl-2 ratio, cytochrome c release, and PARP cleavage) was not performed and remains necessary to identify the specific apoptotic cascade engaged. Finally, no ADMET or pharmacokinetic assessment was conducted; the triglycosidic structure of compound 1 presents inherent drug-likeness liabilities (elevated MW, high TPSA, and predicted low GI absorption) precluding direct clinical translation of the intact molecule, and semi-synthetic structural optimization is identified as an essential next step. Finally, whether the observed antiproliferative activity originates from the intact triglycosidic molecule or from intracellular deglycosylation products (including the kaempferol aglycone) remains unresolved, and metabolite profiling of treated cell lysates alongside direct aglycone comparison studies are identified as essential future experiments.

## Author contributions

M. S.: writing, conceptualization, methodology, investigation, data curation, analysis of spectral data, resources, visualization. A. M. M.: supervision, conceptualization, methodology, biological investigation, data curation, review & editing, resources. I. E. H. and F. G. A.: methodology, visualization, docking and MD studies. E. A. R.: supervision, conceptualization, methodology, data curation, analysis of spectral data, project administration, review & editing.

## Conflicts of interest

The authors declare no competing interests.

## Supplementary Material

RA-016-D6RA03441A-s001

## Data Availability

All data generated or analyzed during this study are contained within the published article and the associated supplementary information (SI). Supplementary information: spectral characterization data (NMR, MS, UV) for the isolated compounds, along with detailed methods and supporting data for the *in silico* (docking, MD simulation, MM-GBSA) and *in vitro* (cytotoxicity, topoisomerase inhibition, flow cytometry) experiments described in the manuscript. See DOI: https://doi.org/10.1039/d6ra03441a.
