# Supplementary material for: Flavonol tri- and tetraglycosides from Albizia guachapele Baker: isolation and biological evaluation
Source: RSC Adv. 2026 Jul 20;16(38):41833–52. doi: 10.1039/d6ra03441a (PMC13384234; doi:10.1039/d6ra03441a)
Supplement: Supplementary file 1 [file RA-016-D6RA03441A-s001.pdf]

This journal is © The Royal Society of Chemistry 2026

Supplementary Data

Compounds Identification

Compound (1)

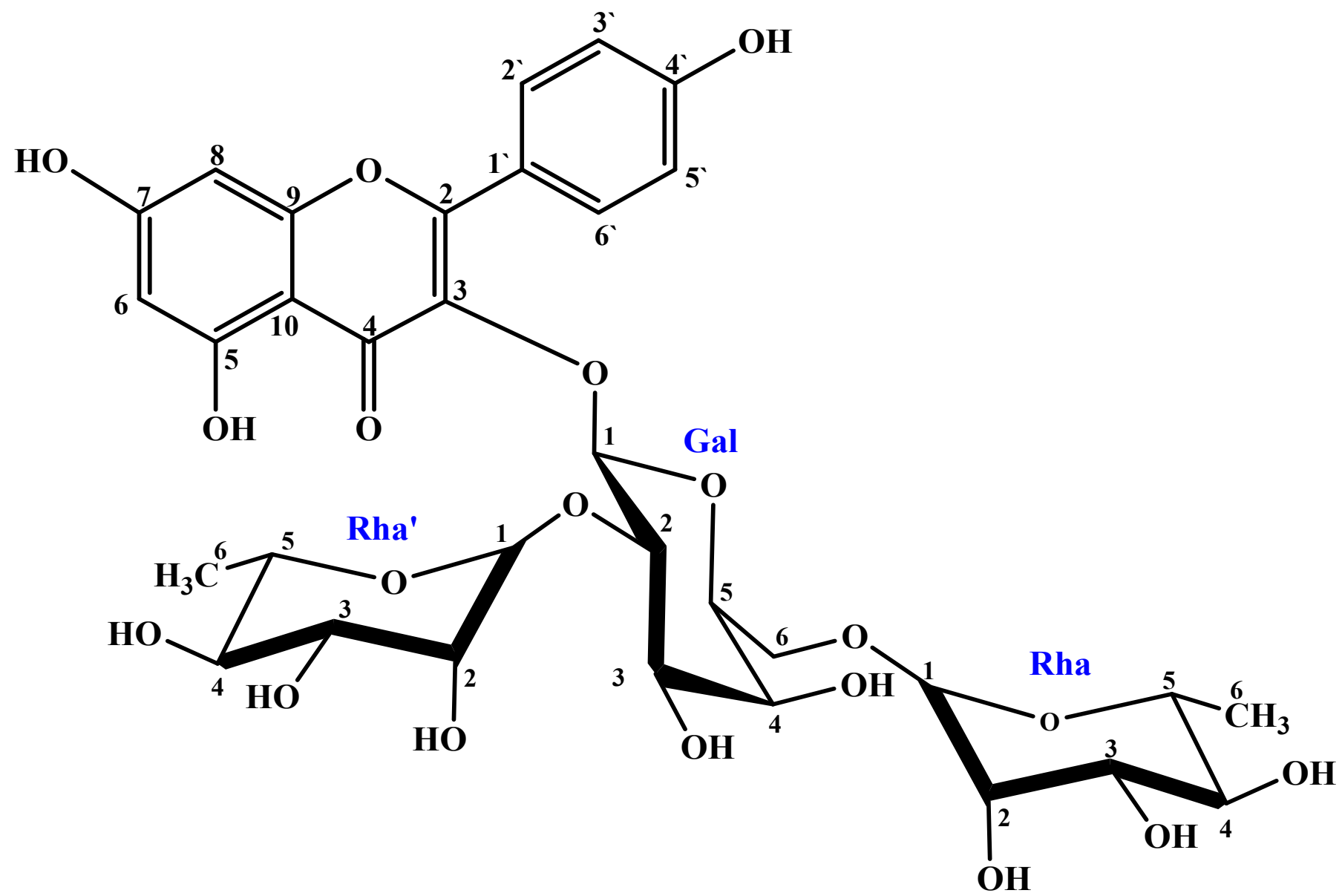

Kaempferol-3-*O*-(2,6-di-*O*-*α*-L-rhamnopyranosyl)-*β*-D-galactopyranoside

Compound **1**:	Kaempferol-3-*O*-(2,6-di-*O*-*α*-L-rhamnopyranosyl)-*β*-D-galactopyranoside (Mauritianin)
Molecular formula:	$C_{33}H_{40}O_{19}$
Molecular weight:	740
+ve ESI–MS *m/z*:	763 $[M+Na]^+$
	741 $[M+H]^+$
	595 $[M+H\text{-}Rha]^+$
	449 $[M+H\text{-}2Rha]^+$
	287 $[M+H\text{-}2Rha\text{-}Gal]^+$
-ve ESI–MS *m/z*:	739 $[M-H]^-$

Table S1: 1D and 2D NMR spectral data of compound **1** (400 MHz for ^{1}H, 100 MHz for ^{13}C, DMSO-d_6).

Position	^{1}H (J in Hz)	^{13}C	COSY	HMBC
**Aglycone**				
2	-	156.55	-	-
3	-	132.58	-	-
4	-	177.10	-	-
5	-	161.23	-	-
6	6.13, d, 1.6	99.23	H-8	C-5, 7, 8, 10
7	-	165.9	-	-
8	6.34, d, 1.6	94.00	H-6	C-6, 9, 10
9	-	156.07	-	-
10	-	103.35	-	-
1′	-	121.03	-	-
2′	8.03, d, 8.8	130.78	H-3′	C-2, 6′, 4′
3′	6.85, d, 8.8	115.13	H-2′	C-1′
4′	-	159.90	-	-
5′	6.85, d, 8.8	115.13	H-6′	C-1′
6′	8.03, d, 8.8	130.78	H-5′	C-2, 2′, 4′
5-OH	12.66, brs	-	-	-
**Gal**				
1	5.56, d, 7.7	99.01	H-2	-
2	3.78, m	74.92	H-3	C-1 of Rha′
3	3.59, m	73.87	H-2, 4	-
4	3.59, m	68.58	H-3, 5	C-3, 5
5	3.56, m	73.34	H- 4, 6	C-6
6a<br>6b	3.56, m<br>3.22, m	65.17	H-5	C-1 of Rha
**Rha**				
1	4.36, s	100.12	H-2	C-6 of Gal, C-5, C-3
2	3.38, m	70.46	H-1	C-3
3	3.30, dd, 9.3, 3.3	70.73	H-2	C-1
4	3.09, t, 9.3	71.95	H-3, 5	C-5
5	3.33, d, 6.2	68.33	H-4	C-4
6	1.05, d, 6.2	17.97	H-5	C-4, 5

Rha′				
1	5.05, s	100.64	H-2	C-2 of Gal, C-3, C-5
2	3.75, m	70.46	H-1	C-3
3	3.50, dd, 9.3, 3.2	70.65	H-2	C-1
4	3.13, t, 9.3	71.95	H-3, 5	C-3, 5
5	3.77, m	68.22	H-4	-
6	0.79, d, 6.0	17.31	H-5	C-4, 5

Table S2: UV Spectral Data for compounds **1-4**.

No.	MeOH		NaOMe		$AlCl_3$		$AlCl_3$/HCl		NaOAc		NaOAc/H_3BO_3	
	**Band I**	**Band II**	**Band I**	**Band II**	**Band I**	**Band II**	**Band I**	**Band II**	**Band I**	**Band II**	**Band I**	**Band II**
**1**	347	264	400	273	402	274	399	274	385	274	350	265
**2**	355	256	405	272	431	275	397	272	391	268	376	261
**3**	348	266	401	274	400	273	395	272	384	275	350	266
**4**	333	267	390	270	393	270	373	270	340	275	333	267

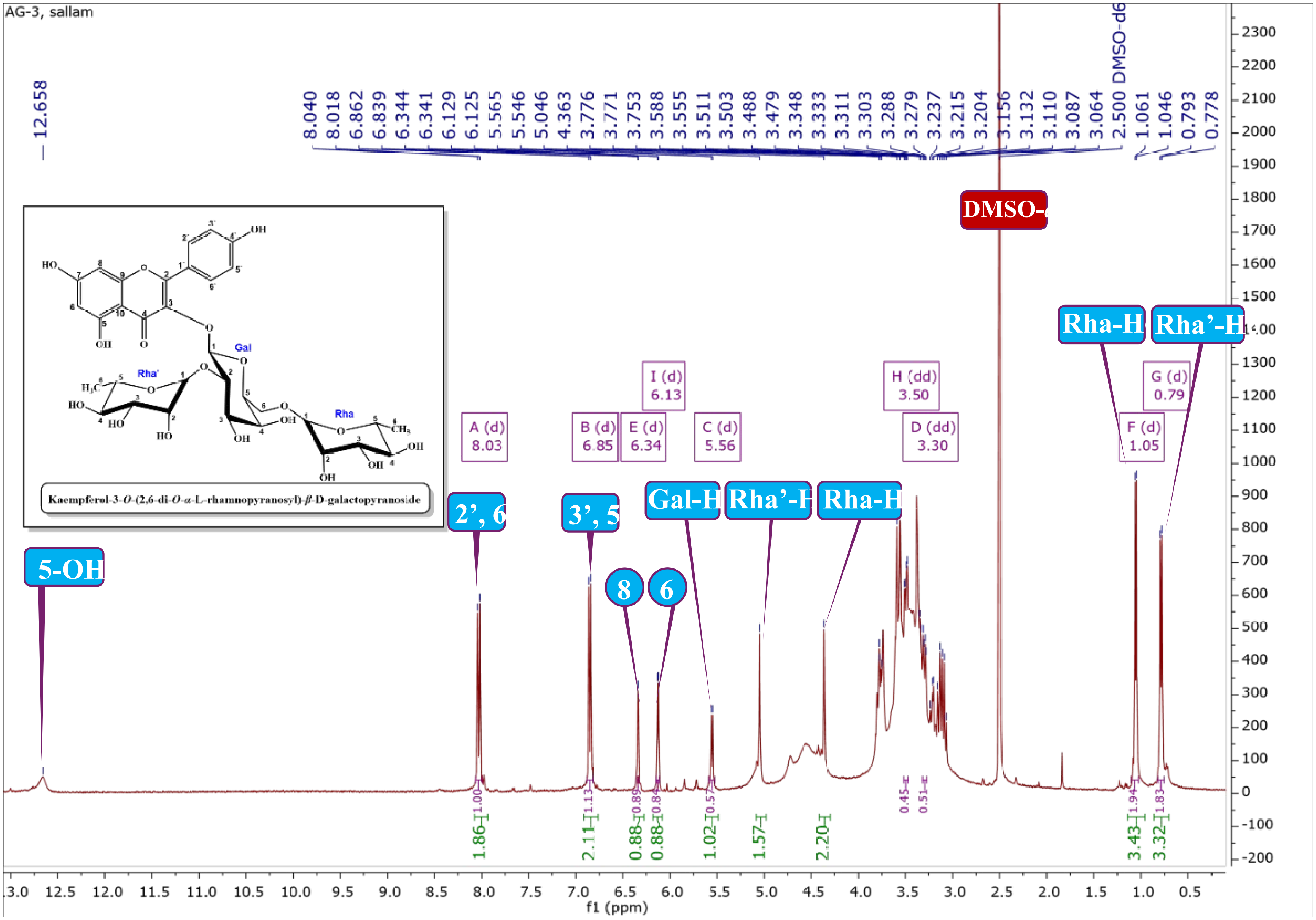

Figure S1: ^{1}H NMR spectrum of compound **1** (DMSO-d_6, 400 MHz).

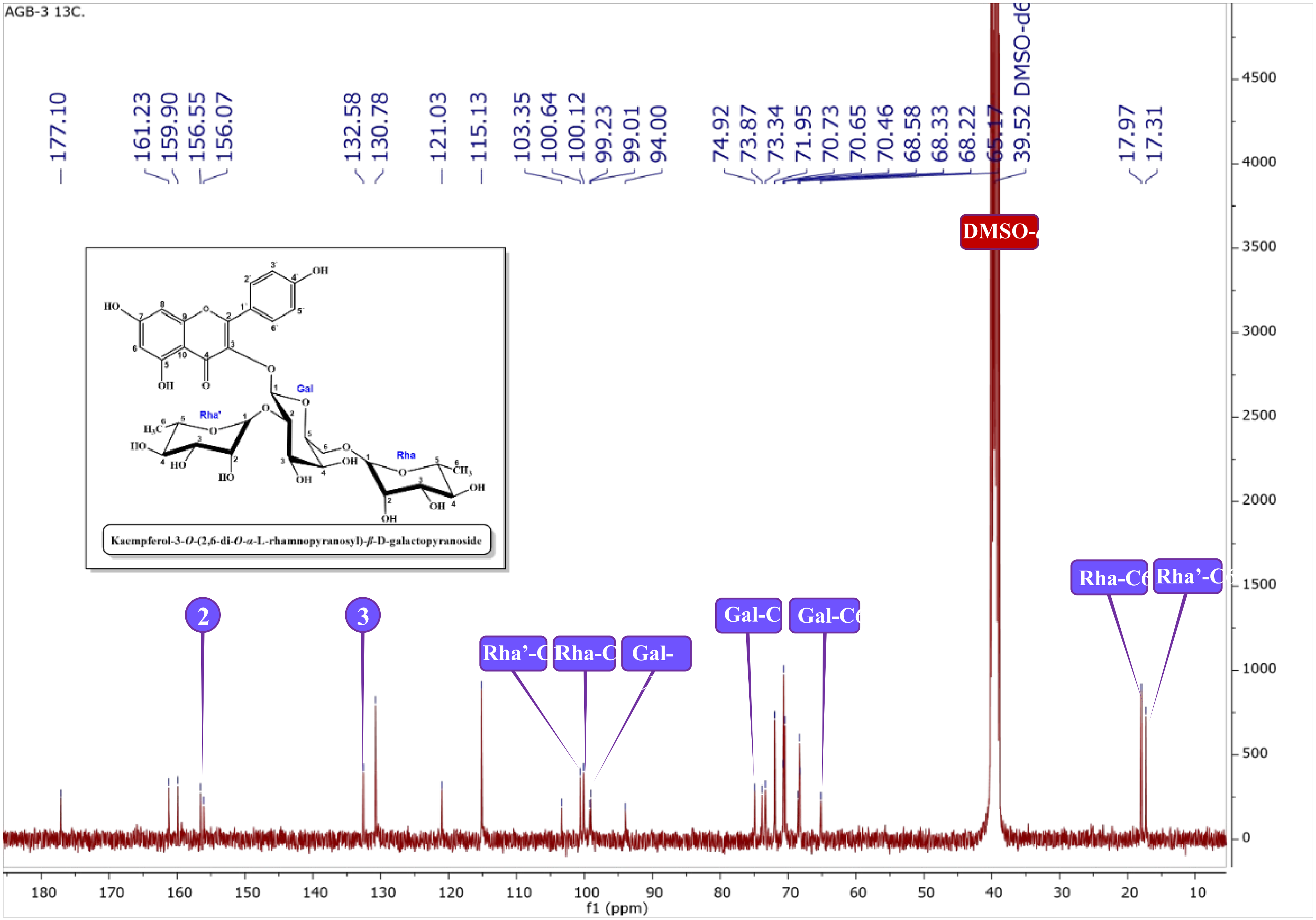

Figure S2: ^{13}C NMR spectrum of compound **1** (DMSO-d_6, 100 MHz).

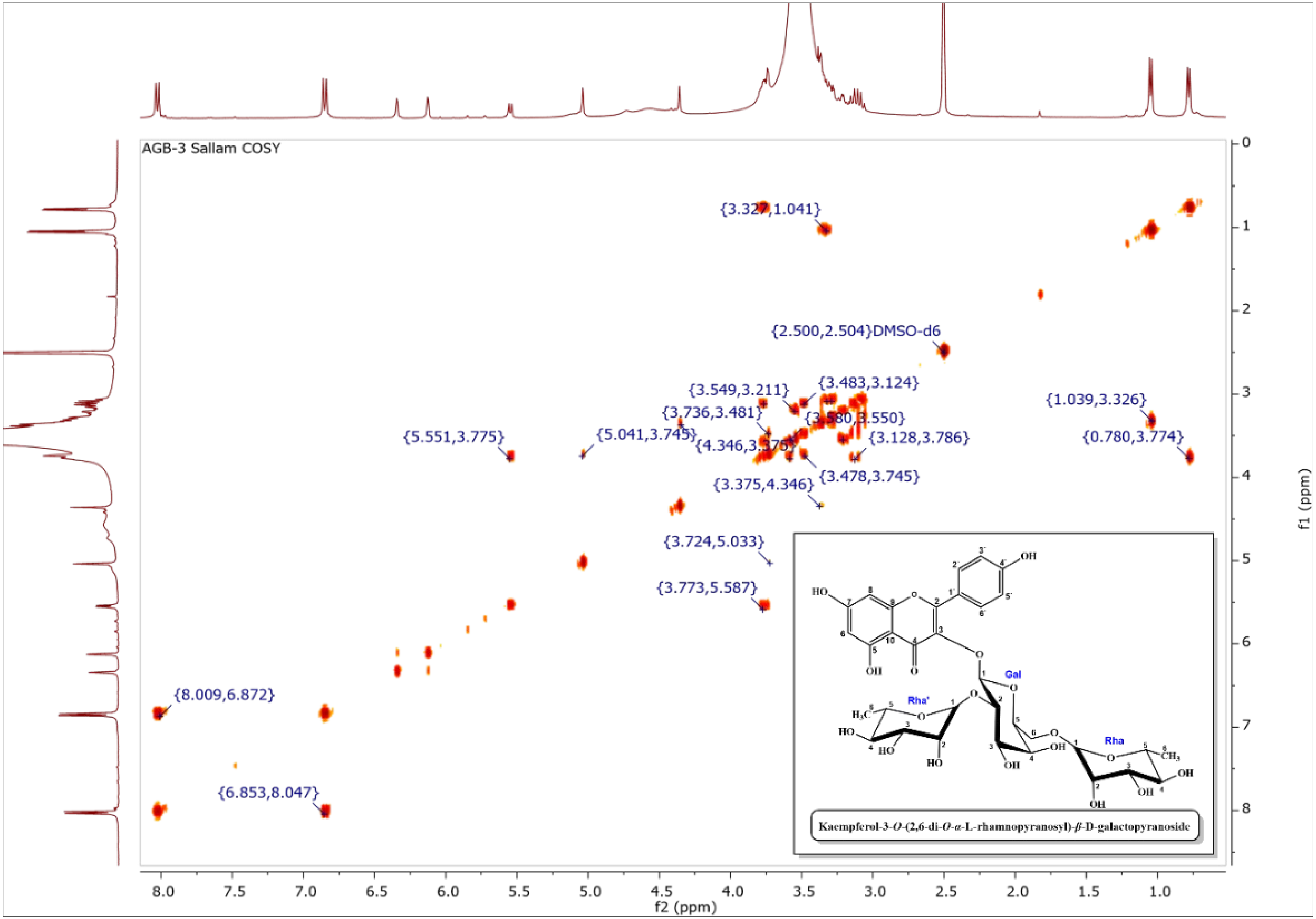

Figure S3: COSY spectrum of compound **1**.

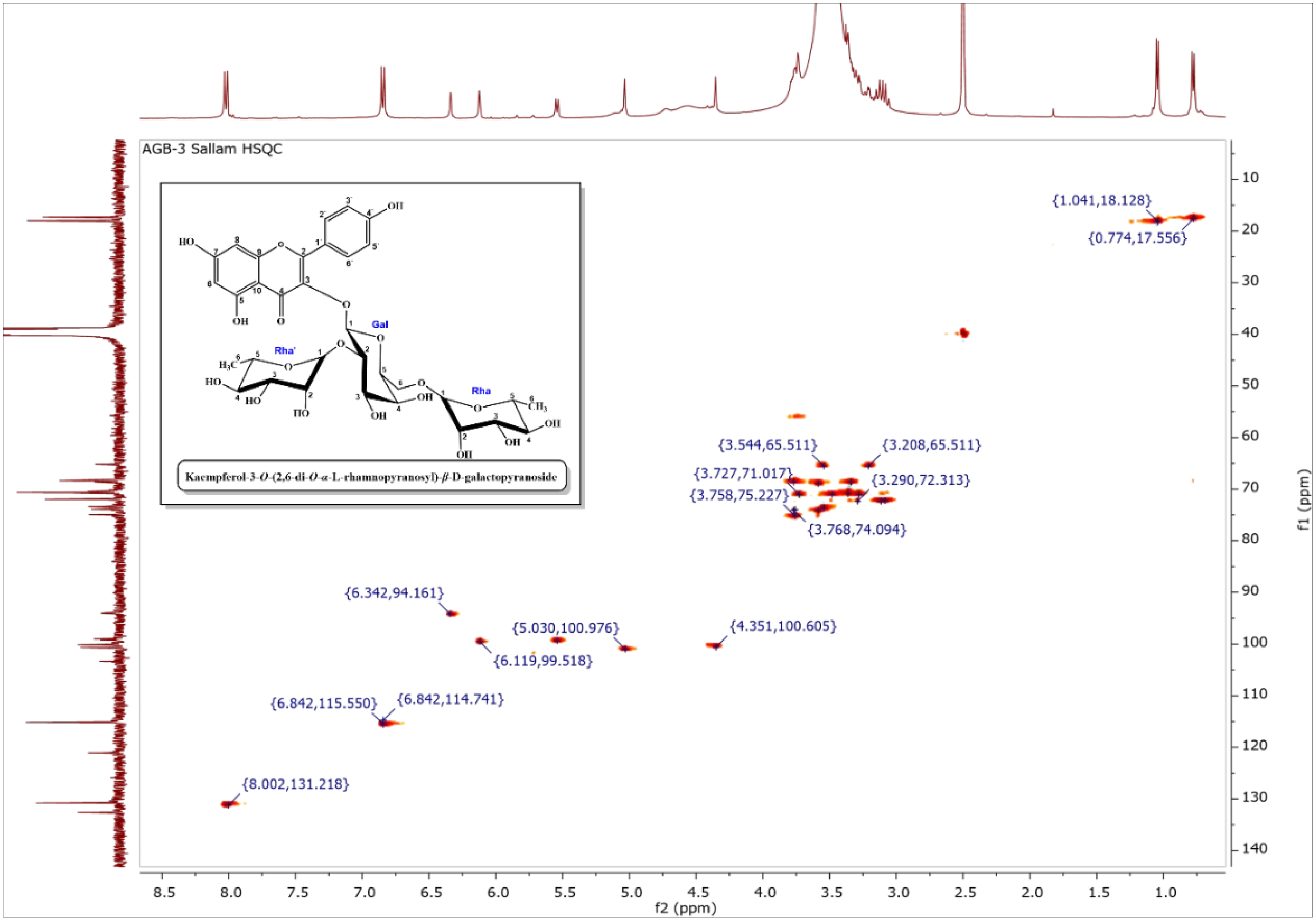

Figure S4: HSQC spectrum of compound **1**.

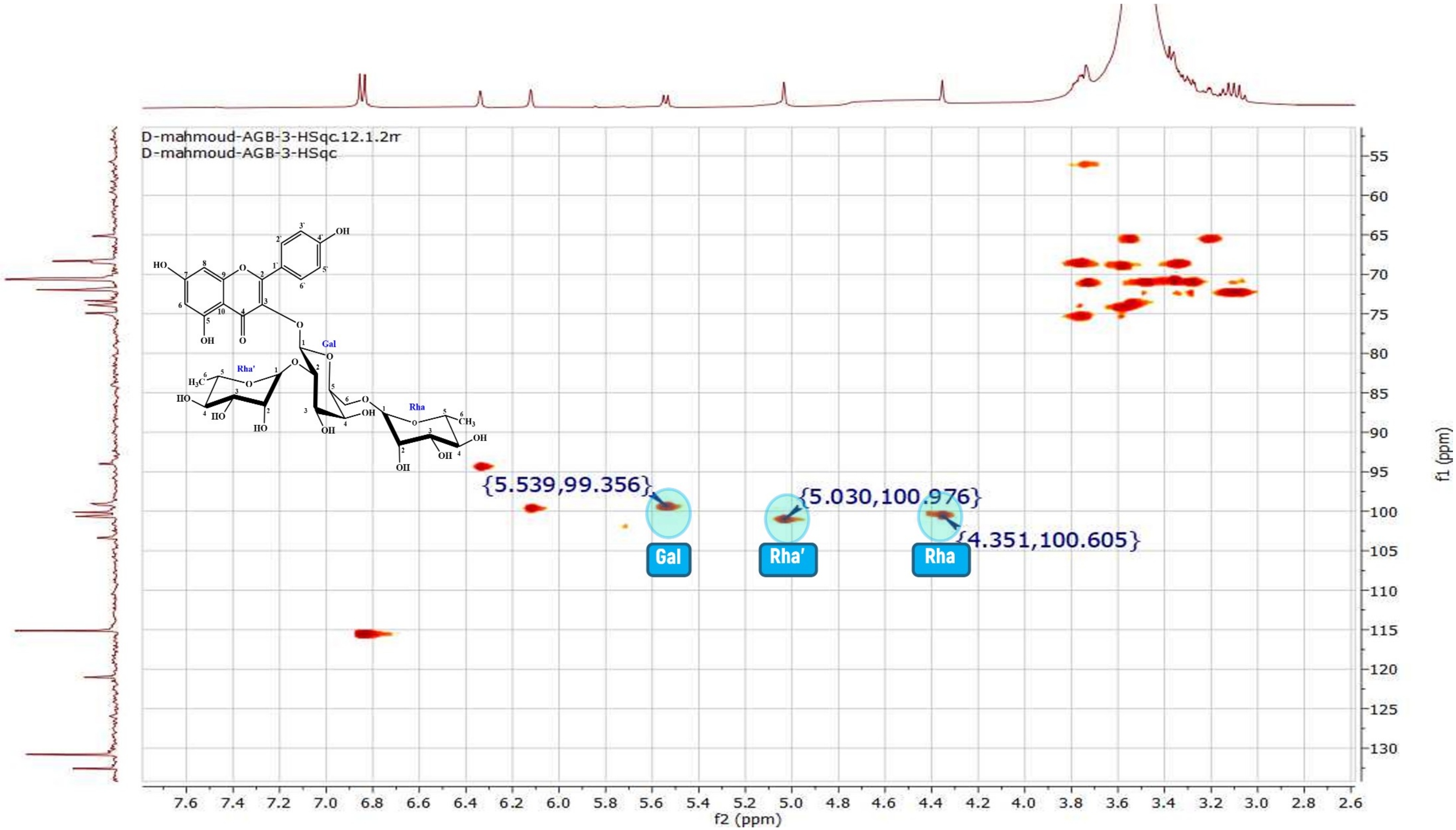

Figure S5: Expanded HSQC spectrum of compound **1**.

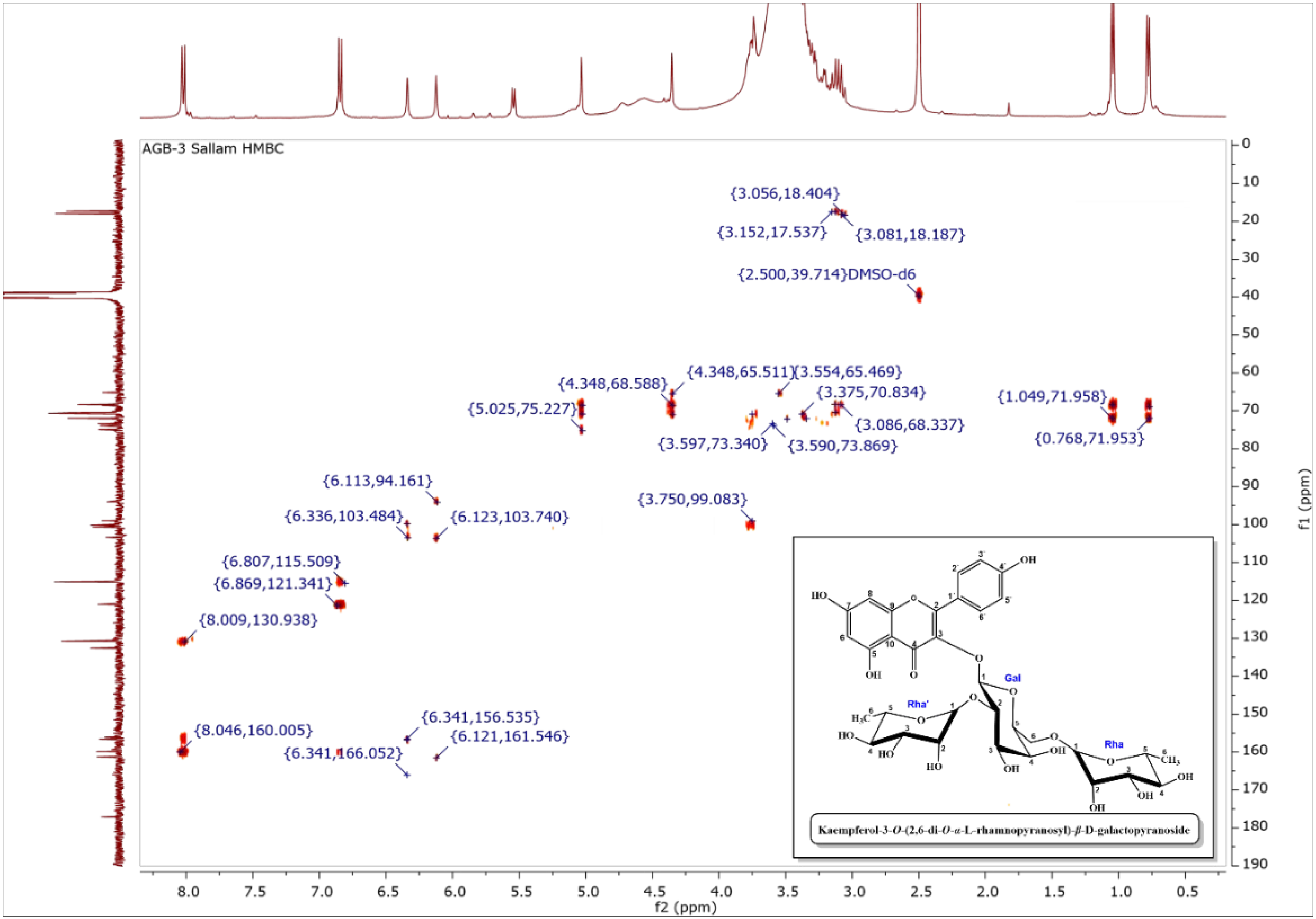

Figure S6: HMBC spectrum of compound **1**.

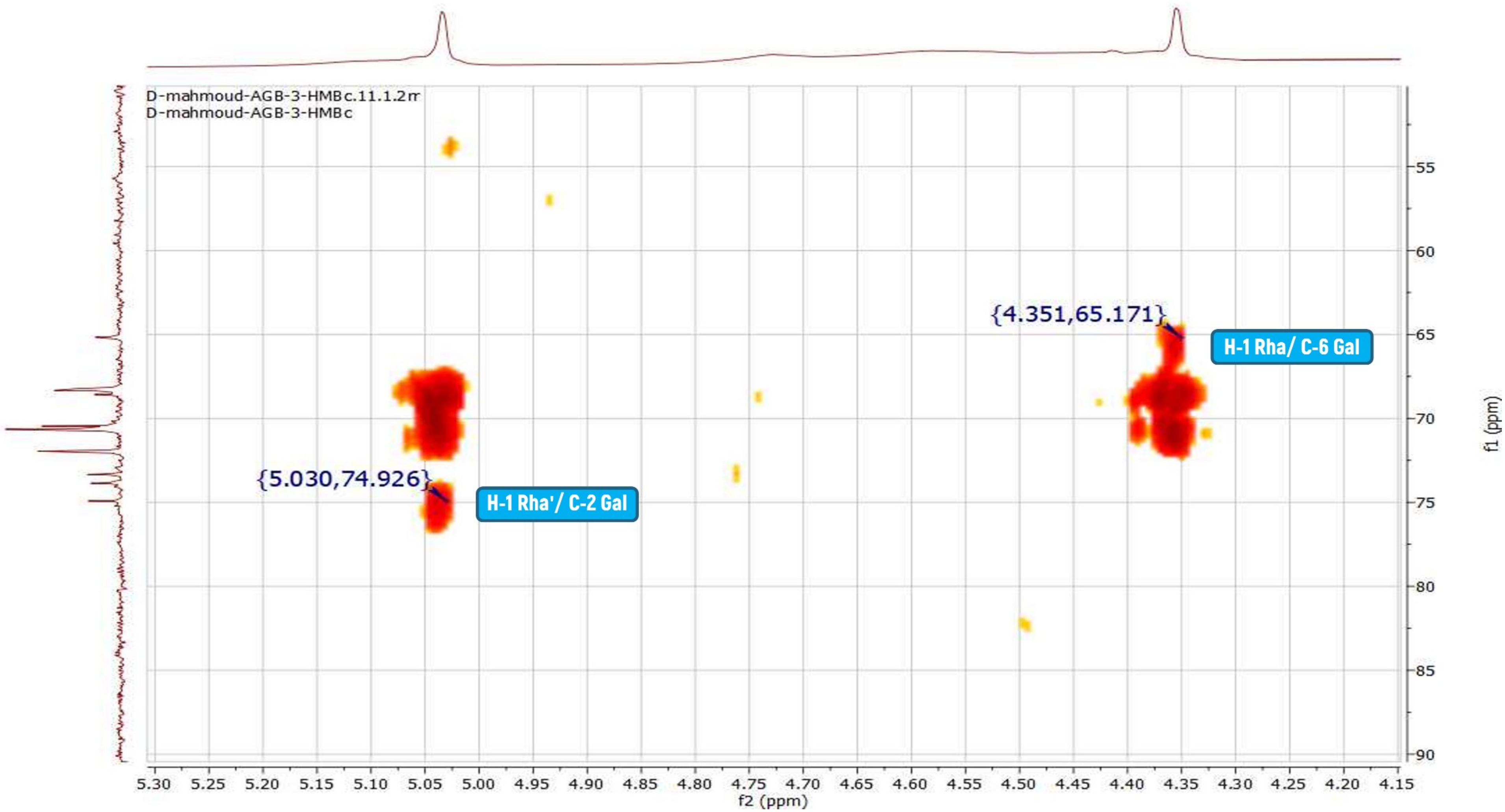

Figure S7: Expanded HMBC spectrum of compound **1**.

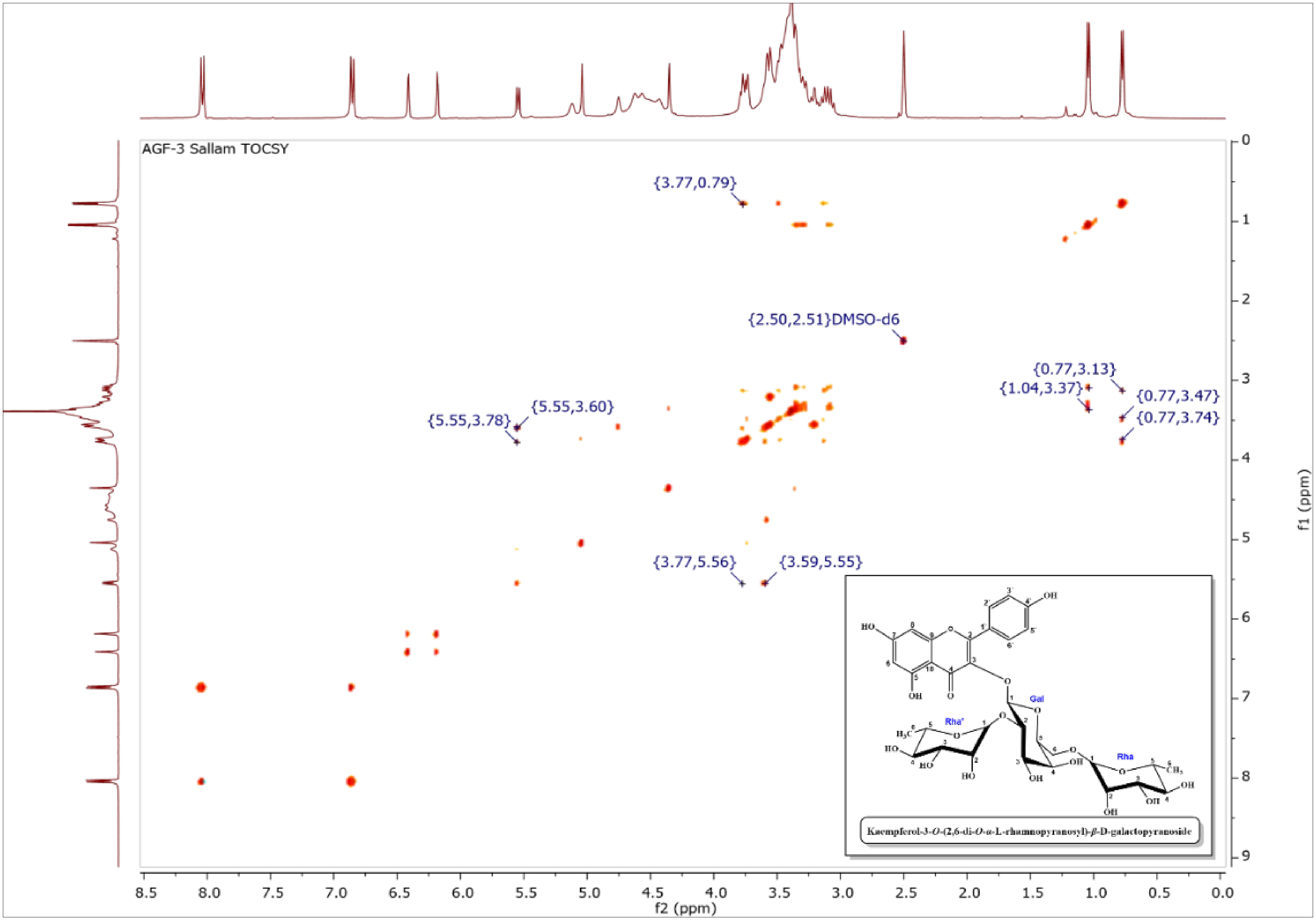

Figure S8: TOCSY spectrum of compound **1**.

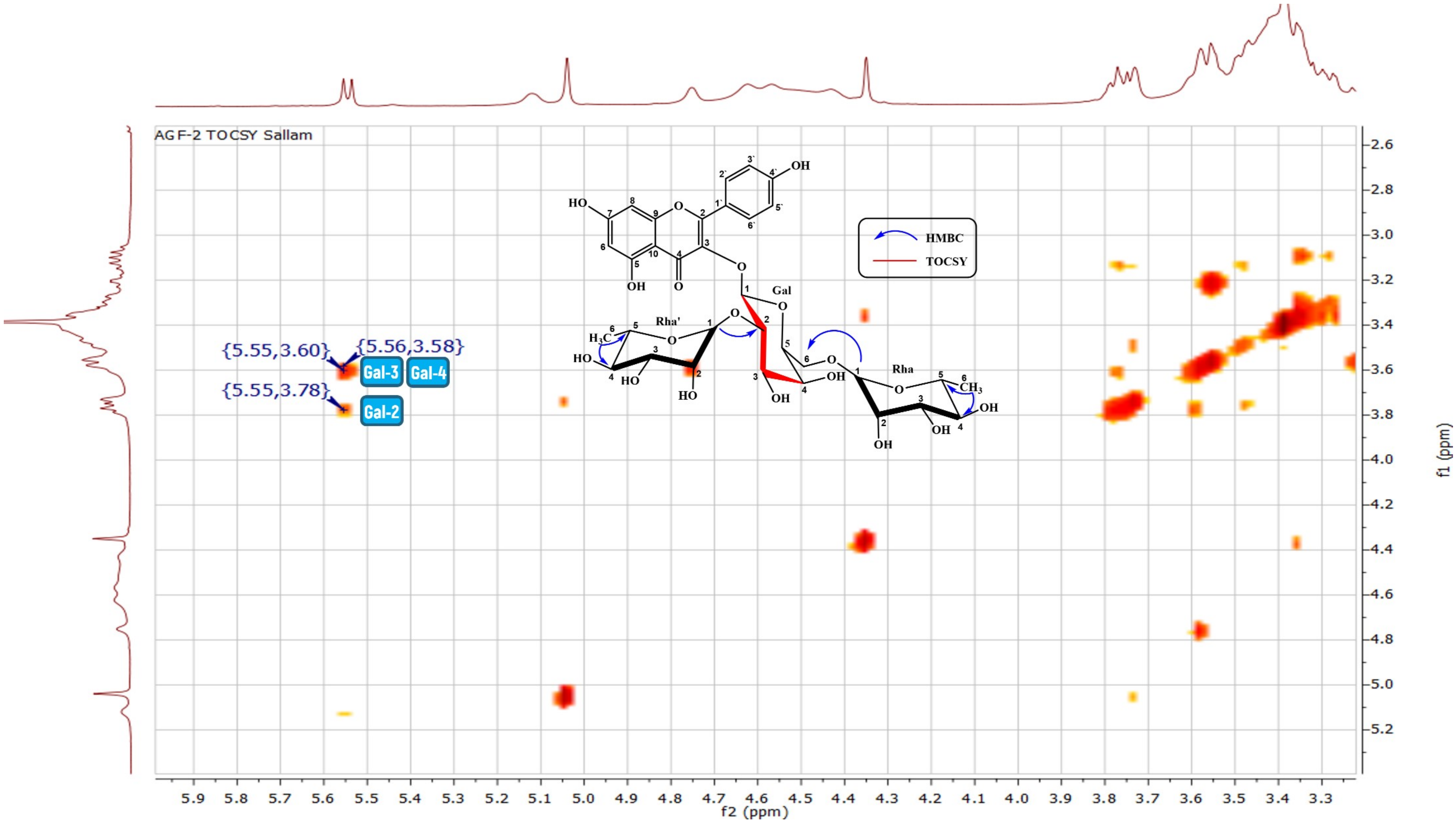

Figure S9: Expanded TOCSY spectrum of compound **1**.

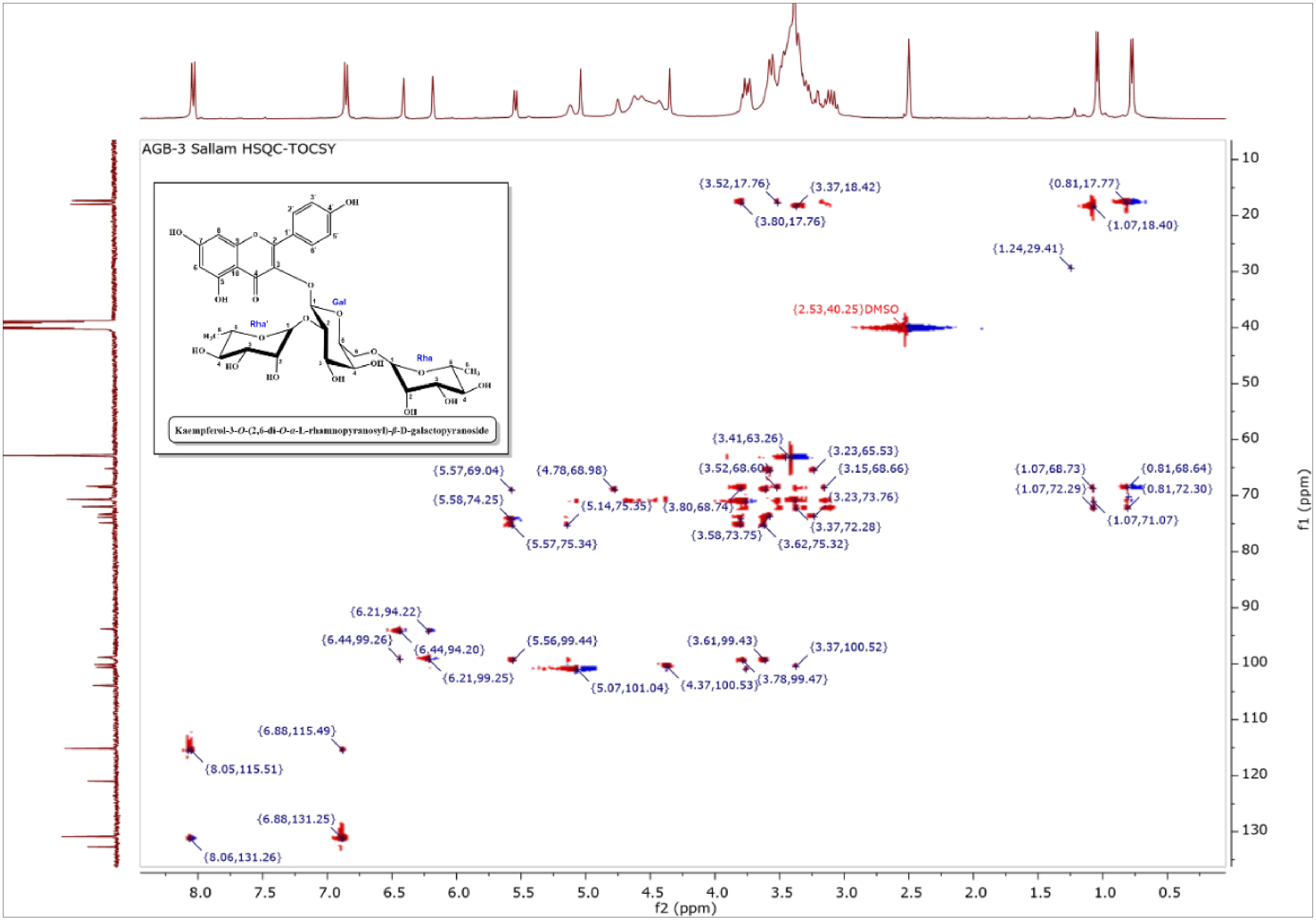

Figure S10: HSQC–TOCSY spectrum of compound **1**.

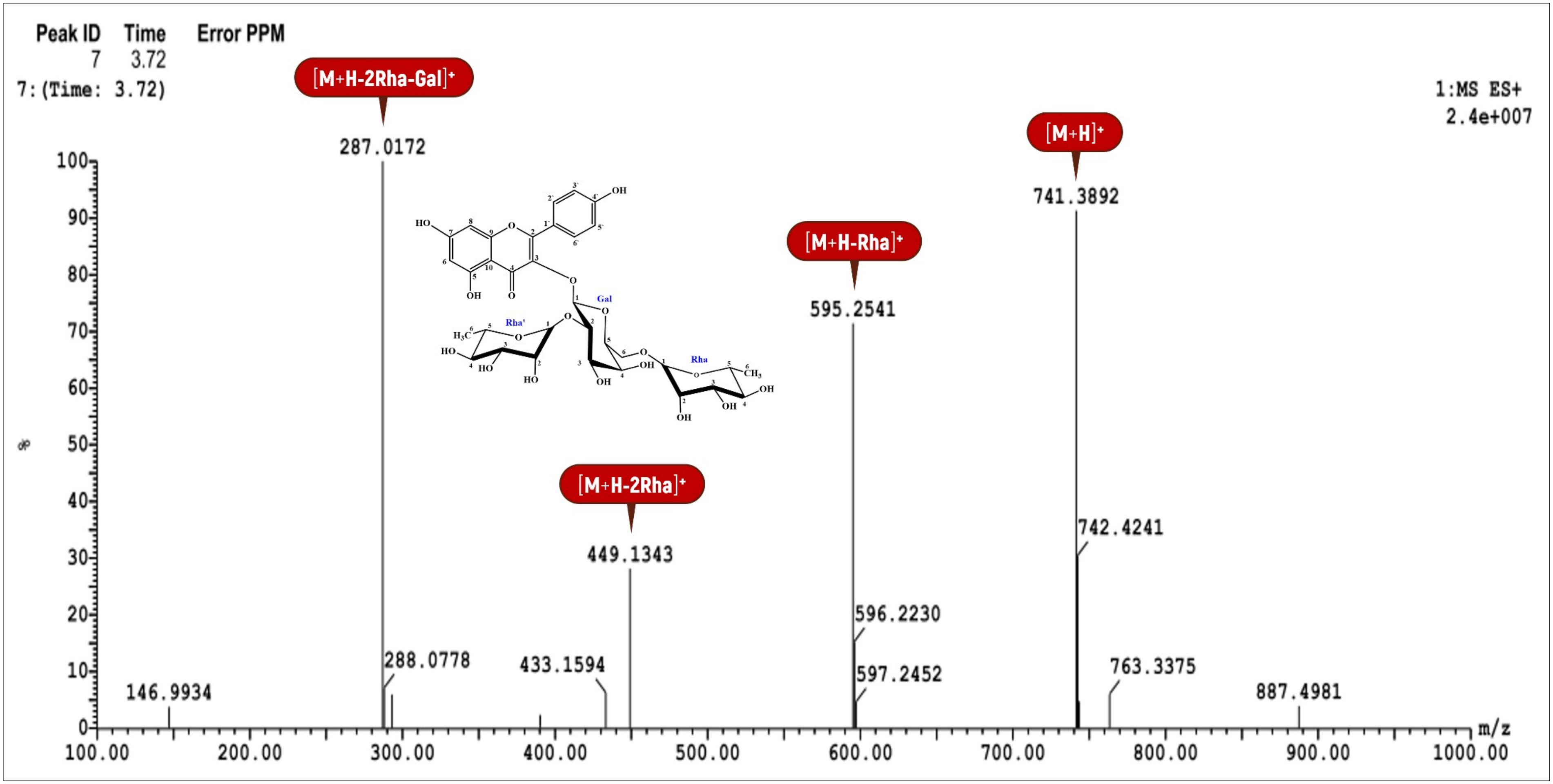

Figure S11: Positive ESI–MS spectrum of compound **1.**

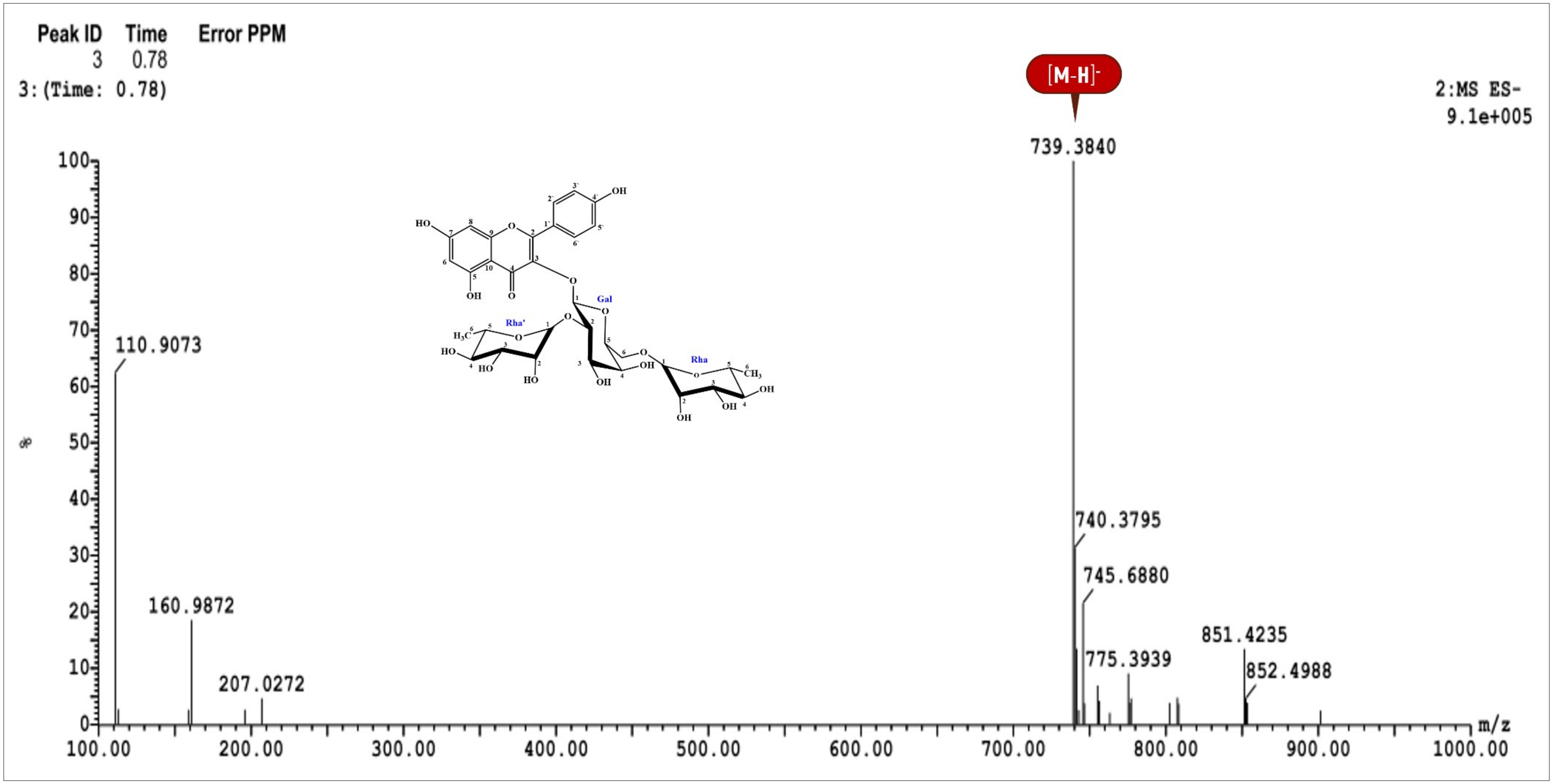

Figure S12: Negative ESI–MS spectrum of compound **1**.

Compound (2)

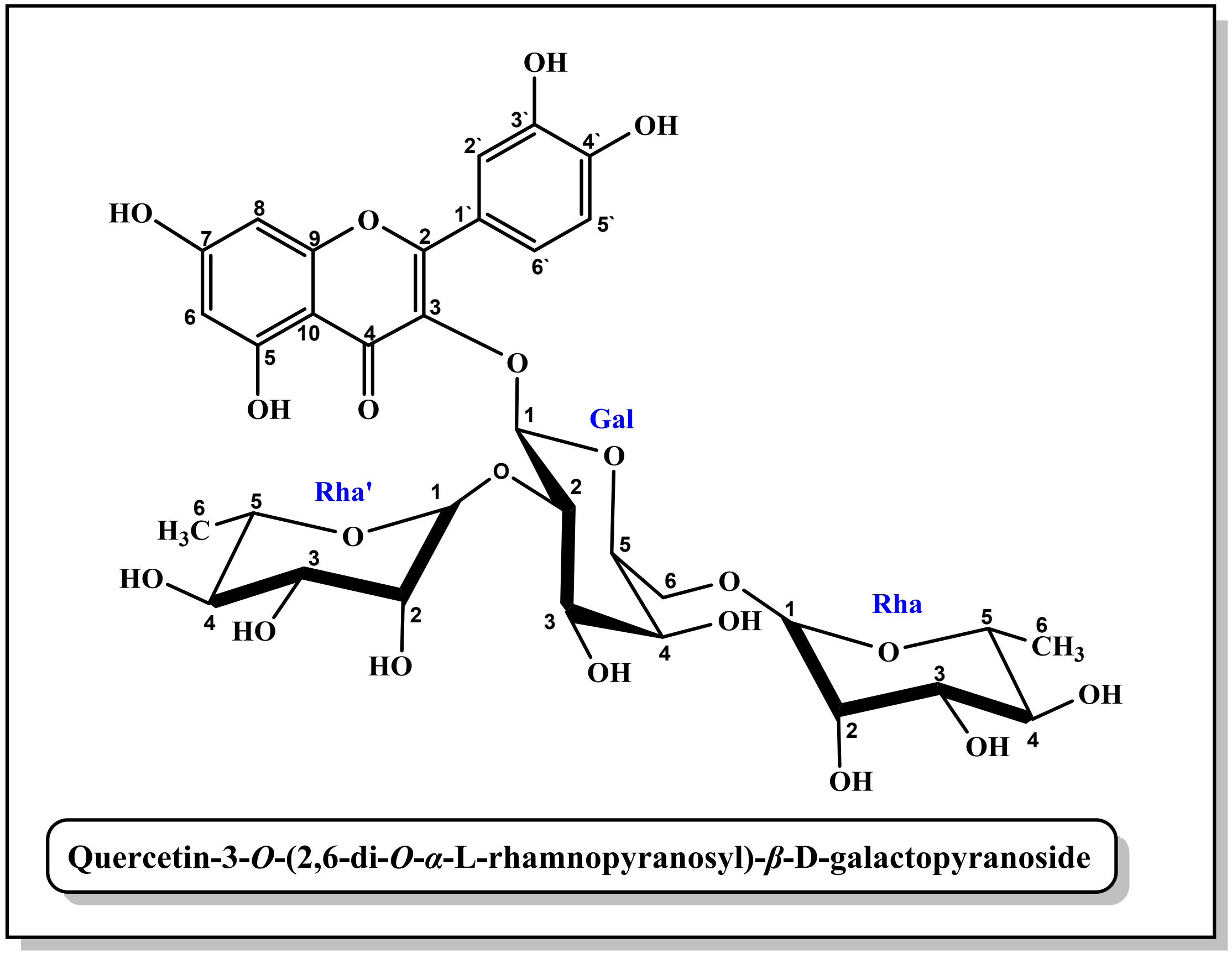

Compound **2**:	Quercetin-3-*O*-(2,6-di-*O*-*α*-L-rhamnopyranosyl)-*β*-D-galactopyranoside
Molecular weight:	756
Molecular formula:	$C_{33}H_{40}O_{20}$
+ve ESI–MS *m/z*:	757 $[M+H]^+$
	611 $[M+H\text{-}Rha]^+$
	303 $[M+H\text{-}2Rha\text{-}Gal]^+$

Table S3: 1D and 2D NMR spectral data of compound **2** (400 MHz for ^{1}H, 100 MHz for ^{13}C, DMSO-d_6).

Position	^{1}H (J in Hz)	^{13}C	COSY	HMBC
**Aglycone**				
2	-	156.72	-	-
3	-	133.30	-	-
4	-	177.67	-	-
5	-	161.71	-	-
6	6.19, d, 2.0	99.15		C-5, C-8, C-10
7	-	164.65	-	-
8	6.40, d, 2.0	93.96		C-6, C-7, C-9, C-10
9	-	156.66	-	-
10	-	104.32	-	-
1′	-	121.56	-	-
2′	7.50, d, 2.2	116.16	H-6′	C-2, C-3′, C-4′, C-6′
3′	-	145.33	-	-
4′	-	148.87	-	-
5′	6.82, d, 8.5	115.62	H-6′	C-1′, C-3′
6′	7.69, dd, 8.5, 2.2	122.50	H-2′, H-5′	C-2′, C-4′
5-OH	12.71, brs	-		
**Gal**				
1	5.59, d, 7.7	99.45	H-2	
2	3.82, m	75.24	H-1	C-1 of Rha′
3	3.62, m	74.36		
4	3.60, d, 3.2	69.01		
5	3.56, m	73.74		
6a<br>6b	3.57, m<br>3.22, m	65.35		 C-1 of Rha
**Rha**				
1	4.40, s	100.45		C-6 of Gal, C-5, C-3
2	3.37*	70.89		
3	3.29, dd, 9.2, 2.8	71.04		
4	3.09, t, 9.2	72.35		
5	3.35*	68.75	H-6	
6	1.06, d, 6.2	18.40	H-5	C-4, C-5

**Rha′**				
1	5.07, s	101.00		C-2 of Gal, C-3, C-5
2	3.74, m	70.89		
3	3.50, dd, 9.2, 2.8	71.04		
4	3.14, t, 9.2	72.35		
5	3.78, m	68.64	H-6	
6	0.81, d, 6.2	17.37	H-5	C-4, C-5

* The signals masked by water peak and the assignments were obtained from HSQC spectrum.

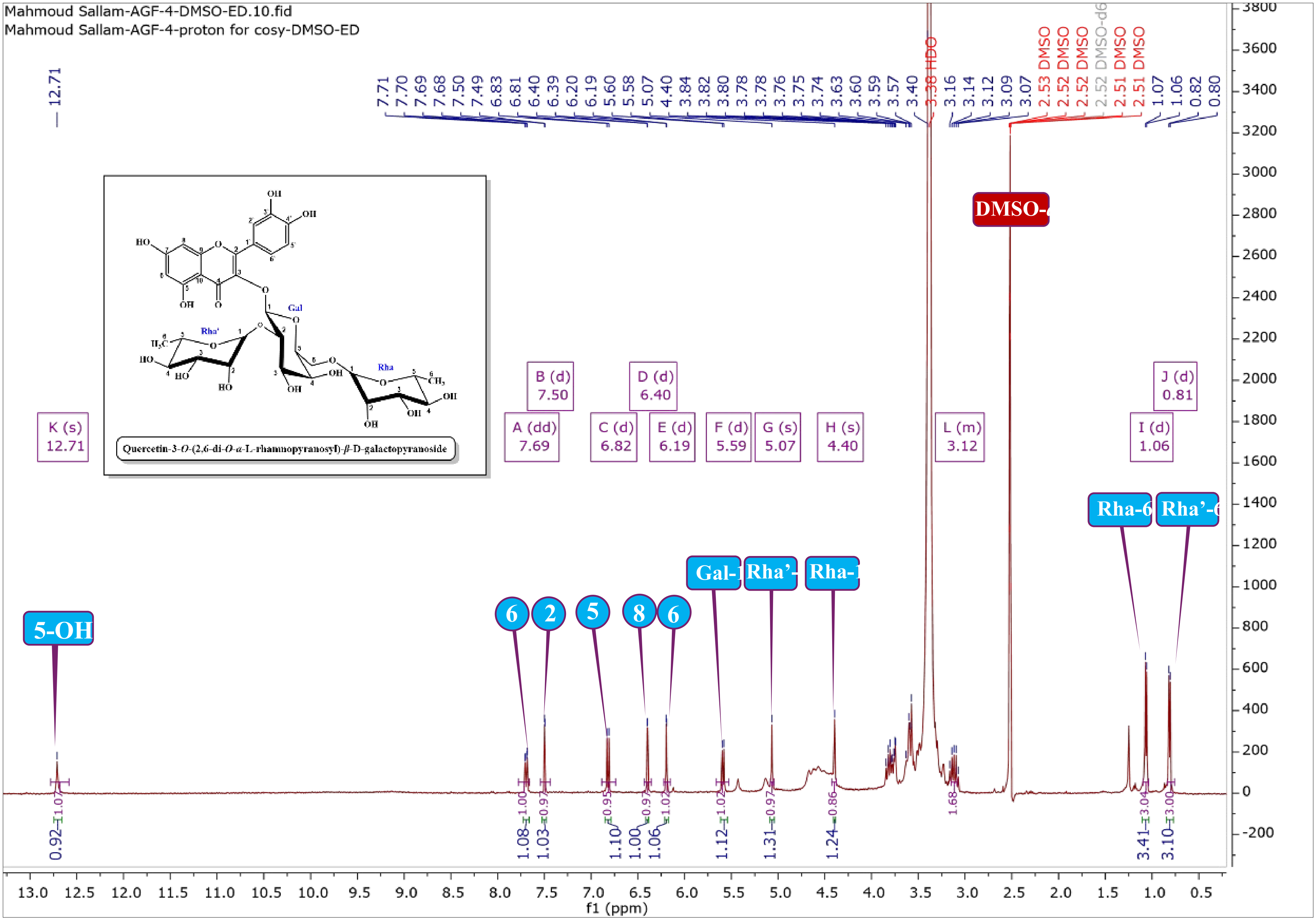

Figure S13: ^{1}H NMR spectrum of compound **2** (DMSO-d_6, 400 MHz).

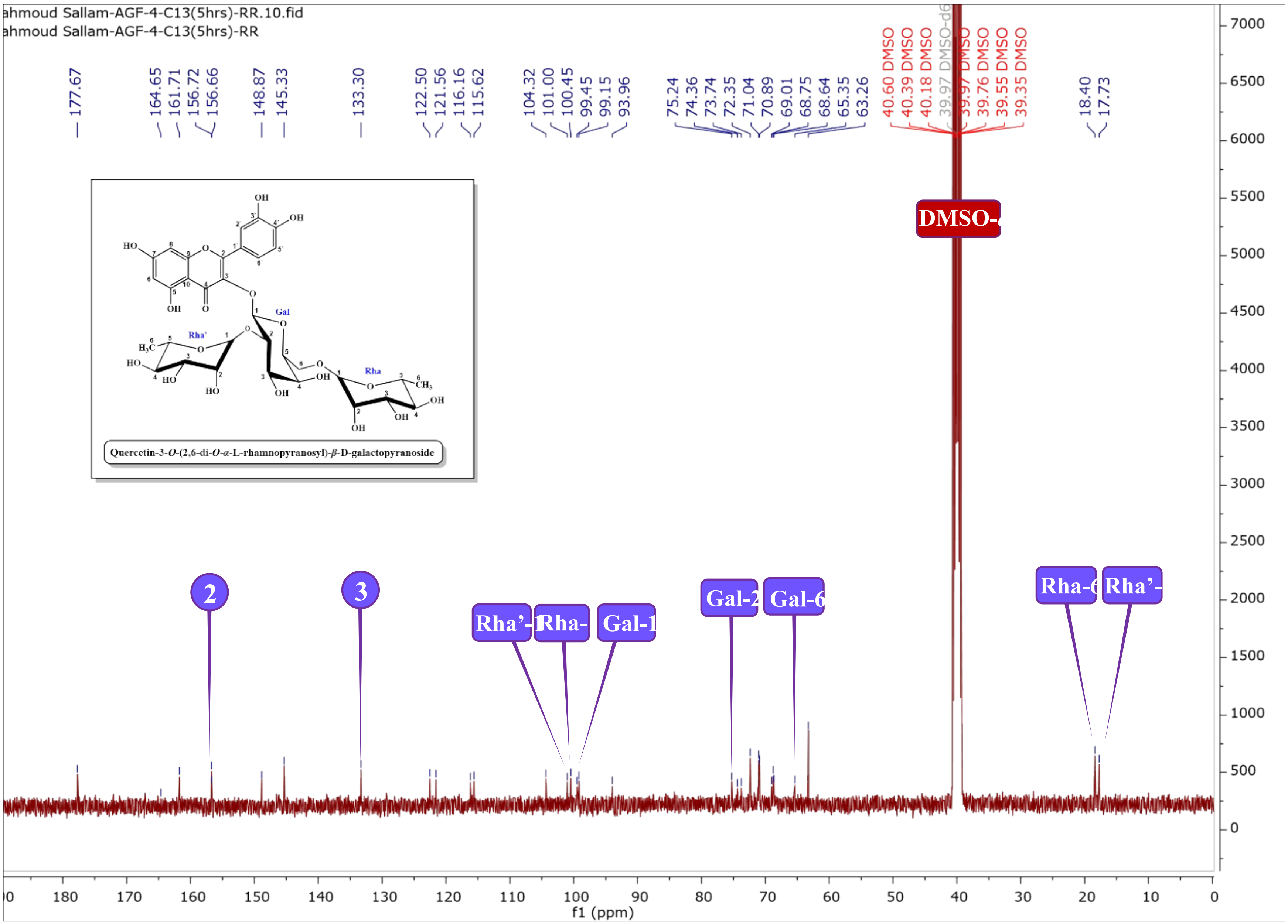

Figure S14: ^{13}C NMR spectrum of compound **2** (DMSO-d_6, 100 MHz).

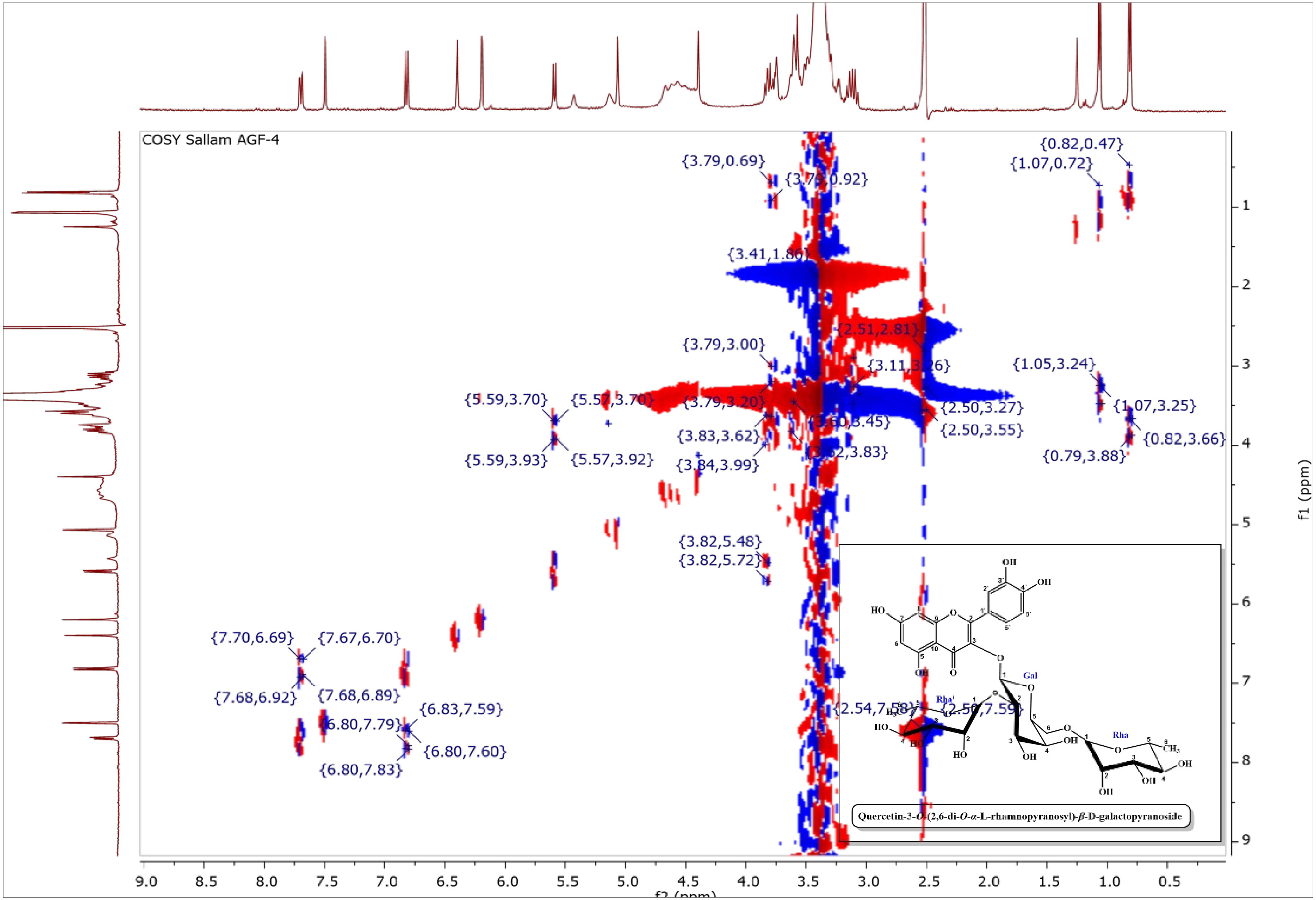

Figure S15: COSY spectrum of compound **2**.

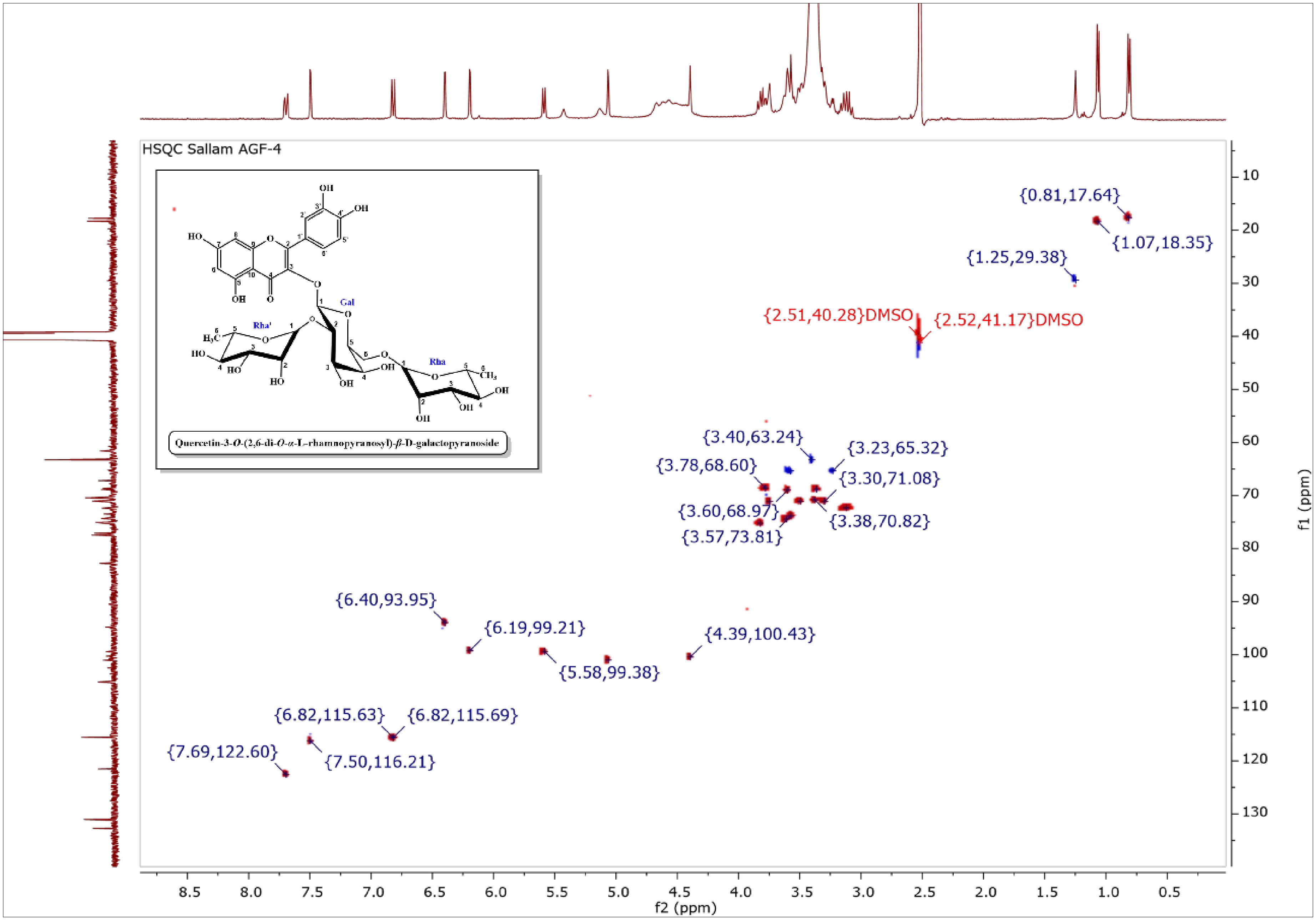

Figure S16: HSQC spectrum of compound **2**.

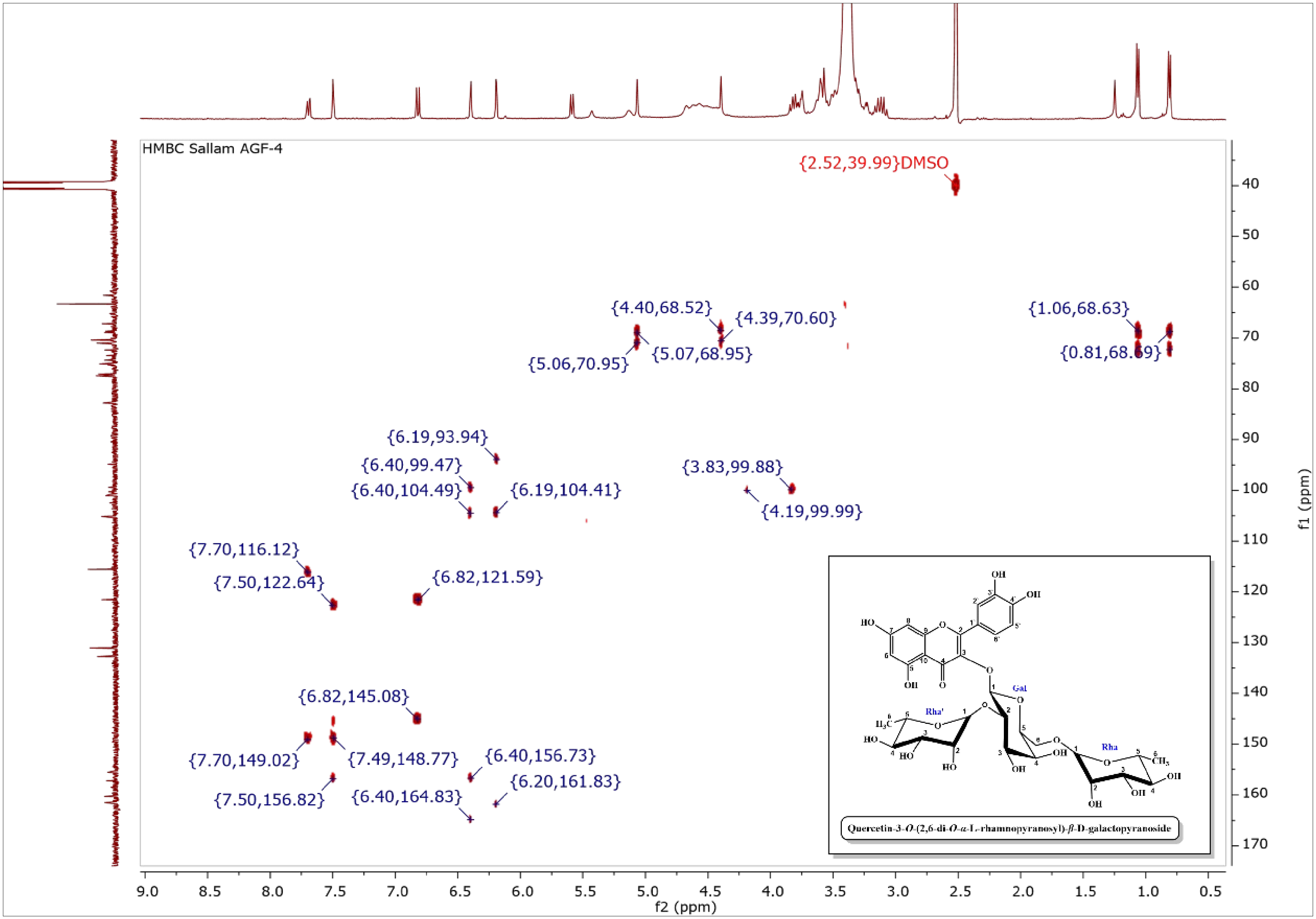

Figure S17: HMBC spectrum of compound **2**.

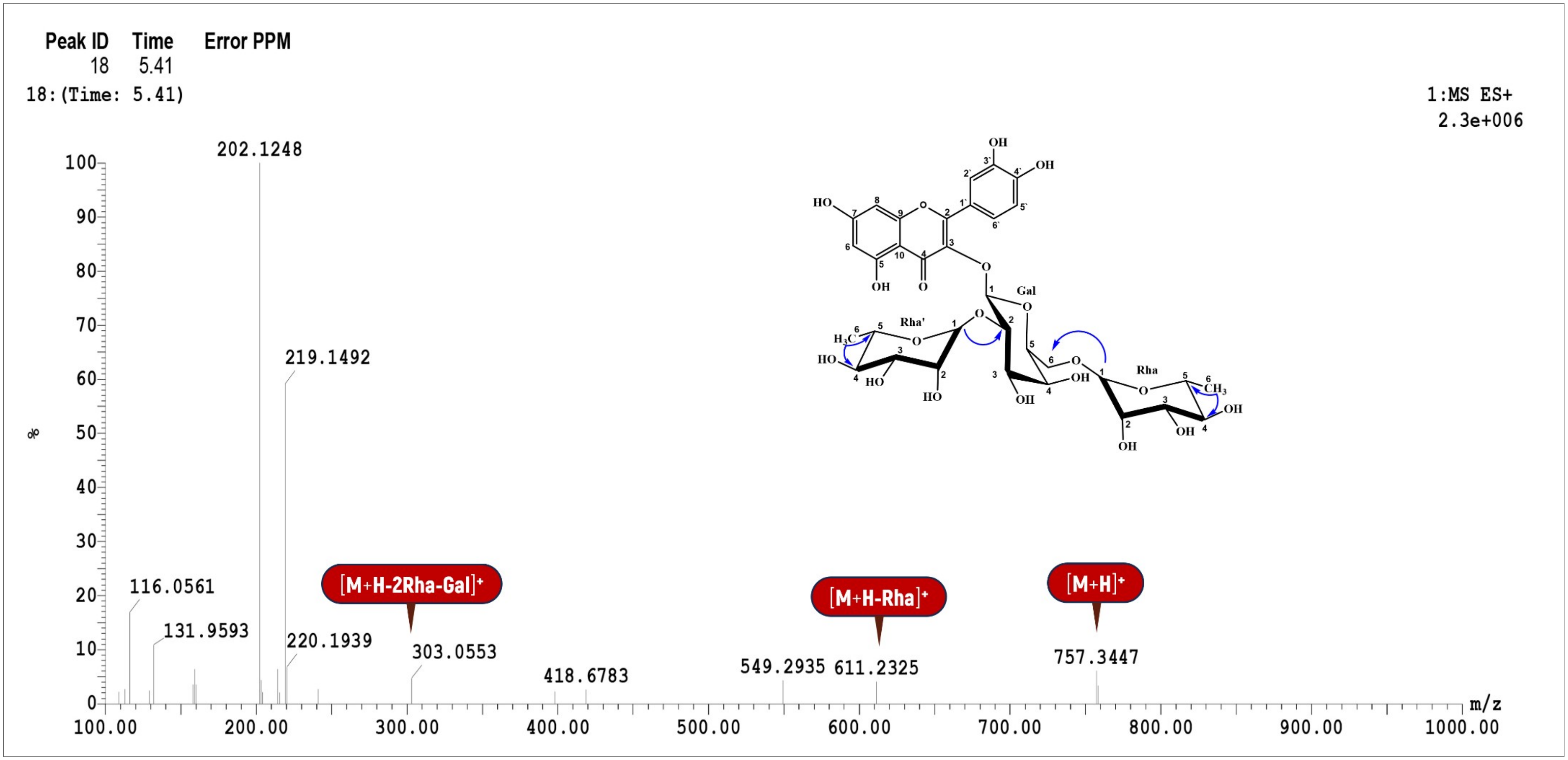

Figure S18: Positive ESI–MS spectrum of compound **2**.

Compound (3)

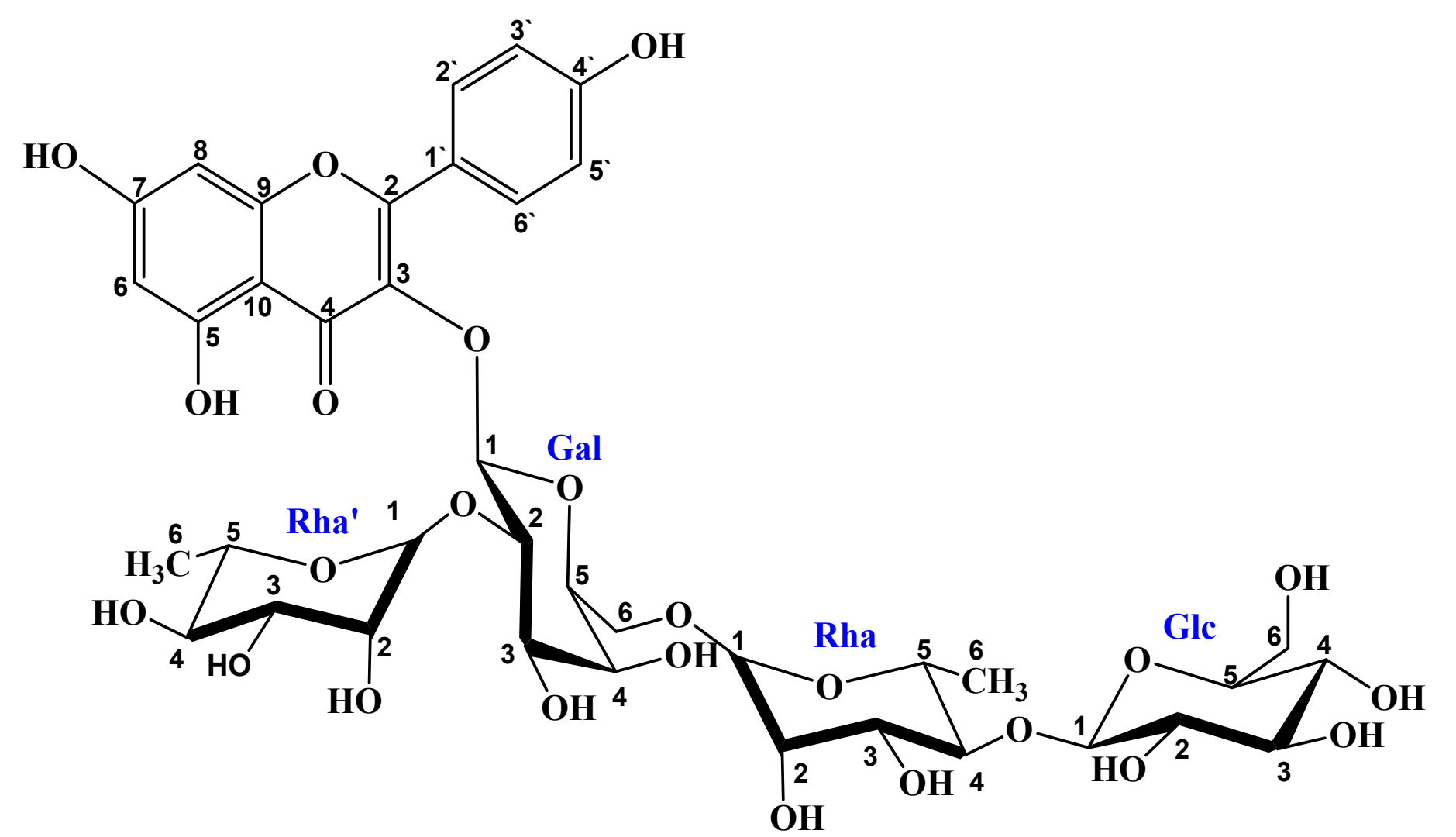

Kaempferol-3-*O*-*β*-D-glucopyranosyl-(1→4)-*α*-L-rhamnopyranosyl-(1→6)-*O*-[*α*-L-rhamnopyranosyl-(1→2)]-*β*-D-galactopyranoside

Compound **3**:	Kaempferol-3-*O*-*β*-D-glucopyranosyl-(1→4)-*α*-L-rhamnopyranosyl-(1→6)-*O*-[*α*-L-rhamnopyranosyl-(1→2)]-*β*-D-galactopyranoside
Molecular formula:	$C_{39}H_{50}O_{24}$
Molecular weight:	902
Melting point (°C):	186–190.
+ve ESIMS *m/z*:	903 $[M+H]^+$
	757 $[M+H\text{-}Rha]^+$
	595 $[M+H\text{-}Rha\text{-}Glc]^+$
	449 $[M+H\text{-}2Rha\text{-}Glc]^+$
	287 $[M+H\text{-}2Rha\text{-}Glc\text{-}Gal]^+$
-ve ESIMS *m/z*:	901 $[M-H]^-$
	755 $[M\text{-}H\text{-}Rha]^-$

Table S4: 1D and 2D NMR spectral data of compound **3** (400 MHz for ^{1}H, 100 MHz for ^{13}C, DMSO-d_6).

Position	^{1}H (J in Hz)	^{13}C	COSY	ROESY	HMBC
**Aglycone**					
2	-	155.11	-	-	-
3	-	132.26	-	-	-
4	-	176.49	-	-	-
5	-	161.06	-	-	-
6	5.98, s	100.30	H-8		C-5, C-8, C-10
7	-	163.50	-	-	-
8	6.18, s	94.39	H-6		C-6, C-9, C-10
9	-	156.73	-	-	-
10	-	102.03	-	-	-
1′	-	121.07	-	-	-
2′	8.00, d, 8.8	130.60	H-3′	H-6′	C-2, C-4′, C-6′
3′	6.83, d, 8.8	115.11	H-2′	H-5′	C-1′, C-4′, C-5\`
4′	-	159.87	-	-	-
5′	6.83, d, 8.8	115.11	H-6′	H-3′	C-1′, C-3′, C-4′
6′	8.00, d, 8.8	130.60	H-5′	H-2′	C-2, C-2′, C-4′
5-OH	12.63, brs	-	-	-	-
**Gal**					
1	5.56, d, 7.7	98.96	H-2	H-3, H-4, H-5	-
2	3.78, m	75.04	H-3	-	C-1
3	3.61, m	74.59	H-2	-	-
4	3.58, m	68.42	H-5	-	-
5	3.54, m	73.05	H-4, H-6	-	-
6a<br>6b	3.59, m<br>3.18, m	64.77	H-6b,<br>H-6a	-	-
**Rha**					
1	4.38, s	99.81	H-2	H-6a of Gal,<br>H-6b of Gal, H-5	C-6 of Gal,<br>C-3, C-5
2	3.46, m	69.98	H-1	-	-
3	3.54, m	70.57	-	-	C-4
4	3.37, m	82.36	H-5	-	C-5

5	3.47, m	66.73	H-4, H-6	-	C-4
6	1.15, d, 6.0	17.81	H-5	-	C-4, C-5
**Glc**					
1	4.32, d, 7.8	104.70	H-2	H-4 of Rha, H-3, H-5	C-4 of Rha
2	2.98, t, 8.4	74.85	H-1, H-3	-	C-1, C-3
3	3.15, m	76.64	H-2	-	C-4
4	3.05	70.71	H-5	-	C-5
5	3.07, m	77.00	H-4, H-6	-	-
6a<br>6b	3.65, brd, 10.8<br>3.43, m	61.13	H-6b,<br>H-6a	-	-
**Rha′**					
1	5.04, s	100.58	H-2	H-2 of Gal	C-2 of Gal, C-2, C-5
2	3.74, m	70.57	H-1	-	-
3	3.51, m	70.68	H-4	-	-
4	3.14, m	71.91	H-3, H-5	-	C-3, C-5
5	3.80, m	68.19	H-4, H-6	-	-
6	0.78, d, 6.1	17.30	H-5	-	C-3, C-5

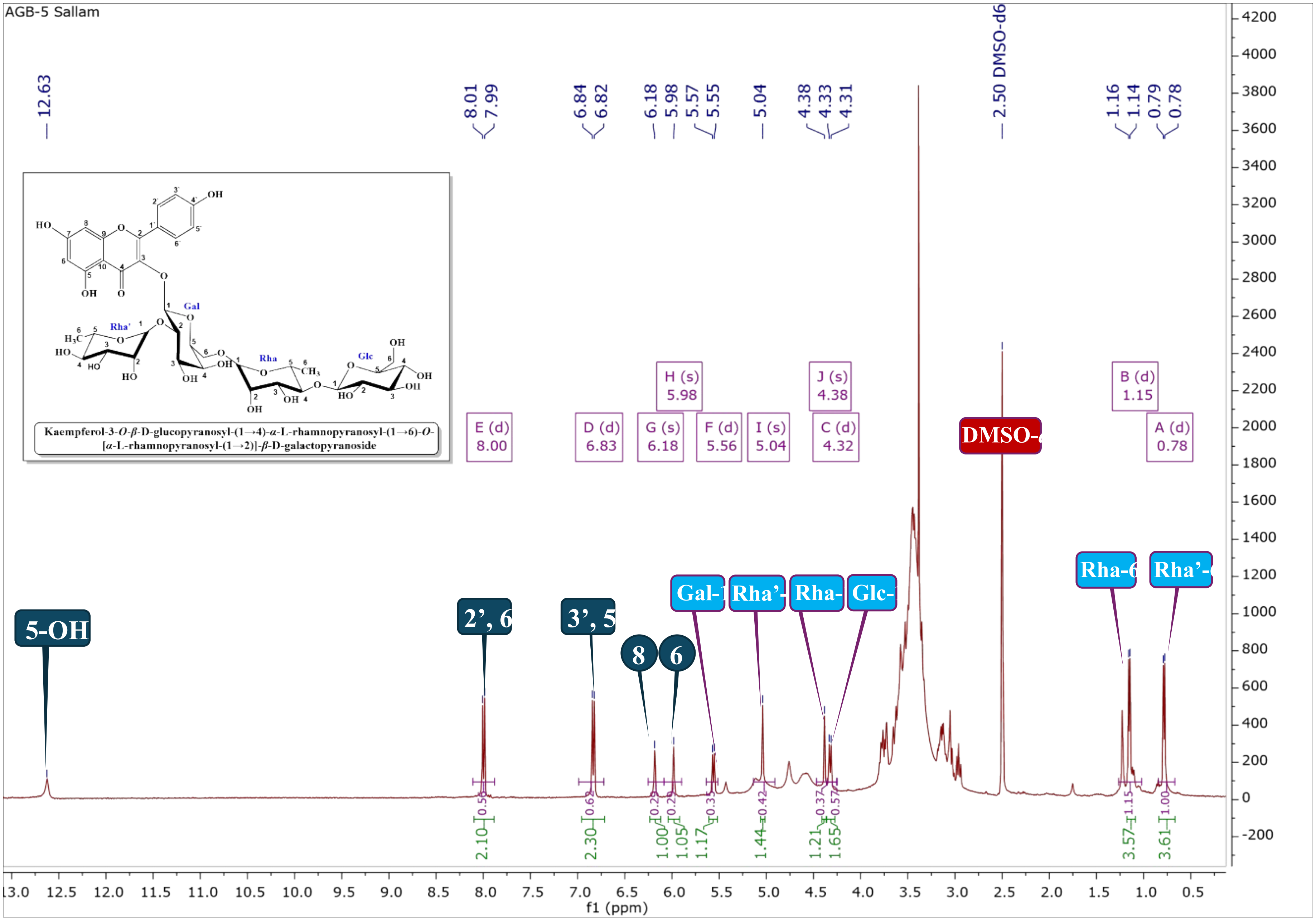

Figure S19: ^{1}H NMR spectrum of compound **3** (DMSO-d_6, 400 MHz).

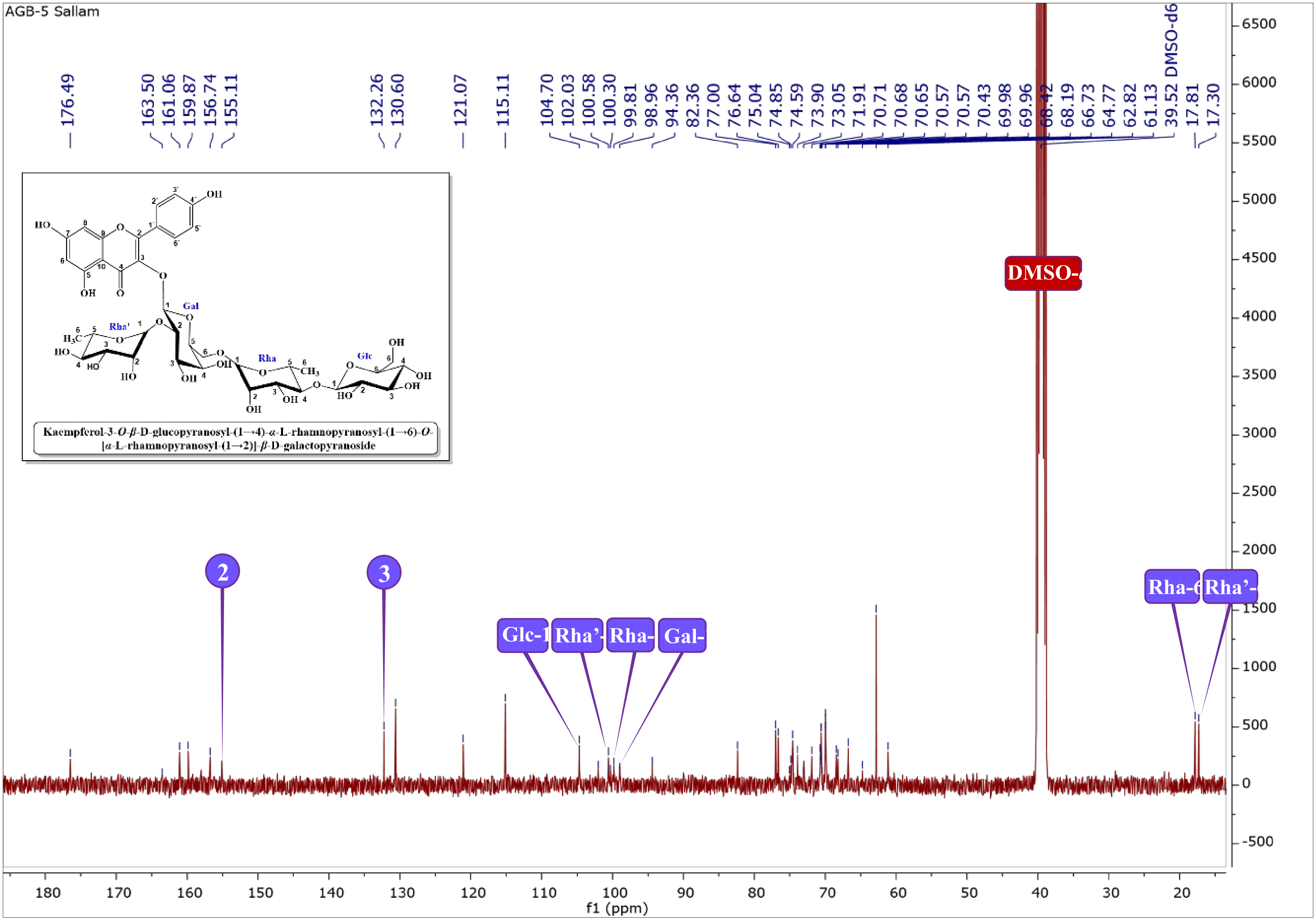

Figure S20: ^{13}C NMR spectrum of compound **3** (DMSO-d_6, 100 MHz).

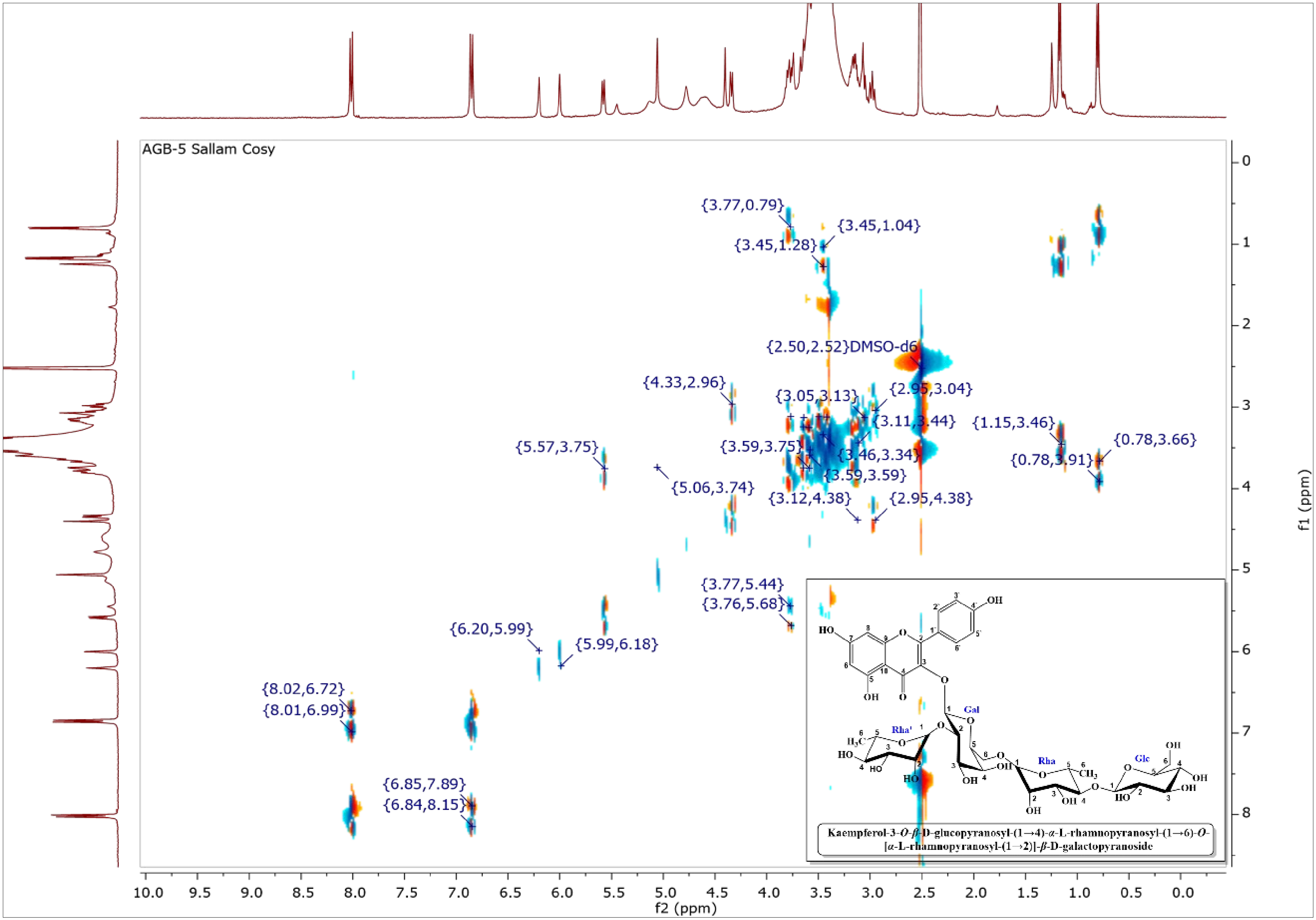

Figure S21: COSY spectrum of compound **3**.

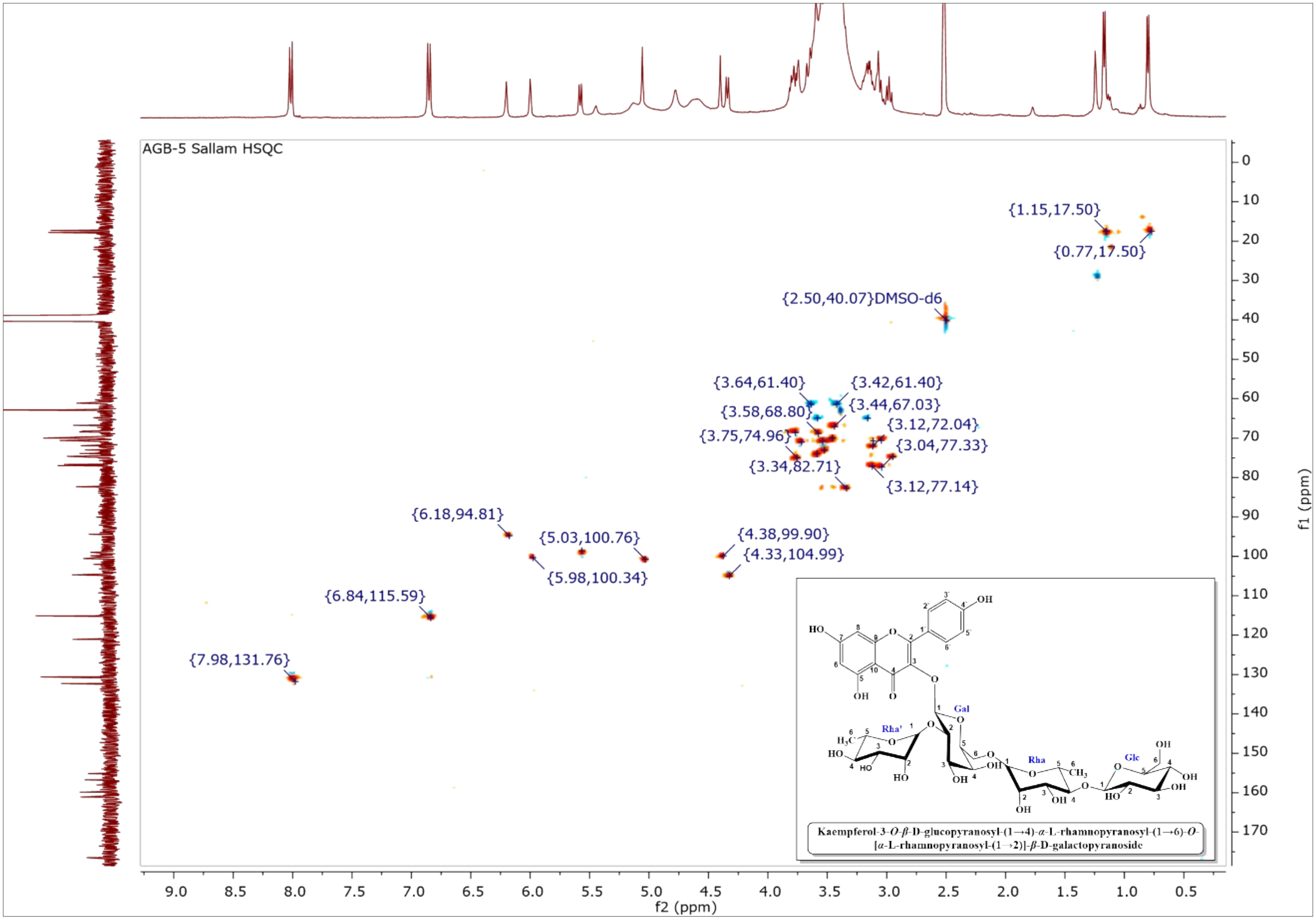

Figure S22: HSQC spectrum of compound **3**.

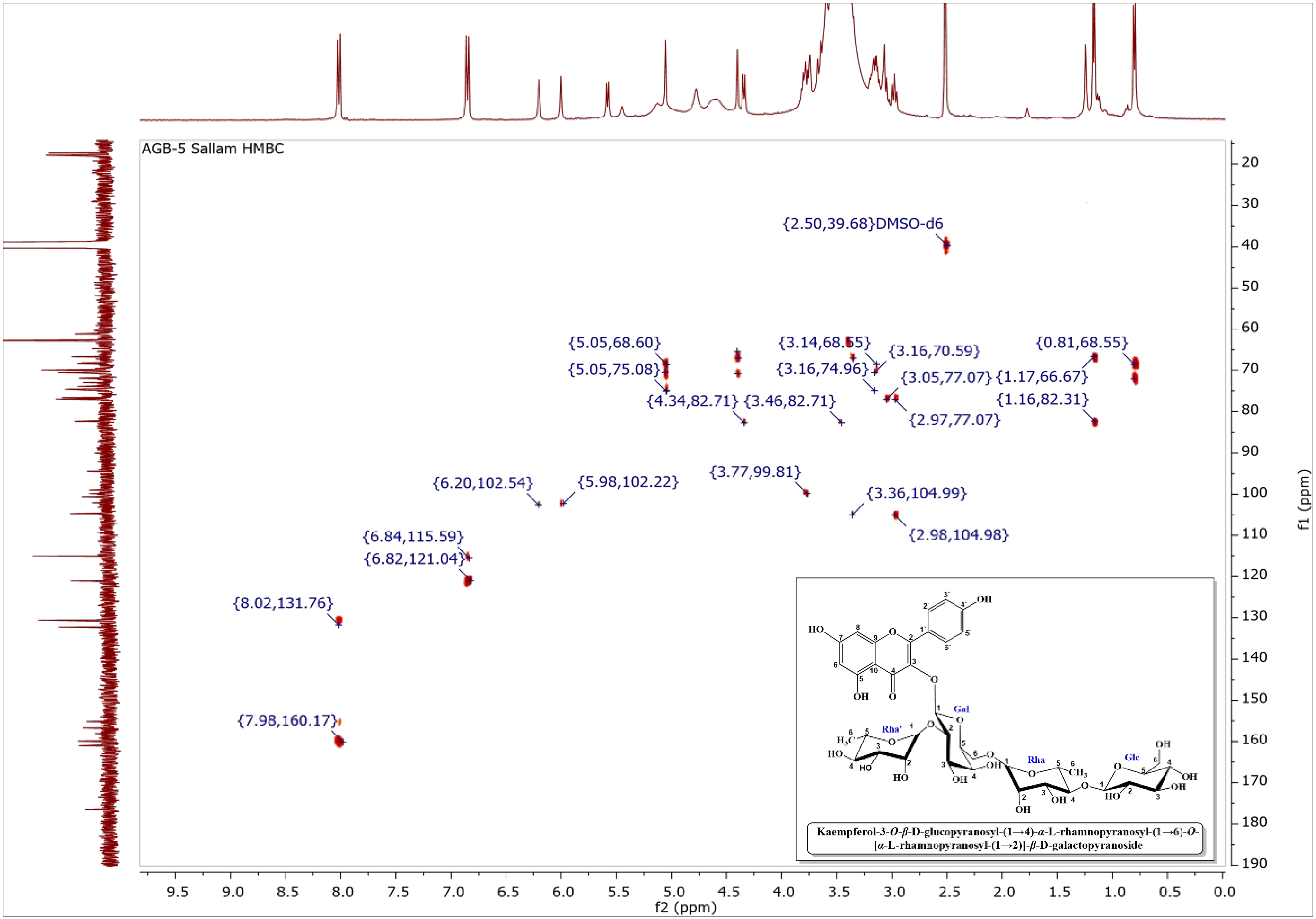

Figure S23: HMBC spectrum of compound **3**.

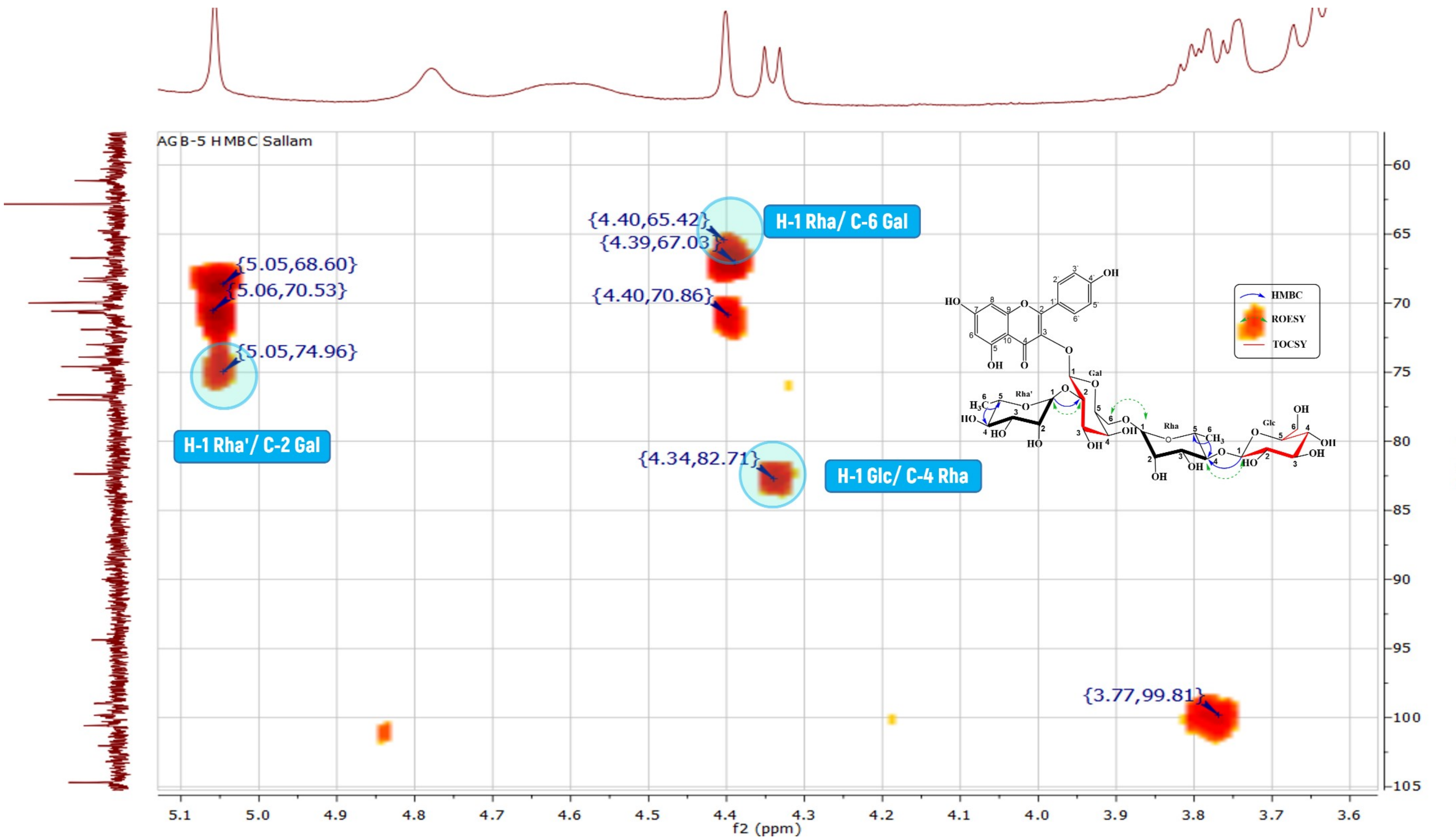

Figure S24: Expanded HMBC spectrum of compound **3**.

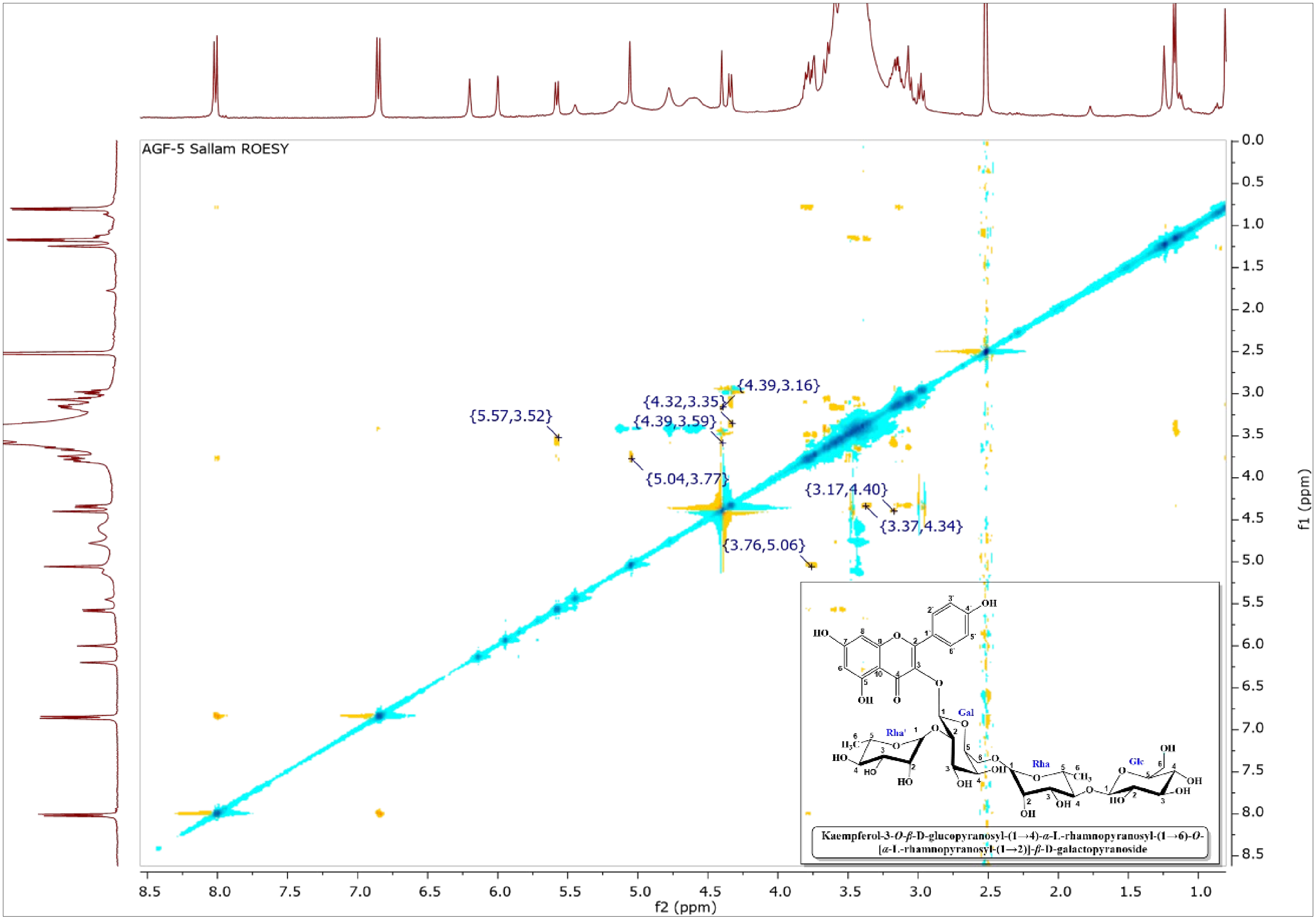

Figure S25: ROESY spectrum of compound **3**.

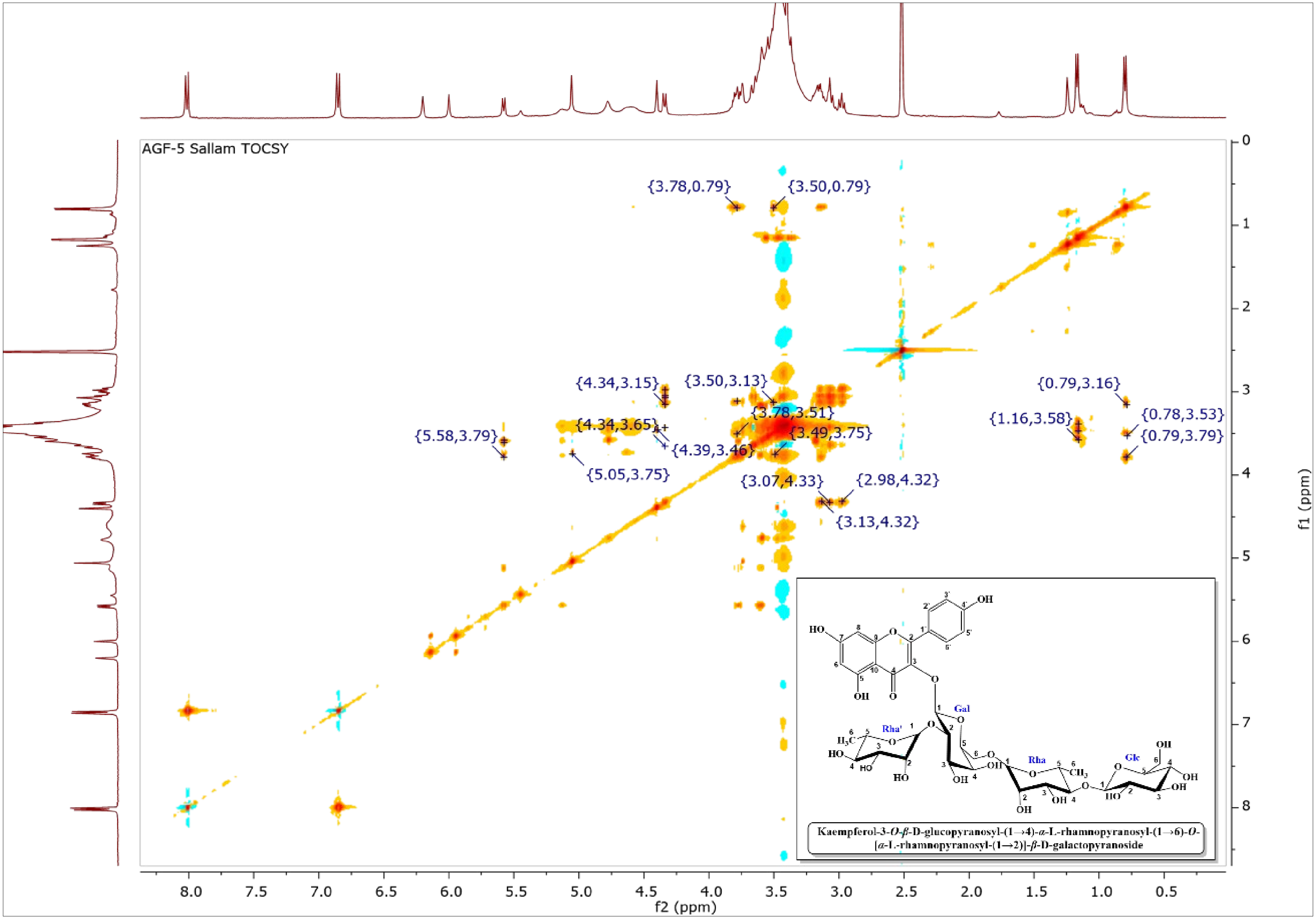

Figure S26: TOCSY spectrum of compound **3**.

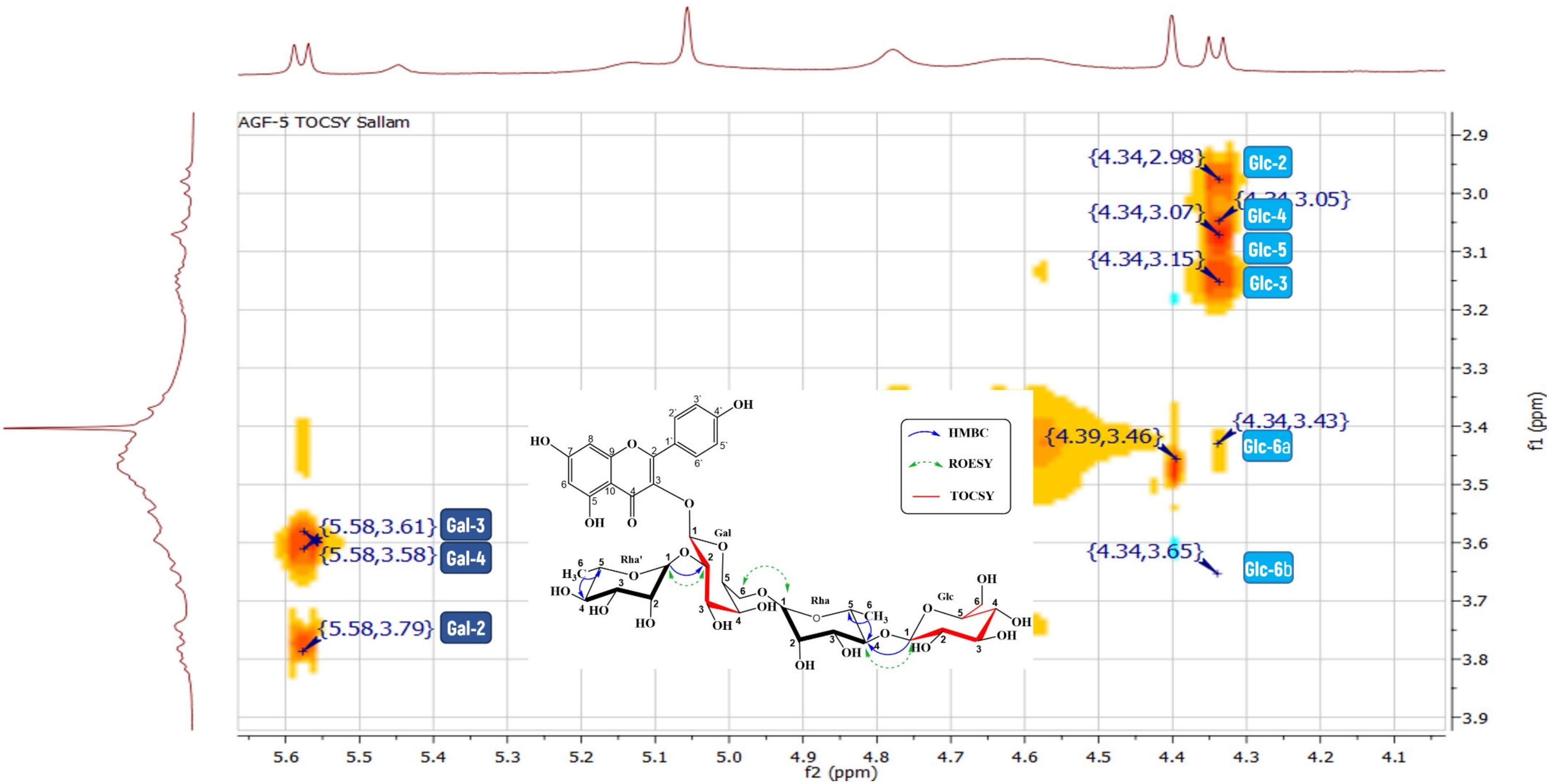

Figure S27: TOCSY spectrum of compound **3**.

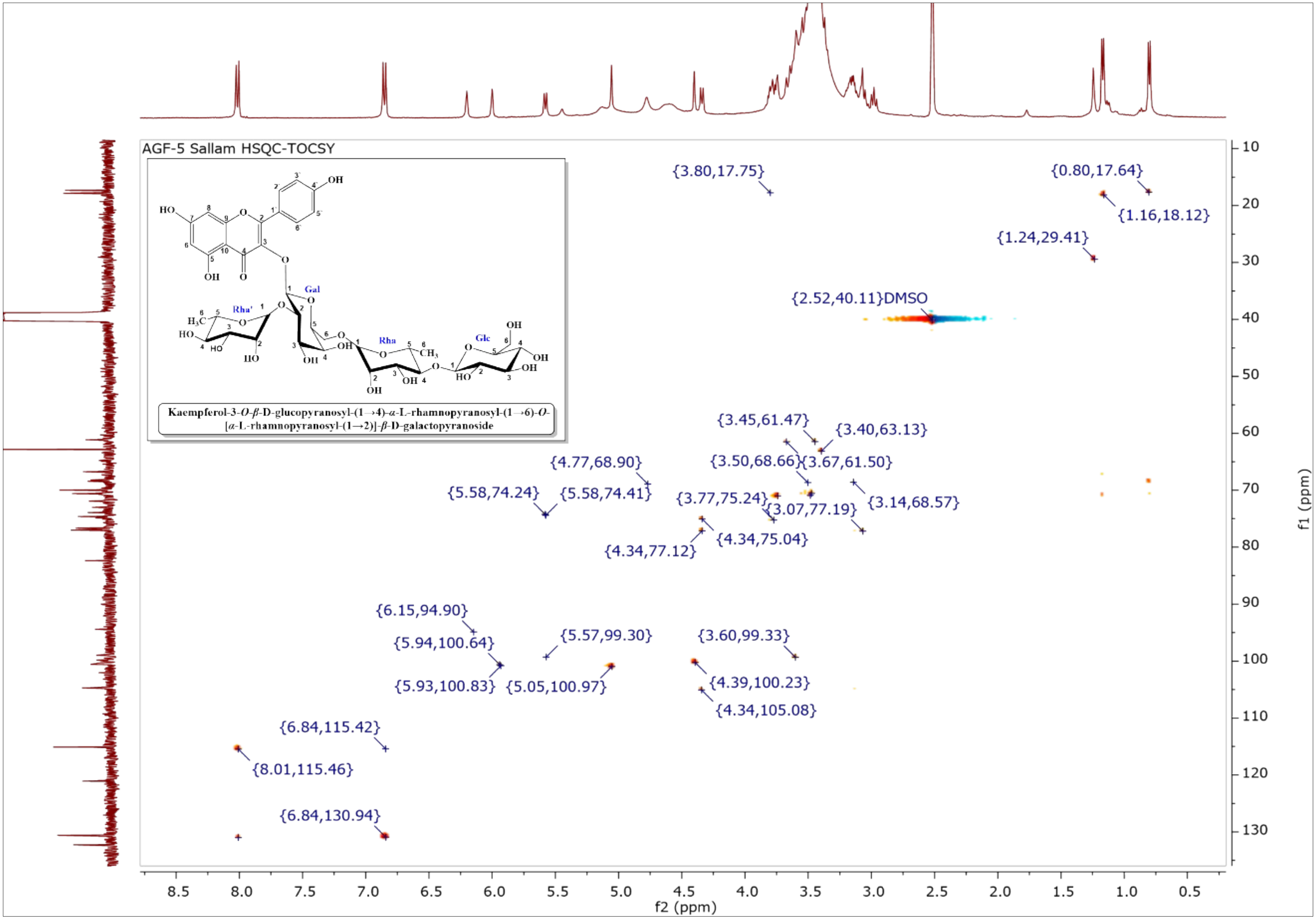

Figure S28: HSQC–TOCSY spectrum of compound **3**.

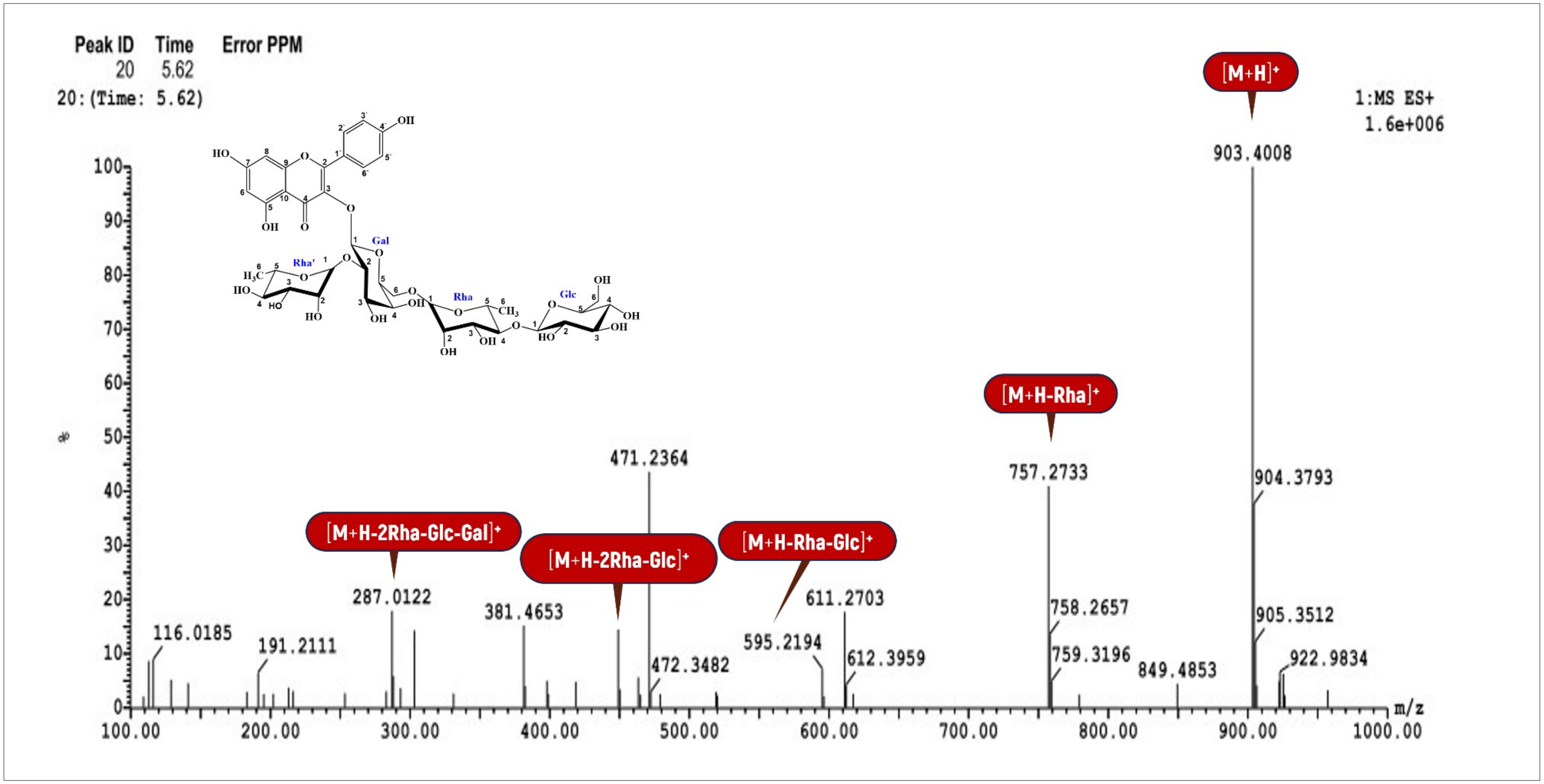

Figure S29: Positive ESI–MS spectrum of compound **3**.

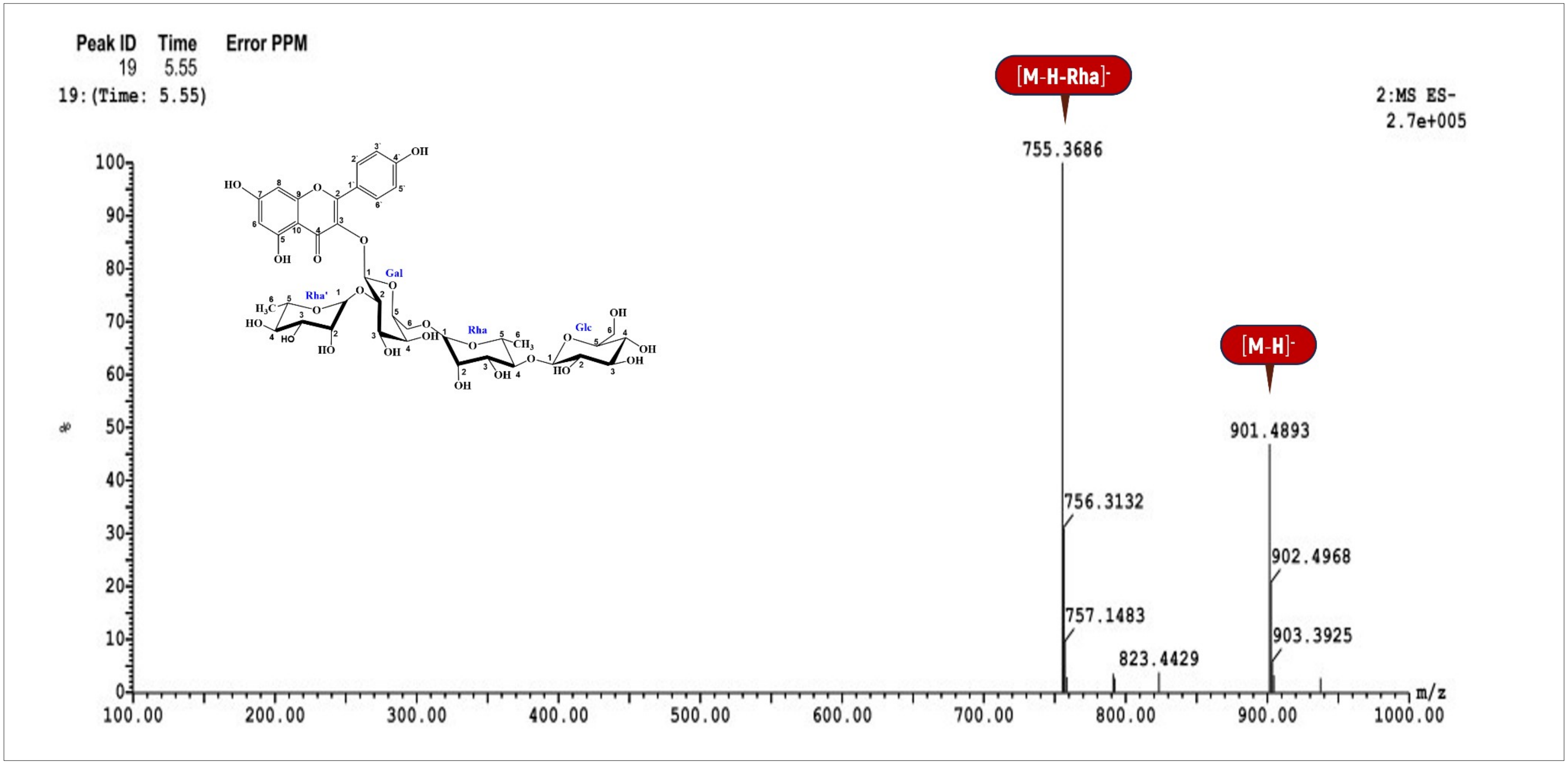

Figure S30: Negative ESI–MS spectrum of compound **3**.

Compound (4)

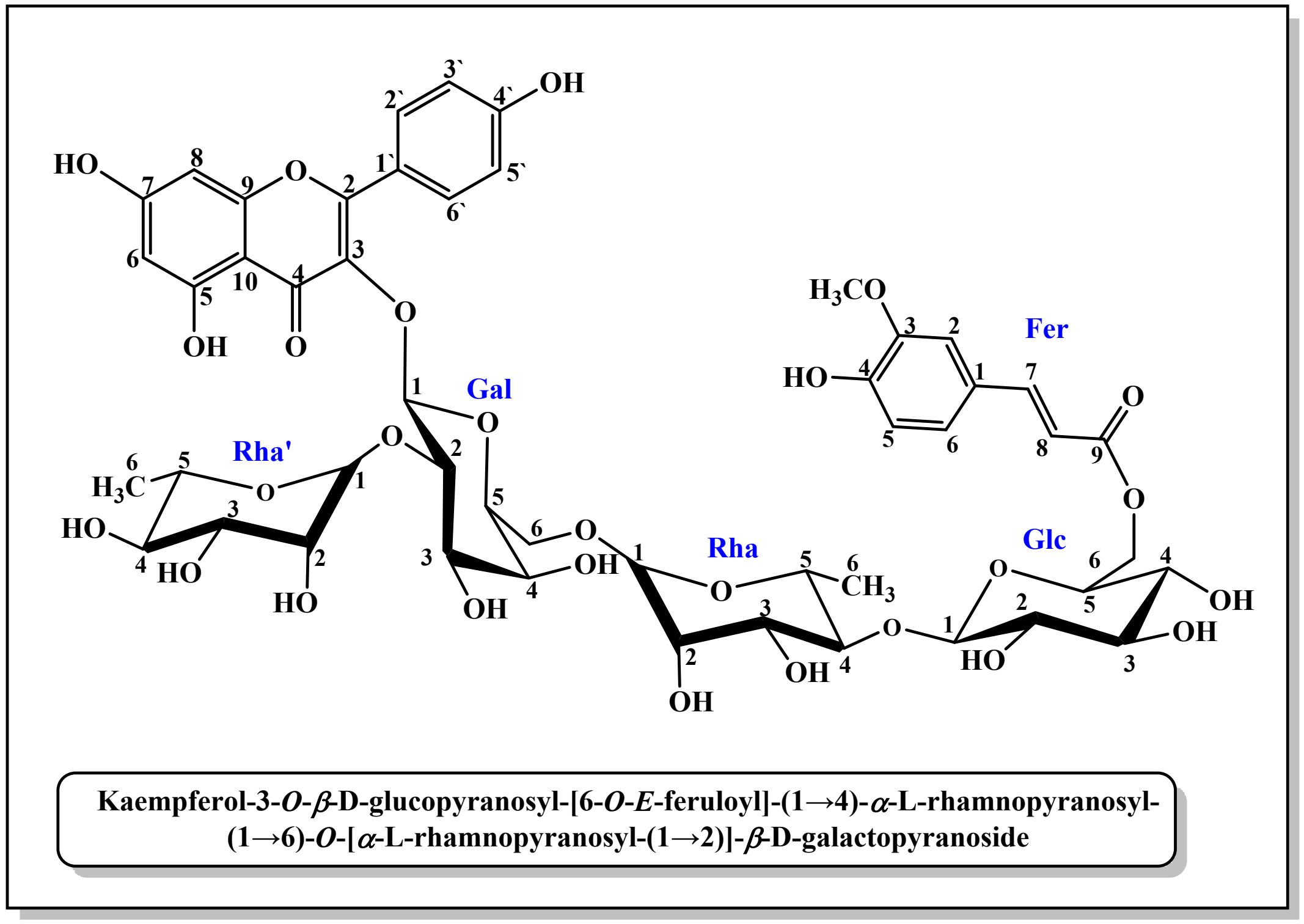

Kaempferol-3-*O*-*β*-D-glucopyranosyl-[6-*O*-*E*-feruloyl]-(1→4)-*α*-L-rhamnopyranosyl-(1→6)-*O*-[*α*-L-rhamnopyranosyl-(1→2)]-*β*-D-galactopyranoside

Compound **4**:	Kaempferol-3-*O*-*β*-D-glucopyranosyl-[6-*O*-*E*-feruloyl]-(1→4)-*α*-L-rhamnopyranosyl-(1→6)-*O*-[*α*-L-rhamnopyranosyl-(1→2)]-*β*-D-galactopyranoside.
Molecular formula:	$C_{49}H_{58}O_{27}$
Molecular weight:	1078
Melting point (°C):	198–202.
+ve ESIMS *m/z*:	1079 $[M+H]^+$
	933 $[M+H\text{-}Rha]^+$
	449 $[M+H\text{-}2Rha\text{-}Glc\text{-}Fer]^+$
	287 $[\text{Aglycone}]^+$

Table S5: 1D and 2D NMR spectral data of compound **4** (400 MHz for ^{1}H, 100 MHz for ^{13}C, DMSO-d_6).

Position	^{1}H (J in Hz)	$^{13}C^{a}$	ROESY	HMBC
**Aglycone**				
2	-		-	-
3	-		-	-
4	-		-	-
5	-	162.00	-	-
6	6.18, d, 2.1	99.18	-	C-5, C-7, C-10
7	-	164.80	-	-
8	6.40, d, 1.9	94.01		C-6, C-10
9	-	156.27	-	-
10	-	103.76	-	-
1′	-	121.34	-	-
2′	8.03, d, 8.8	131.22	H-6′	C-4′, C-6′
3′	6.84, d, 8.8	115.68	H-5′	C-1′
4′	-	160.37	-	-
5′	6.84, d, 8.8	115.68	H-3′	C-1′
6′	8.03, d, 8.8	131.22	H-2′	C-2′, C-4′
5-OH	12.67, brs	-	-	-
**Gal**				
1	5.55, d, 7.6	99.15	H-3, H-5	-
2	3.78, m	75.85	-	-
3	3.61, m	74.43	-	-
4	3.56, m	68.90	-	-
5′	3.53, m	73.40	-	-
6a<br>6b	3.57, m<br>3.18, m	65.19	H-6b<br>H-6a	-
**Rha**				
1	4.38, s	99.90	H-6a of Gal,<br>H-6b of Gal,<br>H-2	C-3, C-5
2	3.44, m	70.30	-	-
3	3.53, m	71.03	-	-
4	3.33[b]	83.22	H-6	-
5	3.45, m	66.84	H-6	-
6	1.09, d, 6.0	18.08	H-4, H-5	C-4, 5

**Glc**				
1	4.36, d, 8.8	105.17	H-4 of Rha; H-3, H-5	-
2	3.02, m	74.16	-	-
3	3.19, m	76.75	-	-
4	3.11	70.43	-	-
5	3.41[b]	74.14	-	-
6a<br>6b	4.21, m<br>4.01, m	64.11	-	-
**Rha′**				
1	5.04, s	100.61	H-2 of Gal	C-3, C-5
2	3.73, m	70.93	-	-
3	3.50, m	70.78	-	-
4	3.14, m	72.19	H-6	-
5	3.77, m	68.54	H-6	-
6	0.77, d, 6.1	17.55	H-4, H-5	C-4, C-5
**Fer**				
1	-	125.50	-	-
2	7.29, d, 1.6	111.60	H-8, OCH_3	C-3, C-4, C-6, C-7
3	-	147.22	-	-
4	-	149.81	-	-
5	6.78, d, 8.1	115.95	-	C-1, C-3, C-4
6	7.09, dd, 8.1, 1.7	123.59	-	C-4
7	7.52, d, 16.0	145.88	-	C-9
8	6.44, d, 16.0	114.65	-	C-1
9	-	167.0	-	-
OCH_3	3.80, s	55.73	-	C-3

[a] Obtained from HSQC and HMBC spectra.

[b] Masked by water peak.

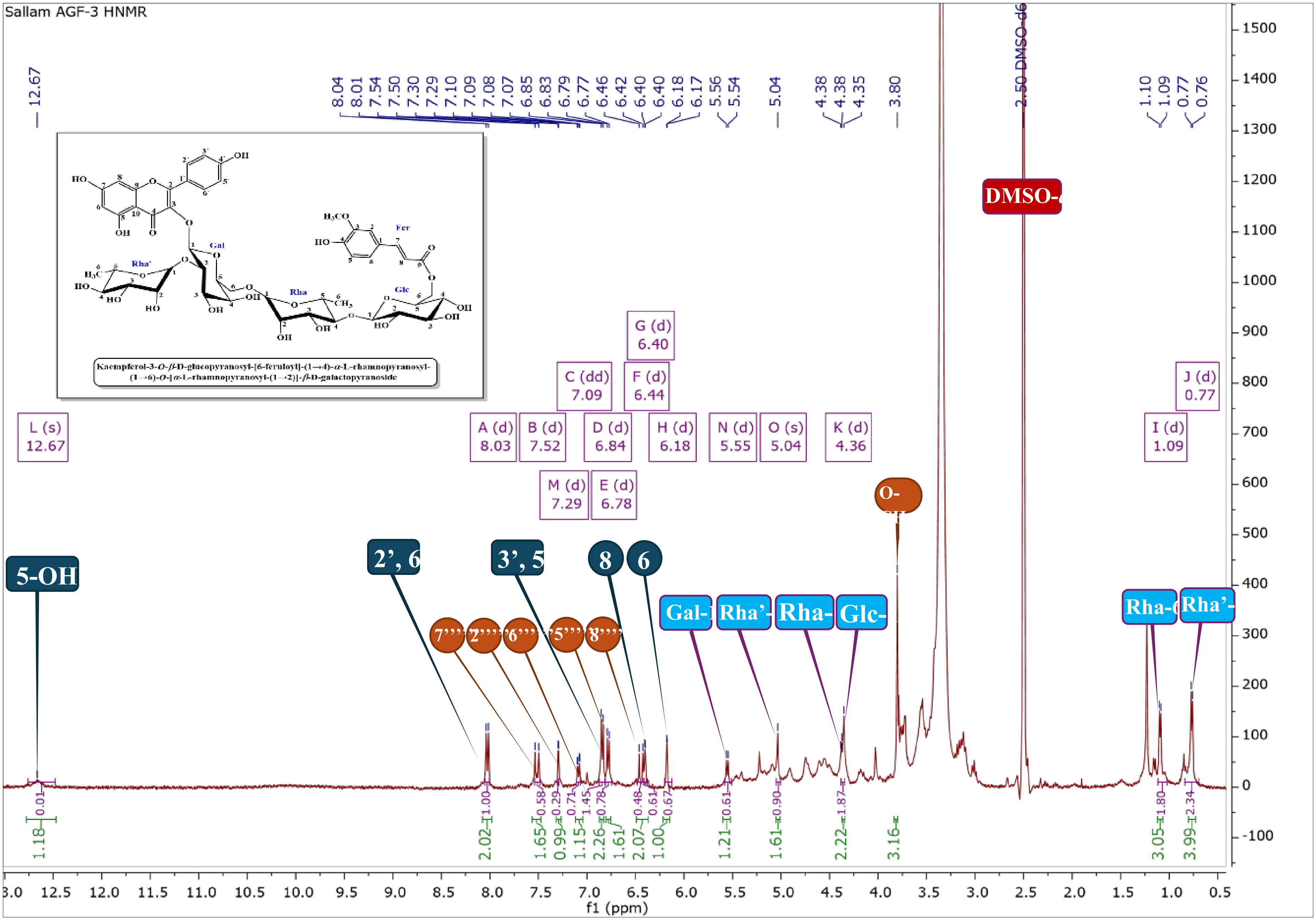

Figure S31: ^{1}H NMR spectrum of compound **4** (DMSO-d_6, 400 MHz).

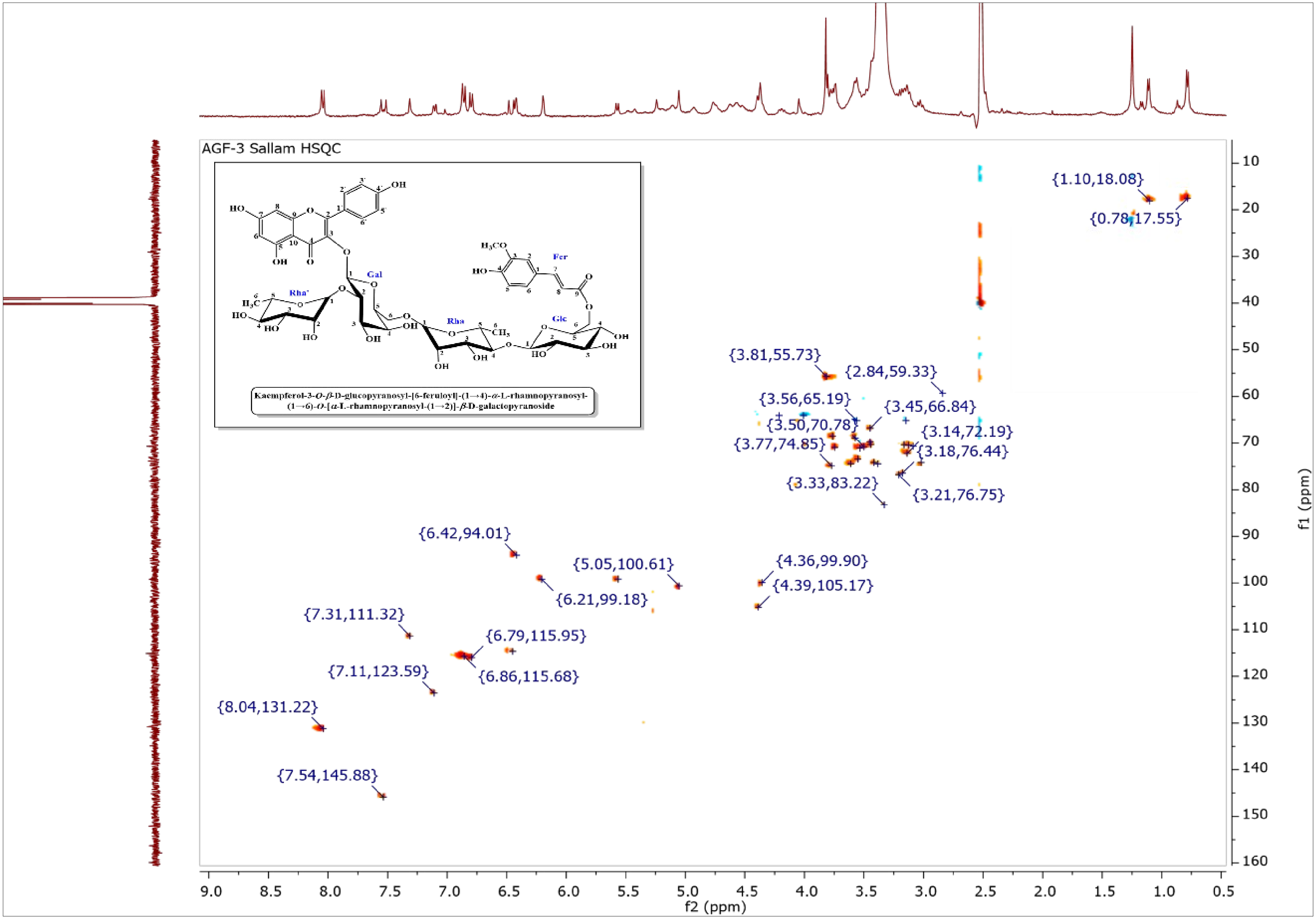

Figure S32: HSQC spectrum of compound **4**.

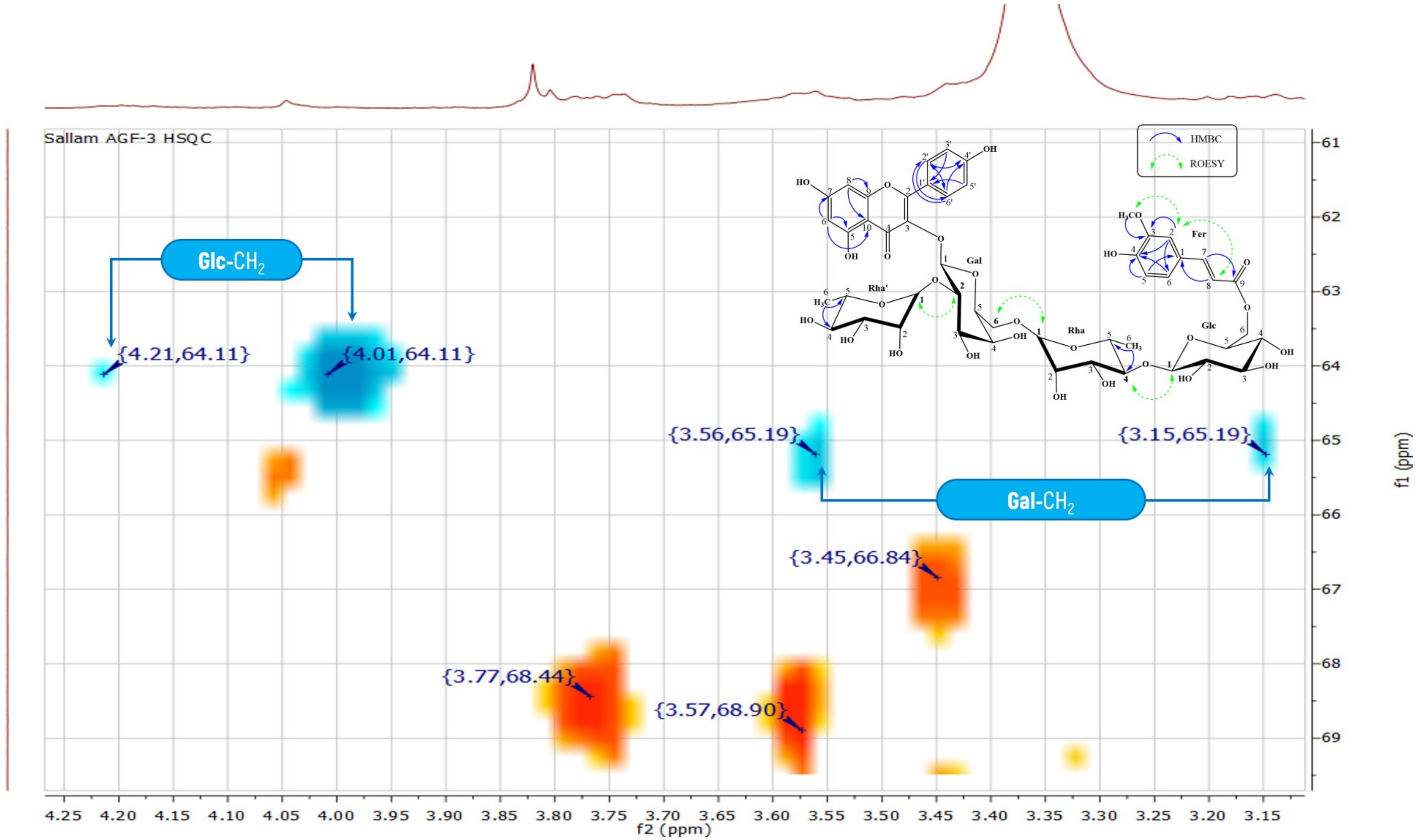

Figure S33: Expanded HSQC spectrum of compound **4**.

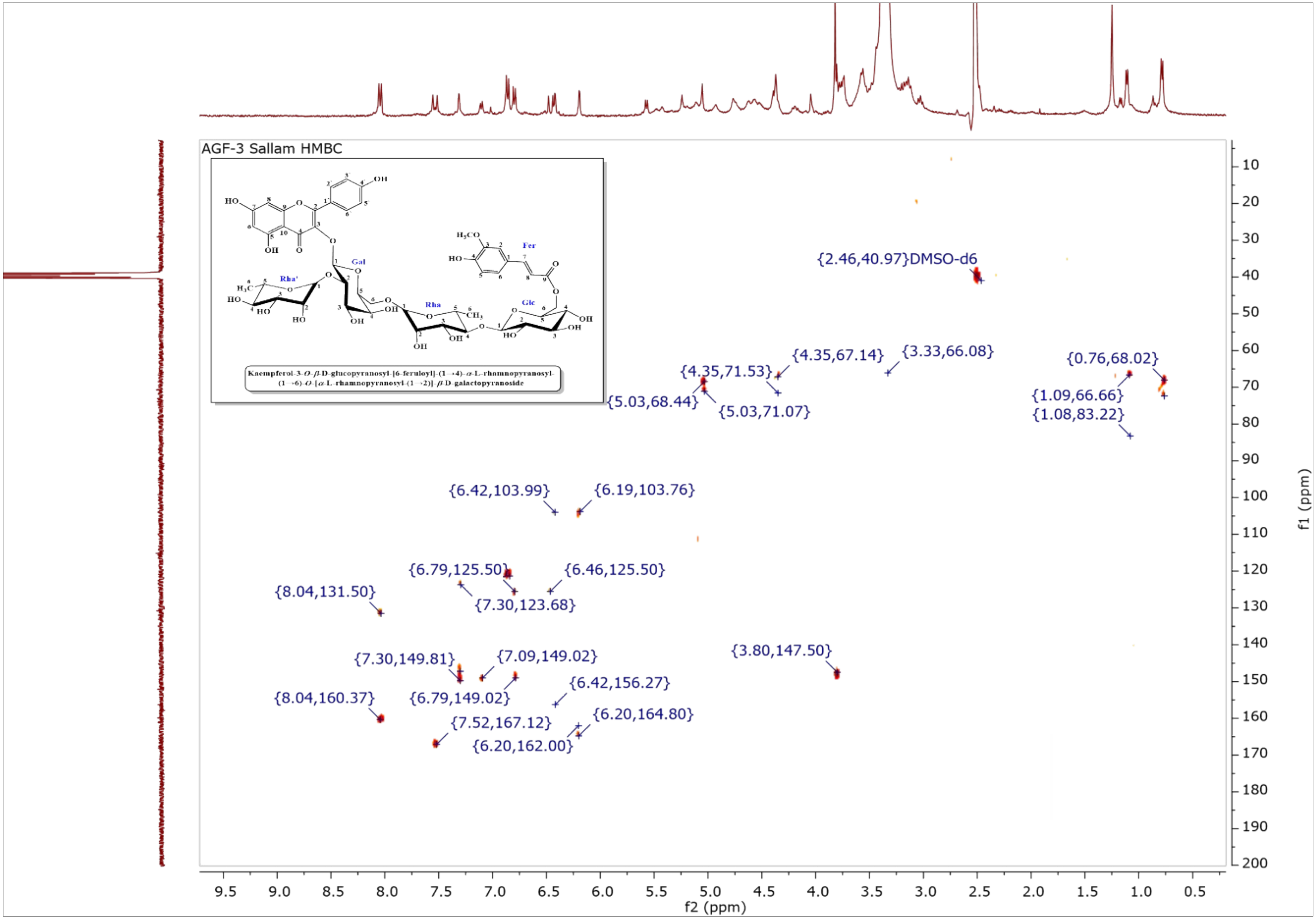

Figure S34: HMBC spectrum of compound **4**.

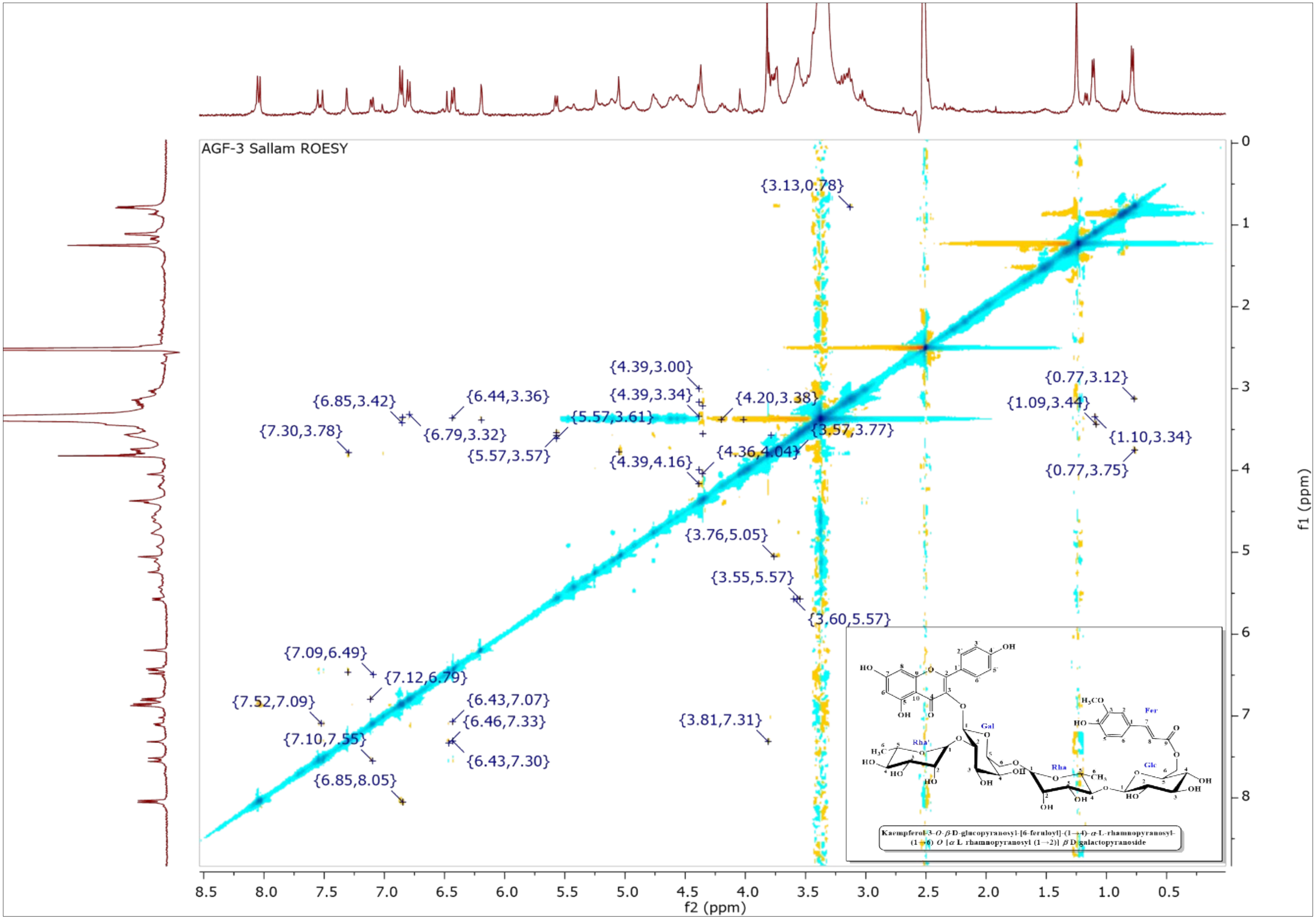

Figure S35: ROESY spectrum of compound **4**.

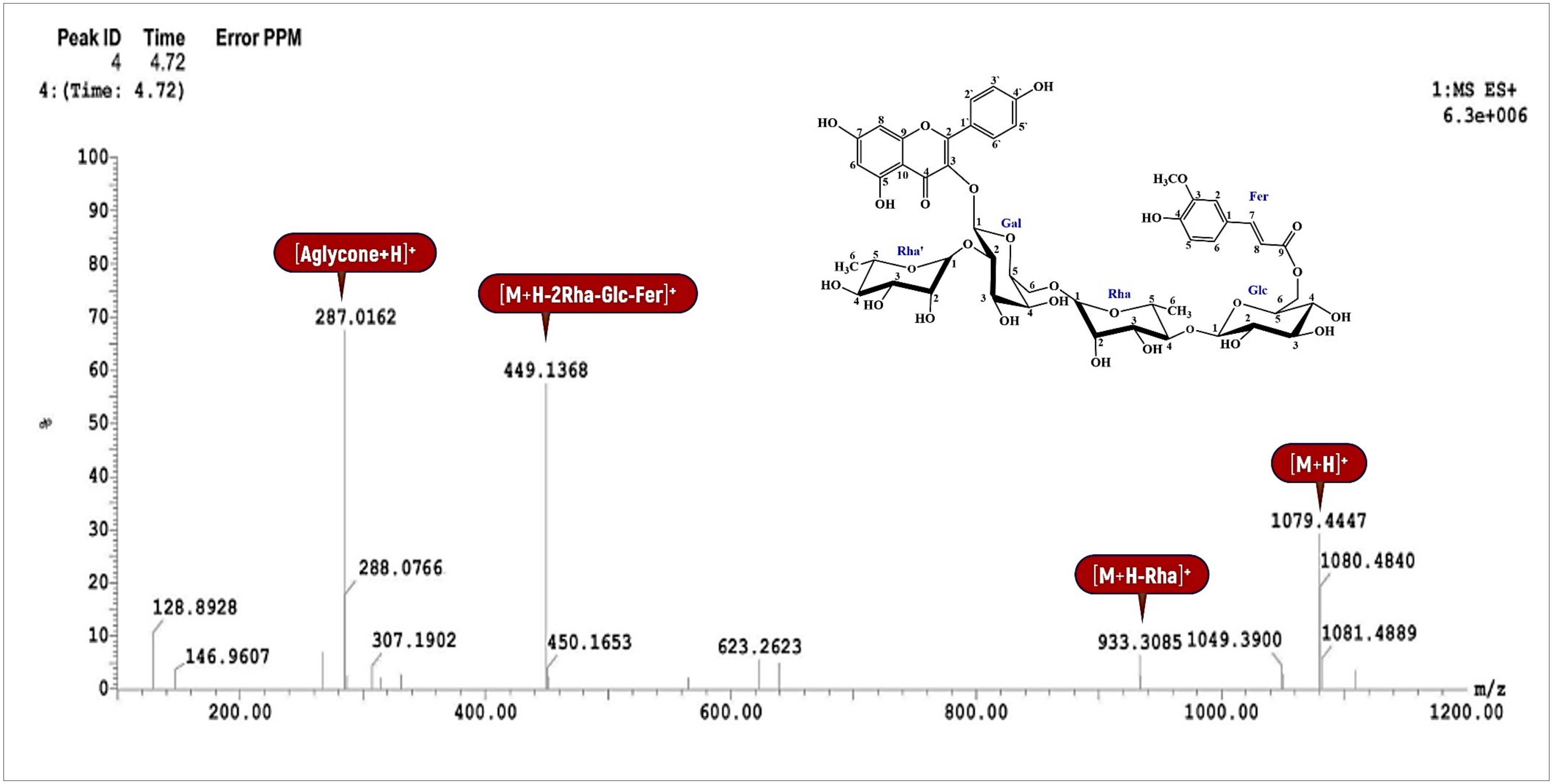

Figure S36: Positive ESI–MS of compound **4**.

Compound (5)

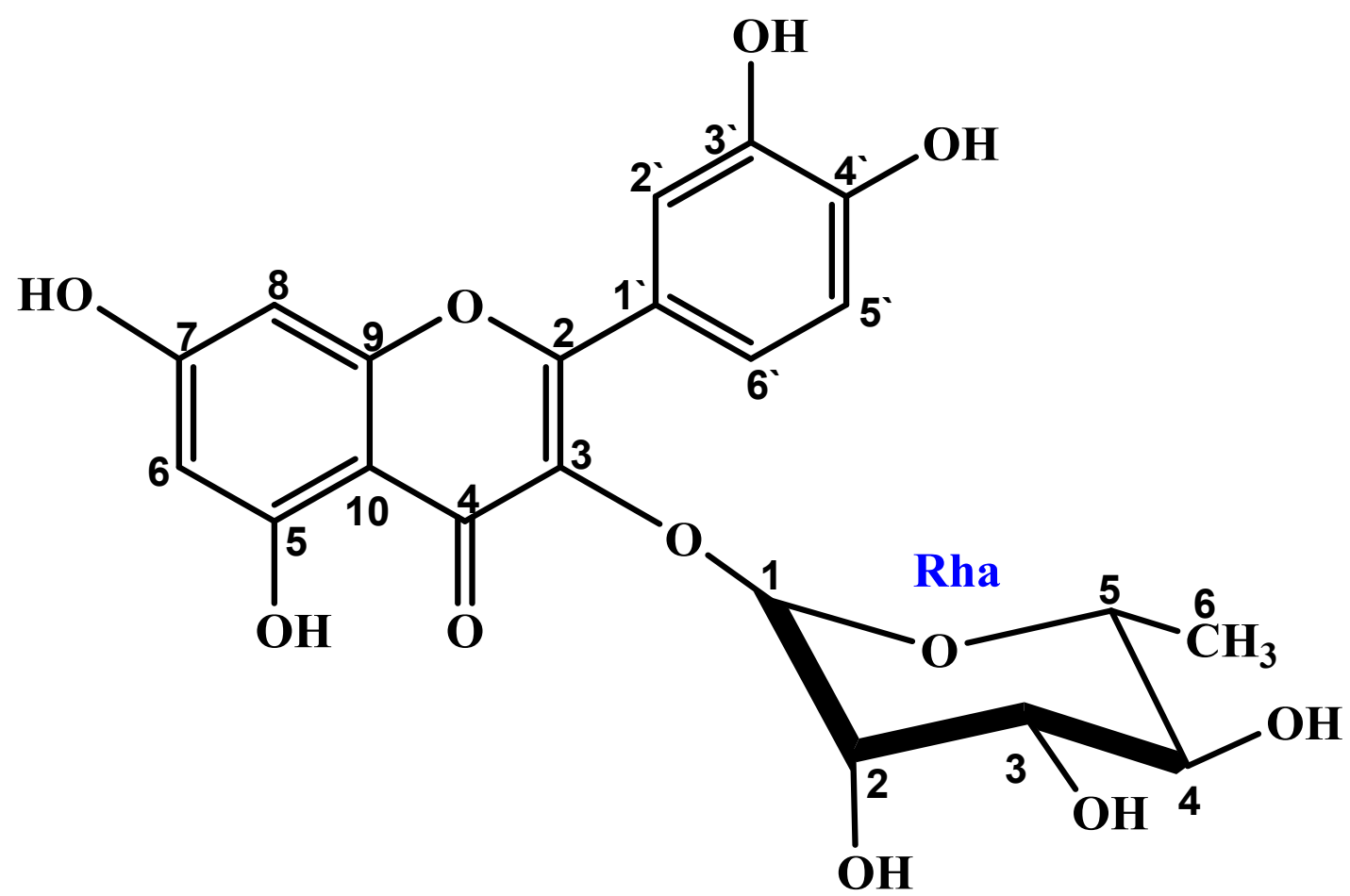

Quercetin 3-*O*-*α*-L-rhamnopyranoside

Compound **5**:	Quercetin 3-*O*-*α*-L-rhamnopyranoside (Quercitrin)
Molecular weight:	448
Molecular formula:	$C_{21}H_{20}O_{11}$
UV λ_{max} (nm):	MeOH (258, 365).
+ve ESIMS *m/z*:	449 $[M+H]^+$
	303 $[M+H\text{-}Rha]^+$
-ve ESIMS *m/z*:	447 $[M-H]^-$
	895 $[2M-H]^-$

Table S6: ^{1}H and ^{13}C NMR spectral data of compound **5** (400 MHz for ^{1}H, 100 MHz for ^{13}C, DMSO-d_6).

Position	^{1}H (*J* in Hz)	^{13}C
**Aglycone**		
2	-	157.46
3	-	134.34
4	-	177.88
5	-	161.42
6	6.21, d, 2.1	98.85
7	-	164.35
8	6.39, d, 2.1	93.79
9	-	156.59
10	-	104.22
1′	-	120.88
2′	7.30, d, 2.1	115.79
3′	-	145.32
4′	-	148.57
5′	6.87, d, 8.3	115.61
6′	7.26, dd, 8.3, 2.1	121.28
5-OH	12.65, brs	-
OH	9.52, brs	-
**Rha**		
1	5.26, brs	101.94
2	3.49, m	70.19
3	3.51, dd, 9.2, 2.8	70.49
4	3.17, dd, 9.2, 2.8	71.31
5	3.22, m	70.73
6	0.82, d, 5.9	17.62

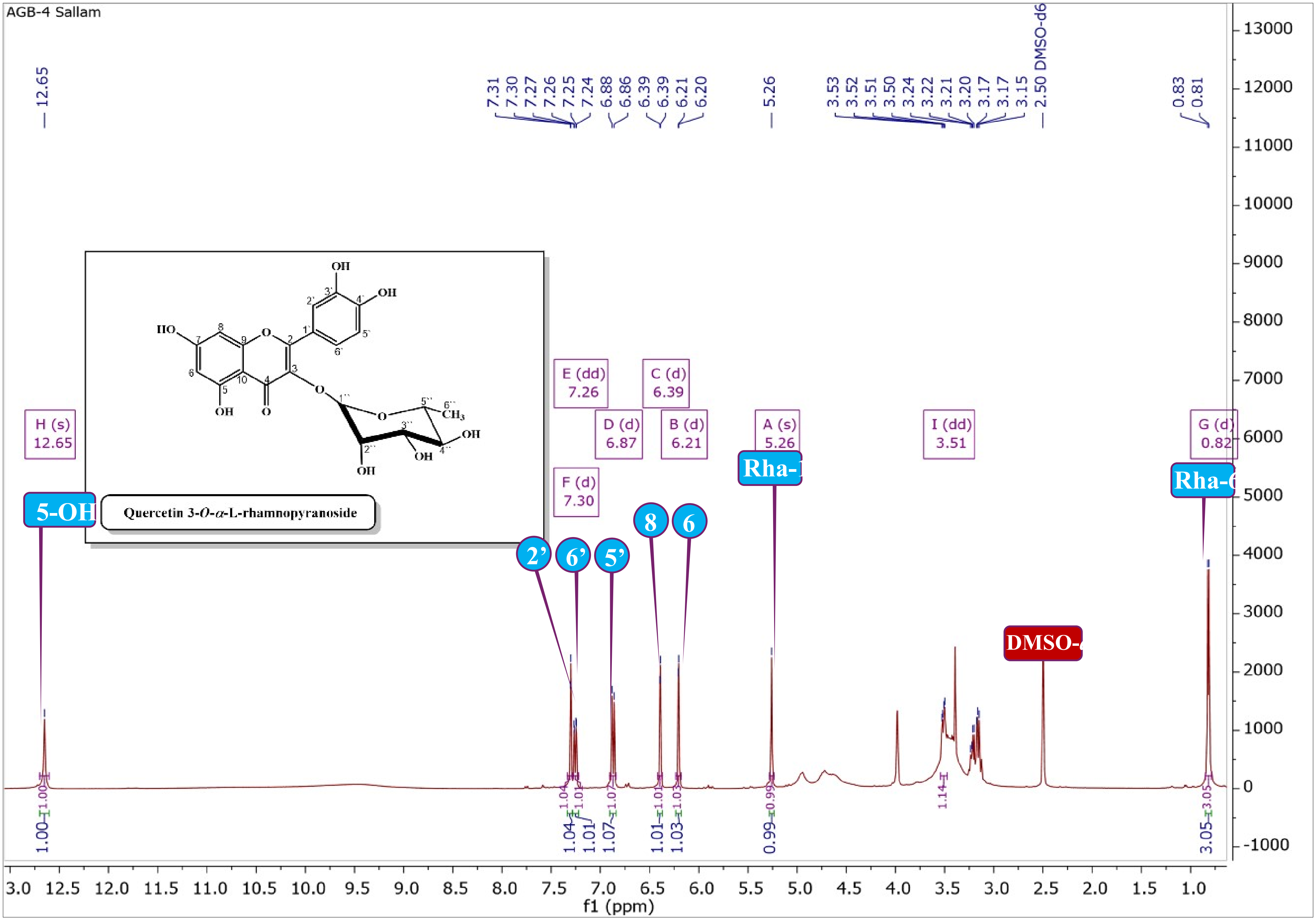

Figure S37: ^{1}H NMR spectrum of compound **5** (DMSO-d_6, 400 MHz).

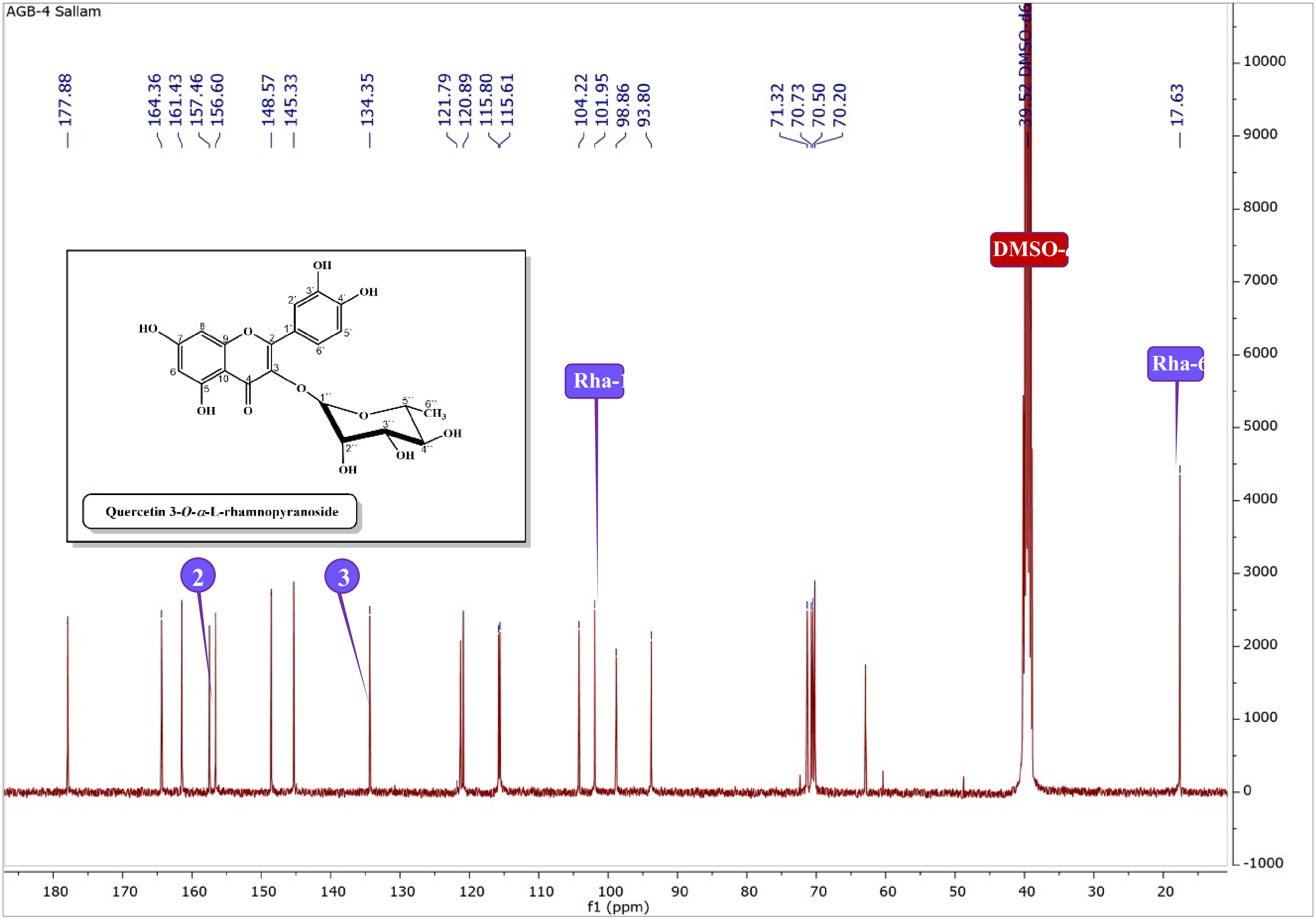

Figure S38: ^{13}C NMR spectrum of compound **5** (DMSO-d_6, 100 MHz).

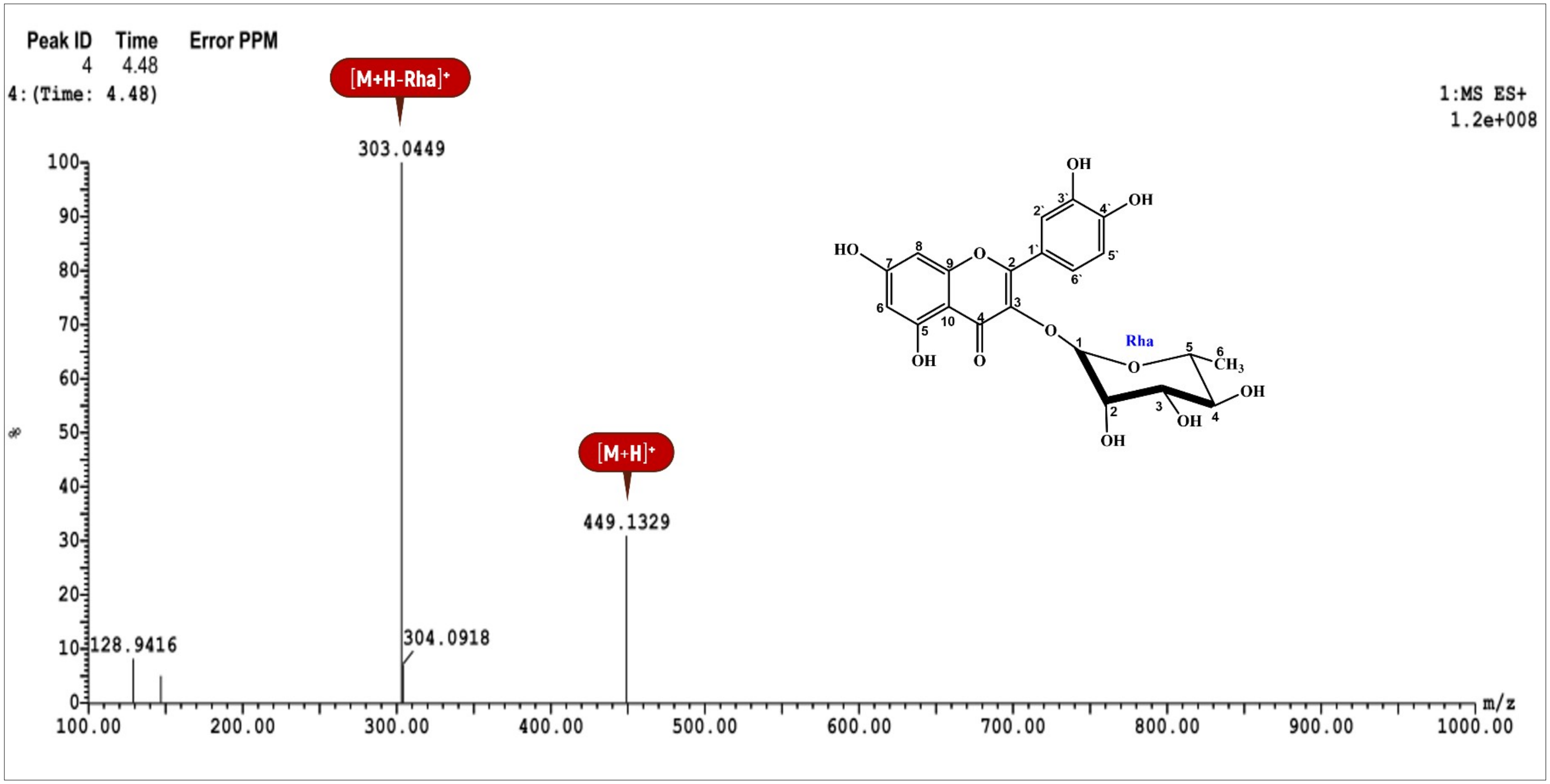

Figure S39: Positive ESI–MS spectrum of compound **5**.

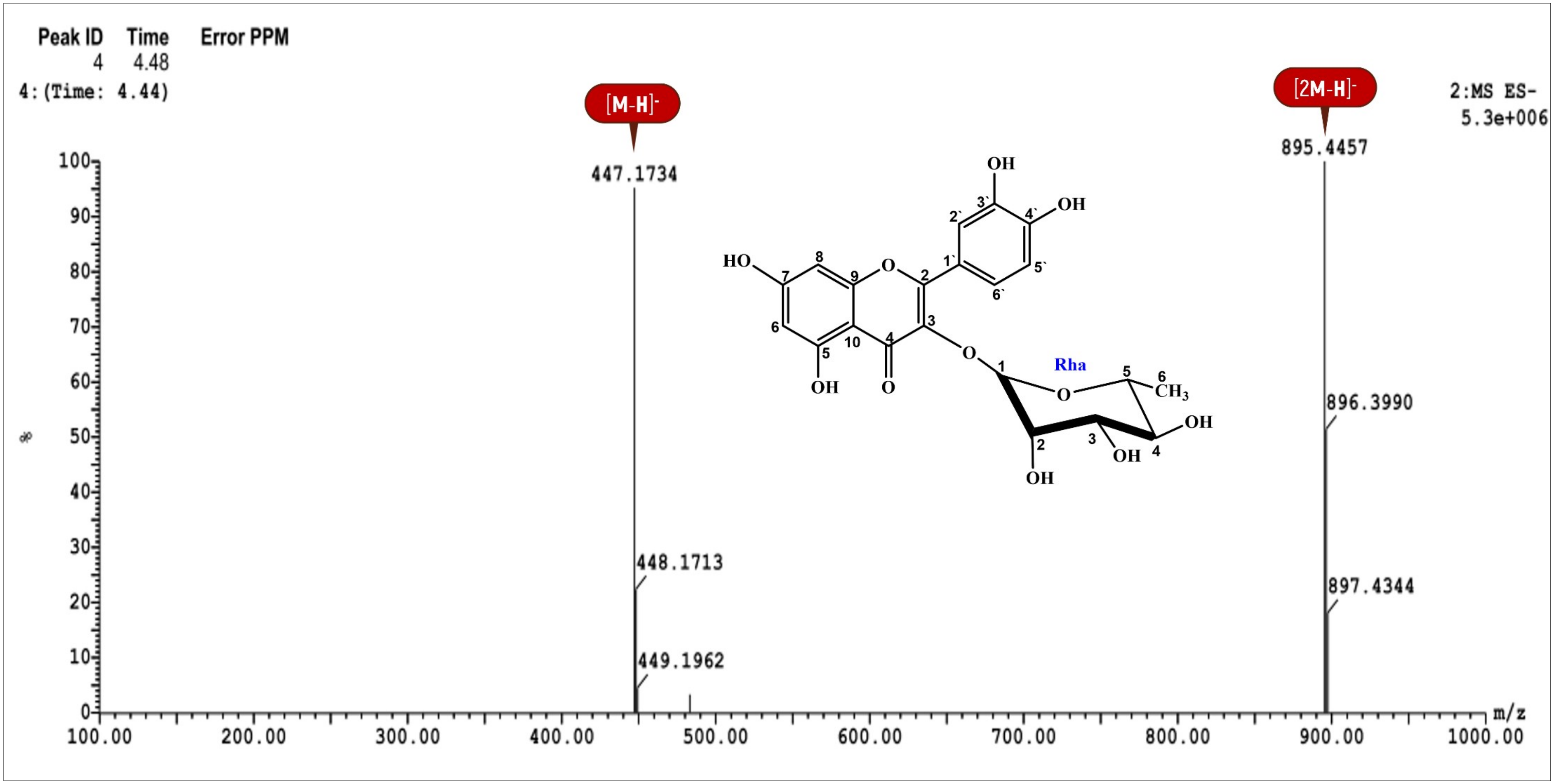

Figure S40: Negative ESI–MS spectrum of compound **5**.

Compound (6)

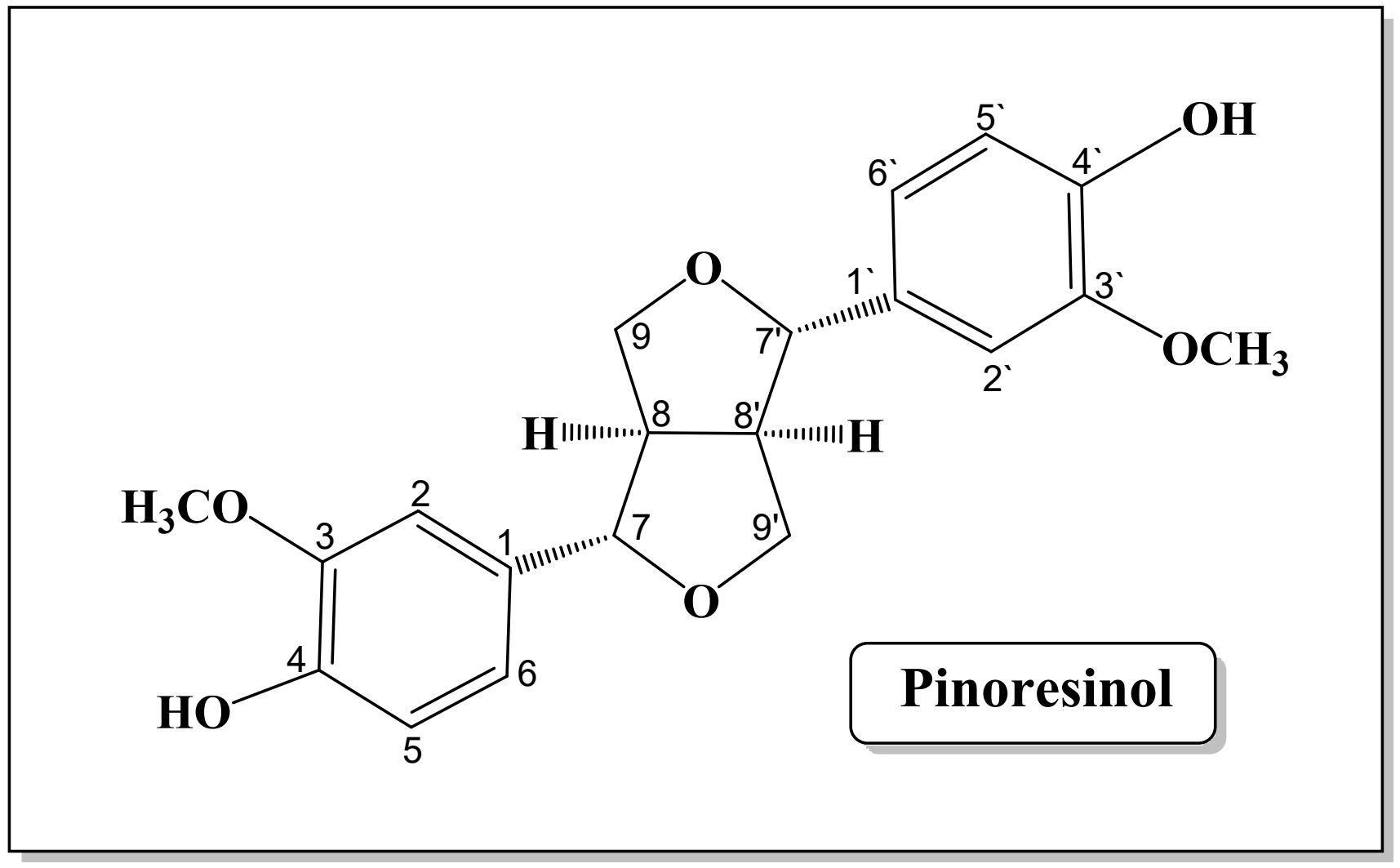

Compound **6**:	Pinoresinol
Molecular weight:	358
Molecular formula:	$C_{20}H_{22}O_6$
+ve ESI–MS *m/z*:	359 $[M+H]^+$
	717 $[2M+H]^+$
	341 $[M+H-H_2O]^+$
	323 $[M+H-2H_2O]^+$
-ve ESI–MS *m/z*:	357 $[M-H]^-$
	715 $[2M-H]^-$

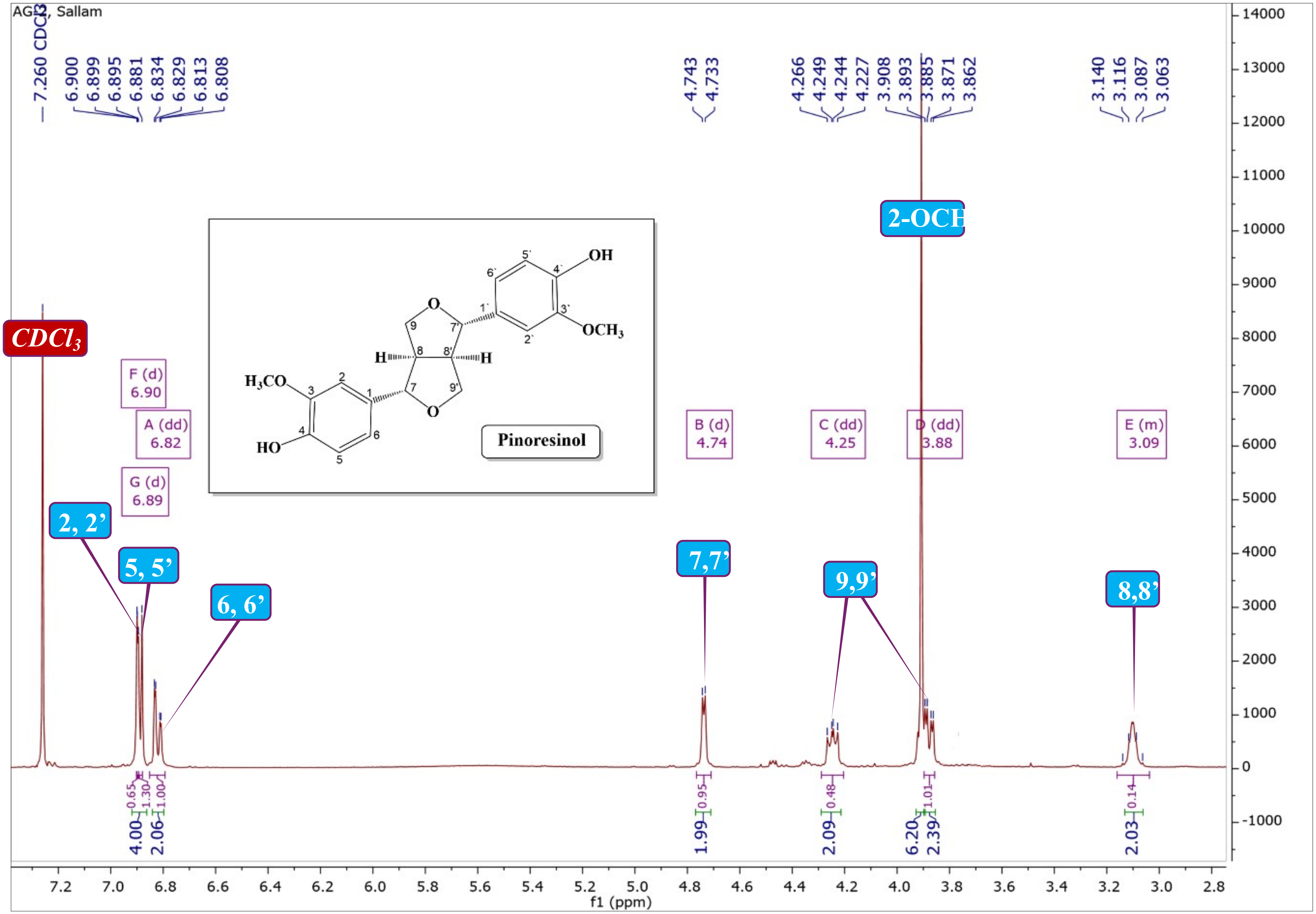

Figure S41: ^{1}H NMR spectrum of compound **6** (*$CDCl_3$*, 400 MHz).

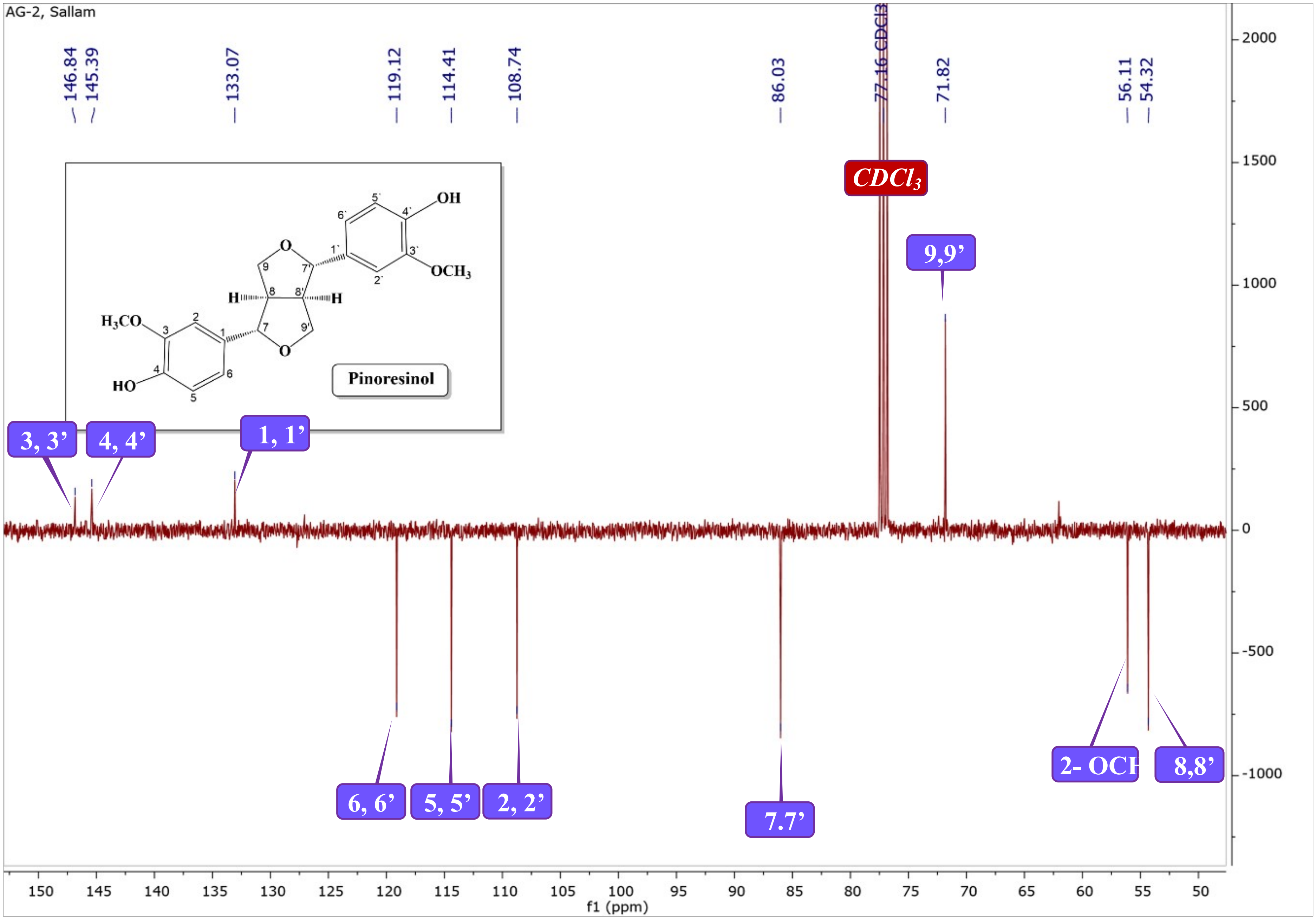

Figure S42: ^{13}C-APT NMR spectrum of compound **6** (*$CDCl_3$*, 100 MHz).

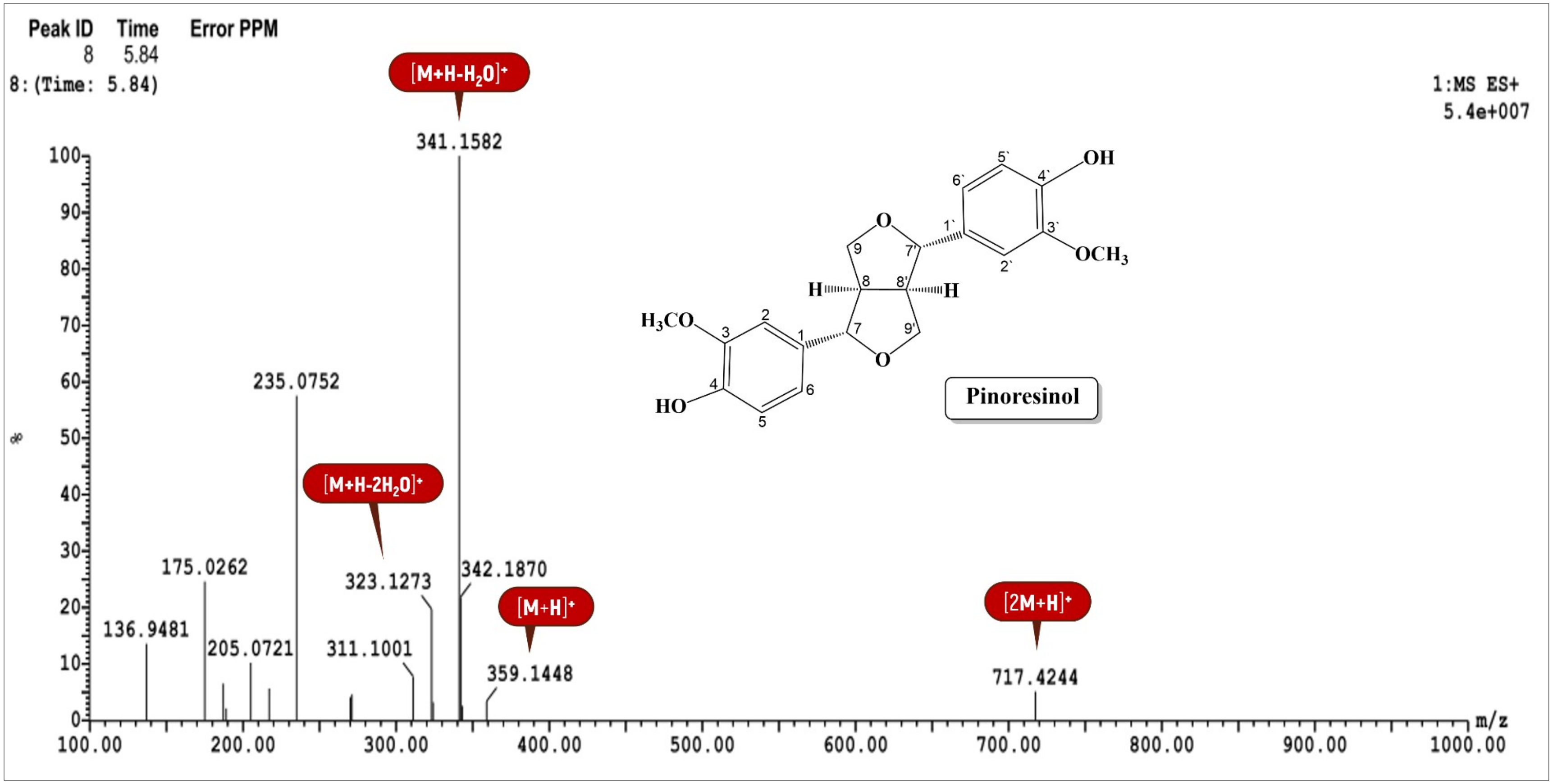

Figure S43: Positive ESI–MS spectrum of compound **6**.

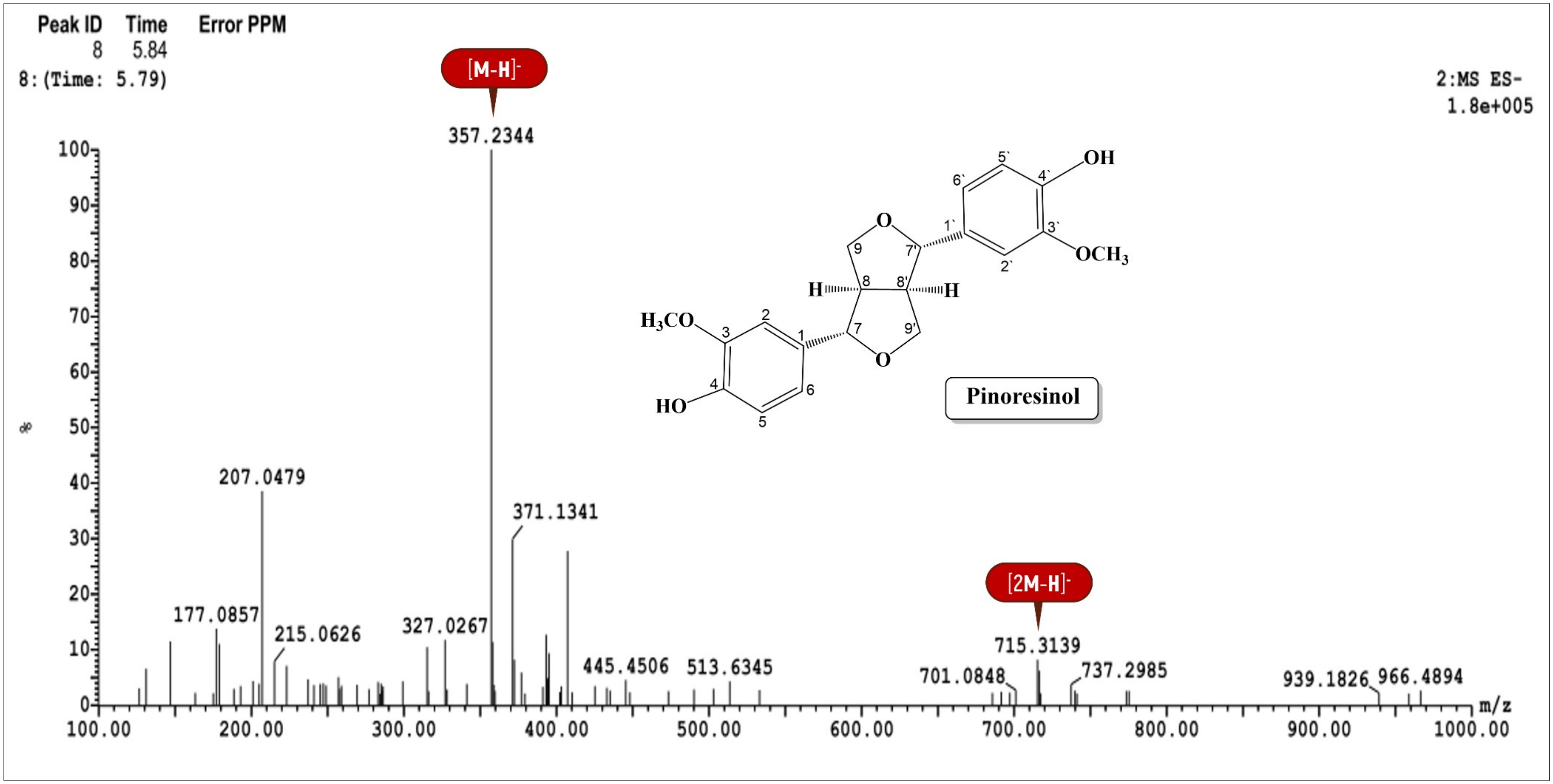

Figure S44: Negative ESI–MS spectrum of compound **6**.

Compound (7)

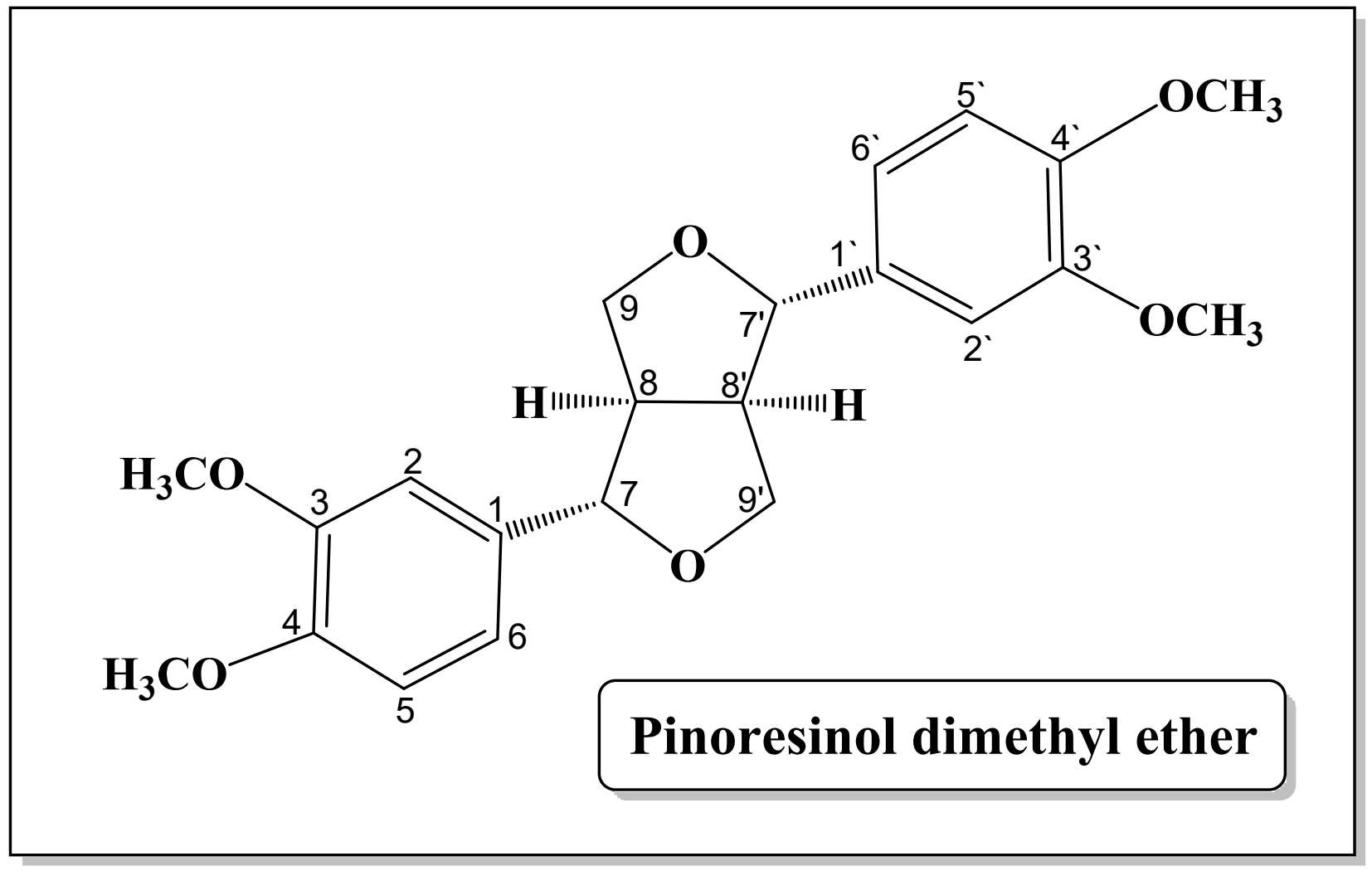

Compound **7**:	Pinoresinol dimethyl ether (Eudesmin)
Molecular weight:	386
Molecular formula:	$C_{22}H_{26}O_6$
ESI–MS *m/z*:	387 $[M+H]^+$
	369 $[M+H-H_2O]^+$
	351 $[M+H-2H_2O]^+$

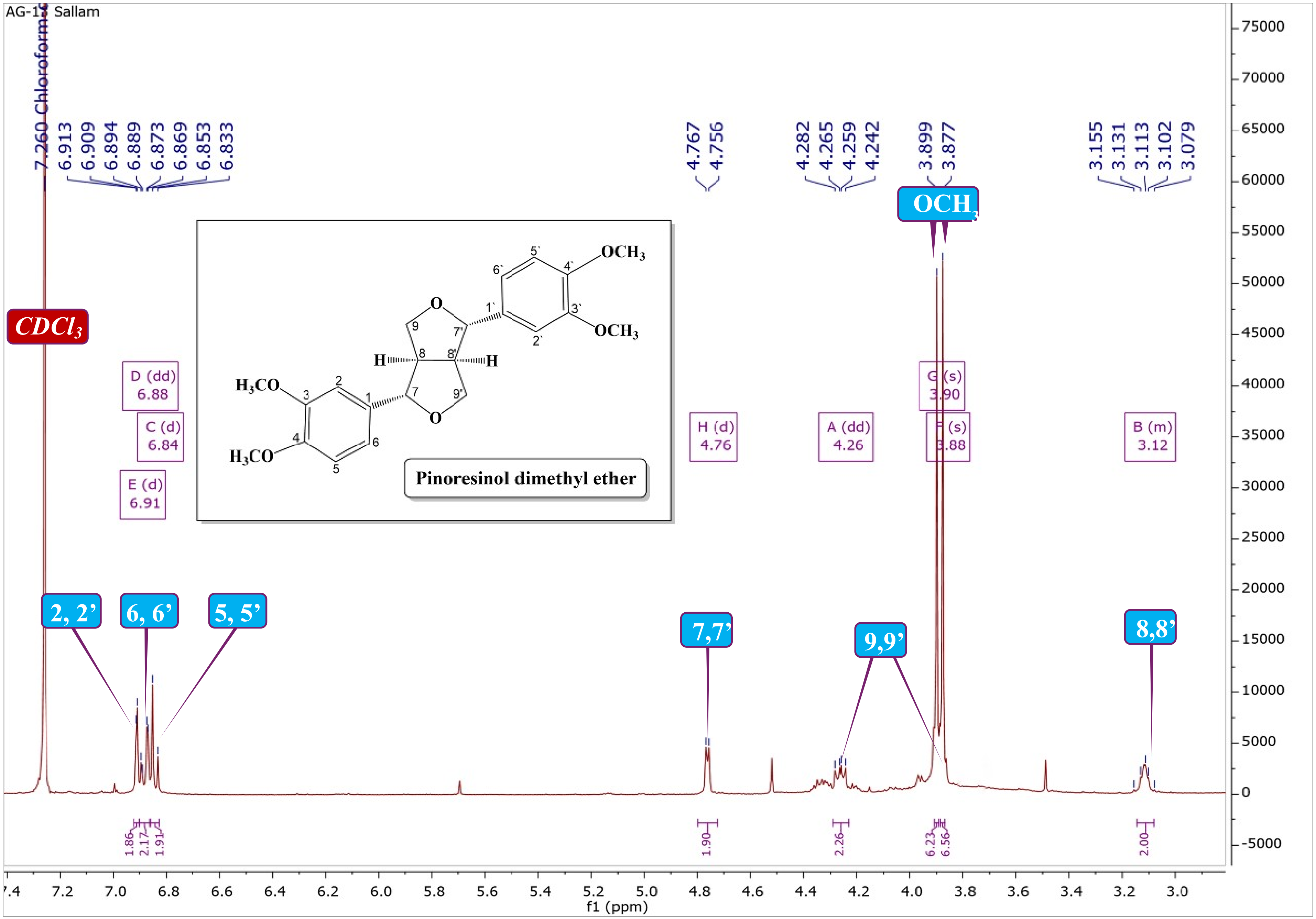

Figure S45: ^{1}H NMR spectrum of compound **7** (*CDCl$_3$*, 400 MHz).

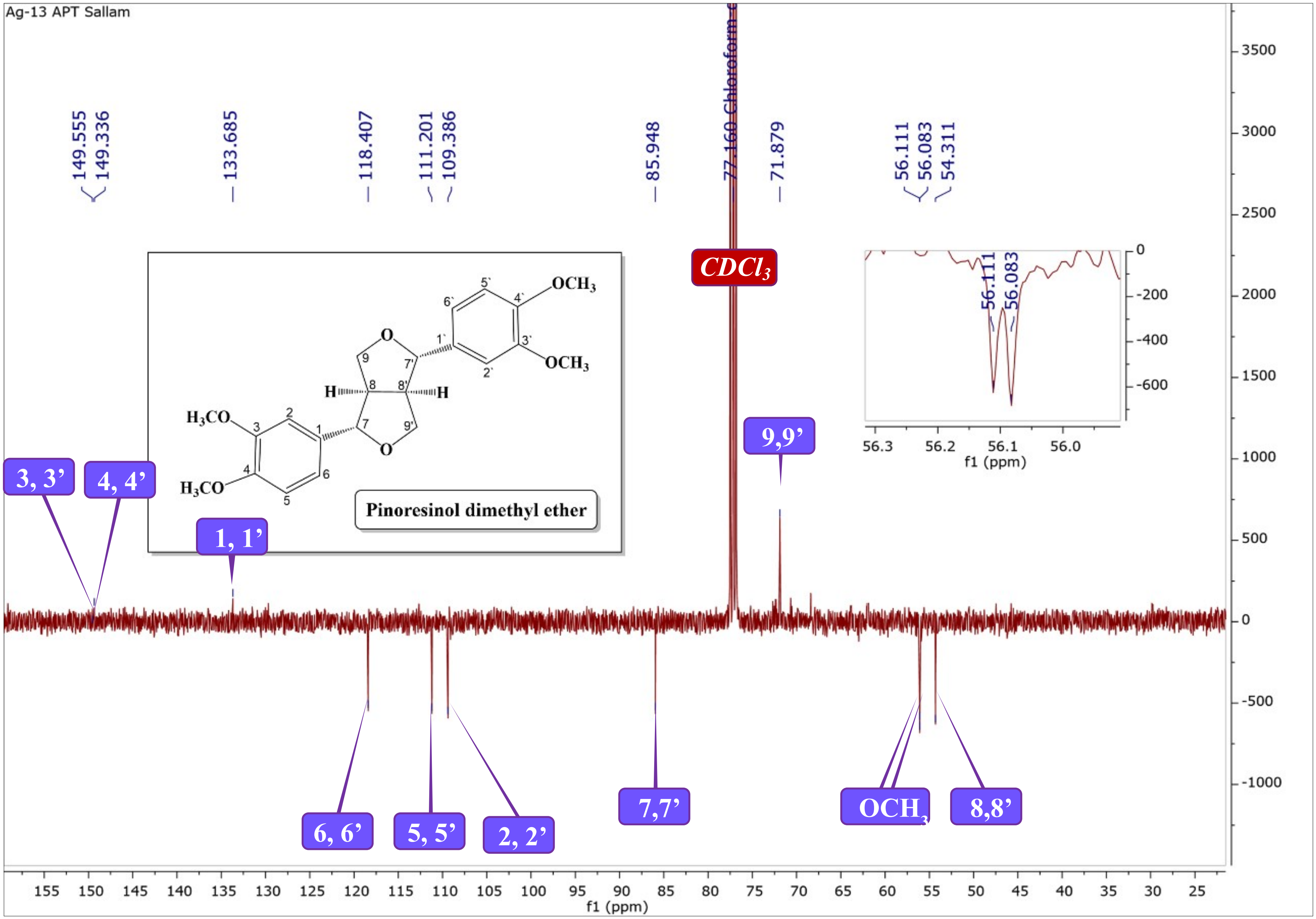

Figure S46: ^{13}C-APT NMR spectrum of compound **7** (*$CDCl_3$*, 100 MHz).

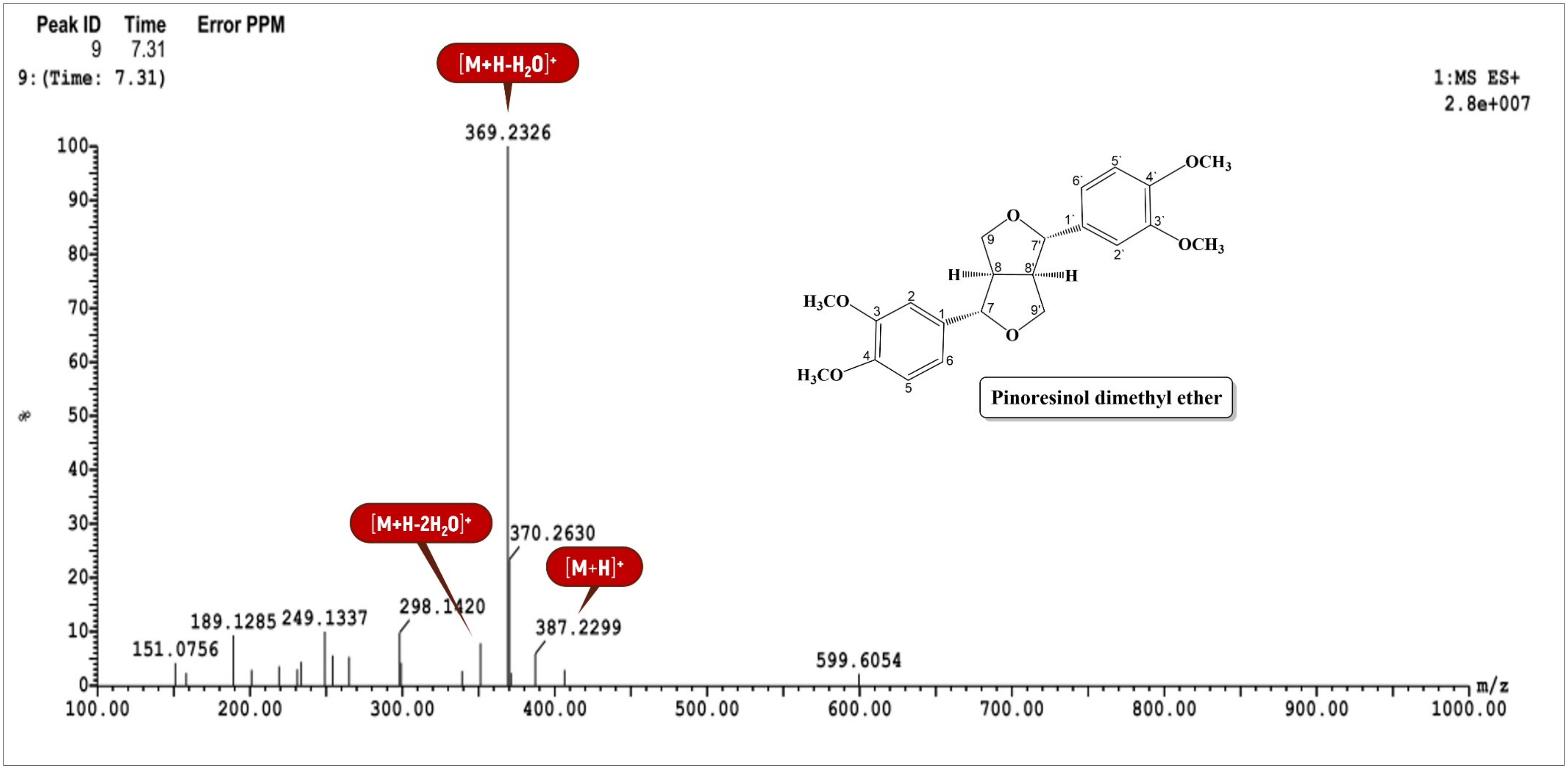

Figure S47: Positive ESI–MS spectrum of compound **7**.

Compound (8)

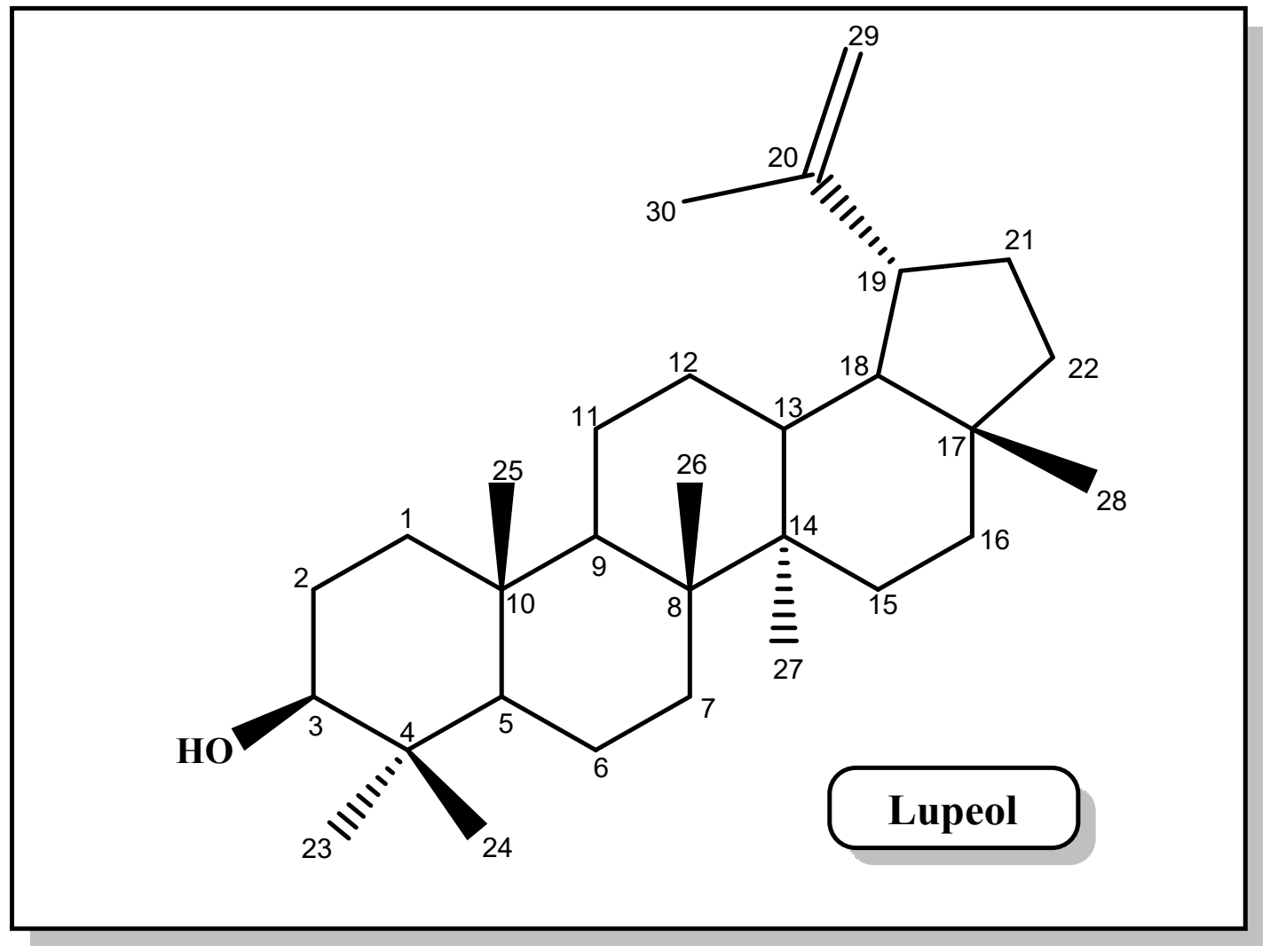

Compound **8**:	Lupeol
Molecular weight:	426
Molecular formula:	$C_{30}H_{50}O$
+ve ESI–MS *m/z*:	427 $[M+H]^+$

Table S7: ^{1}H and ^{13}C-APT NMR data of compound **8** (400 MHz for ^{1}H, 100 MHz for ^{13}C, *CDCl₃*).

Position	^{1}H (*J* in Hz)	^{13}C-APT
1	0.90, 1.65 (o)	38.85, CH_2
2	1.60, 1.57 (o)	27.59, CH_2
3	3.19, dd, 11.1, 4.7	79.16, CH
4	-	39.00, C
5	0.68, brd	55.44, CH
6	1.39, 1.52 (o)	18.46, CH_2
7	1.36, 1.42 (o)	34.42, CH_2

8	-	40.97, C
9	1.26 (o)	50.58, CH
10	-	37.31, C
11	1.22, 1.41 (o)	21.07, CH_2
12	1.07, 1.53 (o)	25.29, CH_2
13	1.66 (o)	38.20, CH
14	-	42.97, C
15	1.05, 1.60 (o)	27.56, CH_2
16	1.33, 1.46 (o)	35.73, CH_2
17	-	43.13, C
18	1.36 (o)	48.45, CH
19	2.38, td, 11.0, 5.8	48.13, CH
20	-	151.11, C
21	1.30 (o), 1.92 (m)	29.99, CH_2
22	1.18 (o)	40.15, CH_2
23	0.95, s	28.14, CH_3
24	0.83, s	15.52, CH_3
25	1.03, s	16.26, CH_3
26	0.79, s	16.12, CH_3
27	0.76, s	14.69, CH_3
28	0.97, s	18.14, CH_3
29a<br>29b	4.57, brs<br>4.69, d, 1.4	109.47, CH_2
30	1.68, s	19.45, CH_3
(o)= overlapped signals.		

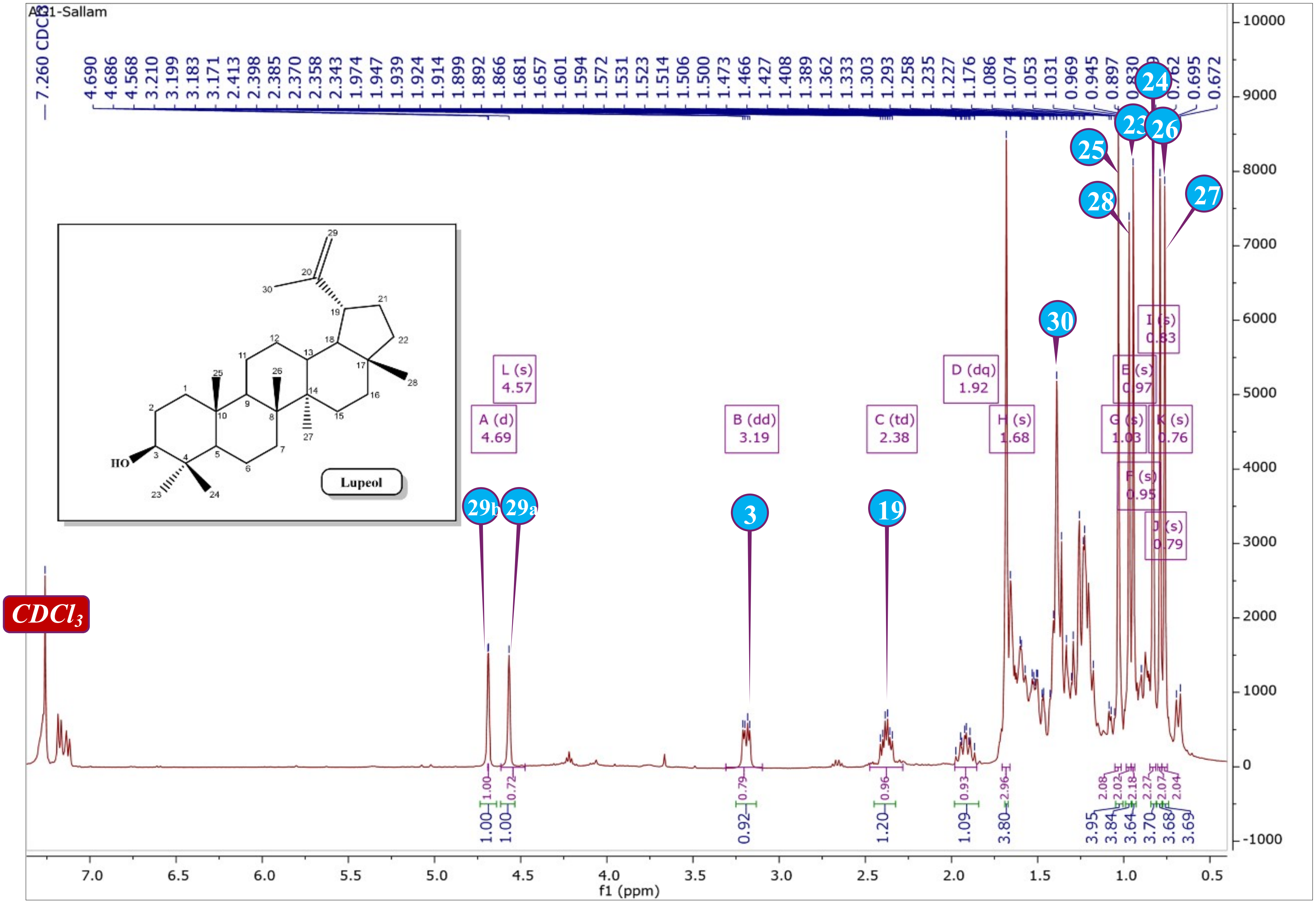

Figure S48: ^{1}H NMR spectrum of compound **8** (*$CDCl_3$*, 400 MHz).

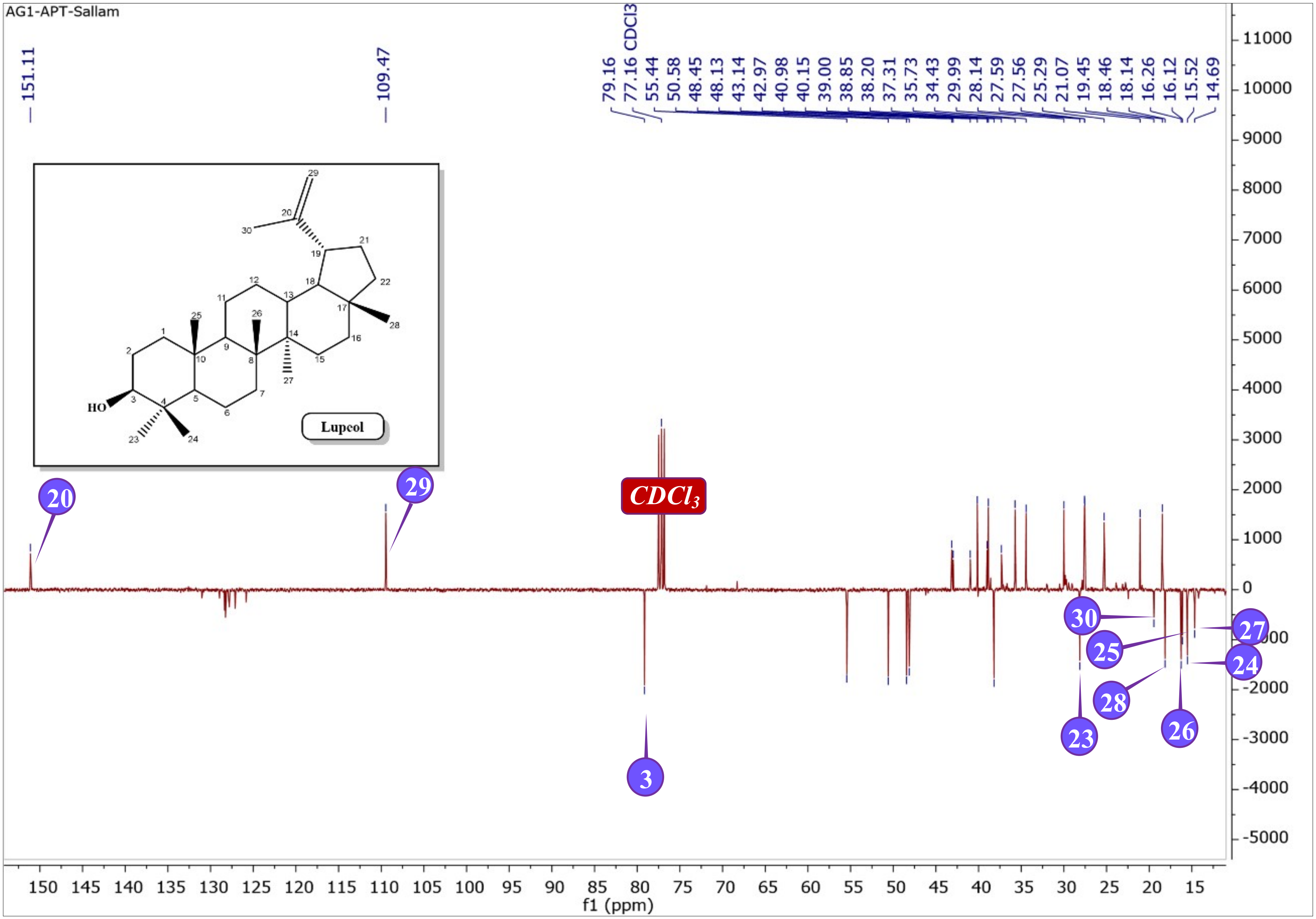

Figure S49: ^{13}C-APT NMR spectrum of compound **8** (*$CDCl_3$*, 100 MHz).

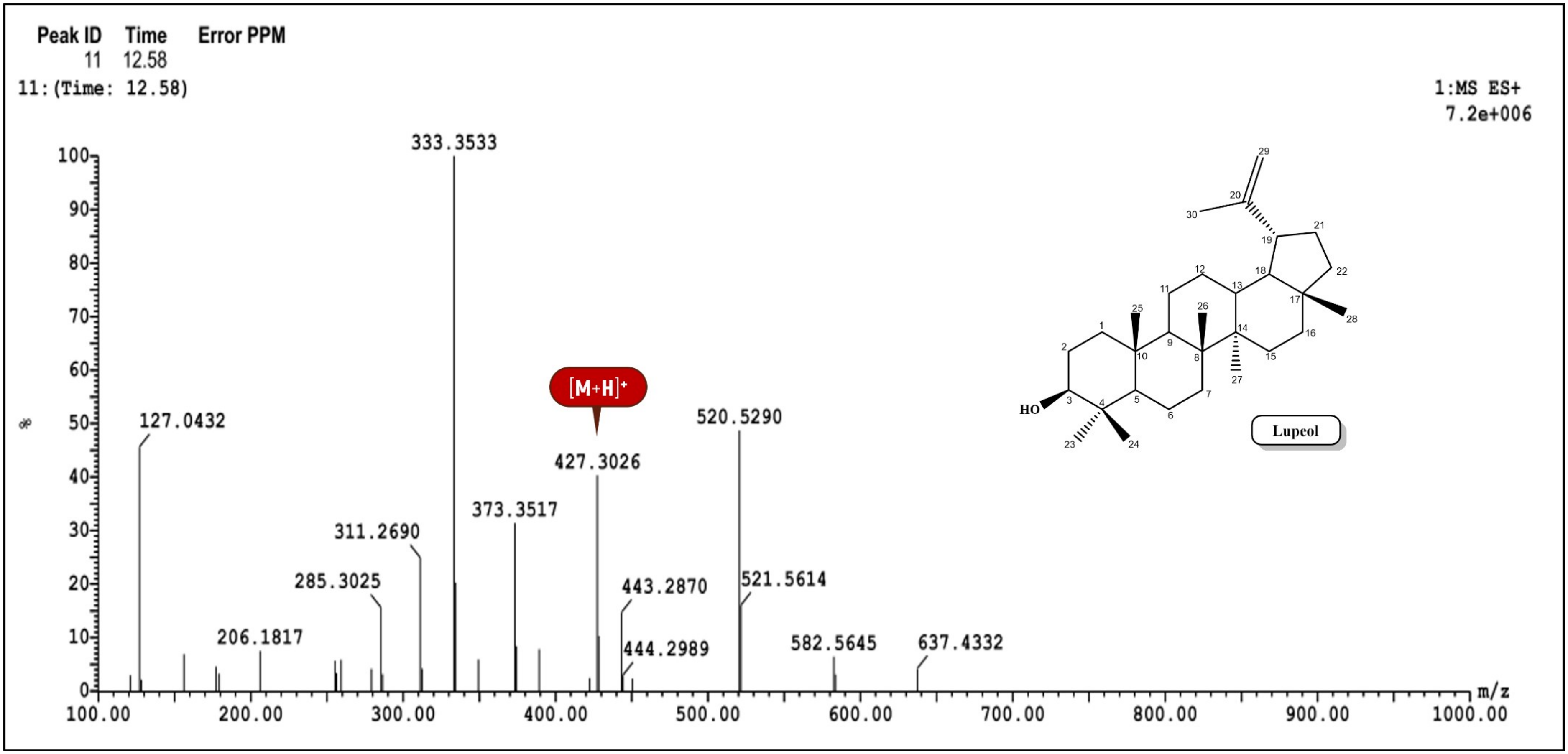

Figure S50: Positive ESI–MS spectrum of compound **8**.

Compound (9)

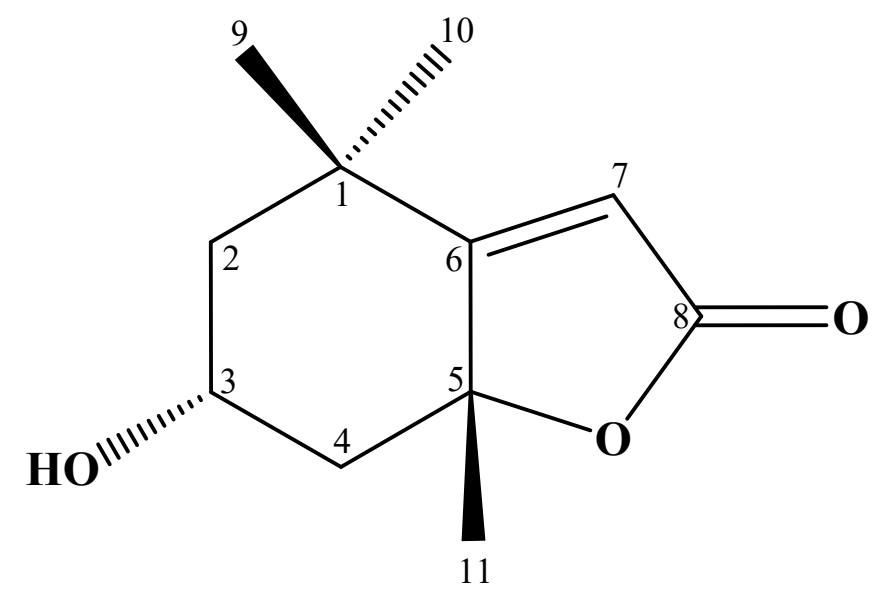

Loliolide (Calendin)

Compound [**9**]:	Loliolide (Calendin)
Molecular weight:	196
Molecular formula:	$C_{11}H_{16}O_3$
ESI–MS *m/z*:	197 $[M+H]^+$
	219 $[M+Na]^+$
	235 $[M+K]^+$
	393 $[2M+H]^+$
	415 $[2M+Na]^+$

Table S8: ^{1}H and ^{13}C-APT NMR data of compound **9** (400 MHz for ^{1}H, 100 MHz for ^{13}C, *$CDCl_3$*)

Position	^{1}H (*J* in Hz)	^{13}C-APT
1	-	36.07, C
2a<br>2b	1.53, dd, 14.6, 3.7<br>1.97, brd, 14.5	47.42, CH_2
3	4.26, m	66.95, CH
4a<br>4b	1.69 (o)<br>2.45, brd, 14.1	45.74, CH_2
5	-	86.88, C
6	-	182.63, C
7	5.62, s	113.04, CH
8	-	172.13, C
9	1.46, s	26.62, CH_3
10	1.27, s	30.79, CH_3
11	1.78, s	27.13, CH_3
(o)= overlapped signal.		

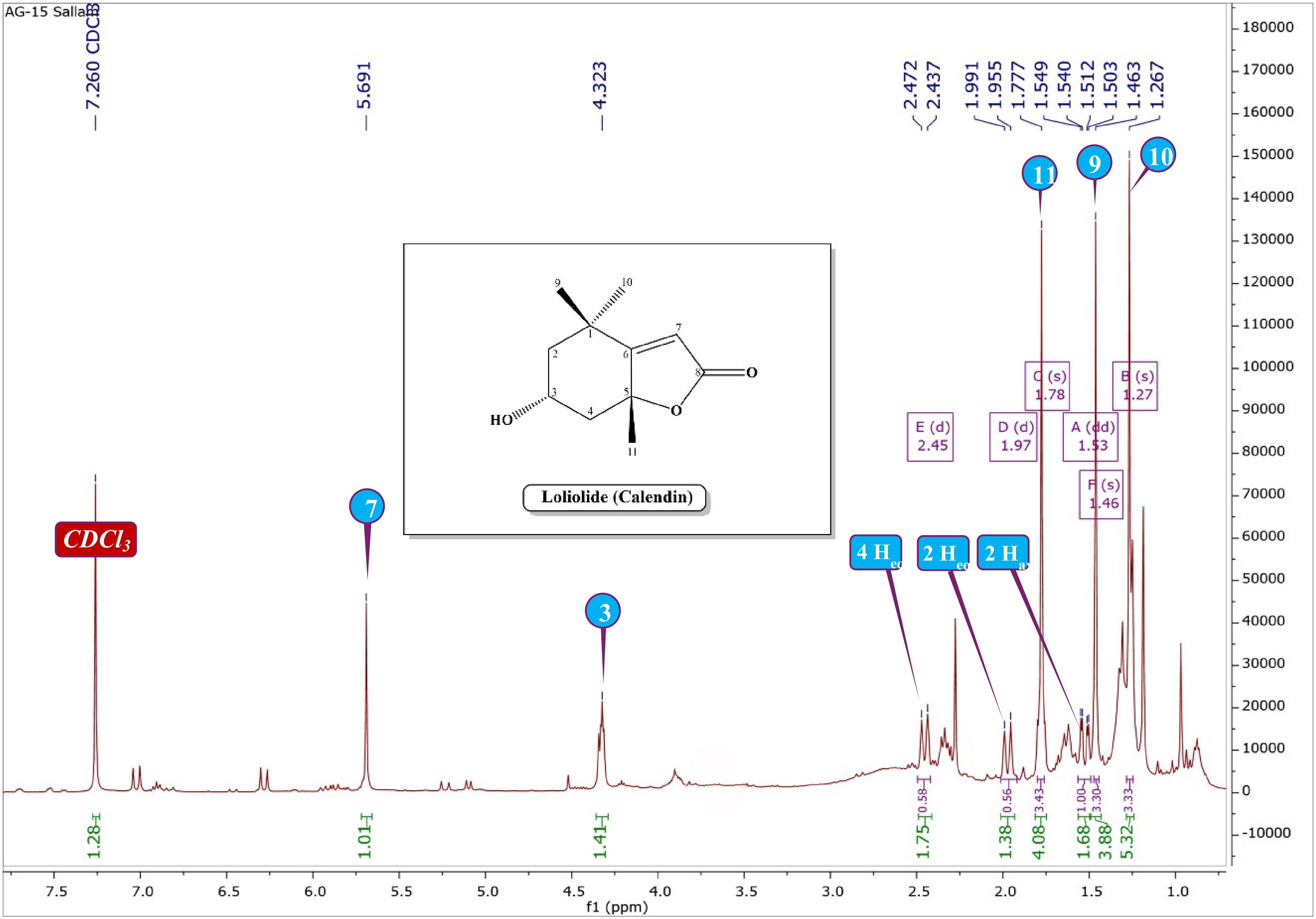

Figure S51: ^{1}H NMR spectrum of compound **9** (*$CDCl_3$*, 400 MHz).

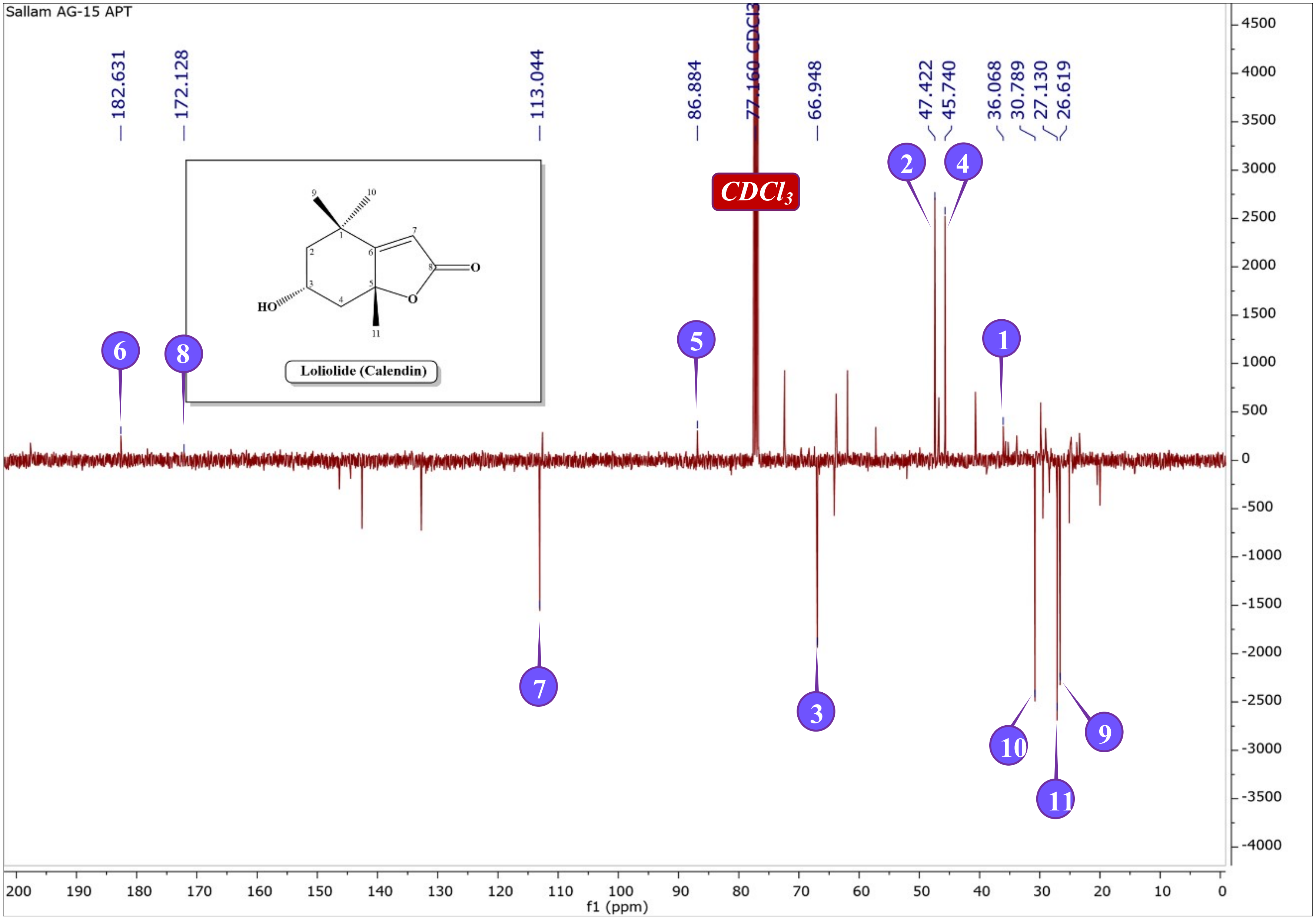

Figure S52: ^{13}C-APT NMR spectrum of compound **9** (*$CDCl_3$*, 100 MHz).

Compound (10)

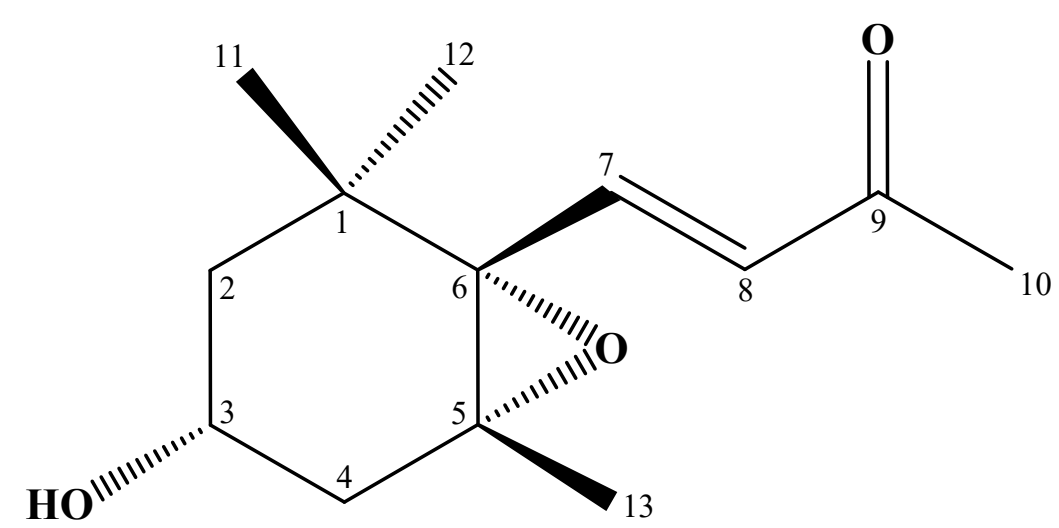

3-hydroxy-5,6-epoxy-β-ionone

Compound [**10**]:	3-hydroxy-5,6-epoxy-*β*-ionone
Molecular weight:	224
Molecular formula:	$C_{13}H_{20}O_3$
ESI-MS *m/z*:	225 $[M+H]^+$

Table S9: ^{1}H and ^{13}C-APT NMR data of compound **10** (400 MHz for ^{1}H, 100 MHz for ^{13}C, *CDCl₃*).

Position	^{1}H (*J* in Hz)	^{13}C-APT
1	-	33.81, C
2a<br>2b	1.54 (o)<br>1.33, m	46.77, CH_2
3	3.90, m	64.16, CH
4a<br>4b	1.88, m<br>2.33, dd, 14.5, 7.1	40.68, CH_2
5	-	63.70, C
6	-	72.23, C
7	7.02, d, 15.6	142.59, CH
8	6.28, d, 15.6	132.73, CH
9	-	197.63, C
10	2.28, s	28.41, CH_3
11	1.25, s	29.48, CH_3
12	0.97, s	25.10, CH_3
13	1.19, s	19.99, CH_3

(o)= overlapped signal.

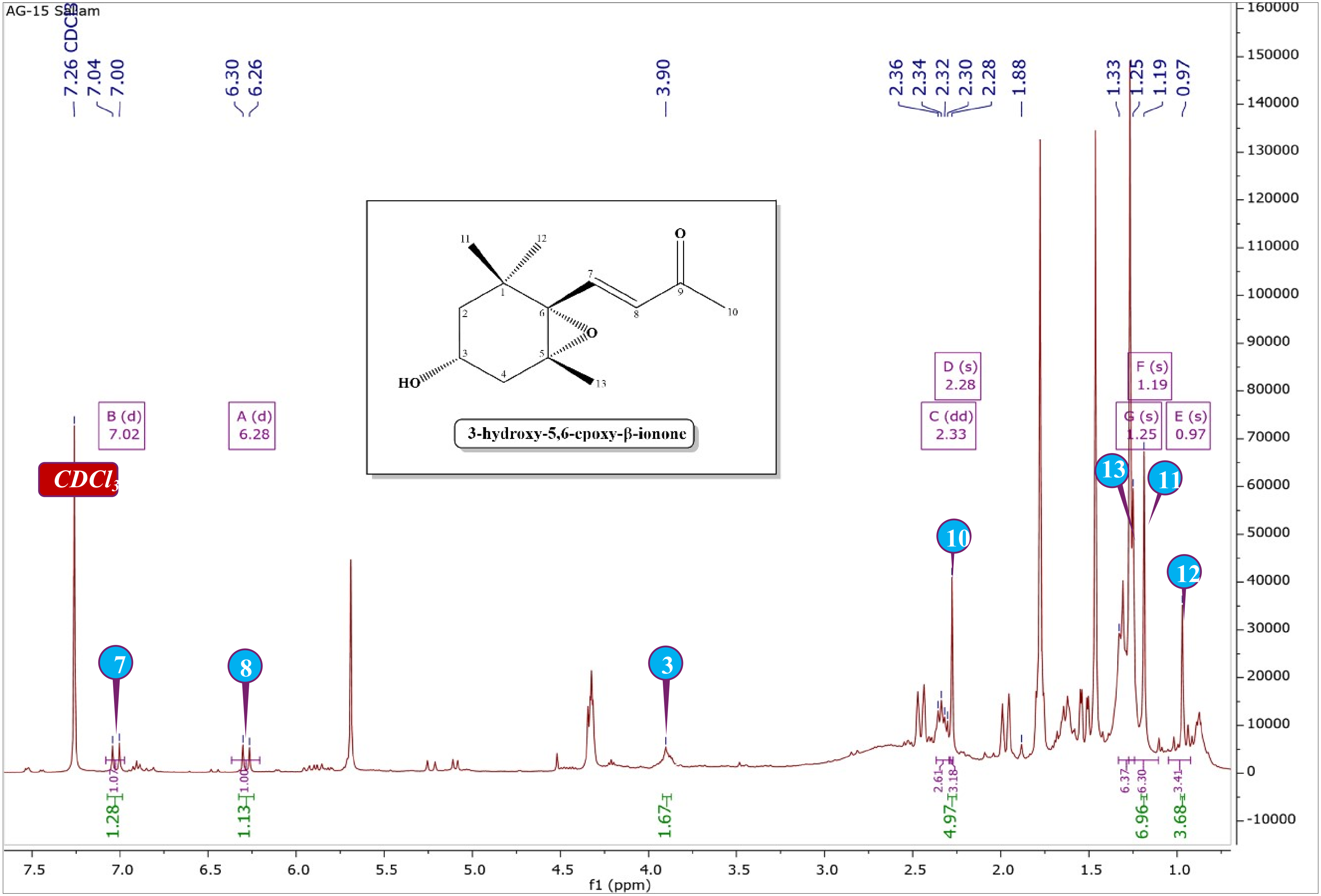

Figure S53: ^{1}H NMR spectrum of compound **10** (*CDCl3*, 400 MHz).

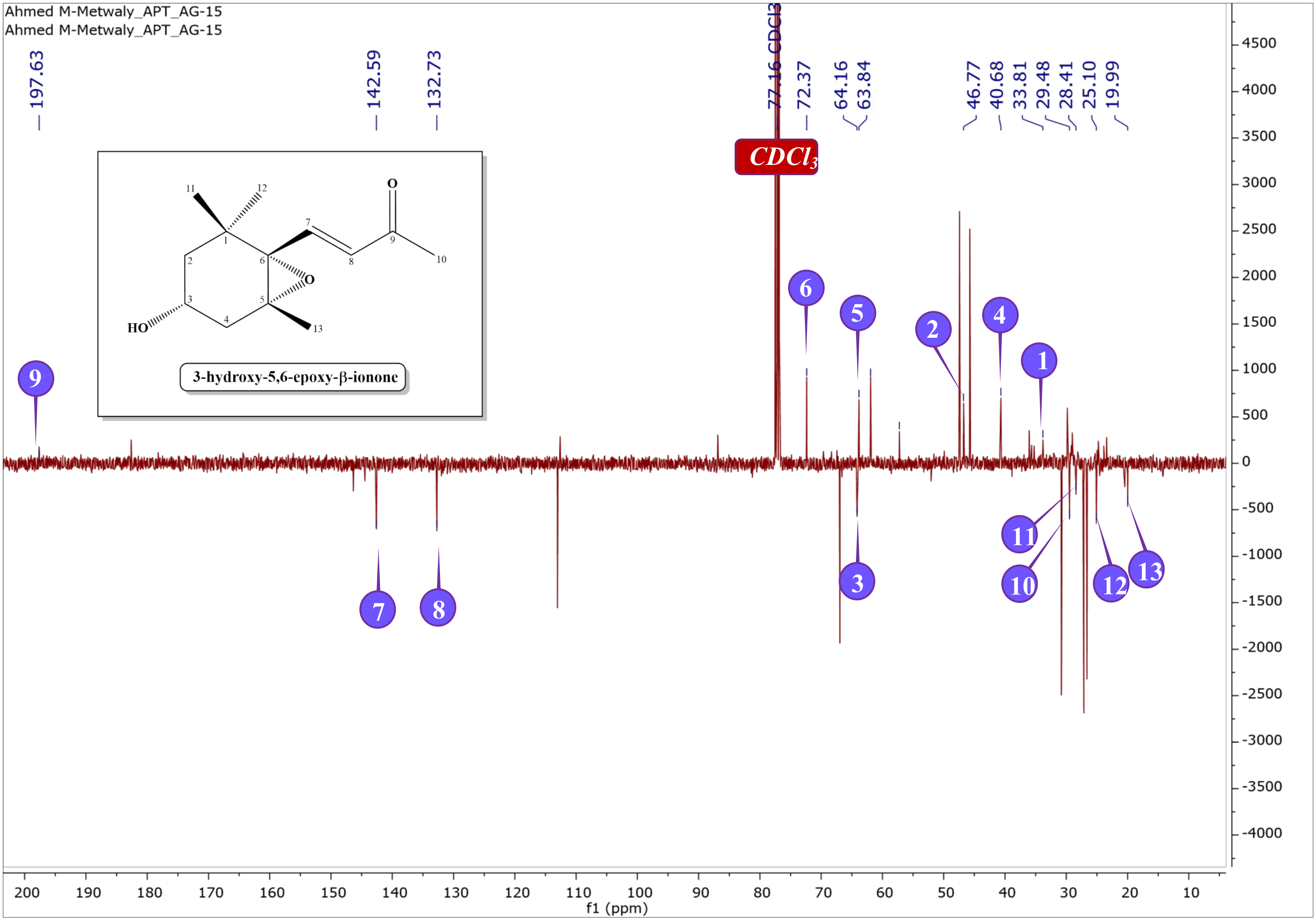

Figure S54: ^{13}C-APT NMR spectrum of compound **10** (*$CDCl_3$*, 100 MHz).

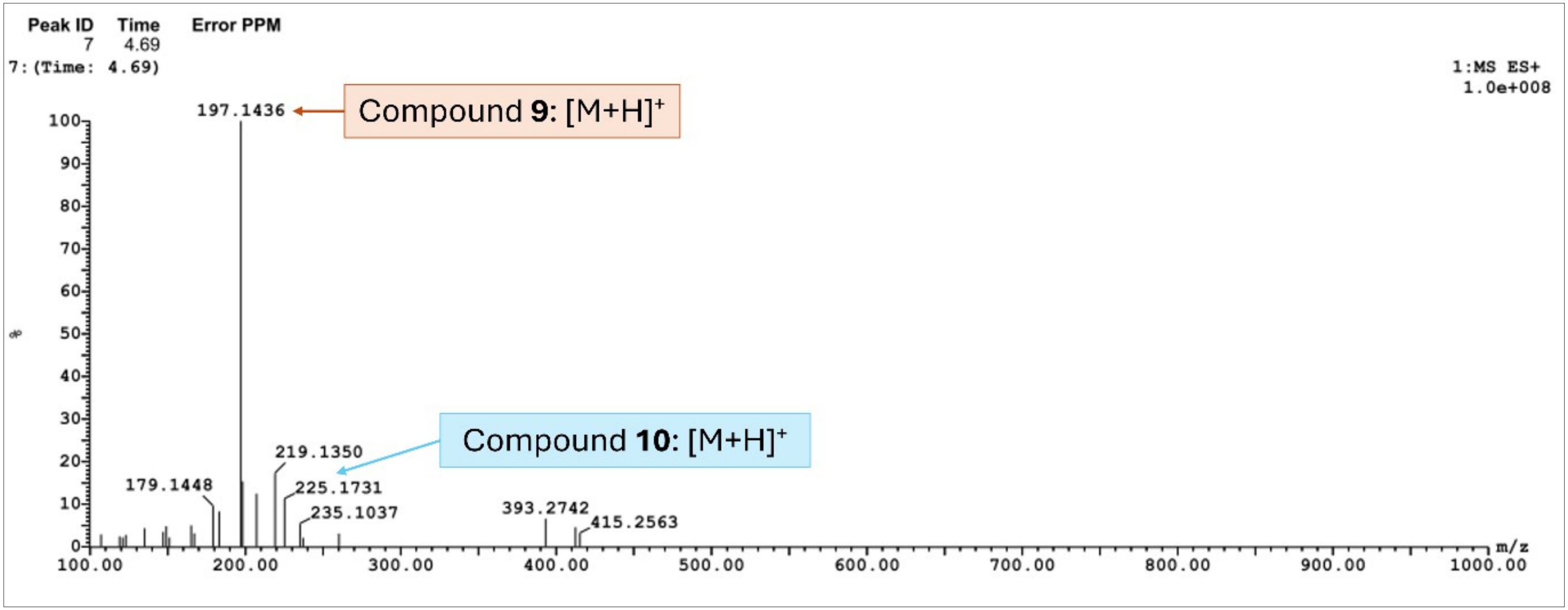

Figure S55: Positive ESI–MS spectrum of compounds **9** and **10**.

Compound (11)

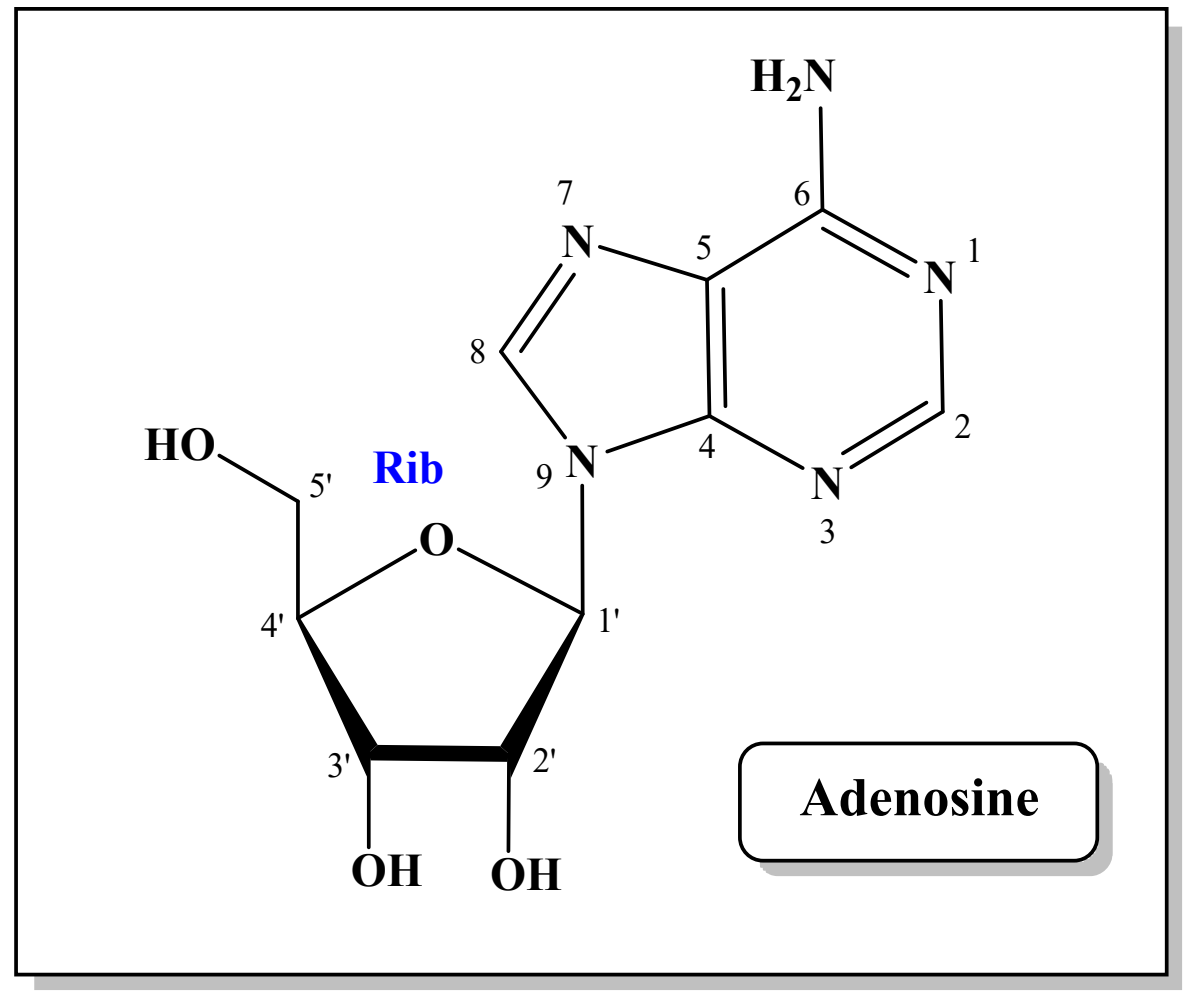

Compound **11**:	Adenosine (adenine 9-*β*-D-ribofuranoside).
Molecular weight:	267
Molecular formula:	$C_{10}H_{13}N_5O_4$
UV λ_{max} (MeOH) nm:	210, 260 nm
+ve ESIMS *m/z*:	rf
	135 [M+H-ribose]$^+$
-ve ESIMS *m/z*:	266 [M-H]$^-$

Table S10: ^{1}H and ^{13}C NMR spectral data of compound **11** (400 MHz for ^{1}H, 100 MHz for ^{13}C, DMSO-d_6).

**Position**	**^{1}H (*J* in Hz)**	**^{13}C**
2	8.15, s	152.41
4	-	149.08
5	-	119.39
6	-	156.20
8	8.36, s	139.96
NH_2	7.37, s	-
1′	5.89, d, 6.2	87.92
2′	4.63, q, 5.63, 5.63, 5.64	70.70
3′	4.15, m	73.44
4′	3.98, q, 3.42, 3.42, 3.43	85.92
5′a<br>5′b	3.69, dt, 12.04, 3.89<br>3.56, ddd, 11.58, 7.02, 3.63	61.70
2′-OH	5.47, d, 6.2	-
5′-OH	5.44, d, 4.5	-
3′-OH	5.21, d, 4.5	-

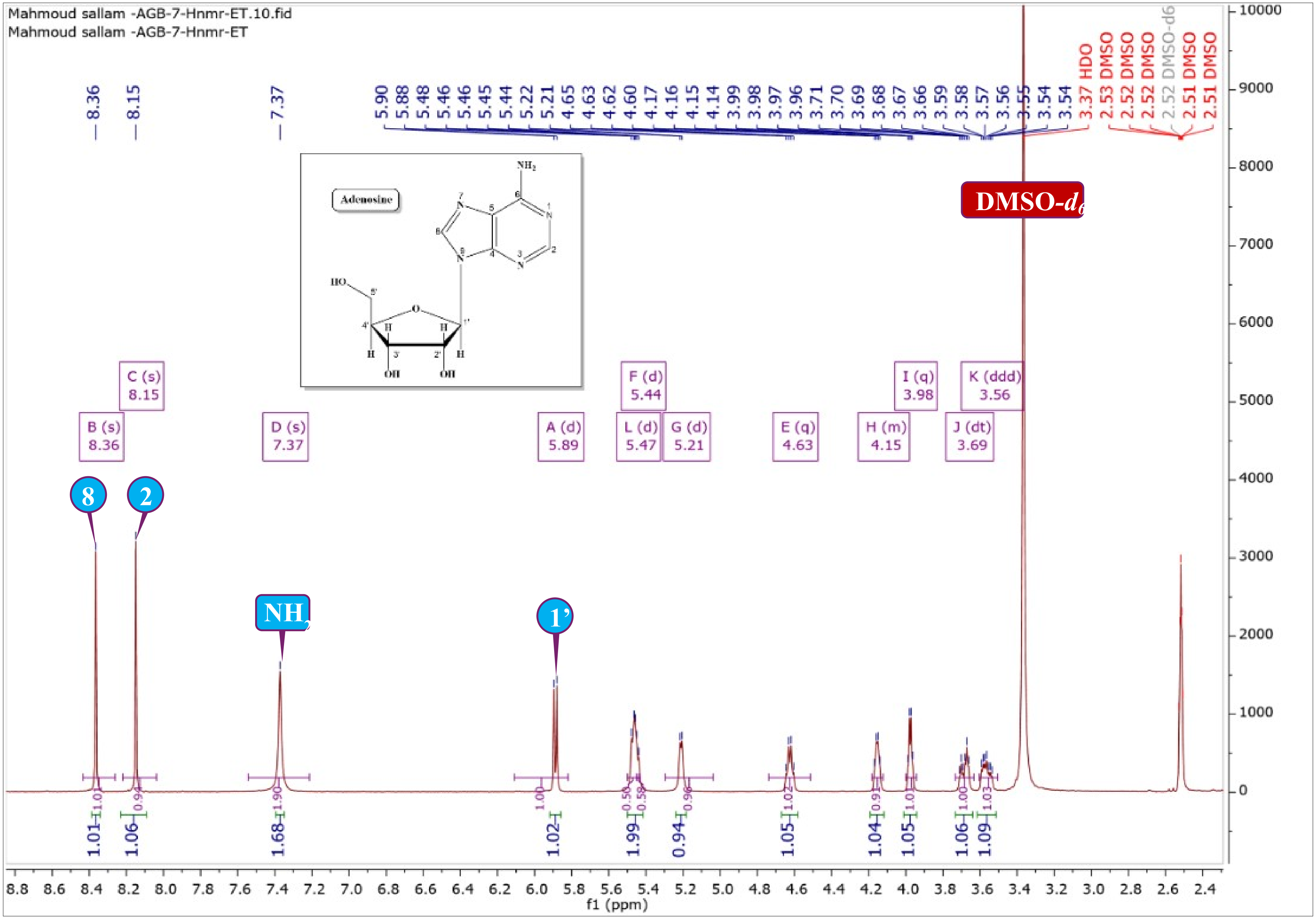

Figure S56: ^{1}H NMR spectrum of compound **11** (DMSO-d_6, 400 MHz).

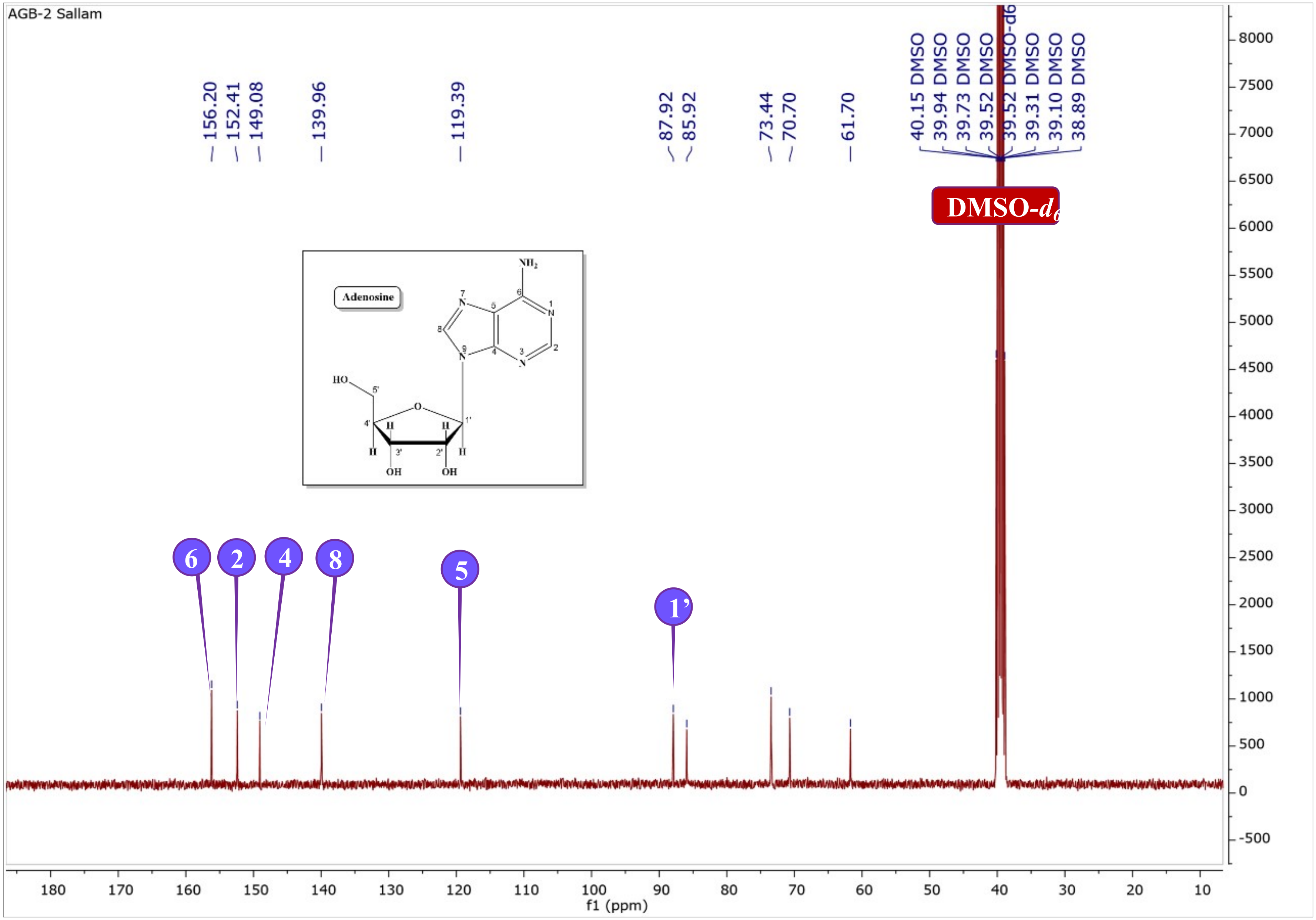

Figure S57: ^{13}C NMR spectrum of compound **11** (DMSO-d_6, 100 MHz).

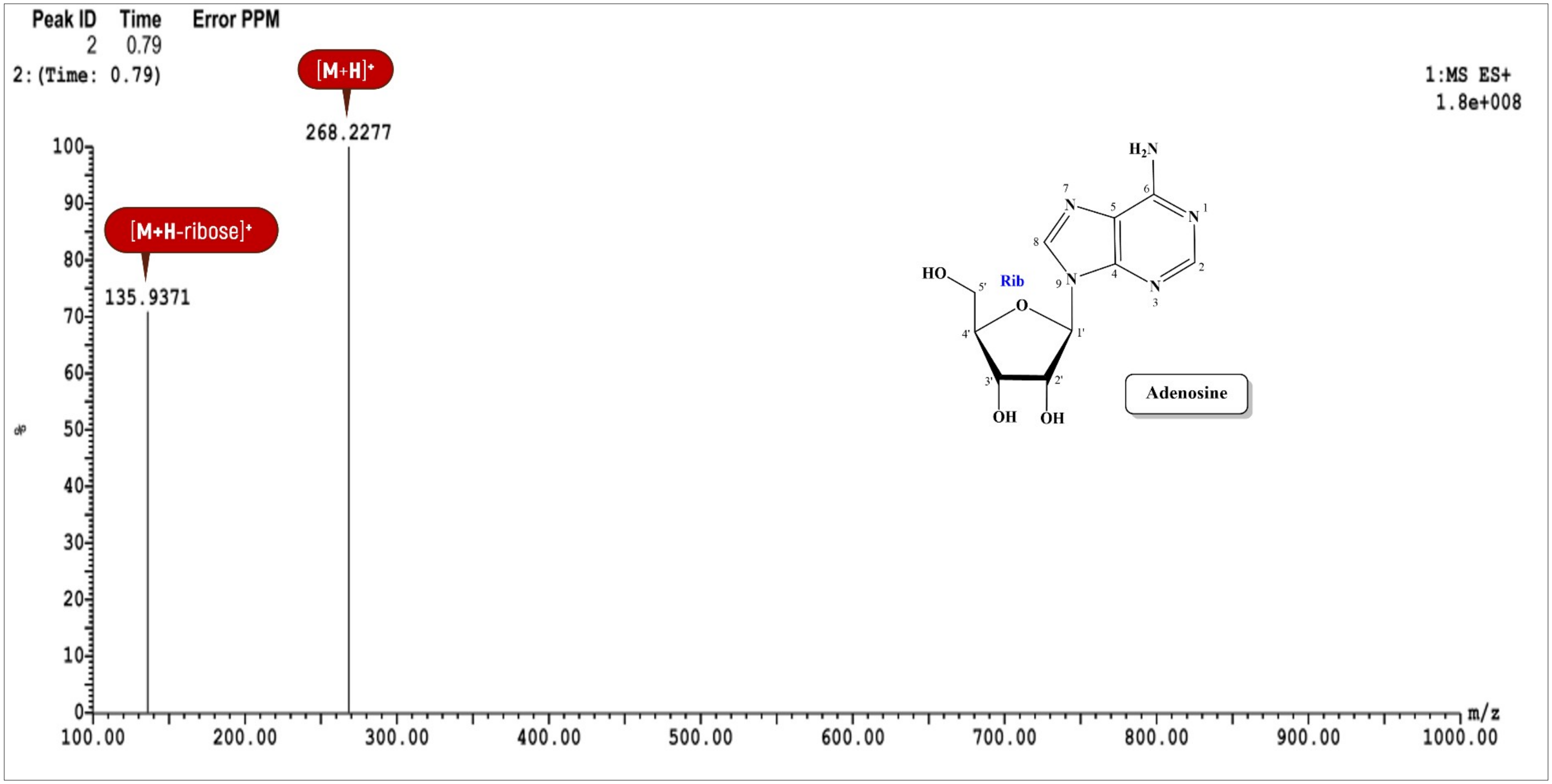

Figure S58: Positive ESI–MS spectrum of compound **11**.

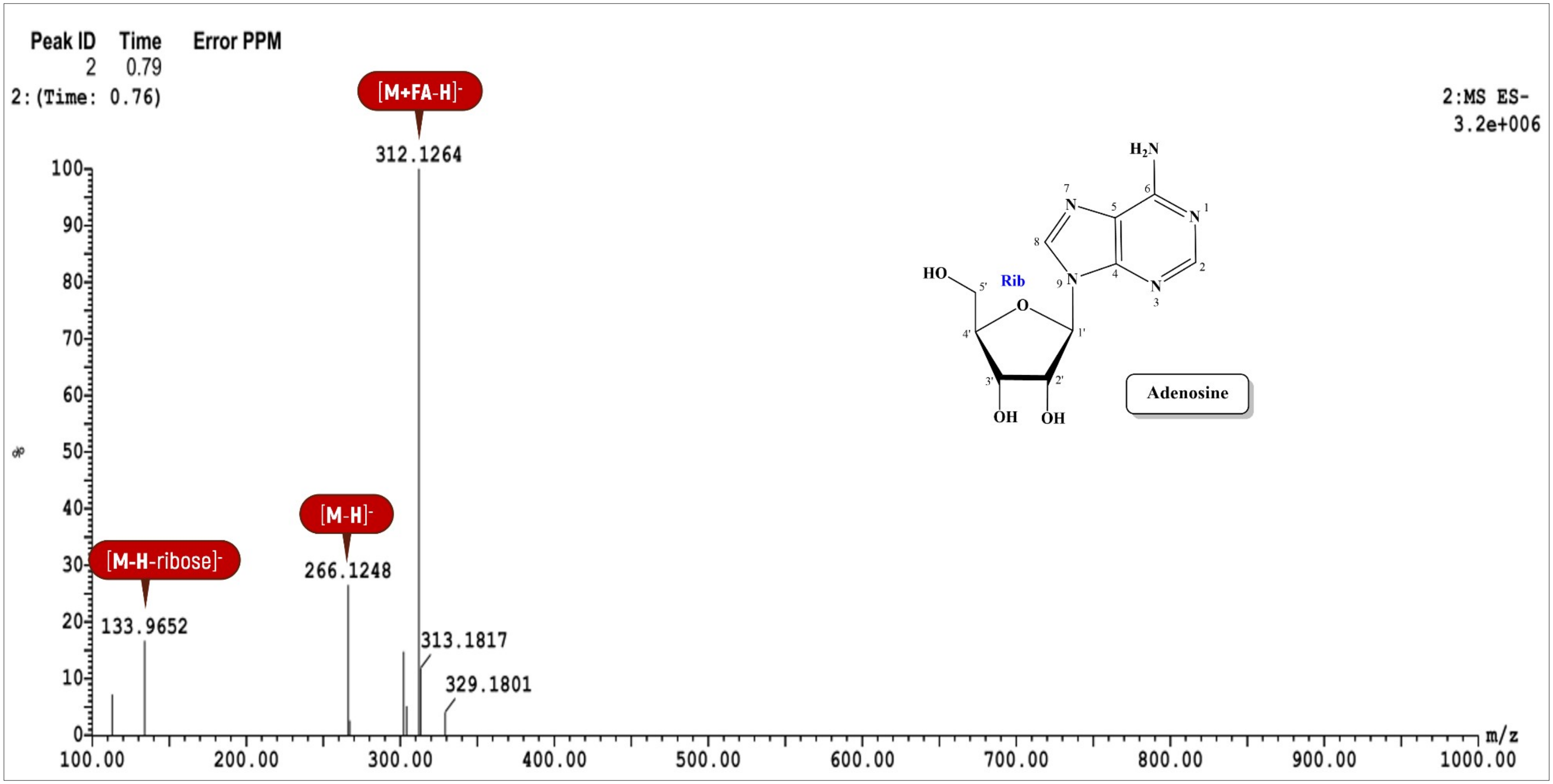

Figure S59: Negative ESI–MS spectrum of compound **11**.

Compound (12)

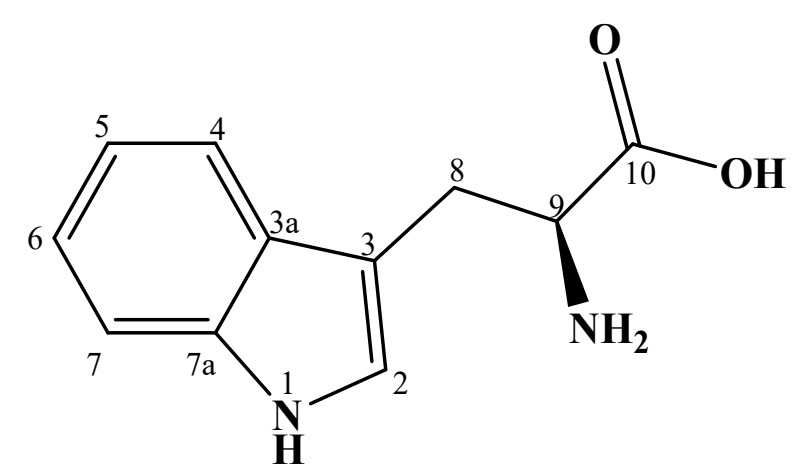

Tryptophan

Compound **12**:	Tryptophan.
Molecular weight:	204
Molecular formula:	$C_{11}H_{12}N_2O_2$
UV λ_{max} (MeOH) nm:	235, 330 nm
+ve ESI–MS *m/z*:	205 $[M+H]^+$

Table S11: ^{1}H and ^{13}C NMR spectral data of compound **12** (400 MHz for ^{1}H, 100 MHz for ^{13}C, DMSO-d_6).

**Position**	**^{1}H (J in Hz)**	**^{13}C**
1 (NH)	10.93, s	-
2	7.21, d, 1.9	124.21
3	-	109.24
3a	-	127.25
4	7.57, d, 7.9	118.36
5	6.97, dt, 7.6, 0.8	118.41
6	7.07, dt, 8.0, 0.8	120.98
7	7.35, d, 8.0	111.37
7a	-	136.36
8α<br>8β	3.31, dd, 15.2, 4.1<br>3.00, dd, 15.1, 8.7	27.03
9	3.54, 8.7, 4.1	54.54
10	-	170.34
OH	3.81, s	-

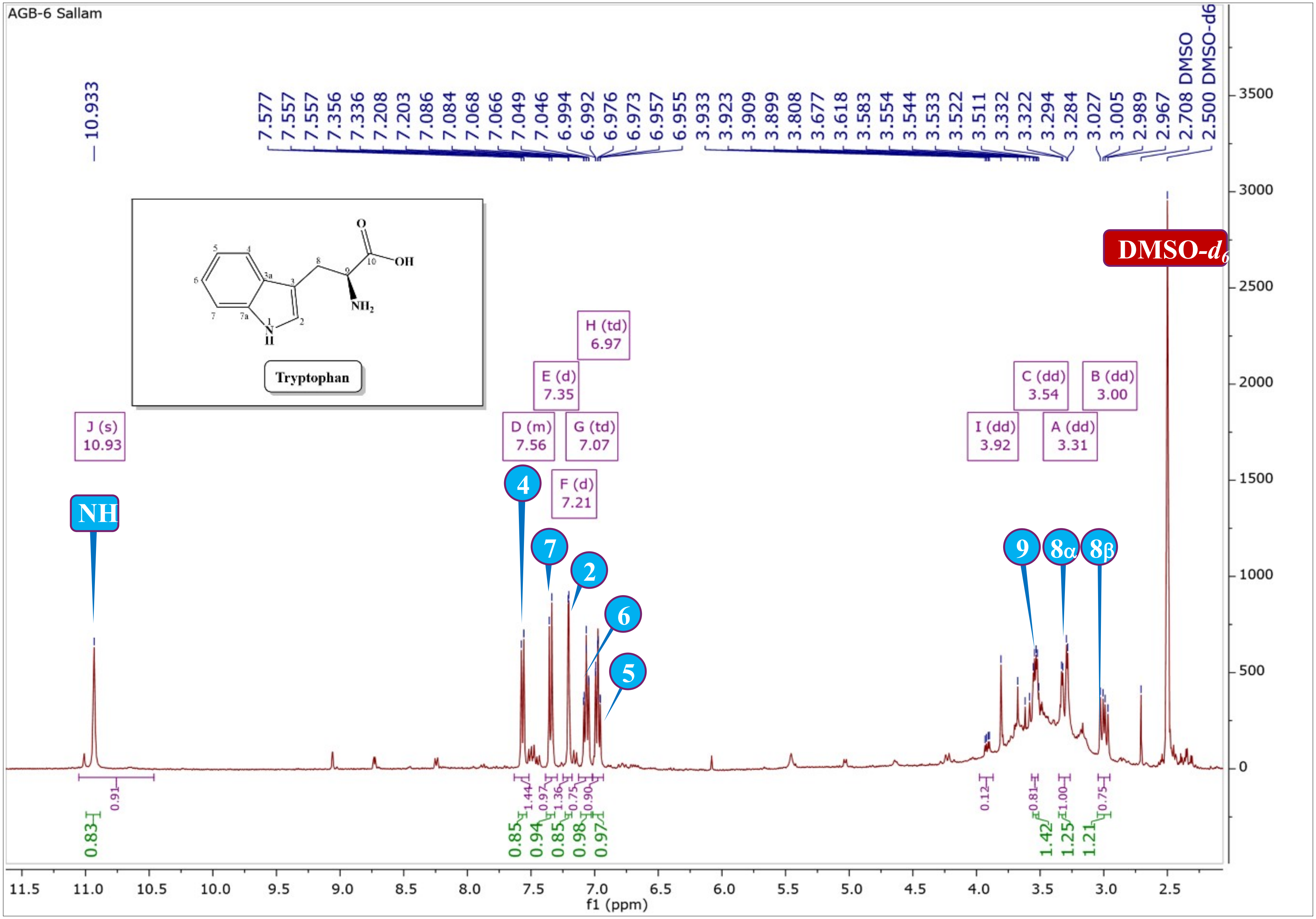

Figure S60: ^{1}H NMR spectrum of compound **12** (DMSO-d_6, 400 Hz).

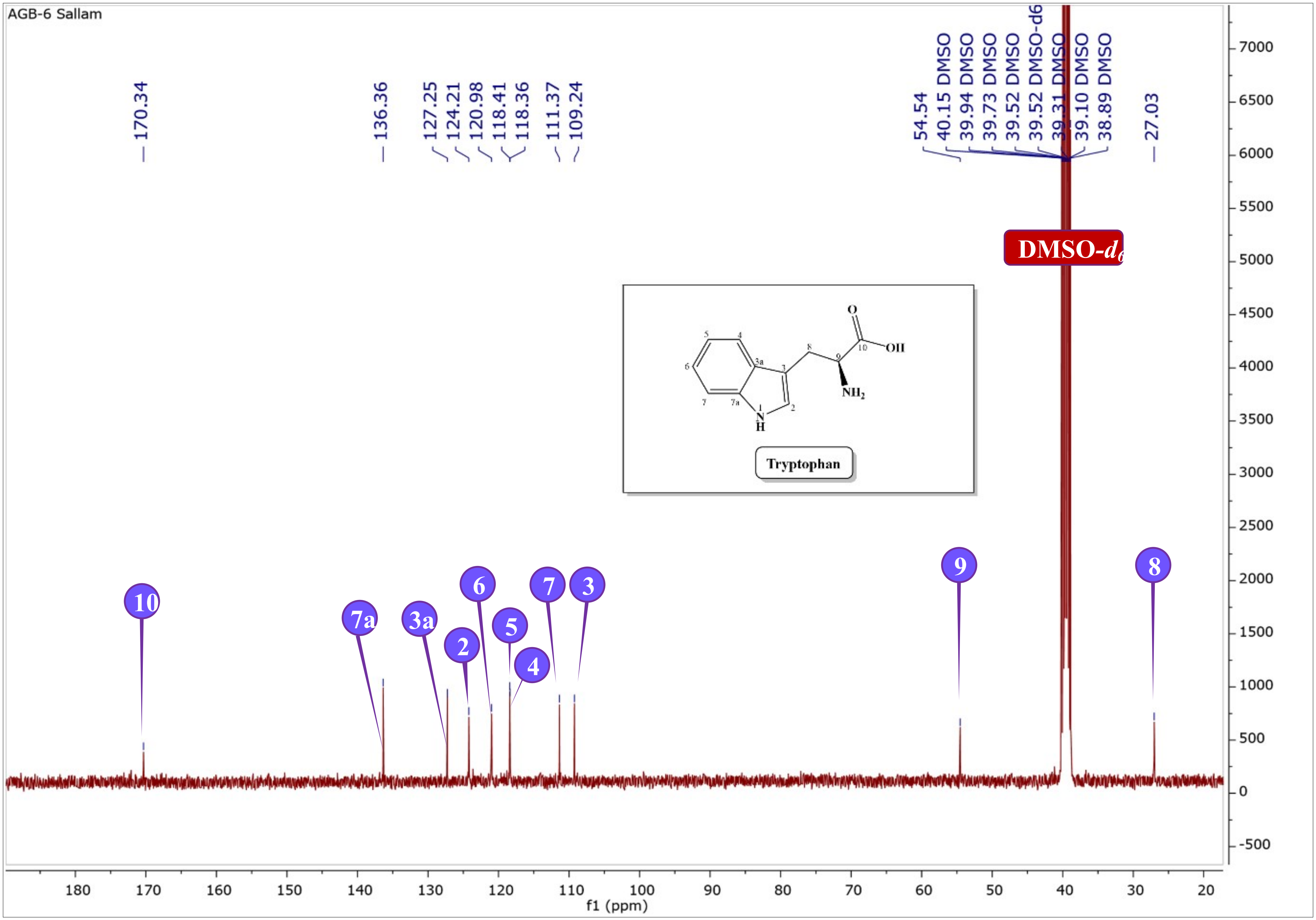

Figure S61: ^{13}C NMR spectrum of compound **12** (DMSO-d_6, 100 Hz).

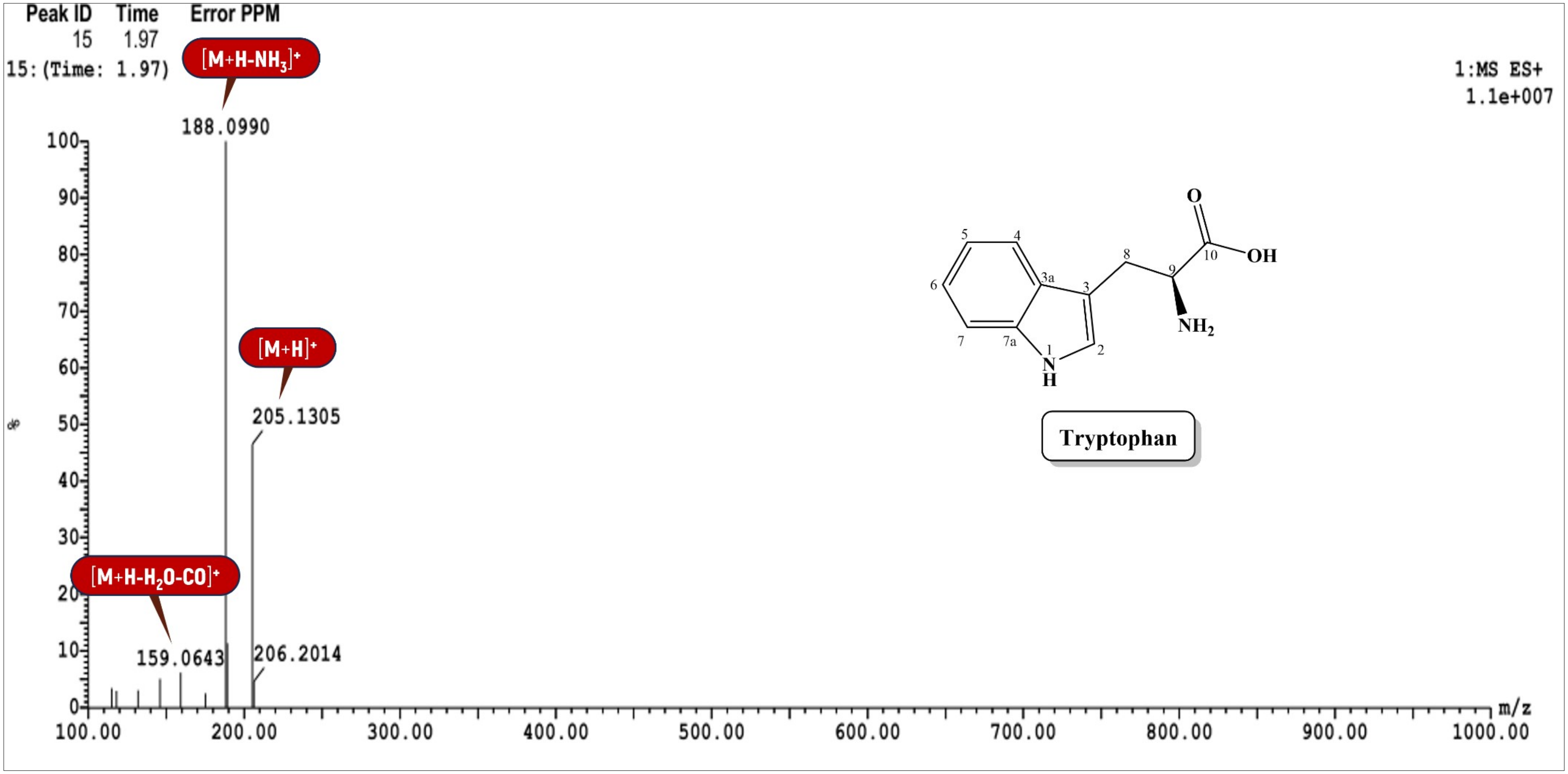

Figure S62: Positive ESI–MS spectrum of compound **12**.

2. Experimental

2.1. General experimental procedures

NMR spectra were recorded on a Bruker FT-NMR Spectrometer (Model: Ascend 400 MHz with Avance III HD console) at 400 (^{1}H) and 100 MHz (^{13}C). Chemical shifts are given in parts per million (ppm) and tetramethylsilane (TMS) was used as an internal standard. All coupling constants (*J*) are given in Hertz. ESI-MS positive and negative ion acquisition mode was carried out using a XEVO TQD triple quadrupole mass spectrometer (Waters Corporation, Milford, MA 01757 U.S.A). Chromatographic separation was achieved on an ACQUITY UPLC BEH C18 column (1.7 µm, 2.1 × 50 mm) with a flow rate of 0.2 mL/min. The mobile phase consisted of solvent A: water with 0.1% formic acid, and solvent B: acetonitrile with 0.1% formic acid. UV absorption spectra were obtained using a Shimadzu UV-1800 double-beam spectrophotometer (Shimadzu, Kyoto, Japan) equipped with UVProbe software v2.43, operating over 190–1100 nm with a 1 nm spectral bandwidth, ±0.1 nm wavelength accuracy, and photometric accuracy of ±0.002 Abs at 0.5 Abs.

2.4. Acid hydrolysis:

Compounds **1**–**4** (5 mg each) were refluxed with 2 M HCl in methanol (5 mL) at 80°C for 2 h. The reaction mixture was evaporated, and the resulting hydrolysate was diluted with distilled water (10 mL) and extracted with EtOAc (3 × 20 mL). The combined EtOAc extracts were evaporated to afford the aglycones, which were identified by co-TLC with authentic kaempferol or quercetin [silica gel, $CHCl_3$–MeOH, 90:10 v/v]. The aqueous layer was neutralized with 2 N KOH and concentrated under reduced pressure at 40°C to a final volume of 1 mL. The sugars were identified by co-TLC with authentic samples [silica gel, EtOAc–MeOH–H_2O–HOAc, 13:3:3:4 v/v].

2.5.1. *In silico* studies

2.5.1.1. Molecular docking:

Protein preparation:

The crystal structure of the DNA–Topo II complex (PDB ID: 3QX3) was retrieved from the Protein Data Bank (https://www.rcsb.org). Initially, the structure of the protein in complex with the co-crystallized ligand (etoposide) was refined by eliminating crystallographic water molecules. Only a single chain, along with the bound ligand, was retained for further studies. Protonation of the selected chain was performed under the following parameters: the electrostatic functional form was set to GB/VI with a 15 Å distance cut-off, a dielectric constant of 2 for the protein, and 80 for the solvent. The Van der Waals functional form was set to 800R3 with a 10 Å distance cut-off. Energy minimization of the protein chain was then conducted using Hamiltonian AM1, followed by optimization with the MMFF94x (Merck Molecular Force Field). The active site for ligand docking and validation was defined as residues located within 5 Å of the co-crystallized ligand.

Ligand preparation:

The 2D chemical structures of compound **1** and the standard compound (Dox) were sketched using ChemBioDraw Ultra 14.0 and saved in MDL-SD format. The ligands were then converted to 3D, protonated, and structurally optimized through energy minimization using the MM2 force field with 10,000 iteration steps at 2 fs intervals. The optimized conformations were subsequently used for molecular docking experiments.

Docking setup and protocol validation:

Validation of the docking protocol was achieved by redocking the co-crystallized ligand (etoposide) into the Topo-II binding pocket and evaluating the root-mean-square deviation (RMSD) of heavy atoms between the redocked and original co-crystallized poses. Once validated, compound **1** was docked into the defined active site following the same protocol. For each docking experiment, 30 poses were generated using the ASE scoring function with rigid receptor refinement. The most favorable binding pose was chosen for further analysis. Docking outcomes were visualized with Discovery Studio (DS) 4.0, and ligand interactions and docking scores were compared against those of the reference compound (Dox).

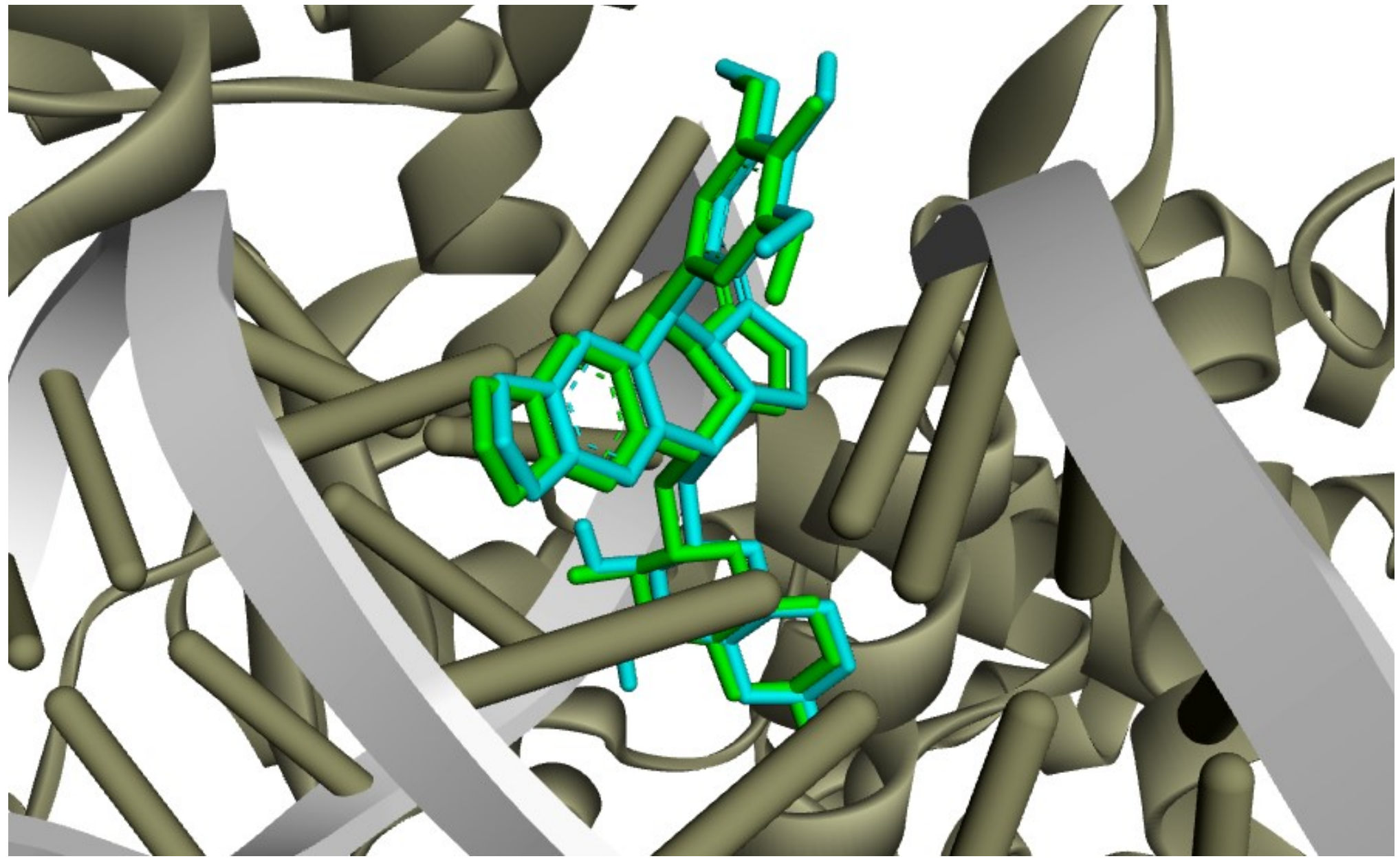

Figure 1: Overlay of the crystallographic (green) and redocked (cyan) poses of etoposide within the DNA–Topo II binding site (PDB: 3QX3); RMSD = 0.491 Å.

2.5.1.2. Molecular dynamics (MD) simulation:

The binding affinity and stability of the ligand–topoisomerase II–DNA complex were evaluated using unbiased molecular dynamics (MD) simulation. A 200 ns trajectory for the system was created using GROMACS 2021 [226]. CHARMM-GUI solution builder was used to prepare starting files [227–230]. To solvate the system, water molecules were used in a 10 nm cubic box with a buffer distance of 1 nm and a transferable intermolecular potential of three points (TIP3P). For neutralization, NaCl ions were added at a concentration of 0.154 M. The CHARMM36m force field was used for the topoisomerase II–DNA complex, TIP3P–water, and ions. Periodic boundary conditions were used in all simulation dimensions. To eliminate steric conflicts, potential energy minimization was first performed using the steepest descent approach. Convergence was achieved when the maximum force applied to an atom was less than 100 kJ/(mol.nm) or after 100,000 steps. The two-stage equilibrium process aimed to achieve thermodynamic equilibrium. The Velocity Rescale thermostat maintained the average temperature in the first stage (NVT ensemble) at 310 K. The Velocity Rescale algorithm was used by the second stage (NPT ensemble) to maintain a temperature of 310 K, and the Berendsen Barostat was used for pressure control at 1 atm [231]. A Parrinello-Rahman barostat was used to regulate pressure at 1 atm, and a Nose-Hoover thermostat was used for temperature control in

the production run (NPT ensemble) [232]. The hydrogen atom bond lengths were maintained with the Linear Constraint Solver (LINCS) method [233]. Using a 1.2 nm cutoff, the Particle Mesh Ewald (PME) approach was used to calculate electrostatic interactions . With time steps of one femtosecond for equilibration and two femtoseconds for production, the leapfrog integrator propagated Newton's equations of motion. 2,000 simulation images were recorded at intervals of 0.1 ns.

After eliminating periodic constraints and restoring protein integrity, trajectories were analyzed using VMD-TK scripts [234]. The ligand system and the root mean square deviation (RMSD) of the backbone of the topoisomerase II–DNA complex were two of the numerous calculations that were performed. In addition to the ligand-protein number, other structural parameters evaluated were the radius of gyration (RoG), the solvent-accessible surface area (SASA), the root mean square fluctuation (RMSF) and the distances between the centers of mass of the ligand, and the protein. Analysis of ligand-amino acid interactions is performed using the Python program Protein-Ligand Interaction Fingerprints (ProLIF) [235] quantified and characterized the frequency and type of interactions in each frame.

2.5.1.3. Binding free energy calculation using MM–GBSA:

The *gmx_MMPBSA* program was used to apply the MM–GBSA (Molecular Mechanics–Generalized Born Surface Area) method to calculate the binding free energies of the ligands [236,237]. Decomposition analysis was performed to identify contributing residues within a 1 nm radius of each ligand. All simulations were conducted at an ionic strength of 0.154 M using solvation parameter *igb* = 5. Internal and external dielectric constants were set to 1.0 and 78.5, respectively. Binding free energies were computed across all 2000 production frames. The net binding free energy, $\Delta G_{\text{binding}}$ (kcal mol−1), is defined as:

$$\Delta G\text{binding} = < G\text{complex} - (G\text{receptor} + G\text{ligand}) > \quad (1)$$

where < > is the mean of the enclosed free energy of the ligand, complex, and receptor throughout the computation frames. We employed the entire route (a total of 2000 frames) in our method. The thermodynamic contributions to the binding free energy are decomposed as:

$$\Delta G\text{binding} = \Delta H - T\Delta S \quad (2)$$

$$\Delta H = \Delta E\text{gas} + \Delta E\text{sol} \quad (3)$$

$$\Delta E\text{gas} = \Delta E\text{ele} + \Delta E\text{vdW} \quad (4)$$

$$\Delta E\text{solv} = E\text{GB} + E\text{SA} \quad (5)$$

$$E\text{SA} = \gamma.\text{SASA} \quad (6)$$

Where:

ΔH = enthalpy change (kcal mol^{-1}).

T = temperature (K); ΔS = entropy change (kcal mol^{-1} K^{-1}).

ΔE_{gas} = gas-phase molecular mechanics energy (kcal mol^{-1}).

ΔE_{ele} = electrostatic energy (kcal mol^{-1}).

ΔE_{vdW} = van der Waals energy (kcal mol^{-1}).

E_{GB} = polar solvation energy from the Generalized Born model (kcal mol^{-1}).

E_{SA} = nonpolar solvation energy estimated from the solvent-accessible surface area (kcal mol^{-1}).

γ = surface tension coefficient (kcal mol^{-1} $Å^{-2}$).

SASA = solvent-accessible surface area ($Å^2$). [238]

2.5.1.4. Principal component analysis:

Mass-weighted covariance matrix (C) and principal component analysis (PCA) were used to evaluate the mobility of the α-carbon of the topoisomerase II–DNA complex [238]. For the combined analysis (for the free energy landscape), the final equilibrium frame of the ligand–topoisomerase II–DNA complex is referenced during the alignment procedure, as well as the final equilibrium frame of each trajectory for individual trajectories. In GROMACS, eigenvectors corresponding to the dominant atom motions were identified by diagonalizing the covariance matrix using *gmx covar*. Eigenvalues showed how much movement there was; The first principal component caused the largest variation, while the contributions of the other components decreased. GROMACS analysis was performed using the *gmx anaeig* command. Using three parameters, we calculated the dimensions of the critical subspace. 1) Using additional eigenvectors, the variance captured by each additional eigenvector was visualized and the cumulative sum of the eigenvalues was calculated. Additionally, 2) creation of a scree plot showed each eigenvalue associated with the relevant eigenvector index. The essential subspace size was given by the index that had the highest slope reduction. Additionally, 3) an evaluation of the eigenvector distribution was carried out. If a distribution other than a Gaussian distribution appeared, this suggested that the corresponding eigenvectors had meaningful dynamics.

We computed the cosine content (*ci*) of each eigenvector of the C matrix, which may take values ranging from 0 (no cosine) to 1 (perfect cosine). The following is the cosine content equation:

$$c_i = \frac{2}{T}\left(\int \cos(i\pi t\, p_i(t)\, dt\right)^2 \left(\int p_i^2(t)\, dt\right)^{-1} \tag{7}$$

Where:

T = total simulation time (ns).

$p_i(t)$ = projection of trajectory onto the i-th eigenvector at time t (nm).

c_i = cosine content, ranging from 0 (no cosine character; meaningful dynamics) to 1 (perfect cosine; diffusive/random motion).

Abnormally large ci values, which represent random motion, are related to insufficient sampling. When the cosine content of the first few PCs is near 1, the behavior of proteins on a large scale is analogous to diffusion. Accordingly, the first 10 PCs were used to calculate their cosine content [239–241].

2.5.1.5. Free energy landscape (FEL):

Understanding the different conformations that a protein can adopt is crucial to understanding its dynamics and function. This can be achieved by exploiting the free energy landscape (FEL) of the protein. This research often uses conformational sampling techniques such as molecular dynamics (MD) simulations, which replicate the inherent temperature fluctuations of proteins over time. Here we used molecular dynamics simulations to reveal the FEL of our protein of interest. To enhance its visual appeal, we projected this complex onto two selected reaction coordinates. The free energy ($G\alpha$) of the protein in a state described by these coordinates is related to the probability of detecting the protein in that state via the exponential relationship e-Gα/kT. The total FEL can be determined using these calculations:

$$G\alpha = -kT\ln\left(\frac{P(q_\alpha)}{P_{max}(q)}\right) \tag{8}$$

Where:

k = Boltzmann constant (1.987×10^{-3} kcal mol^{-1} K^{-1}).

T = simulation temperature (K).

$P(q\alpha)$ = probability density of state $q\alpha$, estimated from a histogram of MD trajectory data.

$P\max(q)$ = probability of the most populated (reference) state.

We derived two-dimensional representations of the free-energy landscapes by considering two separate reaction coordinates: qi and qj. These representations were obtained by analyzing the joint probability distributions, referred to as $P(qi,qj)$, which describe the system [240]. The GROMACS gmx sham command was utilized for this purpose. Equations are referred to in the text as eqn (1)–eqn (8).

2.5.2. *In vitro* studies

2.5.2.1. Topoisomerase II enzyme inhibition assay [242]:

The inhibitory activities of compound **1** (mauritianin) and the reference drug (doxorubicin) against human topoisomerase II were assessed using the Human Topoisomerase II Alpha ELISA Kit (ab288584, Abcam, Cambridge, UK), a 90-min, single-wash sandwich ELISA based on SimpleStep ELISA® technology. This platform employs capture antibodies conjugated to an affinity tag, which is recognized by a monoclonal antibody pre-coated on the plate, allowing the formation of the antibody–analyte sandwich complex in a single step.

For the assay, varying concentrations of mauritianin (0.1–1000 nM) were incubated with Topo II-containing cell lysates under kit-recommended conditions. Following incubation, wells were washed once and the bound complexes were detected using HRP–TMB substrate. The colorimetric reaction was stopped and absorbance measured at 450 nm using a microplate reader.

All assays were performed in triplicate. Percent inhibition was calculated relative to untreated controls, and IC_{50} values were determined by nonlinear regression analysis in GraphPad Prism (GraphPad Software, San Diego, USA). The sensitivity of the assay was 36.55 pg/mL, as per manufacturer's specification. Results are expressed as mean ± standard deviation (SD).

2.5.2.2. Cytotoxicity assay:

A panel of human cell lines was employed to evaluate the cytotoxic activity of the compound **1** (mauritianin). The normal cell lines included human lung fibroblast (WI-38) and human amnion (WISH), while the cancer cell lines comprised hepatocellular carcinoma (HepG-2), epithelioid carcinoma (HeLa), human prostate carcinoma (PC-3), mammary gland carcinoma (MCF-7), colorectal adenocarcinoma (Caco-2), colorectal carcinoma (HCT-116), and breast adenocarcinoma (MDA-MB-231). All cell lines were obtained from ATCC and supplied through the Holding Company for Biological Products and Vaccines (VACSERA, Cairo, Egypt). Doxorubicin was used as a reference anticancer drug for comparison.

The reagents used included RPMI-1640 culture medium, MTT, and dimethyl sulfoxide (DMSO) (Sigma, St. Louis, USA), as well as fetal bovine serum (FBS) (GIBCO, UK). The cytotoxic activity of the tested compounds was assessed using the MTT colorimetric assay [243], which is based on the principle that mitochondrial succinate dehydrogenase in viable cells reduces the yellow tetrazolium salt (MTT) into insoluble purple formazan crystals. The amount of formazan formed is directly proportional to the of metabolically active cells.

Cells were routinely cultured in RPMI-1640 medium supplemented with 10% FBS, 100 U/mL penicillin, and 100 µg/mL streptomycin, and maintained at 37°C in a humidified incubator with 5% CO_2. For the cytotoxicity assay, cells were seeded into 96-well plates at a density of 1.0×10^4 cells per well and allowed to adhere for 48 h under standard culture conditions. Following this period, cells were treated with various concentrations of the tested compounds and incubated for an additional 24 h.

After treatment, 20 µL of MTT solution (5 mg/mL) was added to each well and incubated for 4 h to allow formazan crystal formation. Subsequently, 100 µL of DMSO was added to each well to dissolve the crystals. Absorbance was measured at 570 nm using a microplate reader (EXL 800, USA).

Cell viability percentage was obtained by dividing the absorbance of the treated sample by the absorbance of the untreated control and then multiplying the result by 100. The half-maximal inhibitory concentration (IC_{50}) values were determined by plotting the percentage of cell viability against the logarithm of compound concentration to generate dose–response curves. The IC_{50} values were calculated as the concentration of compound required to reduce cell viability by 50% compared to untreated controls.

Cytotoxic activity of compound 1 against human cell line

**Comp.**	**In vitro Cytotoxicity IC_{50} (µM) •**								
	**WI-38**	**WISH**	**HePG-2**	**MCF-7**	**HCT-116**	**PC-3**	**MDA-231**	**Caco-2**	**HeLa**
**DOX••**	**6.72±0.5**	**8.72±0.7**	**4.50±0.2**	**4.17±0.2**	**5.23±0.3**	**8.87±0.6**	**3.18±0.1**	**12.49±1.1**	**5.57±0.4**
**M-1**	56.20±3.2	61.90±3.4	**14.03±1.3**	**10.28±0.8**	**24.88±1.8**	**29.76±1.8**	**11.66±0.9**	**33.04±2.2**	**18.13±1.4**

• IC_{50} (µM) : 1 – 10 (very strong). 11 – 20 (strong). 21 – 50 (moderate). 51 – 100 (weak) and above 100 (non-cytotoxic)
•• DOX : Doxorubicin

2.5.2.3. Flow cytometry analysis:

Cell cycle distribution:

The effect of compound **1** (mauritianin) on cell cycle progression was investigated in MCF-7 breast cancer cells using propidium iodide (PI) DNA staining. Cells were treated with mauritianin at its previously determined IC_{50} concentration (based on MTT assay results) for 48 h. After treatment, approximately 1×10^6 cells per condition were harvested, washed with cold PBS, and fixed in 70% ethanol at –20°C for at least 12 h to ensure complete permeabilization. Following fixation, cells were washed and incubated with a staining solution containing 50 µg/mL PI (Sigma-Aldrich), 100 µg/mL RNase A (Thermo Fisher), and 0.1% Triton X-100 in PBS for 30 min at 37°C in the dark. Flow cytometric analysis was carried out using a BD FACSAria III (BD Biosciences) equipped with a 488 nm excitation laser, and fluorescence emission was collected with a 617/25 nm bandpass filter. For each sample, at least 20,000 single-cell events were recorded, and doublets were excluded by applying pulse-width versus pulse-area gating. DNA histograms were generated and analyzed using ModFit LT 5.0 software (Verity Software House) with the Dean-Jett-Fox model, which provided quantitative distributions of cells across different phases of the cell cycle.

Apoptosis induction:

The pro-apoptotic effect of compound **1** (mauritianin) against MCF-7 breast cancer cells was further evaluated using Annexin V-FITC/PI dual staining, performed according to the manufacturer's protocol (BD Pharmingen Annexin V-FITC Apoptosis Detection Kit). Briefly, 5×10^5 cells were collected, washed twice with cold PBS, and resuspended in 100 µL of 1× binding buffer containing 5 µL Annexin V-FITC and 5 µL PI (final concentration: 50 µg/mL). Samples were incubated for 15 min at room temperature in the dark, after which 400 µL of binding buffer was added prior to acquisition. Flow cytometric acquisition was performed on the BD FACSAria III, with daily calibration using BD CompBeads to ensure proper compensation of fluorescence overlap. Single-stained controls were included in each experiment to establish compensation matrices, and fluorescence-minus-one (FMO) controls were used to refine gating accuracy. Data were analyzed using FACSDiva 8.0.1 software (BD Biosciences), and cell populations were classified into four quadrants:

- <u>Viable cells:</u> Annexin V–FITC negative / PI negative (lower left quadrant).
- <u>Early apoptotic cells:</u> Annexin V–FITC positive / PI negative (lower right quadrant).
- <u>Late apoptotic cells:</u> Annexin V–FITC positive / PI positive (upper right quadrant).
- <u>Necrotic cells:</u> Annexin V–FITC negative / PI positive (upper left quadrant).

This analysis enabled quantification of apoptotic and necrotic cell populations in response to Mauritianin treatment, thereby providing mechanistic insights into its antiproliferative effects.
